# Highly efficient morpholine-based organocatalysts for the 1,4-addition reaction between aldehydes and nitroolefins: an unexploited class of catalysts

**DOI:** 10.3389/fchem.2023.1233097

**Published:** 2023-08-11

**Authors:** Francesco Vaghi, Giorgio Facchetti, Isabella Rimoldi, Matteo Bottiglieri, Alessandro Contini, Maria Luisa Gelmi, Raffaella Bucci

**Affiliations:** Dipartimento di Scienze Farmaceutiche, DISFARM, Sezione Chimica Generale e Organica “A. Marchesini”, Università degli Studi di Milano, Milan, Italy

**Keywords:** non-coded amino acids, *ß*-amino acids, organocatalysis, morpholine, Michael addition, enamine catalysis

## Abstract

Many studies have demonstrated how the pyrrolidine nucleus is more efficient than the corresponding piperidine or morpholine as organocatalysts in the condensation of aldehydes with electrophiles *via* enamine. Focussing on morpholine–enamines, their low reactivity is ascribed to the presence of oxygen on the ring and to the pronounced pyramidalisation of nitrogen, decreasing the nucleophilicity of the enamine. Thus, the selection of efficient morpholine organocatalysts appears to be a difficult challenge. Herein, we reported on the synthesis of new organocatalysts belonging to the class of *ß*-morpholine amino acids that were tested in a model reaction, *i.e.*, the 1,4-addition reaction of aldehydes to nitroolefins. Starting from commercially available amino acids and epichlorohydrin, we designed an efficient synthesis for the aforementioned catalysts, controlling the configuration and the substitution pattern. Computational studies indeed disclosed the transition state of the reaction, explaining why, despite all the limitations of the morpholine ring for enamine catalysis, our best catalyst works efficiently, affording condensation products with excellent yields, diastereoselection and good-to-exquisite enantioselectivity.

## Introduction

The restriction of raw materials and resources led the organic chemists to change their mindset and design chemical processes based on the “Sustainable Development” concept. Inspired by Nature, since the late 1990s, scientists have laid the groundwork for asymmetric organocatalysis (Ahrendt et al., 2000; List et al., 2000), allowing a green and direct access to highly functionalised chiral products, including important key intermediates in the total syntheses of bioactive compounds (Xiang and Tan, 2020; Han et al., 2021).

At the dawn of organocatalysis, (*S*)-Proline (Pro) was identified as “the simplest enzyme” because of its ability to promote enantioselectivity in different reactions (List et al., 2000; 2001; Movassaghi and Jacobsen, 2002). Since then, plenty of analogues were designed to bypass the Pro limitations as an organocatalyst, such as its poor solubility in organic solvents (Obregón-Zúñiga et al., 2017). Moreover, the introduction of sterically hindered groups allowed the formation of more rigid transition states, leading to better stereo-induction within the studied reaction (Seebach et al., 1985; Seebach et al., 2013; Liu and Wang, 2017). As an example, MacMillan (Ahrendt et al., 2000) and Hayashi–Jorgensen (Hayashi et al., 2007; Reyes et al., 2007) catalysts are at present commercially available compounds for routine enantioselective syntheses. Due to the increasing demand of chiral compounds, the research of new organocatalysts continues to be a hot topic of research.

Recently, our research group reported on the synthesis of non-natural *ß*-amino acids (β-AA) with a constrained heterocyclic core (Oliva et al., 2019), mostly focussing on morpholine *ß*-amino acids (β-Morph-AAs) for different applications (Penso et al., 2012; Bucci et al., 2019; Bucci et al., 2020; Bucci et al., 2021; Vaghi et al., 2020), i.e., from the synthesis of photoluminescent nucleopeptides (Bucci et al., 2020) to their use as inducers of the polyproline helix when inserted in the model’s peptides (Bucci et al., 2019; Bucci et al., 2021; Vaghi et al., 2020).

Being inspired by the use of nitrogen-containing heterocycles in asymmetric synthesis *via* enamine, here, we studied the use of new *ß*-Morph-AAs as very challenging and stimulating organocatalysts. It has already been reported that in comparison to enamines with a pyrrolidine and piperidine core, the morpholine cores are orders of magnitude less reactive. Pyrrolidine enamines are the most reactive due to the higher p-character of the nitrogen lone pair in their five-membered ring, indicating higher nucleophilicity compared to the six-membered piperidine ring. The presence of oxygen in morpholine–enamines further increases the ionisation potential and consequently reduces nucleophilicity compared to piperidine cores (Kempf et al., 2003). Moreover, the most pronounced pyramidalisation of morpholine–enamines, resulting in poor reactivity, should be another limitation of the proposed catalysts (Brown et al., 1978; Schnitzer et al., 2020b).

To test our catalysts, we focussed on a model Michael addition reaction between aldehydes and nitrostyrenes, which is usually promoted by pyrrolidine-based organocatalysts. As the main drawback, except for few examples (Lombardo et al., 2009; Borges-González et al., 2019; Schnitzer et al., 2020a; Schnitzer et al., 2020b), their use requires a 10–20 mol% of the catalyst and an excess of the carbonyl compound (List et al., 2001; Sakthivel et al., 2001; Hayashi et al., 2005; Choudary et al., 2007; Ni et al., 2007; Llopis et al., 2018).

It has to be underlined that some base research on morpholine–enamine to understand the topological rule for C,C-bond-forming processes between prochiral centres was conducted by Seebach and Goliński (1981). On the other hand, the use of morpholine catalysts is, in general, very limited, mostly in terms of reagent conversion. Moreover, to the best of our knowledge, only few examples of chiral morpholine organocatalysts were tested for this reaction, yielding poor diastereo- and enantioselectivity (Mossé et al., 2006; Laars et al., 2009; Laars et al., 2010).

**FIGURE 1:**
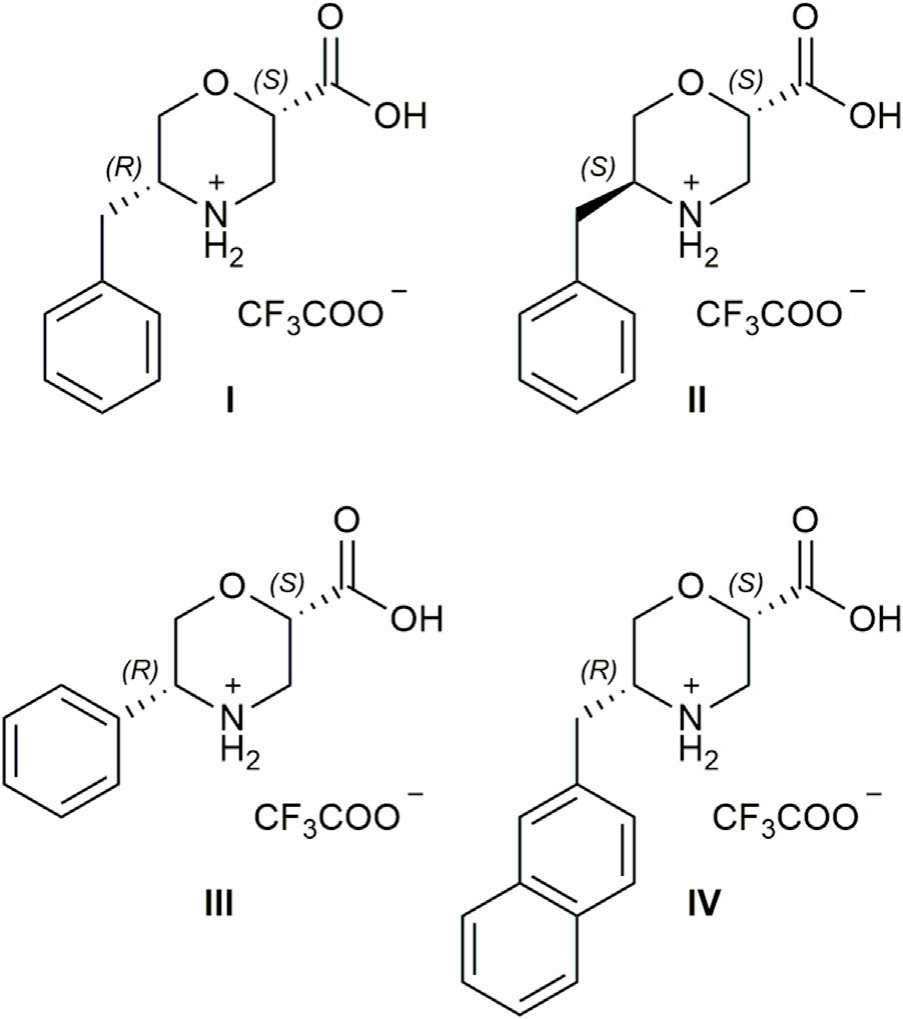
Chemical structure of catalysts **I–IV**.

Starting from inexpensive commercially available *a*-AAs and chiral epichlorohydrin, we designed a straightforward enantioselective synthesis of *ß*-Morph-AAs I–IV (Figure 1) with a different sterically hindered group at C-5, which is derived from the *a*-AA side chain, and a carboxylic function at C-2, which is crucial for the success of this reaction. By playing with different stereochemistries of the two starting materials, it is possible to modulate the formation of *cis* or *trans* isomers of the two substituents and their absolute configuration that will reflect on the stereochemistry of the final compound.

The efficacy of the hindered group, together with the best steric relationship between C-2 and C-5 substituents of morpholine ring, was investigated. Both experimental and computational data confirmed that catalyst **I** has a remarkable ability to control the diastereo- and enantioselectivity of the 1,4-addition reaction between aldehydes and nitroolefins. Despite the already explained limitations of morpholine catalysts with enamine mode of action, different from the majority of the reported organocatalysts, only 1 mol% of **I** and 1.1 eq. of aldehyde are required to reach a quantitative conversion of the reagents. We also proved the crucial role of the carboxylic group; i.e., in the presence of **I** capped as methyl ester under standard conditions, no condensation products were observed after 48 h. Furthermore, excellent diastereoselection was detected (90%–99% *d. e.*), along with the enantioselection ranging from 70% to 99% *e. e.*, depending on the reagents.

## Results and discussion

**SCHEME 1:**
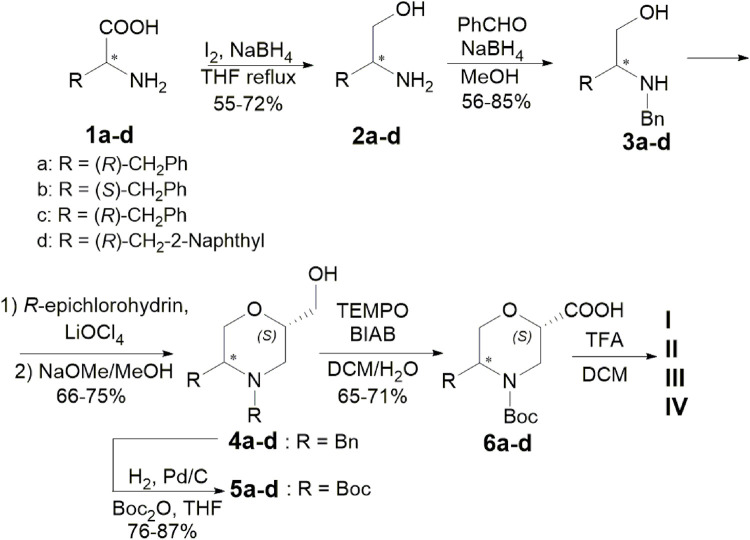
Synthesis of catalysts **I**–**IV**.

5-Substituted *ß*-Morph-AAs were synthesised from commercially available (*R*) or (*S*) *a*-AAs and (*R*)-epichlorohyidrin (Scheme 1). AAs **1** were treated with NaBH_4_ (2.5 eq.) and I_2_ (1 eq.) in refluxing THF, yielding the corresponding amino alcohol **2** (55%–72%). Subsequent reductive amination of **2** with benzaldehyde (1.3 eq.) and NaBH_4_ (3 eq.) in MeOH at r. t. afforded compounds **3** (56%–85%). According to a one-pot procedure reported in the work of Breuning et al. (2007), Morph-derivatives **4** (66%–75%) were obtained by the treatment of **3**, first with *R*-epichlorohydrin (1.3 eq.) in the presence of LiClO_4_ (1.3 eq.) in toluene (60°C) and then with MeONa in MeOH. Using H_2_ and Pd/C (10% loading) in the presence of Boc_2_O (1.05 eq.) in THF, **4** was transformed into the Boc-protected amino alcohol **5** (76%–87%). Oxidation with TEMPO (0.2 eq.) and BIAB (2 eq.) in CH_2_Cl_2_/H_2_O (2:1) provided the desired *ß*-Morph-AAs **6** (65%–71%) and then deprotected yielding catalysts **I**–**IV**, as CF_3_CO_2_H salts, characterised by different stereochemistry and substitution patterns.

In order to demonstrate the potential of our Morph-catalysts, the reaction between butyraldehyde (**7a**, 1 eq.) and trans-β-nitrostyrene (**8a**, 1.5 eq.) was chosen as the model. In principle, it can provide two diastereoisomers as a couple of enantiomers, *i.e.*, (2R*,3S*)- and (2S*,3S*)-2-ethyl-4-nitro-3-phenylbutanals (**9**). To control the diastereo- and enantioselection, several reaction conditions (solvent, temperature, and reaction time) were tested using 1% of catalysts **I**–**IV** (Table 1) in the presence of *N*-methylmorpholine (NMM, 1 mol%) as the base, in order to obtain the free amino group of the catalyst.

**TABLE 1:** Screening of catalysts and reaction conditions for the selected Michael addition.

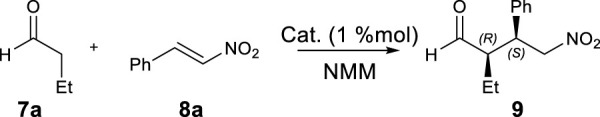							

Entry	Cat. (1%)	Solvent	T (°C)	Conv (%)^a^	Time (h)	*d.e.* (%)^b^	*e.e.* (%)^c^
1	**I**	CHCl_3_/TFE (1/1)	40	>99	12	31	49
2	**I**	ACN/HFIP (1/1)	40	>99	12	65	67
3	**I**	*i*PrOH	40	>99	12	64	55
4	**I**	ACN	40	>99	12	49	31
5	**I**	Toluene	40	>99	12	69	32
6	**I**	CHCl_3_/TFE (1/1)	0	86	12	86	65
7	**I**	ACN/HFIP (1/1)	0	91	12	92	72
8	**I**	*i*PrOH	0	>99	12	87	80
9	**I**	CHCl_3_/TFE (1/1)	−10	88	24	89	60
10	**I**	ACN/HFIP (1/1)	−10	93	24	93	75
11	**I**	*i*PrOH	−10	>99	24	96	90
12	**II**	CHCl_3_/TFE (1/1)	40	>99	12	62	58^d^
13	**II**	ACN/HFIP (1/1)	40	>99	12	70	59^d^
14	**II**	ACN	40	72	12	56	48^d^
15	**II**	*i*PrOH	−10	65	24	85	86^d^
16	**III**	CHCl_3_/TFE (1/1)	40	84	12	72	21
17	**III**	ACN/HFIP (1/1)	40	83	12	70	24
18	**III**	ACN	40	81	12	70	10
19	**III**	*i*PrOH	−10	5	24	99	39
20	**IV**	*i*PrOH	−10	60	24	99	73
21	**IV**	*i*PrOH	−10	70	48	99	67

Reaction condition: **7a** (1.0 eq.)/**8a** (1.5 eq.)/NMM (1 mol%)/Cat (1 mol%).

^a^ Conversion was determined by ^1^H NMR on the crude mixture.

^b^ *d.e.* was determined by ^1^H NMR on the crude mixture since the diastereoisomers are hardly separable by flash chromatography.

^c^ *e.e.* was determined by chiral HPLC analysis in comparison with the authentic racemic material.

^d^ (2*S*,3*R*)-**9** was formed as the main isomer. TFE, trifluoroethanol; HFIP, 1,1,1,3,3,3-hexafluoro 2-propanol.

Catalyst **I** was selected as the first catalyst to predominantly yield the *syn* (2*R*,3*S*)-adduct **9**. Different solvents were screened (40°C, 12 h; entries 1–5, Table 1). Mixtures with fluorinated alcohols were also tested for their peculiar features, being known as efficient additives in Michael additions with proline as the catalyst (Pellissier, 2021).

In general, an excellent conversion of the reagents was achieved, revealing the use of alcoholic solvents or co-solvent beneficial for diastereoselection. These solvents were, thus, selected, and additional studies were performed, decreasing the temperature (0°C, 12h, entries 6–8; −10°C, 24 h, entries 9–11; Table 1). *i*PrOH was found to be the best solvent at −10°C (entry 11, Table 1), giving the expected product **9** with quantitative conversion and higher diastereo- (96% *d. e.*) and enantioselectivity (90% *e. e.*). This result agrees with the computational analysis that predicted the importance of protic solvents for the stabilisation of the transition state (see the following sections).

Catalyst **II**, having the opposite configuration at C-5, was then tested (entries 12–15, Table 1), which resulted to be less efficient; *i.e.*, the reaction reached a quantitative conversion but only at 40°C, giving lower *d. e.* and *e. e.* (entries 12–14, Table 1). Operating in *i*PrOH at −10°C, 85% *d. e.*, and 86% *e. e.* was indeed found but with a modest conversion (entry 15, Table 1). Interestingly, the inversion of the configuration at C-5 of **II** with respect to **I** allows the obtainment of *syn* (2*S*,3*R*)-adduct as the main enantiomer (entries 12–15, Table 1).

Since the (2*S*,5*R*)-stereochemistry of **I** resulted to be the most efficient, we chose to evaluate the effect of the group at C-5 by selecting phenyl and CH_2_-2-naphtyl groups, considering their limited freedom and increased bulkiness, respectively. The phenyl group in **III** at 40°C caused a loss in the conversion rate and stereocontrol (entries 16–18, Table 1). Moreover, operating in *i*PrOH at −10°C, only traces of the desired compound were detected (entry 19, Table 1). On the other hand, operating at −10°C for 24 h and using the more hindered catalyst **IV** (entries 20–21, Table 1), 60% conversion, high level of diastereoselection (99% *d. e.*), and satisfactory enantioselection (73% *e. e.*) were reached. By increasing the reaction time, the conversion was slightly increased but with the loss of enantioselectivity (entry 21, Table 1).

In summary, the C-5 benzyl group of **I**, *cis* with respect to the C-2 carboxylic function, induces an excellent diastereo- and enantioselectivity operating in *i*PrOH at −10°C. Thus, with only 1% of the catalyst and only 1:1.5 ratio of **7a:8a**, excellent conversion was observed. By using the aforementioned best conditions and catalysts, a series of aldehydes **7b–g** and nitroolefins **8b–d** were screened to expand the scope of the reaction (Table 2). To reach the right balance between conversion, *d. e.* and *e. e.*, different attempts were carried out, using different amounts of catalyst, reaction times, and temperatures. The best results are summarised in Table 2 (additional results are described in SI, including two examples with aliphatic nitroolefins that gave poor results in terms of *d. e.* and *e. e.*).

**TABLE 2:** Conjugate addition reactions between aldehydes **7a–g** **and nitroolefins 8a–d catalysed by catalyst** **I**.

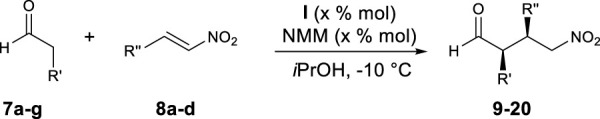								

Entry	Product	Aldehyde 7	Nitroolefin 8	Catalyst % conv (%)^a^	Time (h)	Yield (%)^b^	*d.e.* (%)^c^	*e.e.* (%)^d^
1	**9**	**a**: R’ = Et	**a**: R^’‘^ = Ph	1	24	92	96	90
				>99				
2	**10**	**b**: R’ = Me	**a**: R^’‘^ = Ph	0.5^e^	24	95	94	73
				>99				
3	**11**	**c**: R’ = *n*Pr	**a**: R^’‘^ = Ph	1	48	86	99	99
				90				
4	**12**	**d**: R’ = *i*Pr	**a**: R^’‘^ = Ph	1	48	58	99	95
				68				
5	**13**	**e**: R’ = *n*Bu	**a**: R^’‘^ = Ph	1	48	52	99	87
				60				
6	**14**	**f**: R’ = CH_2_Ph	**a**: R^’‘^ = Ph	1	48	38	99	88
				40				
7	**14**	**f**: R’ = CH_2_Ph	**a**: R^’‘^ = Ph	5	48	72	89	82
				80				
8	**15**	**7g**: cyclopentylaldheyde	**a**: R^’‘^ = Ph	5	48^f^	50	-	35
				53				
9	**16**	**b**: R’ = Me	**b**: R^’‘^ = *p*OMe-Ph	1	48	87	91	80
				>99				
10	**17**	**c**: R’ = *n*Pr	**b**: R” = *p*OMe-Ph	5	48	70	98	70
				74				
11	**18**	**b**: R’ = Me	**c**: R” = tiophenyl	1	48	82	89	67
				>99				
12	**19**	**b**: R’ = Me	**d**: R” = *p*Cl-Ph	1	48	87	93	77
				90				
13	**20**	**c**: R’ = *n*Pr	**d**: R” = *p*Cl-Ph	1	48	55	94	74
				60				

Reaction condition: aldehyde 7(1.0 eq.)/nitrostyrene 8 (1.5 eq.)/catalyst **I**/NMM (x mol%, according to the amount of catalyst) and iPrOH, −10°C.

^a^ Conversion was determined by ^1^H NMR on the crude mixture.

^b^ The yield was calculated after flash chromatography.

^c^ *d.e.* was determined by ^1^H NMR on crude since the diastereoisomers are hardly separable by flash chromatography.

^d^ *e.e.* were determined by chiral HPLC analysis in comparison with the authentic racemic material.

^e^ The same results were obtained with 1% of the catalyst.

^f^ The reaction was performed at 40°C.

Elongating the alkyl chain of the aldehyde, a decrease in conversion was observed, while a growth of *e. e.* was obtained with excellent *d. e.* (up to 99%, entries 1–4, Table 2).

On the other hand, compound **13** (entry 5, Table 2) was obtained with an *e. e.* slightly lower than 90% probably because of the multi-degree of freedom arising from the alkyl chain of the hexanal. To obtain compound **14** from phenylpropionaldehyde (**7f**) with satisfactory conversion, the amount of the catalyst was increased to 5% (comparing entries 6 and 7, Table 2). With the hindered cyclopentylaldheyde (**7g**), a similar amount of catalysts was needed, but operating at 40°C. A low conversion along with a moderate enantioselectivity was observed (entry 8, Table 2). Finally, we focussed on the reactivity of nitrostyrenes **8** containing an electron-rich (*i.e.*, 4-MeOPh, thiophenyl; entries 9–11, Table 2) or electron-poor (*i.e.*, 4-ClPh; entries 12–13, Table 2) aromatic moiety that were matched with **7b** and **7c**. With an exception (entry 10, Table 2), the reaction works with 1% of the catalyst. As a general trend, the more electron rich the aryl substituent in compounds **8** is, the higher is the conversion (comparing entries 9 with 12 and entries 10 with 13, Table 2), and in all cases, high *d. e.* and satisfactory *e. e.* were detected (*d.e.* > 89% and *e. e.* 67%–80%).

## Computational analysis of the reaction mechanism

**FIGURE 2:**
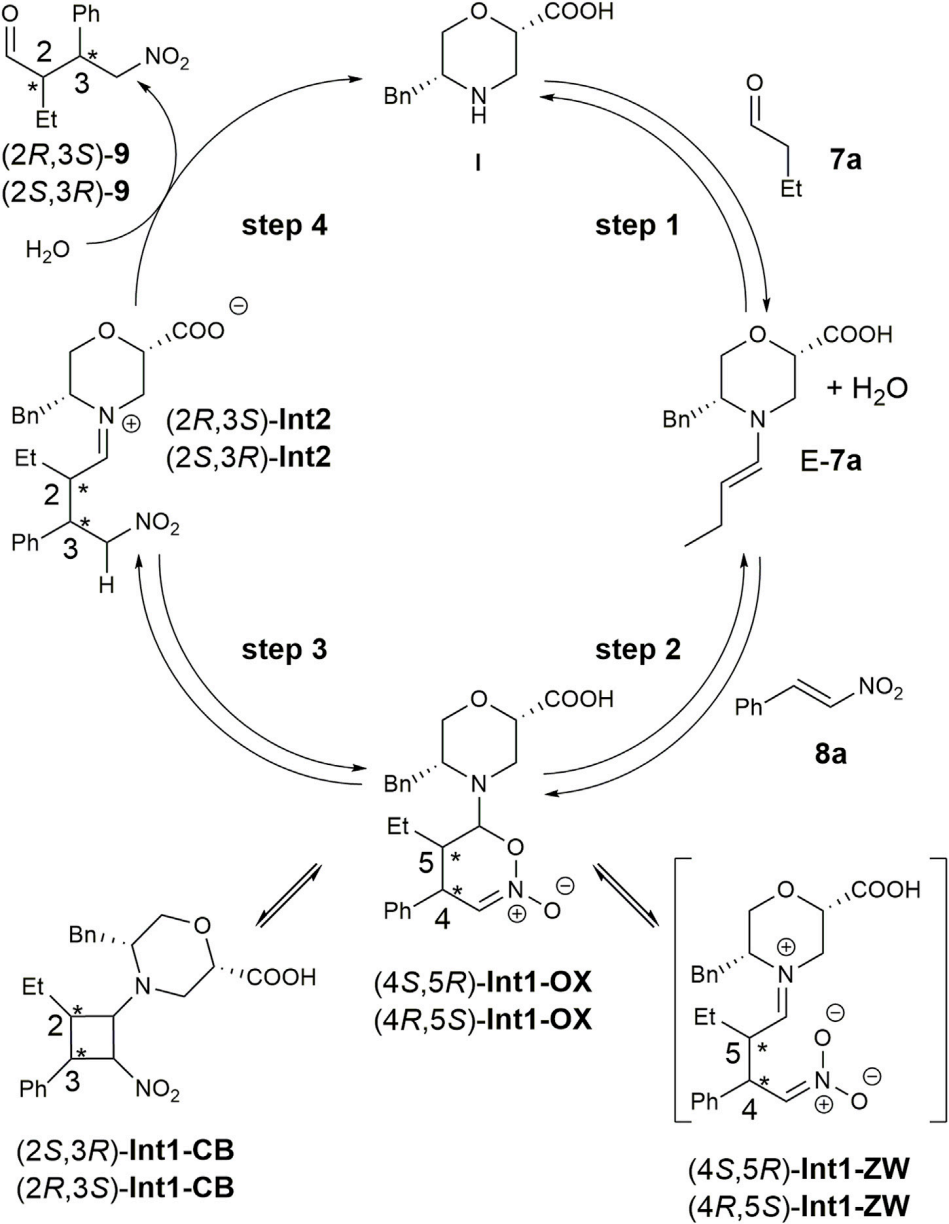
Reaction mechanism for the Michael addition of butanal **7a** to nitrostyrene **8a** in the presence of catalyst **I.**

The mechanism for the addition of aldehydes to nitroalkenes through enamine catalysis was previously analysed both theoretically and experimentally (Sahoo et al., 2012; Földes et al., 2017). Two main hypotheses were carried out for the addition: the first implies the formation of a zwitterion (**Int1-ZW**-like, Figure 2) derived from the addition of the enamine to the *ß*-carbon of the nitroalkene. The second one suggests cycloaddition, leading to dihydrooxazine oxide (**Int1-OX**-like, Figure 2) in equilibrium with a cyclobutane species (**Int1-CB**-like, Figure 2). The dihydrooxazine oxide intermediate can be protonated at the *a*-carbon with respect to the nitro group and evolve to the imino intermediate (**Int2**-like, Figure 2) that is hydrolysed to the final product.

To elucidate the possible role of our catalyst, we decided to model the equilibrium steps described in Figure 2 by using consolidated density functional theory (DFT) methods (Gassa et al., 2010; Grimme et al., 2010; Sahoo et al., 2012; Giofrè et al., 2021).

We initially performed a conformational search on the hypothesised zwitterion **Int1-ZW** at the molecular mechanic level, considering both diastereoisomers. All conformations within 3.0 kcal/mol were optimised at the DFT level, but all simulations either failed or converged into the **Int1-OX** intermediate (Figure 2) for both (4*R*,5*S*) and (4*S*,5*R*) stereoisomers. These results, together with the lack of experimental evidence of the zwitterion intermediate, suggested that the reaction can directly proceed with a cycloaddition-like mechanism.

Consequently, we modelled both the **Int1-OX** and **Int1-CB** intermediates, considering both the 4*S*,5*R*/4*R*,5*S* or 2*S*,3*R*/2*R*,3*S* stereoisomers, respectively (Figure 2), that were subjected to a conformation search using molecular mechanics. Considering that a transient stereocentre is formed at the C-bearing morpholine group, both configurations were evaluated, and the most stable stereoisomer was further considered. All conformations within a range of 3 kcal/mol were optimised by DFT, and energy was calculated by considering *i*PrOH solvent effects and empirical correction for dispersive interactions (Grimme et al., 2010).

**FIGURE 3:**
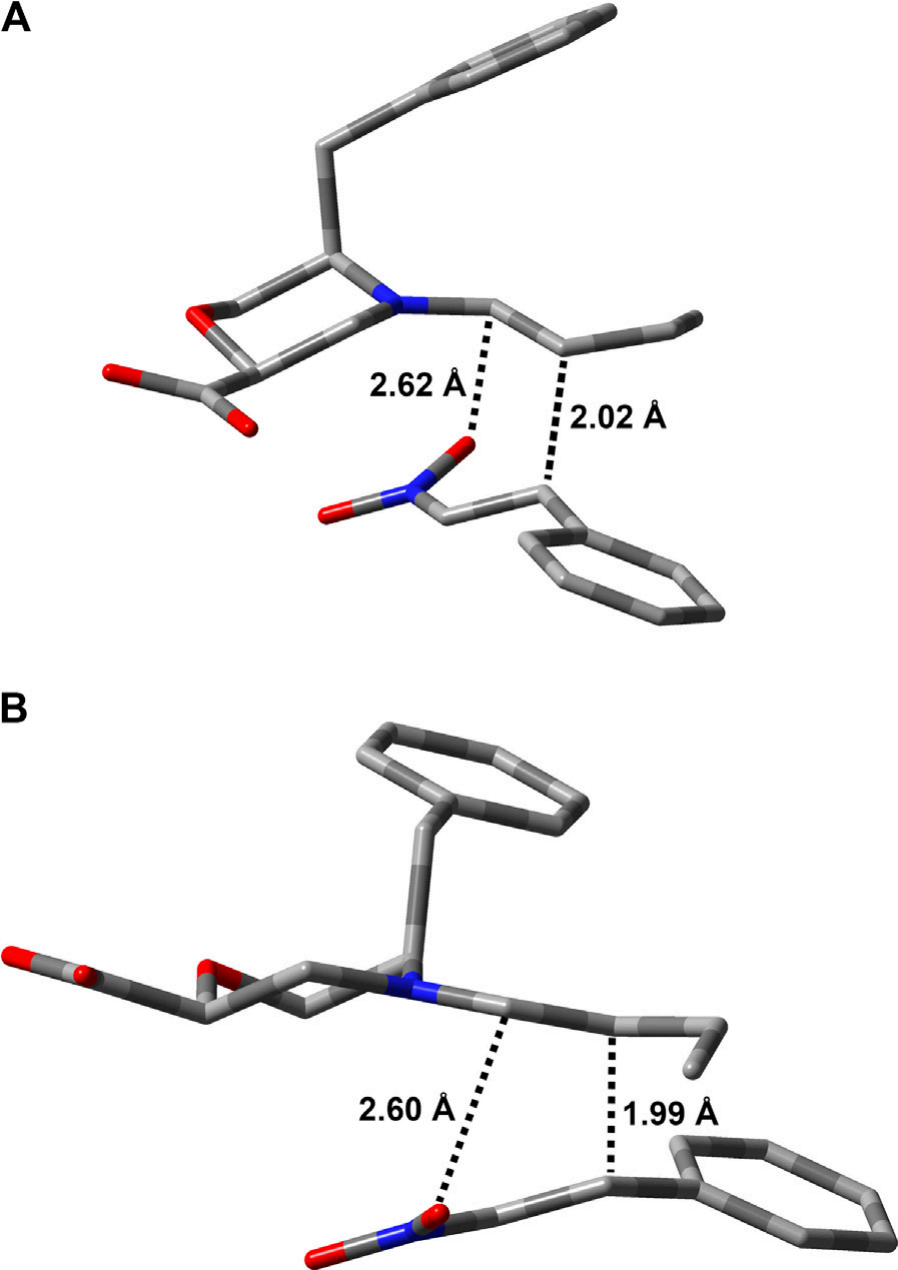
Lowest-energy geometries for **TS1** structures leading to **(A)** (*S,R*)- and **(B)** (*R,S*)-**Int1OX**. Hydrogens are omitted for clarity. Distances between the reacting atoms are reported in Å.

For both stereoisomers, the most stable conformation of the **Int1-OX** intermediate was used to model the transition state (TS1) for the attack of the enamine *E*-**7a** to the nitrostyrene **8a**. The lowest-energy transition states (**TSs)** are shown in Figure 3.

**SCHEME 2:**
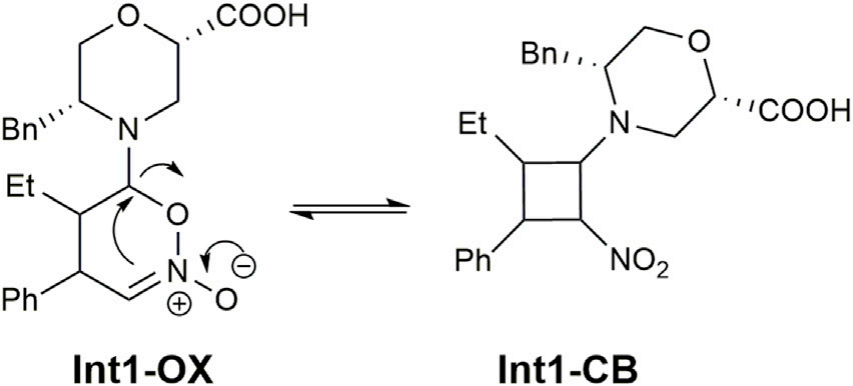
Interconversion between **Int1-OX** and **Int1-CB**.

The geometries of the two **TS1** stereoisomers indicate that the reaction formally occurs as a [4 + 2] cycloaddition directly leading to the **Int1-OX** intermediates. This has been confirmed by intrinsic reaction coordinate (IRC) calculations that showed **TS1** connecting the activated complex between *E*-**7a** and nitrostyrene **8a** to **Int1-OX** (Supplementary Figures S1, S2, SI). A TS directly leading to **Int1-CB** was not located, even if experimental evidence shows that **Int1-CB** is often the dominant species (Burés et al., 2011; Sahoo et al., 2012). We hypothesised that the equilibrium between **Int1-OX** and **Int1-CB** occurs through a concerted ring opening/closure, according to Scheme 2. Despite several attempts, a unique TS for this equilibrium reaction was not found. However, since **Int1-CB** can be considered a dead end in the reaction mechanism (Sahoo et al., 2012), we focussed on identifying TSs that are relevant to step 3 (Figure 2).

**FIGURE 4:**
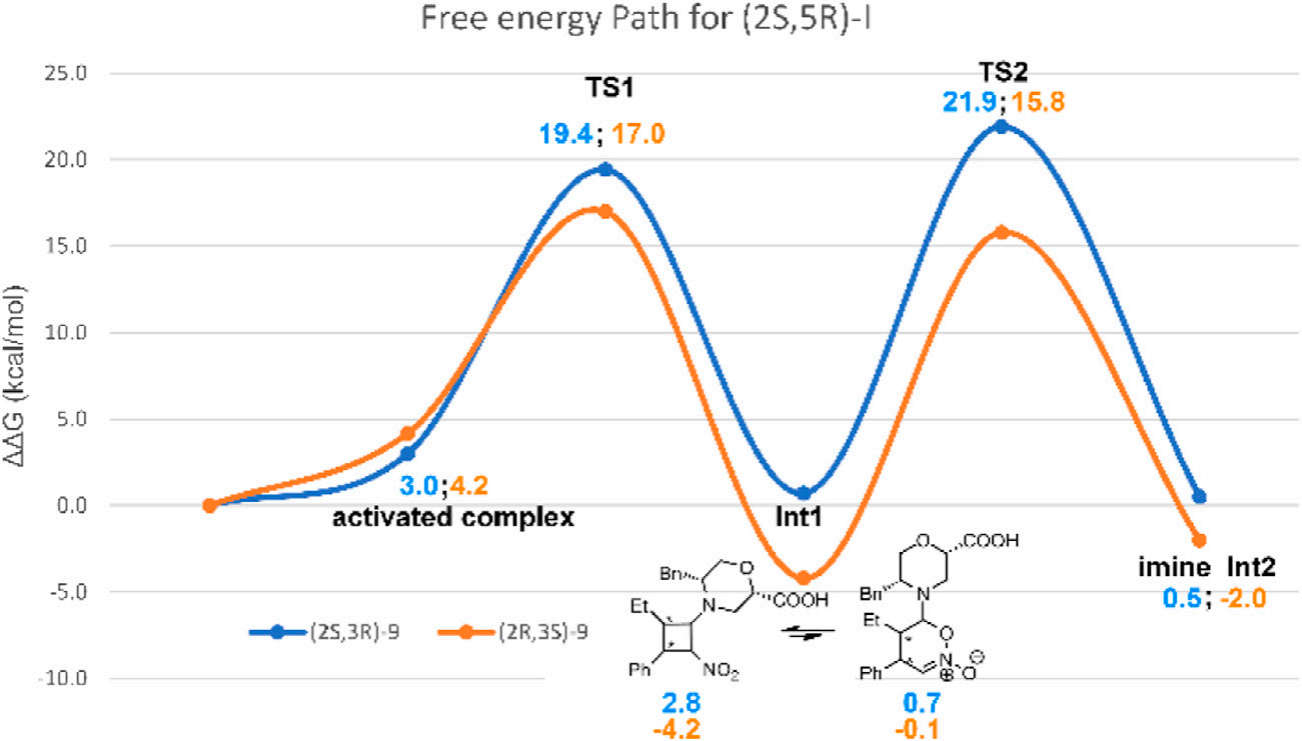
Free-energy path for the reaction of **7a** and **8a** in the presence of catalyst **I**. Relative solution-phase Gibbs free energies (kcal/mol) with respect to reactants are reported.

**FIGURE 5:**
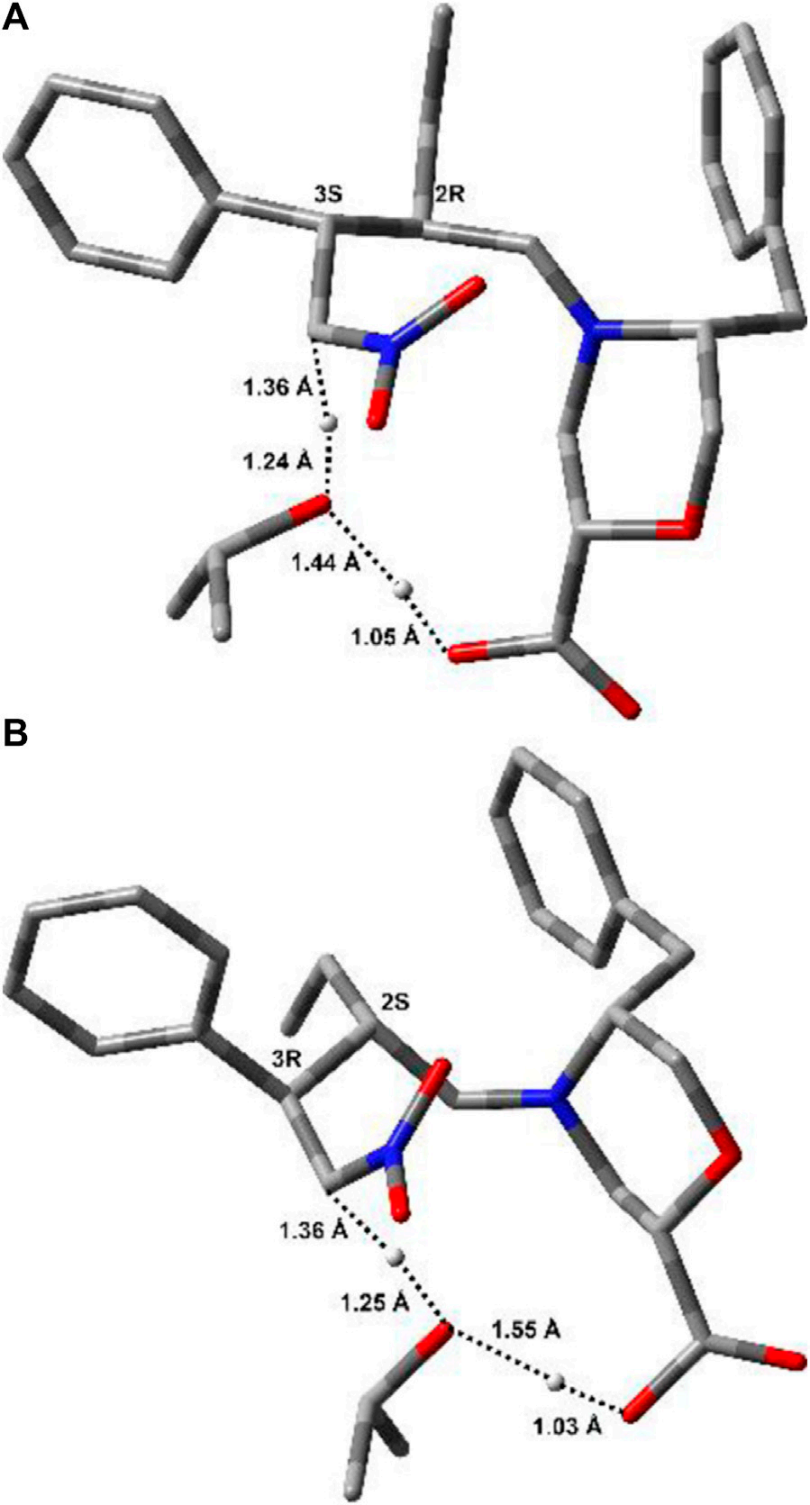
Lowest-energy geometries for (2*R*,3*S*)-**TS2 (A)** and (2*S*,3*R*)-**TS2 (B)**. Non-relevant polar hydrogens are omitted for clarity. Distances (Å) related to the H-transfer are also reported.

Thus, **TS2** was modelled by starting from the lowest-energy structure of **Int1-OX**. It was shown that the protonation step triggering the ring opening in step 3 was the reaction rate determining step (rds) (Sahoo et al., 2012). We, thus, hypothesised that the carboxylic group of catalyst **I** might play a key role in controlling the enantioselectivity observed in this study, as confirmed by experimental data (mentioned previously). We also hypothesised that *i*PrOH, used as a solvent, might bridge the H-transfer between the carboxylic group of the morpholine moiety and Cα-NO_2_. Thus, one molecule of *i*PrOH was explicitly considered in **TS2** geometries for both stereoisomers. Several conformations were evaluated for each **TS2**, and both the *E* and *Z* configurations were considered for the imino group of **Int2**. IRC calculations were conducted to confirm that **TS2** connects **Int1** to **Int2** (Supplementary Figures S1, S2, SI). To compute that activation barriers and reaction energies are comparable with those reported in previous computational studies (Sahoo et al., 2012), all the selected stationary points were reoptimised using the ωB97X-D functional and 6-311G (d,p) basis set, including the solvent effect for *i*PrOH. More accurate single-point energies were then computed at the same level of theory by using 6-311++G (3df,3pd), as suggested by Sahoo et al. (2012). The obtained free energies were then used to draw the reaction path represented in Figure 4. The most stable geometries for (2*R* ,3*S*)- and (2*S*,3*R*)-**TS2** are shown in Figure 5. We can observe that the energy path relative to the formation of the (2*S*,3*R*)-**9** enantiomer is characterised by higher activation free energy barriers (ΔΔG^‡^) for both **TS1** and **TS2**, compared to the favoured (2*R*,3*S*)-**9** enantiomer. Among the two energy paths, the greatest difference in ΔΔG^‡^ is observed for **TS2**, where the protonation step occurs concerted to the ring opening (Figures 4, 5). This step is then confirmed as the r.d.s and the path leading to the isolated (2*R*,3*S*)-**9** as the kinetically favoured path. Interestingly, the **Int1** intermediate results as the global minimum on both the free energy (Figure 4) and enthalpy path (Supplementary Figure S3, SI), suggesting that the hydrolysis of the **Int2** intermediate is the non-equilibrium step that drives the reaction towards the final product.

**TS2** geometries (Figure 5) evidenced the role of the *ß*-Morph carboxylic acid in self-catalysing the proton transfer from *i*PrOH to Cα-NO_2_. This role was also confirmed experimentally; i.e., the reaction between **7a** and **8a** catalysed by the methyl ester of **I** was performed under standard conditions, but no condensation products were observed after 48 h.

Interestingly, both **TS2** structures share similar geometrical parameters related to the H-transfer (see distances in Figure 5). However, the most relevant difference that could be associated to the greater stability of (2*R*,3*S*)-**TS2**, as compared to (2*S*,3*R*)-**TS2**, is the configuration at the imino group of the forming **Int2** product (Supplementary Figure S1, SI). Indeed, the opening of the dihydrooxazine oxide ring led to the *E*-configuration for (2*R*,3*S*)-**TS2**, while the more hindered *Z*-configuration is obtained from (2*S*,3*R*)-**TS2** (Schnitzer et al., 2020a). To confirm this finding, the corresponding *Z* and *E*-**TSs** were also located for (2*R*,3*S*)-**TS2** and (2*S*,3*R*)-**TS2**, but higher energies were obtained in both cases (Supplementary Figure S4, SI).

## Conclusion

In conclusion, we presented here a new class of *ß*-AAs with a morpholine core prepared by a straightforward synthesis, from commercially available *a*-AAs and chiral epichlorohydrin, that can control the configuration of substituent patterns. Despite the known limitations of the morpholine ring during enamine catalysis, our results provided the first evidence on the actual effectiveness of this chiral catalyst that works efficiently in the selected 1,4-addition model reaction between aldehydes and nitroolefins, thanks to the presence of a carboxylic moiety in position *ß* to the amine. Experimental data have proven that the reaction goes with excellent conversion and diastereoselection and satisfactory to excellent enantioselection, depending on the substitution pattern of the two reagents. It is to be noted that only 1% of the catalyst and a 1/1.5 ratio of **7**/**8** is needed, *i*PrOH being the key solvent. These results were supported by a theoretical study on the best catalyst, evidencing the role of the *ß*-Morph carboxylic group in self-catalysing the protonation of the dihydrooxazine oxide intermediate, which is generally considered the rate limiting step of this class of reactions. Experiments confirmed this observation, laying the groundwork for further optimisation of the catalyst and opening its general use for a plethora of asymmetric syntheses.

## Experimental

### Computational methods

The structures of the enamine *E*-**7a** and the dihydrooxazine oxide intermediates (4*S*,5*R*)- and (4*R*,5*S*)-**Int1** were initially constructed using MOE 2020.0901 software (Molecular Operating Environment, 2023). Geometries were minimised and then subjected to a conformational search using the MMFF94x force field (Halgren, 1996) and the Born solvation model for water, since no Born implicit solvent model was observed for *i*PrOH in MOE (Molecular Operating Environment, 2023). All geometries within the 3 kcal/mol interval were successively optimised by DFT using the method described hereafter. Only the lowest-energy structures were further considered. **TS1** structures were originally obtained by modifying the corresponding dihydrooxazine oxide. All structures of reactants, TSs, and products were initially optimised at the mPW1B95/6-31G* level (Zhao and Truhlar, 2004). Frequency calculations were then performed at the same level to confirm the stationary points as minima (0 imaginary frequencies) or TSs (1 imaginary frequency corresponding to the vibration of the forming/breaking bonds). Single-point energy calculations were then performed at the mPW1B95/6-311+G** level, including the GD3 empirical correction for dispersive interactions (Grimme et al., 2010) and the solvent effects for *i*PrOH with the CPCM solvation model (Cossi et al., 2003). Several alternative geometries were constructed and optimised for each TS, and only the lowest-energy structures were further considered. IRC analyses were conducted starting from each TS and following the reaction path in both the “forward” and “reverse” direction. Fifty points on the reaction path were requested for each IRC calculation that was conducted at the same level of theory used for geometry optimisation.

To compute a more reliable activation and reaction energies, as well as to provide a direct comparison with the energies computed for similar reactions previously (Sahoo et al., 2012), all the selected stationary points were reoptimised using the range-separated ωB97X-D functional that includes empirical atom–atom dispersion corrections (Chai and Head-Gordon, 2008). The triple-split valence 6-311G (d,p) basis sets were adopted in geometry optimisations and frequency calculations, while single-point energies were computed on the optimised structure using the 6-311++G (3df, 3pd) basis set. The CPCM solvent model for *i*PrOH was used both in optimisations and frequency calculations and in the single-point calculations. Gaussian16 software was used for all calculations (Frisch et al., 2016).

### General information

Chemicals were purchased from Sigma-Aldrich and were used without further purification. Mass spectra were recorded on an LCQESI MS and LCQ Advantage spectrometer from Thermo Finningan and an LCQ Fleet spectrometer from Thermo Scientific. The NMR spectroscopic experiments were carried out either on Varian MercuryPlus 300 MHz (300 and 75 MHz for ^1^H and ^13^C, respectively) or Bruker Avance I 400 MHz spectrometers (400 and 101 MHz for ^1^H and ^13^C, respectively). Optical rotations were measured on a Perkin–Elmer 343 polarimeter at 20°C (concentration in g/100 mL). Chemical shifts (*δ*) are given in ppm relative to the CHCl_3_ internal standard, and the coupling constants *J* are reported in Hertz (Hz).

Enantiomeric excess was monitored by HPLC with a Merck Hitachi L-7100 HPLC System equipped with a UV6000LP detector and Chiral column (Chiralcel AD, OD-H, and IC). Spectroscopic analyses for each compound are reported in SI.

### General procedure for amino alcohols 2a–d synthesis

A three-neck round-bottom flask was fitted with a magnetic stirring bar and a reflux condenser. The remaining neck was sealed with a septum and nitrogen line attached. The flask was charged with sodium borohydride (1146.3 mg, 30.3 mmol) in THF (0.2 M). Amino acid **1** (12.1 mmol) was added in one portion, and the flask was cooled to 0°C in an ice bath. A solution of iodine (3071.1 mg, 12.1 mmol) dissolved in THF (80 mL, 0.15 M) was slowly dropped over 30 min. After the addition of iodine was completed and gas evolution ceased, the flask was heated to reflux for 18 h. The reaction was cooled to r. t., and MeOH (30 mL) was added cautiously until the mixture became clear. After stirring (30 min), the solvent was removed, yielding a white paste, and then dissolved in aqueous KOH (20%, 24 mL). The solution was stirred for 4 h and extracted CH_2_Cl_2_ (3 × 15 mL). The organic layers were dried over Na_2_SO_4_ and concentrated under reduced pressure, yielding a white semisolid. The crude material was crystallised from toluene to yield the final amino alcohol **2** as colourless crystals.

### General procedure for benzyl-amino alcohols 3a–d synthesis

A solution of amino alcohol **2** (6.5 mmol) and benzaldehyde (902.0 mg, 8.5 mmol) in absolute MeOH (0.3 M, 30 mL) was stirred at 20°C for 2 h, NaBH_4_ (741.5 mg, 19.6 mmol) was added at 0°C, and the reaction mixture was left stirred for 1 h CH_2_Cl_2_ (20 mL) and saturated aq. NH_4_Cl (30 mL) was added, and the layers were separated. The aqueous layer was extracted with CH_2_Cl_2_. The combined organic layers were washed with brine and dried with Na_2_SO_4_, and the solvent was removed under reduced pressure. The crude material was purified by flash column chromatography (*n*hexane/AcOEt, from 0% to 100%), yielding pure compound **3** as a white solid.

### General procedure for benzyl-morpholine amino alcohols 4a–d synthesis

A solution of the amino alcohol **3** (4.0 mmol) in absolute toluene (0.3 M, 13.4 mL) was treated with (*R*)-epichlorohydrin (490.3 mg, 5.3 mmol) and LiClO_4_ (563.9 mg, 5.3 mmol). After 24 h at 60°C, MeONa (545.6 mg, 10.1 mmol) in MeOH (25%v/v) was added and stirring was continued for 24 h. The reaction mixture was quenched with a saturated aq. NH_4_Cl (12 mL), and the aqueous layer was extracted with AcOEt (3 × 10 mL). The combined organic layers were washed with brine and dried with Na_2_SO_4_, and the solvent was removed under reduced pressure. Chromatographic purification (silica gel; Et_2_O/hexanes, 1:1) gave compound **4** as a colourless oil.

### General procedure for Boc-morpholine amino alcohols 5a–d synthesis

Operating in a round-bottom flask equipped with a magnetic stirrer, compound **4** (1 eq.,1.7 mmol) was dissolved in THF (0.1 M, 17 mL). Boc_2_O (401.6 mg, 1.84 mmol) and Pd/C (931 mg, 10% loading) were added to the solution. The suspension was stirred under H_2_ (1 atmosphere) at 25°C. After 24 h, the mixture was filtered on the Celite pad. The solvent was evaporated, and the yellow oil was dissolved in CH_2_Cl_2_ (5 mL) and washed with a solution of KHSO_4_ (5%, 5 mL) and a saturated solution of NaCl (6 mL). The organic layer was dried over Na_2_SO_4_, filtered, and concentrated in vacuum. The purification of the crude by flash chromatography (*n*hexane/AcOEt, 1:1) yielded product **5a–d** as a colourless oil.

### General procedure for Boc-morpholine amino acids 6 synthesis

To a vigorously stirred solution of Boc-morpholine amino alcohol **5** (0.33 mmol) in CH_2_Cl_2_/H_2_O (2:1; 0.15 M, 2 mL), TEMPO (11.0 mg, 0.07 mmol) and BIAB [(diacetoxyiodo)benzene, 225.4 mg, 0.7 mmol] were added at 0°C. After 6 h, the reaction was quenched with MeOH (2 mL), and the mixture was evaporated to dryness. Silica gel column chromatography (CH_2_Cl_2_/MeOH, 20:1) yielded Boc-morpholine amino acid **6** as a colourless oil.

### General procedure of morpholine *ß*-amino acids I–IV synthesis

To a round-bottom flask equipped with a magnetic stirring bar was added Boc-Morph-AA **6** (0.2 mmol) and dissolved in CH_2_Cl_2_ (0.1 M). The solution was cooled to 0°C and TFA (1 mL TFA for 25 mg reagent) was slowly added dropwise, and then, the mixture was stirred for 3 h. The crude mixture was concentrated *in vacuo*, yielding products **I–IV** in the quantitative yield as white solids.

### Synthesis γ-nitroaldehydes 9–20

Catalyst (1–5 mol%; see Table 2) was added to a solution of *N*-methylmorpholine (1–5 mol%), nitroolefin **8** (0.17 mmol), and aldehyde **7** (0.11 mmol) in *i*PrOH (0.380 mL). The reaction mixture was stirred at −10°C for 24–48 h (Table 2). The solvent was removed under reduced pressure, and the crude mixture was subjected to flash chromatography (silica gel; 5% → 20% EtOAc in hexane) to yield γ-nitroaldehyde **9–20** (Table 2). The diastereomeric ratio was determined by the ^1^H NMR spectroscopic analysis of the isolated product by comparison of the aldehyde R-CHO signals. The enantiomeric excess was determined by chiral stationary phase HPLC.

Further details and spectroscopic analyses for each compound are reported in SI.

## Data Availability

The original contributions presented in the study are included in the article/Supplementary Material, further inquiries can be directed to the corresponding author.
