# Supplementary material for: Highly efficient morpholine-based organocatalysts for the 1,4-addition reaction between aldehydes and nitroolefins: an unexploited class of catalysts
Source: Front Chem. 2023 Aug 11;11:1233097. doi: 10.3389/fchem.2023.1233097 (PMC10451084; doi:10.3389/fchem.2023.1233097)

Supplementary Material

Highly efficient morpholine-based organocatalysts for 1,4-addition reaction between aldehydes and nitroolefins: an unexploited class of catalysts

Francesco Vaghi,† Giorgio Facchetti,† Isabella Rimoldi, Matteo Bottiglieri, Alessandro Contini, Maria Luisa Gelmi and Raffaella Bucci*

DISFARM, Dipartimento di Scienze Farmaceutiche, Sezione Chimica Generale e Organica "A. Marchesini", Università degli Studi di Milano, Milan, Italy

*** Correspondence:**
Raffaella Bucci
raffaella.bucci@unimi.it

Computational studies ***3***

General information ***16***

Synthesis of Catalysts I-IV ***16***

(*R*)-2-amino-3-phenylpropan-1-ol (2a) 16
(*S*)-2-Amino-3-phenylpropan-1-ol (2b) 16
(*R*)-2-amino-2-phenylethan-1-ol (2c) 16
(*R*)-2-amino-3-(naphthalen-2-yl)propan-1-ol (2d) 17
(*R*)-2-(Benzylamino)-3-phenylpropan-1-ol (3a) 17
(*S*)-2-(Benzylamino)-3-phenylpropan-1-ol (3b) 17
(*R*)-2-(Benzylamino)-2-phenylethan-1-ol (3c) 17
(*R*)-2-(Benzylamino)-3-(naphthalen-2-yl)propan-1-ol (3d) 18
(2*S*,5*R*)-[4,5-Dibenzylmorpholin-2-yl]methanol (4a) 18
[(2*S*,5*R*)-4,5-Dibenzylmorpholin-2-yl]-methanol (4b) 18
[(2*S*,5*R*)-4-Benzyl-5-phenylmorpholin-2-yl]methanol (4c) 19
(2*S*,5*R*)-4-Benzyl-5-(naphthalen-2-yl-methyl)morpholin-2-yl]methanol (4d) 19
Butyl (2*S*,5*R*)-5-benzyl-2-(hydroxymethyl)morpholine-4-carboxylate (5a) 19
*t*Butyl (2*S*,5*S*)-5-benzyl-2-(hydroxymethyl)morpholine-4-carboxylate (5b) 20
*t*Butyl (2*S*,5*R*)-2-(hydroxymethyl)-5-phenylmorpholine-4-carboxylate (5c) 20
*t*Butyl (2*S*,5*R*)-2-(hydroxymethyl)-5-(naphthalen-2-yl-methyl)morpholine-4-carboxylate (5d) 20
(2*S*,5*R*)-5-Benzyl-4-(*t*butoxycarbonyl)morpholine)-2-carboxylic acid (6a) 20
(2*S*,5*S*)-5-Benzyl-(*t*butoxycarbonyl)morpholine-2-carboxylic acid (6b) 20
(2*S*,5*R*)-4-(*t*Butoxycarbonyl)-5-phenyl-morpholine-2-carboxylic acid (6c) 21
(2*S*,5*R*)-4-(*t*Butoxycarbonyl)-5-(naphthalen-2-yl-methyl)morpholine-2-carboxylic acid (6d) 21
(2*S*,5*R*)-5-Benzyl-morpholine-2-carboxilic acid TFA salt I 21
(2*S*,5*S*)-5-Benzyl-morpholine2-carboxilic acid TFA salt II 21
(2*S*,5*R*)-5-phenylmorpholine-2-carboxylic acid TFA salt III 21
(2*S*,5*R*)-5-(naphthalen-2-ylmethyl)Morpholine 2-carboxilic acid TFA salt IV 22

Synthesis γ-Nitroaldehydes 9-22 .... ***22***

Diastereoisomeric and Enantiomeric excess analyses for compound 9-22 .... ***22***

(2*R*,3*S*)-2-Ethyl-4-nitro-3-phenyl-butanal (9) 23
Synthesis of (2*R*,3*S*)-2-methyl-4-nitro-3-phenylbutanal (10) 25
Synthesis of (*R*)-2-((*S*)-2-nitro-1-phenylethyl)pentanal (11) 27

Synthesis of (*2R,3S*)-2-isopropyl-4-nitro-3-phenylbutanal (12) 29
Synthesis of (*R*)-2-((*S*)-2-nitro-1-phenylethyl)hexanal (13) 32
Synthesis of (*2S,3S*)-4-nitro-2,3-diphenylbutanal (14) 34
Synthesis of (*R*)-1-(2-nitro-1-phenylethyl)cyclopentane-1-carbaldehyde (15) 37
Synthesis of (*2R,3S*)-3-(4-methoxyphenyl)-2-methyl-4-nitrobutanal (16) 39
Synthesis of (*R*)-2-((*S*)-1-(4-methoxyphenyl)-2-nitroethyl)pentanal (17) 41
Synthesis of (*2R,3R*)-2-methyl-4-nitro-3-(thiophen-2-yl)butanal (18) 43
Synthesis of (*2R,3S*)-3-(4-chlorophenyl)-2-methyl-4-nitrobutanal (19) 45
Synthesis of (*R*)-2-((*S*)-1-(4-chlorophenyl)-2-nitroethyl)pentanal (20) 47
Synthesis of (2*R*,3*R*)-3-cyclohexyl-2-methyl-4-nitrobutanal (21) 49
Synthesis of (2*R*,3*R*)-2-methyl-3-(nitromethyl)heptanal (22) 51

References for the synthesis and Spectroscopical analysis of known compounds 53

^{1}H and ^{13}C NMR of new compounds 55

Compound 3d 55
Compound 4a 57
Compound 4b 59
Compound 4c 61
Compound 4d 63
Compound 5a 65
Compound 5b 67
Compound 5c 69
Compound 5d 71
Compound 6a 73
Compound 6b 75
Compound 6c 77
Compound 6d 79
Catalyst I 81
Catalyst II 83
Catalyst III 85
Catalyst IV 87

Copy of γ-Nitroaldehydes ^{1}H-NMR 89

Compound 9 89
Compound 10 90
Compound 11 91
Compound 12 92
Compound 13 93
Compound 14 94
Compound 15 95
Compound 16 96
Compound 17 97
Compound 18 98
Compound 19 99
Compound 20 100
(2*R*,3*R*)-3-cyclohexyl-2-methyl-4-nitrobutanal (21) 101
(2*R*,3*R*)-2-methyl-3-(nitromethyl)heptanal (22) 102

Computational studies

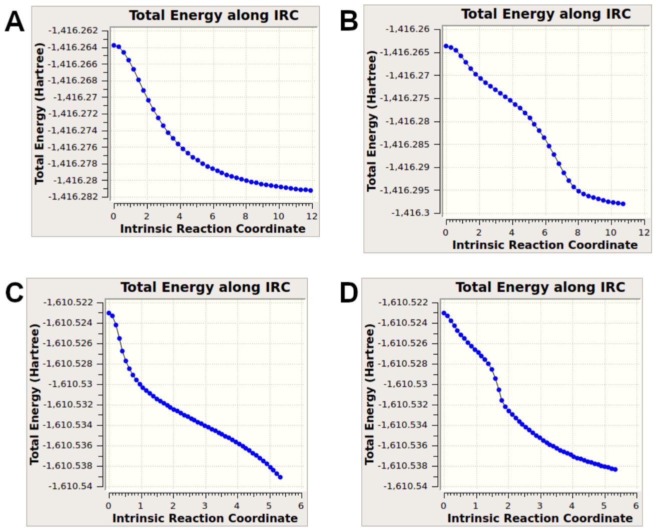

Figure S1: IRC analyses conducted at the mPW1B95/6-31G* level for the reaction leading to product (2*R*,3*S*)-**9**. Panels A and B represent reaction paths from **TS1** to the activated complex and dihydrooxazine oxide **Int1-OX**, respectively. Panels C and D represent reaction paths from **TS2** to the dihydrooxazine oxide **Int1** and imino **Int2**, respectively. Energies (a.u.) are uncorrected.

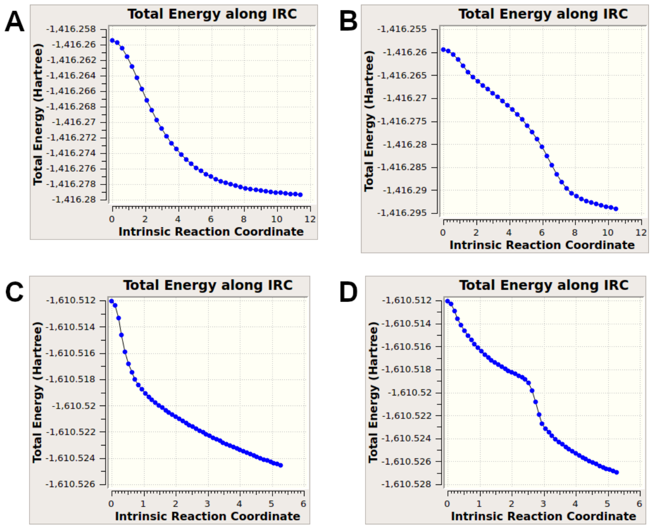

Figure S2: IRC analyses conducted at the mPW1B95/6-31G* level for the reaction leading to product (2*S*,3*R*)-**9**. Panels A and B represent reaction paths from **TS1** to the activated complex and dihydrooxazine oxide **Int1-OX**, respectively. Panels C and D represent reaction paths from **TS2** to the dihydrooxazine oxide **Int1** and imino **Int2**, respectively. Energies (a.u.) are uncorrected.

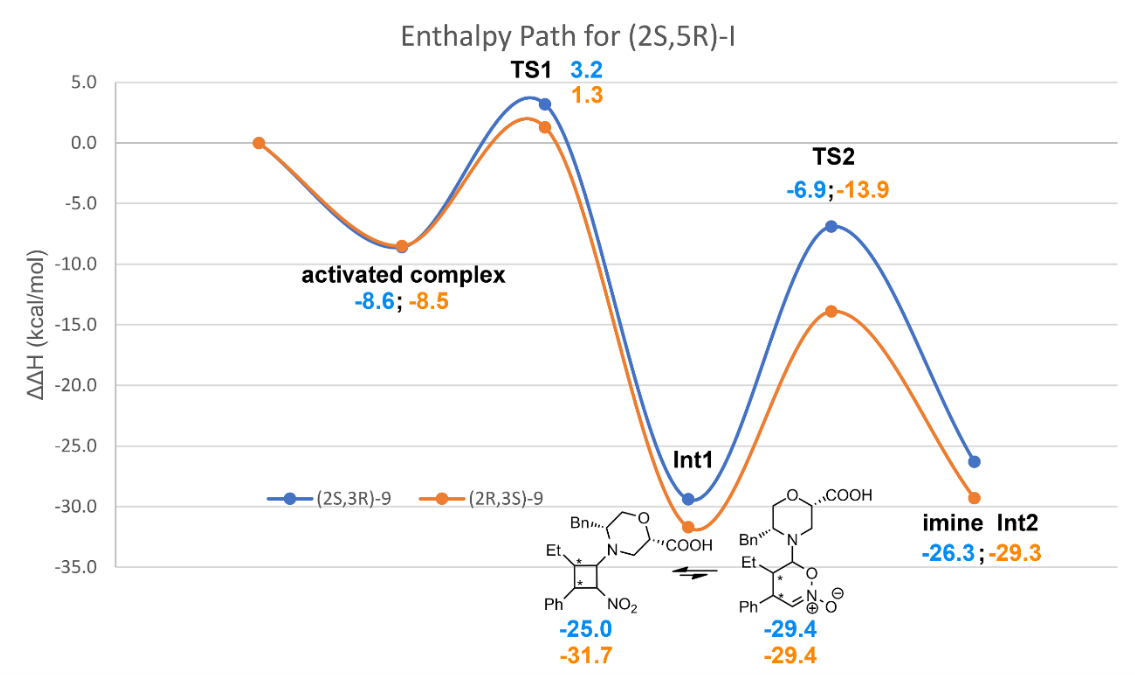

Figure S3: Enthalpy path for the reaction of **7a** and **8a** in presence of catalyst **I**. Relative solution-phase enthalpies (kcal/mol) with respect to reactants are reported.

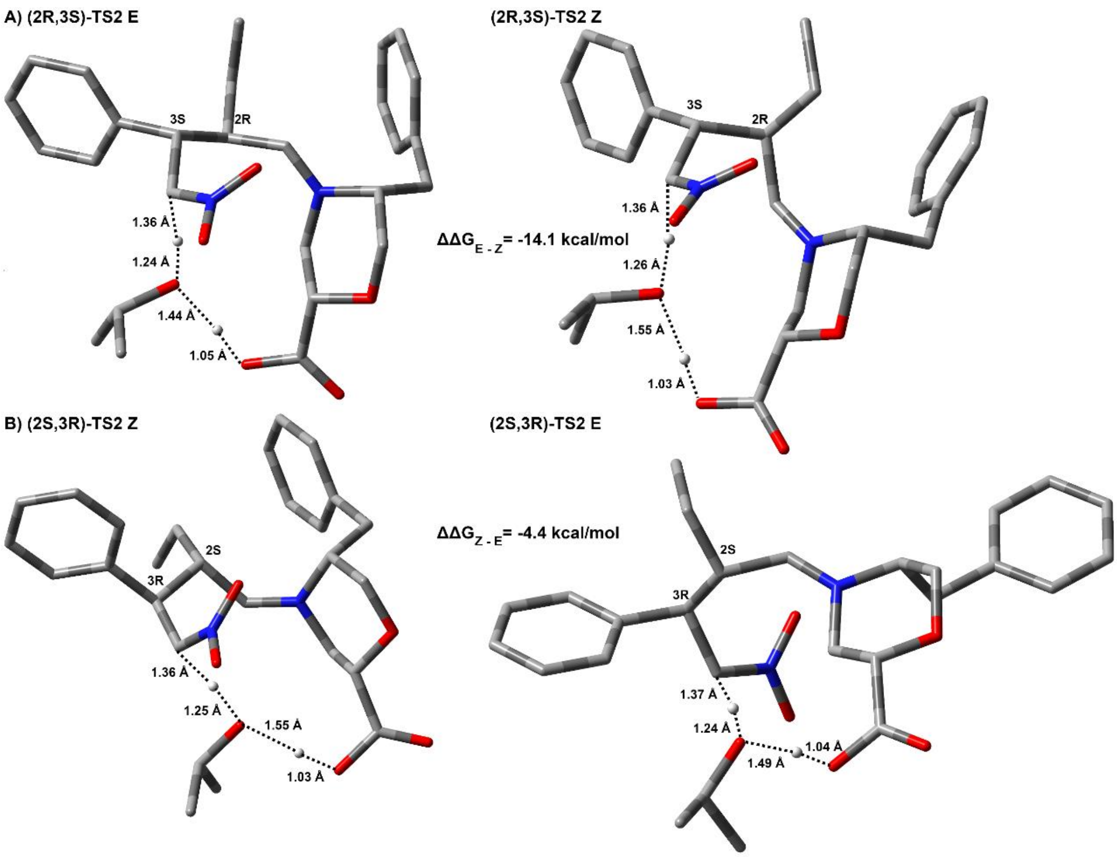

Figure S4: Comparison between the immine *E/Z* configurations in (2*R*,3*S*)-**TS2** (A) and (2*S*,3*R*)-**TS2**. Free energy differences are reported in kcal/mol, selected distances in Angstrom.

Cartesian coordinates and full Gaussian input (optimization and frequency, followed by single point calculation) for all the discussed stationary points.

The obtained number of imaginary frequencies (IF), absolute electronic energies (Eel) and thermochemical corrections at 1 atm, 298.15 K (ZPEc, Hc and Gc for zero-point energy, enthalpy and Gibbs free energy corrections, respectively) are reported in the title of the Gaussian input.

Isolated Reactants

Enamine E-7a

```
%chk=Enamine_9a.chk
# opt freq wb97xd/6-311g(d,p) scrf=(cpcm,solvent=2-propanol)
10f 6d gfinput gfprint

opt+freq; IF=0; Eel=-902.73166; ZPEc=0.351531;
Hc=0.371185; Gc=0.302736

0 1
 N     0.47920500    0.57554200   -0.81853600
 C     0.49292000   -0.78211700   -1.36149800
 C    -0.74587100    1.23489200   -0.75336800
 C     1.90438100   -1.07598200   -1.85799600
 O     2.84186800   -0.90878000   -0.79993500
 C     1.46499800    0.83251500    0.20829000
 C     2.84013300    0.41436800   -0.30496900
 C    -3.88653000   -1.07591700    1.21610900
 C    -2.76483400   -0.95061300    2.02750800
 C    -1.50196400   -1.23290100    1.51890300
 C    -1.33819700   -1.64179200    0.19747700
 C    -2.47306100   -1.78082700   -0.60303600
 C    -3.73716200   -1.49823700   -0.10160500
 C     3.88844800    0.50196200    0.79418600
 O     4.62719100   -0.59022900    0.95705300
 O     4.04262600    1.48707500    1.46496500
 C     0.03406200   -1.88112000   -0.37492500
 C    -1.11537900    2.20213700    0.09096100
 C    -2.44891500    2.90314200    0.05567800
 C    -3.43663100    2.39702700   -0.99109200
 H    -1.43362500    0.89464100   -1.52140700
 H     4.31254400   -1.25605200    0.32371900
 H     1.50205000    1.90058600    0.42253200
 H     1.23236600    0.31319200    1.15094300
 H     2.16221500   -0.39986300   -2.68166900
 H     1.99958700   -2.10791500   -2.19348600
 H    -0.17114000   -0.78826300   -2.23094200
 H     0.76616100   -1.96777100    0.43201300
 H     0.04925100   -2.83171100   -0.91796100
 H    -0.62902500   -1.12246400    2.15462300
 H    -2.87208100   -0.62830400    3.05722900
 H    -4.87199900   -0.85178800    1.60832500
 H    -4.60763400   -1.60691600   -0.73901600
 H    -2.36378800   -2.10590400   -1.63344200
 H     3.14292800    1.10459500   -1.10537000
 H    -0.44529200    2.51838500    0.88503000
 H    -4.37581500    2.95161200   -0.92931900
 H    -3.66040500    1.33650700   -0.84228300
 H    -3.04393700    2.51950800   -2.00498200
 H    -2.28835400    3.97890800   -0.09282800
 H    -2.91031900    2.81646900    1.04764000

--link1--

%chk=Enamine_9a.chk
# wb97xd/6-311++g(3df,3pd) scrf=(cpcm,solvent=2-propanol)
10f 6d gfinput gfprint geom=checkpoint guess=read

SP; Eel=-902.818083421

0 1
```

Nitrostyrene 8a

```
%chk=Nitrostirene.chk
# opt freq wb97xd/6-311g(d,p) scrf=(cpcm,solvent=2-propanol)
10f 6d gfinput gfprint

opt+freq; IF=0; Eel=-514.11514; ZPEc=0.137786;
Hc=0.147781; Gc=0.102034

0 1
 N    -2.97745400   -0.03312700    0.00009600
 C    -1.59208900   -0.45656700    0.00027700
 H    -1.50026700   -1.53154900    0.00072000
 O    -3.81202600   -0.92440600    0.00019300
 O    -3.25070000    1.15505800   -0.00012300
 C    -0.61501900    0.44858900   -0.00017900
 H    -0.91329300    1.49268200   -0.00058800
 C     3.58560800   -0.22765600    0.00004100
 H     4.65758900   -0.38689900    0.00010300
 C     3.07353300    1.06389600    0.00000900
 H     3.74234600    1.91619700    0.00004800
 C     1.69947600    1.26302900   -0.00008700
 H     1.29924300    2.27100900   -0.00012400
 C     0.82045600    0.17464300   -0.00012500
 C     1.34914500   -1.12332800   -0.00010800
 H     0.69180100   -1.98476400   -0.00018800
 C     2.72019200   -1.31973700   -0.00002500
 H     3.11875900   -2.32722400   -0.00002300

--link1--

%chk=Nitrostirene.chk
# wb97xd/6-311++g(3df,3pd) scrf=(cpcm,solvent=2-propanol)
10f 6d gfinput gfprint geom=checkpoint guess=read

SP; Eel=-514.167824623

0 1
```

iPrOH

```
%chk=iPrOH.chk
# opt freq wb97xd/6-311g(d,p) scrf=(cpcm,solvent=2-propanol)
10f 6d gfinput gfprint

opt+freq; IF=0; Eel=-194.36022; ZPEc=0.108521;
Hc=0.114850; Gc=0.081189

0 1
 O    -0.00000200    1.40972000    0.02389100
 C     0.00000000    0.03992700   -0.37402000
 H     0.00000000    0.07398700   -1.46736100
 H    -0.00000700    1.42795500    0.98396600
 C     1.26530500   -0.66446300    0.09965100
 H     1.29232000   -0.70239600    1.19421700
 H     1.30672200   -1.69043200   -0.27545400
 H     2.15188700   -0.13000700   -0.24847500
 C    -1.26530300   -0.66446700    0.09965100
 H    -2.15188600   -0.13001100   -0.24847200
 H    -1.30671900   -1.69043400   -0.27545700
 H    -1.29231700   -0.70240400    1.19421600

--link1--
```

%chk=iPrOH.chk
wb97xd/6-311++g(3df,3pd) scrf=(cpcm,solvent=2-propanol) 10f 6d gfinput gfprint geom=checkpoint guess=read

SP; Eel=-194.381626157

0 1

Stationary points for the reaction giving (2*R*,3*S*)-9

Activated complex

%chk=2R3S_activated_complex.chk
opt freq wb97xd/6-311g(d,p) scrf=(cpcm,solvent=2-propanol) 10f 6d gfinput gfprint

opt+freq; IF=0; Eel=-1611.226974; ZPEc=0.491356; Hc=0.521565; Gc=0.427537

0 1

N	-0.79178300	0.57177600	0.39378200
C	-1.99277600	1.27784700	0.84550100
C	-0.79264800	-0.81589200	0.47564500
H	-1.39413100	-1.17766400	1.30262000
C	-1.61081000	2.72108900	1.16074400
O	-0.99979500	3.33143900	0.02538700
C	-0.08640000	1.20253900	-0.70076400
C	0.18676100	2.65654800	-0.33342200
C	-4.20709000	-2.78176300	-1.27648800
C	-4.50815200	-2.31015800	-0.00208400
C	-4.19980100	-1.00168800	0.34704900
C	-3.57965800	-0.14562300	-0.56433800
C	-3.29940200	-0.62363700	-1.84213900
C	-3.60866800	-1.93141400	-2.19888400
C	0.83650100	3.39980900	-1.48741500
O	0.21682500	4.51021500	-1.86740100
H	-0.56788200	4.61811300	-1.30507300
O	1.84383700	3.01127200	-2.01786300
H	0.86932200	0.70242500	-0.85321800
H	-0.64555600	1.14745000	-1.64748200
H	-0.91412500	2.75007800	2.00507400
H	-2.49220800	3.31702800	1.39367300
H	-2.30432700	0.80783700	1.78329500
C	-3.17347700	1.24464200	-0.15223900
H	-2.91245700	1.83496100	-1.03446700
H	-4.01631400	1.75164200	0.32871500
H	-4.43192700	-0.64276200	1.34538500
H	-4.98349500	-2.96407000	0.72060400
H	-4.44369300	-3.80392700	-1.54929900
H	-3.37685300	-2.28785400	-3.19635600
H	-2.81939800	0.03128700	-2.56260900
H	0.88793400	2.68940700	0.51110200
C	-0.11429100	-1.67718200	-0.28917500
H	0.45672600	-1.31902100	-1.14028300
C	-0.08062500	-3.16548700	-0.07355600
C	-0.89064400	-3.68017200	1.11163300
H	-1.95057800	-3.43204800	1.00293600
H	-0.53859000	-3.24796300	2.05319700
H	-0.80594300	-4.76637500	1.18995900
H	0.96518800	-3.48219600	0.03908100
H	-0.43063400	-3.66012100	-0.98838300
N	1.17237700	0.70426800	2.85310700
C	2.11326700	0.46467100	1.79109000
H	2.56728100	1.37488700	1.43189800
O	1.01574200	1.87428500	3.18379400
O	0.58367800	-0.22852300	3.37170300
C	2.34102100	-0.77293700	1.35070600
H	1.84510300	-1.58983300	1.86302900
C	4.87939600	-1.87041100	-1.88296400
H	5.51990100	-2.16188800	-2.70716800
C	4.49619200	-2.80982600	-0.93281400
H	4.83821400	-3.83495300	-1.01075000
C	3.66661700	-2.43575900	0.11558900
H	3.36377900	-3.16890800	0.85531900
C	3.22129800	-1.11549200	0.23463800
C	3.60768600	-0.17793000	-0.73212500
H	3.24562600	0.84381000	-0.68858400
C	4.43027500	-0.55604200	-1.78181300
H	4.71514700	0.17445700	-2.52964500

--link1--

%chk=2R3S_activated_complex.chk
wb97xd/6-311++g(3df,3pd) scrf=(cpcm,solvent=2-propanol) 10f 6d gfinput gfprint geom=checkpoint guess=read

SP; Eel=-1417.00205322

0 1

(4S,5R)-TS1

%chk=4S5R-TS1.chk
opt=(readfc,ts,noeigentest) freq wb97xd/6-311g(d,p) scrf=(cpcm,solvent=2-propanol) guess=read 10f 6d geom=checkpoint gfinput gfprint

opt+freq; #IF=1; Eel=-1416.85059; ZPEc=0.492233; Hc=0.520935; Gc=0.431814

0 1

N	-0.92062000	0.49600100	0.19330600
C	-2.18914500	0.95550900	0.78121900
C	-0.60510800	-0.79666200	0.28728500
H	-1.23592200	-1.35427600	0.96589800
C	-2.04756600	2.41156600	1.21655800
O	-1.56457400	3.21439200	0.14338800
C	-0.31838800	1.37625300	-0.80085300
C	-0.27657700	2.79204600	-0.24601400
C	-3.51727000	-3.41229600	-1.29312900
C	-4.06782300	-2.98732800	-0.08754900
C	-4.05657700	-1.63903700	0.24650900
C	-3.49568100	-0.69457400	-0.61509100
C	-2.95997100	-1.13035500	-1.82602300
C	-2.96786100	-2.47946100	-2.16434000
C	0.26878500	3.76799100	-1.27517100
O	-0.44826400	4.87152700	-1.43932400
H	-1.22903800	4.81005900	-0.86523100
O	1.28473500	3.56046500	-1.88316400
H	0.69242100	1.05126200	-1.02425700
H	-0.90336900	1.35299200	-1.72803600
H	-1.35597400	2.47818600	2.06250000
H	-3.01816100	2.81529800	1.49996600
H	-2.34456900	0.36011700	1.68414300
C	-3.37258900	0.74390200	-0.18692900
H	-3.25243300	1.39769300	-1.05460600
H	-4.27777500	1.06487100	0.33570800
H	-4.47752600	-1.31475000	1.19337600
H	-4.50396500	-3.70831900	0.59451200
H	-3.51976400	-4.46482400	-1.55196800
H	-2.54083100	-2.80084300	-3.10746600
H	-2.52159800	-0.40895600	-2.50838400
H	0.39578500	2.82036900	0.62257000
C	0.49921400	-1.41072100	-0.27985400
H	0.94372600	-0.93899900	-1.14858700
C	0.61174900	-2.91945100	-0.27379200
C	-0.11905700	-3.62760100	0.86426600
H	-1.20436800	-3.55107300	0.75213700
H	0.14879200	-3.20782200	1.83849400
H	0.13607400	-4.68902500	0.87318500
H	1.67343100	-3.18721100	-0.25282700
H	0.23589700	-3.29505300	-1.23223100
N	0.75574300	0.64406300	2.60329700
C	1.70202200	0.52160100	1.61790800
H	2.17590300	1.44600900	1.32972700
O	0.54804000	1.76897300	3.11085200

O	0.11491100	-0.36244200	2.97004100
C	1.98968000	-0.72978400	1.07100100
H	1.77771200	-1.57925400	1.71021000
C	5.46158300	-1.16947100	-1.39616500
H	6.34151800	-1.28671300	-2.01789200
C	5.11617600	-2.15518300	-0.48069700
H	5.72719800	-3.04480000	-0.38113200
C	3.98375200	-2.00424700	0.31198400
H	3.72220900	-2.77299200	1.03113600
C	3.18522200	-0.86418200	0.20478100
C	3.54215100	0.12211800	-0.72122000
H	2.93542600	1.01519700	-0.83239100
C	4.67036200	-0.02954100	-1.51329200
H	4.93205300	0.74254500	-2.22746600

--link1--

%chk=4S5R-TS1.chk
wb97xd/6-311++g(3df,3pd) scrf=(cpcm,solvent=2-propanol)
10f 6d gfinput gfprint geom=checkpoint guess=read

SP; Eel= -1416.98587346

0 1

(4S,5R)-Int1-OX

%chk=4S5R-Int1_6-IRC.chk
opt freq wb97xd/6-311g(d,p) scrf=(cpcm,solvent=2-propanol)
10f 6d gfinput gfprint

opt+freq; #IF=0; Eel=-1611.269996; ZPEc=0.606648;
Hc=0.640917; Gc=0.539711

0 1

N	-1.58235100	-0.05847200	-0.54779100
C	-2.89402400	-0.09400800	-1.19611500
C	-0.52337500	0.51018600	-1.25754800
H	-0.92907500	1.28580100	-1.90484900
C	-3.14756900	-1.48159500	-1.77971200
O	-3.03757000	-2.47529700	-0.77379400
C	-1.36788400	-1.14335100	0.39651800
C	-1.74862400	-2.50943400	-0.20857500
C	-2.95412700	4.01364100	1.75257500
C	-3.26935800	4.00603700	0.39735300
C	-3.62747000	2.81925300	-0.22888000
C	-3.67716600	1.61998200	0.48378600
C	-3.36711300	1.64127800	1.84208600
C	-3.00745400	2.82719400	2.47449800
C	-1.76230900	-3.57628000	0.88423100
O	-0.57720300	-3.91787000	1.37157800
H	0.18609100	-3.48105400	0.91109600
O	-2.77543000	-4.05986900	1.31596900
H	-0.32566100	-1.17452300	0.70896600
H	-1.98009800	-0.96854900	1.28775200
H	-2.42641500	-1.67794500	-2.58336000
H	-4.15960700	-1.56173100	-2.17885400
H	-2.87174100	0.62191100	-2.02139500
C	-4.00444300	0.32672200	-0.21648700
H	-4.16804300	-0.46818700	0.51528300
H	-4.93223000	0.42484200	-0.78841100
H	-3.86841800	2.82311300	-1.28776300
H	-3.23209200	4.92686900	-0.17386300
H	-2.67048500	4.93858800	2.24157300
H	-2.76648800	2.82251700	3.53169500
H	-3.40482500	0.71828000	2.41219700
H	-1.00733500	-2.77418400	-0.97792900
C	0.59996100	1.08975000	-0.40320400
H	0.78751600	0.41520700	0.43617100
C	0.17312300	2.44003800	0.19163900
C	0.03954600	3.59338800	-0.79910600
H	-0.69950200	3.38848500	-1.57804100
H	0.99051100	3.82775500	-1.28417200
H	-0.29436300	4.49058900	-0.27399100
H	0.90364400	2.71020800	0.95944900
H	-0.78052100	2.28705500	0.70435800
N	1.24215600	-1.01638500	-2.08747400
C	2.19678100	-0.27247600	-1.64498100
H	3.14859700	-0.76311900	-1.50240600
O	1.29487600	-2.24980600	-2.35745000
O	0.02451300	-0.42731600	-2.33054400
C	1.90722100	1.13394500	-1.22814900
H	1.74231600	1.74590100	-2.12347000
C	5.18464300	2.77818900	1.03797700
H	6.00503800	3.19341000	1.61173000
C	4.76416500	3.40062800	-0.13067400
H	5.25634700	4.30329100	-0.47396500
C	3.71106000	2.86665100	-0.86679400
H	3.39150900	3.35473500	-1.78167400
C	3.06562800	1.70898000	-0.44232300
C	3.49705600	1.08862600	0.73153600
H	3.00549100	0.18514600	1.07877300
C	4.54862800	1.61727700	1.46730600
H	4.87120000	1.12308700	2.37645200
O	1.48814400	-2.74585100	0.27008000
C	2.77064800	-2.87832000	0.89522600
H	3.43597300	-2.11853000	0.46630900
H	1.55552400	-2.81369700	-0.70365200
C	3.35476700	-4.25724200	0.63402300
H	3.46284600	-4.43394700	-0.43916700
H	4.34118600	-4.34827200	1.09537000
H	2.70172700	-5.02924400	1.05076100
C	2.57242800	-2.58586200	2.37068200
H	3.52983600	-2.61171700	2.89486000
H	2.12537000	-1.59860800	2.51140200
H	1.91045300	-3.33060900	2.82157000

--link1--

%chk=4S5R-Int1_6-IRC.chk
wb97xd/6-311++g(3df,3pd) scrf=(cpcm,solvent=2-propanol)
10f 6d gfinput gfprint geom=checkpoint guess=read

SP; Eel= -1611.42151248

0 1

(2S,3R)-Int1-CB

%chk=4S5R-Int1_4-IRC.chk
# opt freq wb97xd/6-311g(d,p) scrf=(cpcm,solvent=2-propanol)
guess=read 10f 6d gfinput gfprint

opt+freq; #IF=0; Eel=-1611.274833; ZPEc=0.606166;
Hc=0.641357; Gc=0.537354

0 1

N	-1.51867200	0.50023800	0.29512000
C	-2.53239400	1.04448200	1.19108100
C	-0.71396800	-0.58207200	0.73980300
H	-1.20320100	-1.05662700	1.59281200
C	-2.04811000	2.34880800	1.82383900
O	-1.61402200	3.26952200	0.83381200
C	-0.96493600	1.46311600	-0.63207000
C	-0.53094000	2.74813800	0.09734700
C	-4.92675700	-2.44078900	-1.55887800
C	-5.01014100	-2.34980500	-0.17320000
C	-4.69085200	-1.15924700	0.46784300
C	-4.28003500	-0.04104300	-0.25935500
C	-4.20239300	-0.14391400	-1.64701800
C	-4.52384500	-1.33211800	-2.29474600
C	-0.09813000	3.79528800	-0.91950300
O	1.17044200	3.72935500	-1.30873600
H	1.70128100	3.08045900	-0.79029900
O	-0.85618200	4.59800500	-1.39621100
H	-0.12184000	1.02818200	-1.17160100
H	-1.71697600	1.73941500	-1.37993000
H	-1.22265900	2.13872800	2.51952700
H	-2.84982400	2.84464800	2.37264000
H	-2.68525300	0.32016900	1.99518100
C	-3.87181600	1.22347000	0.45084200
H	-3.79010200	2.04897400	-0.26116700
H	-4.62964200	1.51192200	1.18576200
H	-4.75778300	-1.09806500	1.55011500

H	-5.32331600	-3.20879800	0.40969800
H	-5.17392100	-3.36932200	-2.06080500
H	-4.45597900	-1.39242100	-3.37533800
H	-3.88341500	0.71604700	-2.22756100
H	0.29923300	2.51787200	0.77969600
C	-0.27047500	-1.68064000	-0.25467900
H	0.01833500	-1.18874400	-1.18939200
C	-1.21707600	-2.82767600	-0.57368300
C	-1.68667900	-3.64022700	0.62896200
H	-2.22614500	-3.01841100	1.34853000
H	-0.85267900	-4.12098800	1.14803700
H	-2.37334300	-4.42646400	0.30707500
H	-0.72808400	-3.49112800	-1.29544000
H	-2.08965700	-2.40292600	-1.08179200
N	1.14716800	-0.06913900	2.48022600
C	0.79551500	-0.43022600	1.08979700
H	1.29971100	0.28936800	0.45054400
O	1.69370300	1.01215200	2.66367800
O	0.84881200	-0.83369900	3.37370400
C	0.99757700	-1.87938200	0.61708500
H	0.77039200	-2.54554600	1.45375900
C	4.91285400	-2.62674000	-0.99344200
H	5.90951300	-2.78093700	-1.39041500
C	4.71008300	-2.55830700	0.38248100
H	5.54837300	-2.66142800	1.06193300
C	3.43297300	-2.35632000	0.88650400
H	3.28002600	-2.30093100	1.96006000
C	2.34005700	-2.20696300	0.02782500
C	2.55035500	-2.29535100	-1.34538700
H	1.71774400	-2.19206900	-2.03165200
C	3.82928300	-2.50442300	-1.85334500
H	3.97698400	-2.56449100	-2.92558800
O	2.67365800	1.95084500	-0.00844200
C	3.69603600	1.16780900	-0.65848100
H	3.66691900	0.15769800	-0.23645000
H	2.74852200	1.88095400	0.95012000
C	5.06533900	1.77702500	-0.41566500
H	5.28253700	1.83049600	0.65420700
H	5.83732000	1.16382600	-0.88745000
H	5.11500500	2.78604900	-0.83395700
C	3.32080000	1.10249500	-2.12529600
H	4.04575900	0.49337400	-2.66818000
H	2.33535400	0.64732200	-2.25055400
H	3.30609700	2.10434400	-2.56394100

--link1--

%chk=4S5R-Int1_4-IRC.chk
wb97xd/6-311++g(3df,3pd) scrf=(cpcm,solvent=2-propanol)
10f 6d gfinput gfprint geom=checkpoint guess=read

SP; Eel= -1611.42565856

0 1

(2R,3S)-TS2

%chk=2R3S-TS2.chk
# opt=(readfc,ts,noeigentest) freq wb97xd/6-311g(d,p)
scrf=(cpcm,solvent=2-propanol) guess=read 10f 6d
geom=checkpoint gfinput gfprint

opt+freq; IF=1; Eel=-1611.240093; ZPEc=0.600200;
Hc=0.634461; Gc=0.533933

0 1

N	-1.59359900	0.36325100	0.40089600
C	-2.80874300	0.77016500	1.14165500
C	-0.98340500	-0.72269500	0.71329200
H	-1.44047000	-1.29556700	1.51096400
C	-2.58841400	2.19251000	1.65681800
O	-2.24704000	3.07726200	0.60943100
C	-1.15981700	1.31439400	-0.62710400
C	-1.03337300	2.70102500	0.01443600
C	-3.90340700	-3.32064900	-1.53164600
C	-4.14393000	-3.17833700	-0.16844800
C	-4.22913900	-1.91178400	0.39534500
C	-4.07022300	-0.76816800	-0.39063900
C	-3.83765900	-0.92212700	-1.75590800
C	-3.75711000	-2.18941600	-2.32504700
C	-0.65025900	3.73291700	-1.05188200
O	0.52537000	3.54097200	-1.59663700
H	1.08034300	2.75520500	-1.18322700
O	-1.38867400	4.63207300	-1.36571300
H	-0.21260700	1.00107500	-1.05155100
H	-1.91819300	1.33438900	-1.41171200
H	-1.79613800	2.17471100	2.41567900
H	-3.50861400	2.56443600	2.10544000
H	-2.88420900	0.10707900	2.00379800
C	-4.04960400	0.59825600	0.25032200
H	-4.07859200	1.38418500	-0.50714800
H	-4.92123600	0.74136300	0.89411000
H	-4.41784500	-1.81052700	1.45985600
H	-4.26526100	-4.05536200	0.45675800
H	-3.83298300	-4.30841200	-1.97189500
H	-3.57529200	-2.29046600	-3.38878300
H	-3.72057500	-0.04441300	-2.38357100
H	-0.23031100	2.67142700	0.76686800
C	0.19533000	-1.31916700	0.04330200
H	0.45951700	-0.73982700	-0.84067200
C	-0.21544600	-2.73146400	-0.44362400
C	-0.48958200	-3.76934500	0.64144800
H	-1.30577400	-3.47745800	1.30732900
H	0.39526800	-3.96319700	1.25124500
H	-0.78179800	-4.71226500	0.17485500
H	0.59150300	-3.08491300	-1.09010100
H	-1.09943700	-2.62517200	-1.08082800
N	1.14582600	0.72174900	2.42655800
C	1.86471000	0.11346700	1.36408600
H	2.92322000	0.19998800	1.60253100
O	1.58019100	1.75704500	2.92535100
O	0.05755000	0.24104100	2.78425000
C	1.43326200	-1.30266000	0.99354500
H	1.17658400	-1.85879500	1.90079700
C	4.75787200	-3.27500300	-0.92499200
H	5.59417300	-3.76936600	-1.40578300
C	4.25405900	-3.76197700	0.27468400
H	4.69594000	-4.63829300	0.73514500
C	3.18021900	-3.12599200	0.89028400
H	2.79467900	-3.50891000	1.82967400
C	2.59713100	-1.99985200	0.31535800
C	3.11180100	-1.51499600	-0.88960700
H	2.67616300	-0.62974900	-1.34234000
C	4.18405600	-2.14731700	-1.50472900
H	4.57347800	-1.75720900	-2.43836200
O	1.91355500	1.67689800	-0.70300100
C	3.27678300	2.06407200	-0.75647000
H	3.87945400	1.23559300	-0.35357900
H	1.79104100	0.96779800	0.31170200
C	3.51819800	3.29280800	0.11584200
H	3.19251700	3.08959300	1.13938200
H	4.57745500	3.56547700	0.13183100
H	2.94731700	4.14549400	-0.26500000
C	3.69126700	2.28399200	-2.20542900
H	4.75057300	2.54725600	-2.27249300
H	3.52267100	1.37662200	-2.79132000
H	3.10675000	3.09494200	-2.65054600

--link1--

%chk=2R3S-TS2.chk
wb97xd/6-311++g(3df,3pd) scrf=(cpcm,solvent=2-propanol)
10f 6d gfinput gfprint geom=checkpoint guess=read

SP; Eel= -1611.39035323

0 1

(2R,3S)-TS2-Z

%chk=2R3S-TS2_Z.chk
opt=(calcfc,ts,noeigentest) freq wb97xd/6-311g(d,p) scrf=(cpcm,solvent=2-propanol) guess=read 10f 6d geom=checkpoint gfinput gfprint

opt+freq; IF=1; Eel=-1611.219994; ZPEc=0.601290; Hc=0.635443; Gc=0.535711

0 1

O	-2.46605800	-0.82316700	-0.61788200
C	-3.81842600	-0.41300000	-0.71321700
H	-3.88504400	0.63735100	-0.38836900
C	-4.27476500	-0.48084900	-2.16538300
H	-5.30743100	-0.13587500	-2.27006700
H	-3.63545200	0.14489600	-2.79393800
H	-4.21772800	-1.50978600	-2.53448600
C	-4.70213300	-1.24197700	0.21603000
H	-4.32202300	-1.17966600	1.23871500
H	-5.73607700	-0.88460900	0.20205700
H	-4.69720700	-2.29331700	-0.08863500
N	0.92909300	-1.02152900	-0.19164300
C	1.94329500	-1.57939800	0.73891100
C	0.52923500	0.19238500	-0.32228700
H	-0.21348800	0.30189300	-1.10707900
C	1.32354000	-2.76641900	1.50518400
O	0.64661600	-3.69080900	0.67114200
C	0.25049400	-2.04833800	-1.00051500
C	-0.38032100	-3.01769600	-0.00416300
C	5.03036500	1.58789700	-1.67199900
C	4.01868900	0.94145300	-2.37286900
C	3.46051500	-0.22871500	-1.87062000
C	3.89380100	-0.76076100	-0.65545700
C	4.92347400	-0.11401200	0.02920500
C	5.48957600	1.05090400	-0.47398600
C	-1.34207300	-3.95991300	-0.71543500
O	-2.39693800	-3.33257000	-1.19795600
O	-1.15893600	-5.14259700	-0.83237600
H	-0.49067000	-1.56157100	-1.62628900
H	0.97807600	-2.57594400	-1.61660400
H	0.63594400	-2.37103600	2.25921400
H	2.12423700	-3.31137500	2.00299900
H	2.17922400	-0.81435100	1.47185600
C	3.21498000	-1.95913200	-0.04514500
H	2.98208500	-2.71156100	-0.80188000
H	3.88329700	-2.43673200	0.67429500
H	2.68251700	-0.73219500	-2.43535200
H	3.66481700	1.34498800	-3.31451300
H	5.46523800	2.50075400	-2.06207800
H	6.28595900	1.54259900	0.07268700
H	5.27325500	-0.51914600	0.97333500
H	-0.98724600	-2.44171000	0.70991700
C	0.77924700	1.45585500	0.40684300
H	0.90385200	2.17259700	-0.41479500
C	1.99429300	1.62611600	1.31910700
C	2.28584400	3.10110000	1.58825100
H	2.46469400	3.64157800	0.65418000
H	1.46305900	3.59549000	2.11081100
H	3.17728700	3.20046600	2.21069800
H	2.86835800	1.19219900	0.83075700
H	1.83141500	1.10080000	2.26060200
N	-1.21701600	-0.21583300	2.37636400
C	-1.59608600	0.68279200	1.33401700
H	-2.58795500	1.05641700	1.58859400
C	-0.61310200	1.83260400	1.08862500
O	-2.05625000	-0.99894600	2.81545000
H	-0.33750300	2.26196200	2.05598900
C	-2.55881700	4.92323300	-1.21662900
H	-3.03940200	5.70286400	-1.79627500
C	-1.94917100	5.22673100	-0.00544700
H	-1.95261500	6.24528900	0.36565600
C	-1.33361000	4.22449100	0.73733900
H	-0.86224500	4.47052400	1.68319300
C	-1.31725300	2.90758500	0.28273900
C	-1.93765500	2.61182000	-0.93395600
H	-1.96215300	1.59029400	-1.29938100
C	-2.55237100	3.61087000	-1.67777400
H	-3.03162000	3.36074000	-2.61751400
O	-0.04023000	-0.24697700	2.75337400
H	-1.95903400	-0.13927400	0.31197400
H	-2.41795900	-2.33724800	-0.94554300

--link1--

%chk=2R3S-TS2_Z.chk
wb97xd/6-311++g(3df,3pd) scrf=(cpcm,solvent=2-propanol) 10f 6d gfinput gfprint geom=checkpoint guess=read

SP; Eel= -1611.36973220

0 1

(2R,3S)-Int2

%chk=2R3S-Int2.chk
opt freq wb97xd/6-311g(d,p) scrf=(cpcm,solvent=2-propanol) 10f 6d gfinput gfprint

opt+freq; IF=0; Eel=-1611.269868; ZPEc=0.606797; Hc=0.642081; Gc=0.537771

0 1

N	-1.55626000	0.28593300	0.43263900
C	-2.79626300	0.57652600	1.19135100
C	-0.90376700	-0.79542300	0.65383000
H	-1.32192100	-1.44112200	1.41920400
C	-2.68714600	2.00921000	1.71928200
O	-2.46662600	2.92957500	0.67754100
C	-1.21780600	1.29836100	-0.58283500
C	-1.22686400	2.69214200	0.04299300
C	-3.64336700	-3.47034100	-1.68122600
C	-3.86956700	-3.41536300	-0.30948000
C	-4.02615700	-2.18844400	0.32268600
C	-3.95664000	-0.99738500	-0.40378300
C	-3.73965000	-1.06463200	-1.77892400
C	-3.58545100	-2.29229600	-2.41549600
C	-0.93942200	3.75131300	-1.05538800
O	0.13969000	3.54801500	-1.68060300
H	1.30236100	2.45061800	-1.25186600
O	-1.74944200	4.66030600	-1.21943000
H	-0.24895700	1.07962300	-1.01167700
H	-1.97651900	1.23110700	-1.36478300
H	-1.87562500	2.05756100	2.45835900
H	-3.62344600	2.28063200	2.20683700
H	-2.81077000	-0.10674100	2.04133500
C	-4.02866200	0.33386800	0.30265400
H	-4.13038900	1.14962500	-0.41544900
H	-4.89828400	0.37698200	0.96341400
H	-4.20355200	-2.15597200	1.39332600
H	-3.92291900	-4.32993100	0.26973200
H	-3.51644500	-4.42711800	-2.17403800
H	-3.41697300	-2.32546300	-3.48565800
H	-3.69497900	-0.15003700	-2.36149800
H	-0.41648700	2.74861500	0.78498400
C	0.33370200	-1.25567300	-0.02294200
H	0.64125800	-0.53740800	-0.78177400
C	0.00692200	-2.58842400	-0.74401000
C	-0.27691900	-3.78740300	0.15642100
H	-1.11996400	-3.61704700	0.83014200
H	0.59379900	-4.05890800	0.75679000
H	-0.53341600	-4.64999200	-0.46152600
H	0.85935800	-2.80913000	-1.39070600
H	-0.84776500	-2.41124500	-1.40412100
N	0.86687200	0.51797600	2.59795500
C	1.86854100	-0.01871300	1.61368100
H	2.79292300	-0.09198900	2.18873100
O	0.87316500	1.71584300	2.79124100
O	0.11583200	-0.25781800	3.16053400
C	1.49730700	-1.37104100	1.00421200
H	1.20564100	-2.04632600	1.81066200
C	5.04253300	-2.95375700	-0.87378800

H	5.93493200	-3.35188800	-1.34274200
C	4.48144800	-3.60064300	0.22038900
H	4.93384300	-4.50537900	0.60976700
C	3.33608200	-3.08845100	0.82045700
H	2.90529200	-3.59509800	1.67793400
C	2.73934200	-1.92895700	0.33194100
C	3.30962300	-1.28038600	-0.76603800
H	2.86121000	-0.36976500	-1.15346200
C	4.45479300	-1.79161600	-1.36350000
H	4.88925600	-1.27932000	-2.21436200
O	1.97915700	1.77803700	-0.98313400
C	3.20375300	2.46671700	-0.75115800
H	3.89528000	1.71133600	-0.35893000
H	1.96886200	0.75778200	0.85220500
C	3.01117200	3.55670500	0.29738100
H	2.61058100	3.13242200	1.22137300
H	3.95744400	4.05565200	0.52279700
H	2.30219200	4.30365900	-0.07189400
C	3.76274900	3.01286600	-2.05886900
H	4.72725600	3.50287800	-1.89991300
H	3.89825300	2.20517200	-2.78221600
H	3.06846200	3.74462100	-2.48337100

--link1--

%chk=2R3S-Int2.chk
wb97xd/6-311++g(3df,3pd) scrf=(cpcm,solvent=2-propanol)
10f 6d gfinput gfprint geom=checkpoint guess=read

SP; Eel= -1611.42248497

0 1

Stationary points for the reaction giving (2*S*,3*R*)-9

Activated complex

%chk=activated_complex_2S3R.chk
opt freq wb97xd/6-311g(d,p) scrf=(cpcm,solvent=2-propanol)
10f 6d gfinput gfprint

opt+freq; IF=0; Eel=-1416.865592; ZPEc=0.490986;
Hc=0.521433; Gc=0.425666

0 1

N	-1.28186000	-0.55675700	0.65769900
C	-0.63455500	0.66334200	0.18972500
C	-0.59156500	-1.40851400	1.51188800
H	-1.21304600	-2.22372400	1.86898000
C	-1.40110000	1.18683900	-1.02193600
O	-2.77826300	1.35949900	-0.71403200
C	-2.71438300	-0.44497800	0.89295200
C	-3.37617500	0.12980500	-0.35032000
C	2.32853900	4.75503600	0.00873000
C	2.77077200	3.48684700	0.37335100
C	1.86091700	2.52814100	0.79947900
C	0.49492100	2.81395600	0.86862300
C	0.06466400	4.08803400	0.49966900
C	0.97260500	5.05305000	0.07431900
C	-4.86043900	0.37576100	-0.13454500
O	-5.28967200	1.57946100	-0.49873200
H	-4.52126500	2.08475800	-0.81105900
O	-5.59691300	-0.45775900	0.32049100
H	-3.12510100	-1.43665100	1.08559700
H	-2.94200400	0.19258100	1.75975900
H	-1.31138600	0.48759100	-1.86043200
H	-1.02334600	2.16208400	-1.32627000
H	0.36182200	0.38419800	-0.16314800
C	-0.48280100	1.74441000	1.28327400
H	-0.14008000	1.26177200	2.20206400
H	-1.46017500	2.18706300	1.49163300
H	2.21969200	1.54250800	1.08046600
H	3.82635600	3.24309600	0.32644100
H	3.03695900	5.50510000	-0.32380400
H	0.61840200	6.03841300	-0.20717300
H	-0.99352800	4.32613100	0.54447800
H	-3.27198200	-0.59000900	-1.17451100
C	0.70140500	-1.35630800	1.85095900
H	1.32203500	-0.53615100	1.50468400
C	1.41647400	-2.39368100	2.67288000
C	0.57945000	-3.59388500	3.10164300
H	-0.26440300	-3.29019100	3.72787500
H	0.17886900	-4.12857100	2.23540800
H	1.18598000	-4.29534100	3.67844800
H	1.84135200	-1.91171500	3.56202300
H	2.28439900	-2.74821900	2.10167900
N	-0.98071800	-2.66141200	-1.65008700
C	0.26307700	-1.94756500	-1.78670200
H	0.19917200	-1.15449500	-2.51489200
C	1.31800600	-2.27272500	-1.03962100
O	-1.91050900	-2.26687900	-2.34383500
H	1.22239800	-3.13144100	-0.38408300
C	5.03486700	-0.21031700	-0.81756600
H	5.97932200	0.31491200	-0.73662600
C	4.89540700	-1.48155800	-0.27247300
H	5.73058100	-1.95347500	0.23110500
C	3.68038200	-2.14610600	-0.36609000
H	3.57021600	-3.13579900	0.06357200
C	2.59093800	-1.55319800	-1.01264300
C	2.74538900	-0.27398000	-1.56169200
H	1.91356000	0.21928600	-2.05111000
C	3.95726900	0.39068500	-1.46273400
H	4.05850000	1.38577900	-1.87902400
O	-1.06584800	-3.59817900	-0.87544000

--link1--

%chk=
wb97xd/6-311++g(3df,3pd) scrf=(cpcm,solvent=2-propanol)
10f 6d gfinput gfprint geom=checkpoint guess=read

SP; Eel=-1417.00204854

0 1

(4R,5S)-TS1

%chk=4R5S-TS1.chk
# opt=(calcfc,ts,noeigentest) freq wb97xd/6-311g(d,p)
scrf=(cpcm,solvent=2-propanol) guess=read 10f 6d
geom=checkpoint gfinput gfprint

opt+freq; IF=1; Eel=-1416.846724; ZPEc=0.492521;
Hc=0.521179; Gc=0.432789

0 1

N	-1.13500300	-0.64541100	0.75091600
C	-0.78625900	0.67911000	0.22065500
C	-0.25952400	-1.40015800	1.41252800
H	-0.68908100	-2.28050900	1.87261200
C	-1.64226100	0.97389800	-1.00838500
O	-3.01993900	0.79478700	-0.71960500
C	-2.56142600	-0.94793000	0.88563600
C	-3.28555700	-0.55580300	-0.39295600
C	1.05174300	5.35879900	-0.04925800
C	1.80494000	4.25332300	0.33519400
C	1.17062800	3.10491200	0.79020400
C	-0.22251300	3.03861600	0.86685100
C	-0.96645100	4.15164800	0.47987400
C	-0.33482600	5.30567200	0.02614600
C	-4.78852800	-0.72642500	-0.25497300
O	-5.51140000	0.28805300	-0.71540700
H	-4.89979800	0.97994200	-1.01483200
O	-5.28841800	-1.71209700	0.21601200
H	-2.67625500	-2.01752300	1.05348500
H	-2.97971000	-0.40463900	1.73929200

H	-1.35832700	0.31086300	-1.83205300
H	-1.51113700	2.01015000	-1.31577700
H	0.24409900	0.62894800	-0.12408500
C	-0.89182600	1.76228600	1.31068000
H	-0.40936400	1.38366000	2.21641300
H	-1.94293600	1.94206700	1.54883900
H	1.76824000	2.24992300	1.09375900
H	2.88731500	4.28638400	0.28328700
H	1.54441200	6.25663500	-0.40423300
H	-0.92856900	6.16317100	-0.26954100
H	-2.04991200	4.11426400	0.53240300
H	-2.94866400	-1.21150300	-1.20931900
C	1.11677300	-1.21385200	1.42959400
H	1.48128700	-0.20922700	1.24764600
C	1.98234000	-2.01773300	2.37647100
C	1.46517200	-3.41558600	2.70957700
H	0.56936800	-3.37804200	3.33508900
H	1.21568800	-3.97932300	1.80618700
H	2.22316700	-3.97627900	3.25960600
H	2.11279100	-1.44661700	3.30202600
H	2.98371200	-2.09284200	1.94056500
N	-0.50471000	-2.57331000	-1.37071200
C	0.59208000	-1.75249500	-1.41606300
H	0.56669300	-1.00484800	-2.19217500
C	1.62403800	-1.91143000	-0.48765800
O	-1.40484200	-2.43949000	-2.23129600
H	1.73344900	-2.91099600	-0.08251800
C	5.31964300	0.18027400	-1.05992100
H	6.25774000	0.70581600	-1.19490100
C	5.31360500	-1.13777200	-0.62179400
H	6.24755200	-1.64825300	-0.41747100
C	4.10850300	-1.80762900	-0.44050700
H	4.11004000	-2.83831000	-0.10261500
C	2.89311800	-1.17248600	-0.70186000
C	2.91268600	0.15303100	-1.14866500
H	1.98483100	0.67450300	-1.35817800
C	4.11353200	0.82397000	-1.32415800
H	4.10788400	1.85295700	-1.66462300
O	-0.59344000	-3.42959900	-0.46640300

--link1--

%chk=
wb97xd/6-311++g(3df,3pd) scrf=(cpcm,solvent=2-propanol)
10f 6d gfinput gfprint geom=checkpoint guess=read

SP; Eel=-1416.98297682

0 1

(4R,5S)-Int1-OX

%chk=4R5S-Int1_6-IRC.chk
opt freq wb97xd/6-311g(d,p) scrf=(cpcm,solvent=2-propanol)
10f 6d gfinput gfprint

opt+freq; IF=0; Eel=-1611.272497; ZPEc=0.608251;
Hc=0.642178; Gc=0.542299

0 1

N	0.29225900	1.14244300	-0.22463800
C	0.19799000	1.47787400	1.20577400
C	-0.19572400	-0.08913100	-0.69393000
H	-0.05451500	-0.12355000	-1.77913400
C	1.44466900	0.99371900	1.95575600
O	2.63323200	1.49826300	1.35885900
C	1.56524700	1.55412900	-0.80906100
C	2.74425000	1.03840400	0.02445800
C	-3.65413500	4.39915500	-0.36527600
C	-2.47505400	4.39264300	-1.10257400
C	-1.28990000	3.96032700	-0.52047100
C	-1.26228800	3.52707800	0.80458400
C	-2.44809900	3.55210600	1.53973100
C	-3.63693500	3.98165400	0.96133100
C	4.05717900	1.54020800	-0.55515500
O	4.76140100	0.64246100	-1.23329000
O	4.41860700	2.68516800	-0.45984100
H	1.62875100	1.17946000	-1.83255500
H	1.61800300	2.64531400	-0.84881100
H	1.47120000	-0.10167100	1.96657700
H	1.43976500	1.35875900	2.98330500
H	-0.66889400	0.96292200	1.62373900
C	0.00114800	2.99187600	1.41918200
H	0.87222300	3.52955100	1.03736500
H	-0.01790700	3.16183900	2.49920100
H	-0.37590200	3.94984800	-1.10461200
H	-2.47965300	4.71868400	-2.13665400
H	-4.57989500	4.73271600	-0.82021500
H	-4.54970600	3.99084000	1.54653200
H	-2.44039300	3.22197900	2.57412300
H	2.72642000	-0.05230200	0.01542000
C	-1.62262900	-0.47524300	-0.33910600
H	-1.67078200	-0.61254700	0.74464600
C	-2.66137300	0.57546700	-0.74947100
C	-2.56652600	1.04967200	-2.19823000
H	-1.63089600	1.58628600	-2.37301200
H	-2.63197400	0.22683600	-2.91618900
H	-3.38202100	1.74205200	-2.41549900
H	-2.56225500	1.43968300	-0.09202000
H	-3.65387100	0.15376900	-0.55973400
N	0.47358400	-2.40438300	-0.76046600
C	-0.71363700	-2.71942400	-1.15379800
H	-0.78367600	-3.68437700	-1.63561400
C	-1.92187700	-1.84851800	-0.98992700
O	1.51511000	-3.10563200	-0.84412600
H	-2.28908400	-1.67198300	-2.00691500
C	-5.04619900	-3.90005600	1.16492900
H	-5.83303900	-4.40772100	1.71077500
C	-5.30987900	-3.33670500	-0.07730400
H	-6.30364400	-3.40265200	-0.50517000
C	-4.29776700	-2.68779200	-0.77794100
H	-4.50699900	-2.25100900	-1.74912000
C	-3.01619200	-2.59199100	-0.24240500
C	-2.75892500	-3.16134900	1.00538900
H	-1.76297300	-3.09575700	1.43297900
C	-3.76635800	-3.81162100	1.70510200
H	-3.55304800	-4.25115900	2.67272200
O	0.68319700	-1.18063200	-0.16058100
H	3.07003500	-2.20914800	-0.91660100
O	3.98609700	-1.87897300	-0.82987800
C	4.55389800	-2.48434200	0.34200600
H	4.44864500	-3.57070400	0.23727100
H	4.40332700	-0.27609700	-1.12573700
C	6.02914800	-2.13055800	0.37010700
H	6.51681300	-2.61036700	1.22135800
H	6.52144000	-2.46327300	-0.54619200
H	6.16099200	-1.04883600	0.46264100
C	3.81834400	-2.03710600	1.59918500
H	2.74981900	-2.24379000	1.50213100
H	4.19795600	-2.57361900	2.47242700
H	3.95342700	-0.96448100	1.76985100

--link1--

%chk=
wb97xd/6-311++g(3df,3pd) scrf=(cpcm,solvent=2-propanol)
10f 6d gfinput gfprint geom=checkpoint guess=read

SP; Eel=-1611.42280049

0 1

(2R,3S)-Int1-CB

%chk=4R5S-Int1_4-IRC.chk
opt freq wb97xd/6-311g(d,p) scrf=(cpcm,solvent=2-propanol)
10f 6d gfinput gfprint

opt+freq; IF=0; Eel=-1611.264742; ZPEc=0.606294;
Hc=0.640289; Gc=0.539366

0 1

N	0.11558300	-1.14690700	-0.18089900
C	-0.42941500	-1.60164200	-1.45241800

C	-0.37239600	-0.05414900	0.57500700
H	-0.13833400	-0.23366800	1.62866500
C	0.61973800	-1.44878400	-2.55940900
O	1.84082900	-2.08115400	-2.20153400
C	1.43239200	-1.65513500	0.14090000
C	2.38772200	-1.48330300	-1.05195200
C	-3.98548900	-3.74054100	1.55469500
C	-2.64652500	-3.75576100	1.93064700
C	-1.65249700	-3.54377500	0.98299600
C	-1.97614900	-3.31460000	-0.35523700
C	-3.32300400	-3.30102500	-0.71997900
C	-4.32161200	-3.51276200	0.22453200
C	3.73316100	-2.12470400	-0.74748000
O	4.48469600	-1.44660400	0.11565800
O	4.09333900	-3.17703200	-1.20221600
H	1.82252100	-1.12271300	1.00883600
H	1.39272700	-2.72496600	0.38703700
H	0.79704500	-0.38492500	-2.76317900
H	0.28614600	-1.93005000	-3.47930700
H	-1.28217800	-0.97488700	-1.71511700
C	-0.90338800	-3.07087100	-1.37989500
H	-0.03891500	-3.71020700	-1.18098800
H	-1.27490200	-3.34730800	-2.37068800
H	-0.61063100	-3.56076600	1.28428600
H	-2.37528300	-3.93100300	2.96571600
H	-4.76152200	-3.90597900	2.29330800
H	-5.36249000	-3.50078000	-0.07903600
H	-3.59164800	-3.12567300	-1.75723700
H	2.53966800	-0.41174900	-1.23927600
C	-1.82690900	0.44930100	0.46684300
H	-2.14787100	0.42073600	-0.57606700
C	-2.91398400	-0.15993700	1.34329500
C	-2.55607200	-0.32312300	2.81730100
H	-1.73628700	-1.03333900	2.95192000
H	-2.26880000	0.62467700	3.28235200
H	-3.41329100	-0.71395400	3.37021700
H	-3.18598300	-1.13387800	0.93232300
H	-3.80479600	0.47217500	1.25375600
N	0.50174700	1.71056500	-1.02266000
C	0.12338600	1.45972600	0.37244400
H	0.95535400	1.78901000	0.98504000
C	-1.30854700	1.87689100	0.76515200
O	1.69836100	1.85664200	-1.26600400
H	-1.26893500	2.03016700	1.84828400
C	-3.08530800	5.39267000	-0.96960000
H	-3.52937100	6.28289500	-1.39991900
C	-3.74045900	4.17083400	-1.05863900
H	-4.69986000	4.10360000	-1.55905300
C	-3.16787900	3.02687400	-0.51130500
H	-3.68804200	2.07954600	-0.59629400
C	-1.93735600	3.09129000	0.13625200
C	-1.28916100	4.32445200	0.22802600
H	-0.33105900	4.39014300	0.73491400
C	-1.85459500	5.46611500	-0.32394700
H	-1.33550400	6.41489300	-0.24849700
O	-0.34612500	1.68184500	-1.88957900
H	2.99497800	1.40929500	0.09808000
O	3.40499100	0.90673200	0.81700000
C	4.19867500	1.78509700	1.63502300
H	3.53869900	2.57273400	2.01621800
H	4.08758500	-0.57402300	0.35526600
C	4.71126400	0.95592300	2.79656900
H	5.28274200	1.58302500	3.48371500
H	3.88013100	0.50757200	3.34470100
H	5.36530700	0.15625700	2.43674400
C	5.31477100	2.40971500	0.81324600
H	4.90837900	2.98353000	-0.02377900
H	5.90982100	3.08647600	1.43125800
H	5.97237100	1.63145000	0.41570700

--link1--

%chk=

wb97xd/6-311++g(3df,3pd) scrf=(cpcm,solvent=2-propanol)
10f 6d gfinput gfprint geom=checkpoint guess=read

SP; Eel=-1611.41478259

0 1

(2S,3R)-TS2

%chk=2S3R-TS2.chk
# opt=(calcfc,ts,noeigentest) freq wb97xd/6-311g(d,p)
scrf=(cpcm,solvent=2-propanol) guess=read 10f 6d
geom=checkpoint gfinput gfprint

opt+freq; IF=1; Eel=-1611.228751; ZPEc=0.600995;
Hc=0.635425; Gc=0.533551

0 1

N	-1.02050900	1.00793900	-0.16142600
C	-0.88847300	1.73156700	-1.44814200
C	-0.07724400	0.46957800	0.52085000
H	-0.40351800	0.02152300	1.45491200
C	-1.84712500	1.11280700	-2.48627600
O	-3.15313500	0.88147300	-1.98373000
C	-2.40921900	0.80381500	0.27259700
C	-3.06068800	0.02974100	-0.87375500
C	1.74990900	4.86415000	1.55152800
C	0.53830300	4.36872600	2.01918000
C	-0.39711200	3.85853500	1.12588400
C	-0.13355800	3.83012400	-0.24407600
C	1.08672800	4.32838700	-0.70233100
C	2.02107300	4.84547000	0.18722100
C	-4.40178500	-0.55423300	-0.44990700
O	-4.27052700	-1.54407800	0.41173300
O	-5.46371100	-0.15643200	-0.85058700
H	-2.40300900	0.24008000	1.19943800
H	-2.90750500	1.76369700	0.40367100
H	-1.41294700	0.17193900	-2.83846000
H	-1.94440200	1.80167800	-3.32409800
H	0.12573400	1.58119900	-1.81178200
C	-1.12321200	3.23398800	-1.21231900
H	-2.14945800	3.40605900	-0.87964700
H	-1.02488400	3.71373200	-2.18858600
H	-1.34659000	3.48867100	1.49998800
H	0.31785600	4.38149700	3.08026200
H	2.47913300	5.26401000	2.24638200
H	2.96253100	5.23242600	-0.18524000
H	1.30522500	4.31340600	-1.76540700
H	-2.41624000	-0.82690900	-1.11600100
C	1.34565000	0.28832700	0.16204000
H	1.51718100	0.56645100	-0.87401600
C	2.26180900	1.15717200	1.05138700
C	2.06843200	1.02224000	2.55942900
H	1.07082900	1.34161300	2.87369200
H	2.22666600	-0.00054900	2.91063600
H	2.78741500	1.66027600	3.07692900
H	2.11502900	2.19908200	0.76073500
H	3.29026200	0.89931800	0.78444400
N	0.21646300	-2.10878900	-1.61536100
C	0.52143800	-2.16737400	-0.22323100
H	0.73662600	-3.20934100	0.01015400
C	1.61113600	-1.24034800	0.32620700
O	-0.34318100	-3.07159500	-2.13042500
H	1.56588300	-1.44144800	1.40198600
C	5.61493200	-2.32007100	-0.89006400
H	6.62004500	-2.59482600	-1.18887500
C	5.17438400	-2.57581500	0.40250900
H	5.83418000	-3.05141100	1.11935800
C	3.88516000	-2.21763200	0.78216800
H	3.54983000	-2.41788000	1.79505200
C	3.01622600	-1.60405500	-0.11981200
C	3.46964100	-1.35028700	-1.41706800
H	2.80917900	-0.87910500	-2.13498300

C	4.75753200	-1.70614400	-1.79750900
H	5.09314100	-1.50209500	-2.80813400
O	0.41354000	-1.06051300	-2.24692600
H	-0.70985000	-2.03745100	0.34514400
O	-1.80053500	-2.01503300	0.95864600
C	-1.66415000	-2.89000500	2.05901100
H	-0.59129900	-2.98309900	2.29596900
H	-3.28924800	-1.77156800	0.60310800
C	-2.36604100	-2.30158500	3.27821500
H	-2.24562500	-2.94548300	4.15405300
H	-1.95548900	-1.31625500	3.51516100
H	-3.43671900	-2.18845100	3.08041600
C	-2.19076100	-4.27865500	1.70617300
H	-1.67329700	-4.66254500	0.82302500
H	-2.04488300	-4.98446100	2.52929900
H	-3.26096800	-4.22895400	1.47977600

--link1--

%chk=
wb97xd/6-311++g(3df,3pd) scrf=(cpcm,solvent=2-propanol)
10f 6d gfinput gfprint geom=checkpoint guess=read

SP; Eel=-1611.38020430

0 1

(2S,3R)-TS2-E

%chk=2S3R-TS2_E.chk
# opt=(calcfc,ts,noeigentest) freq wb97xd/6-311g(d,p)
scrf=(cpcm,solvent=2-propanol) guess=read 10f 6d
geom=checkpoint gfinput gfprint

opt+freq; IF=1; Eel=-1611.221074; ZPEc=0.599845;
Hc=0.634382; Gc=0.531732

0 1

N	0.99949700	-0.40870500	-0.84057800
C	2.47449200	-0.54036100	-0.86407900
C	0.21658500	-1.42682600	-0.84025300
H	0.71735300	-2.39009300	-0.85474400
C	2.98847700	0.47926600	-1.88355900
O	2.61449000	1.79444200	-1.52164500
C	0.52212200	0.95607600	-0.63673500
C	1.21476700	1.95022700	-1.55166500
C	7.20976500	-1.46361400	0.62618900
C	6.21693200	-2.43406900	0.71881600
C	4.87788300	-2.06419200	0.70178700
C	4.51160100	-0.72205500	0.59213800
C	5.51386000	0.24301900	0.50093000
C	6.85532300	-0.12389400	0.51802800
C	0.88329400	3.40393400	-1.16808800
O	-0.25046000	3.64155600	-0.54956500
H	-0.86906500	2.88199900	-0.20572300
O	1.64214900	4.28977300	-1.47233200
H	-0.54376600	0.98717400	-0.80907700
H	0.70522000	1.20242300	0.41175300
H	2.60586600	0.23189000	-2.88167300
H	4.07688400	0.45192900	-1.90709900
H	2.70175900	-1.54598600	-1.22063500
C	3.05554800	-0.33284300	0.54548500
H	2.47266400	-0.92958600	1.25005600
H	2.94543100	0.71494500	0.83188300
H	4.10644600	-2.82418200	0.78052800
H	6.48625700	-3.48032000	0.80868900
H	8.25480900	-1.75099800	0.64147200
H	7.62305100	0.63829000	0.44875500
H	5.24044700	1.29013500	0.41699100
H	0.84832400	1.80115000	-2.57905800
C	-1.27133300	-1.40464200	-0.97853300
H	-1.55637300	-0.44662800	-1.41701600
C	-1.64833000	-2.49491100	-2.01059600
C	-1.50743100	-3.94042000	-1.54042600
H	-1.72195900	-4.61794100	-2.36907600
H	-0.50009200	-4.17380300	-1.18468300
H	-2.20687700	-4.17345400	-0.73592200
H	-1.04439400	-2.33753100	-2.90972700
H	-2.68359100	-2.30797000	-2.30556300
N	-0.58233300	-0.63671600	2.14055500
C	-1.76623200	-0.43447700	1.37778100
H	-1.73745400	0.82782600	0.84977900
C	-2.05818500	-1.53839300	0.36568400
O	-0.38435400	0.05404200	3.13524300
H	-1.80597300	-2.50726600	0.80664600
C	-6.29768100	-1.47325900	-0.45179100
H	-7.36220300	-1.45623400	-0.65548300
C	-5.71445500	-2.59418100	0.12561400
H	-6.32219900	-3.45616000	0.37640600
C	-4.34750700	-2.61426000	0.38556600
H	-3.90108200	-3.49083700	0.84381300
C	-3.54741200	-1.51929200	0.06939300
C	-4.14365400	-0.39286800	-0.50303400
H	-3.53761300	0.47907700	-0.72935200
C	-5.50764700	-0.37023500	-0.76203500
H	-5.95525100	0.51277500	-1.20427300
O	0.25170500	-1.47310100	1.75448600
H	-2.57732400	-0.29289000	2.09145800
O	-1.89449500	1.96209700	0.36517900
C	-2.92813300	2.60383500	1.08355100
H	-3.64449600	1.83265600	1.40983300
C	-3.66562200	3.57375000	0.16912400
H	-4.07169400	3.04552100	-0.69762800
H	-4.49170100	4.05911300	0.69648800
H	-2.98259900	4.34982300	-0.18970700
C	-2.36525200	3.28710700	2.32660100
H	-1.84254300	2.55444000	2.94719100
H	-1.65130300	4.06447600	2.03613200
H	-3.15801700	3.74917300	2.92243500

--link1--

%chk=2S3R-TS2_E.chk
wb97xd/6-311++g(3df,3pd) scrf=(cpcm,solvent=2-propanol)
10f 6d gfinput gfprint geom=checkpoint guess=read

SP; Eel= -1611.37142237

0 1

(2S,3R)-Int2

%chk=2S3R-Int2.chk
opt freq wb97xd/6-311g(d,p) scrf=(cpcm,solvent=2-propanol)
10f 6d gfinput gfprint

opt+freq; IF=0; Eel=-1611.26238; ZPEc=0.607105;
Hc=0.642454; Gc=0.537269

0 1

N	-0.98398500	1.02953300	-0.08873200
C	-0.81771200	1.68100600	-1.40967000
C	-0.07893600	0.42212100	0.58346400
H	-0.42831800	-0.00366300	1.52195800
C	-1.85450700	1.07574100	-2.37444700
O	-3.16446900	1.09364600	-1.85348500
C	-2.37670700	1.01454200	0.39323800
C	-3.21472100	0.31768600	-0.67392900
C	2.02400900	4.85295700	1.37756400
C	0.79344100	4.44588700	1.88016000
C	-0.17296100	3.93029800	1.02384900
C	0.07786500	3.80740100	-0.34345800
C	1.31639400	4.21926000	-0.83701300
C	2.28229000	4.74147100	0.01523900
C	-4.64405900	0.06372700	-0.13948200
O	-4.64588800	-0.72278100	0.84994900
O	-5.59783700	0.61551800	-0.68549800
H	-2.40971800	0.49804300	1.34624600
H	-2.72008800	2.04131700	0.52036600
H	-1.55288600	0.04934600	-2.61195800
H	-1.85669900	1.66577900	-3.29083400
H	0.17411000	1.43807700	-1.78404700
C	-0.94806200	3.20721000	-1.27007400
H	-1.96079800	3.46731000	-0.95577500

H	-0.82087500	3.61105400	-2.27688900
H	-1.13557300	3.63077200	1.42574300
H	0.58247200	4.53201900	2.93971800
H	2.77742700	5.25686900	2.04364600
H	3.23793200	5.06007600	-0.38478700
H	1.52451000	4.13172200	-1.89857200
H	-2.76883600	-0.66800100	-0.86835400
C	1.34306700	0.18893200	0.22319400
H	1.50968800	0.41719100	-0.82687700
C	2.26139200	1.12143500	1.05021400
C	2.13345400	1.02339500	2.56723800
H	1.13059600	1.28756900	2.91348400
H	2.37385100	0.02536100	2.94211700
H	2.82935500	1.72348100	3.03311000
H	2.06247000	2.14543700	0.73304800
H	3.28696000	0.89468400	0.74847300
N	0.67210400	-2.30586300	-1.63586200
C	0.64076600	-2.29335900	-0.14181800
H	0.86713800	-3.31080400	0.16631600
C	1.64973900	-1.31188100	0.47818800
O	0.93973900	-3.34436700	-2.19538300
H	1.52120600	-1.48905400	1.55060100
C	5.72279600	-2.36525900	-0.47560900
H	6.74654000	-2.63286200	-0.70982700
C	5.20005500	-2.63595700	0.78307400
H	5.81387000	-3.11547400	1.53670000
C	3.88763500	-2.28845500	1.08123900
H	3.48606100	-2.50095300	2.06668400
C	3.08131000	-1.66825000	0.12850400
C	3.61528800	-1.39904600	-1.13338400
H	3.01444500	-0.91085500	-1.89323000
C	4.92654200	-1.74621300	-1.43277400
H	5.32669500	-1.53008800	-2.41647300
O	0.42360000	-1.25864600	-2.21231600
H	-0.38996500	-2.04554900	0.13406000
O	-2.24988100	-1.88629000	1.40490700
C	-2.42655200	-3.29868900	1.45253400
H	-1.43252800	-3.71380900	1.65113700
H	-3.13933100	-1.49128400	1.23354200
C	-3.35648500	-3.68317500	2.59675200
H	-3.44794200	-4.76942200	2.67995900
H	-2.97554100	-3.29218500	3.54296900
H	-4.35294500	-3.26427600	2.42565200
C	-2.92622600	-3.81588200	0.10781300
H	-2.22937000	-3.55348000	-0.69270100
H	-3.04112600	-4.90286300	0.12030100
H	-3.89642900	-3.36523900	-0.12240200

--link1--

%chk=
wb97xd/6-311++g(3df,3pd) scrf=(cpcm,solvent=2-propanol)
10f 6d gfinput gfprint geom=checkpoint guess=read

SP; Eel=-1611.41803439

0 1

General information

Chemicals were purchased from Sigma Aldrich and were used without further purification. Mass spectra were recorded on an LCQESI MS and on a LCQ Advantage spectrometer from Thermo Finningan and a LCQ Fleet spectrometer from Thermo Scientific. The NMR spectroscopic experiments were carried out either on Varian MERCURY 300 MHz (300 and 75 MHz for ^{1}H and ^{13}C, respectively) or Bruker Avance I 400 MHz spectrometers (400 and 101 MHz for ^{1}H and ^{13}C, respectively). Optical rotations were measured on a Perkin-Elmer 343 polarimeter at 20 °C (concentration in g/100 mL). Chemical shifts (δ) are given in ppm relative to the $CHCl_3$ internal standard, and the coupling constants J are reported in Hertz (Hz).

Enantiomeric excess was monitored by HPLC with a Merck-Hitachi L-7100 HPLC System equipped with a UV6000LP detector and Chiral Column (Chiralcel AD, OD-H and IC)

Synthesis of Catalysts I-IV

(*R*)-2-amino-3-phenylpropan-1-ol (2a)

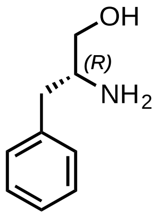

Yield: 72%. ^{1}H NMR (400 MHz, $CDCl_3$) δ 7.92 – 6.40 (m, 5H), 3.66 (dd, *J* = 10.7, 3.9 Hz, 1H), 3.41 (dd, *J* = 10.7, 7.2 Hz, 1H), 3.21 – 3.07 (m, 1H), 2.82 (dd, *J* = 13.5, 5.2 Hz, 1H), 2.55 (dd, *J* = 13.5, 8.6 Hz, 1H), 2.03 (s, 3H). ^{13}C NMR (101 MHz, $CDCl_3$) δ 138.7, 129.2, 128.6, 126.4, 66.3, 54.2, 40.8. [1]

(*S*)-2-Amino-3-phenylpropan-1-ol (2b)

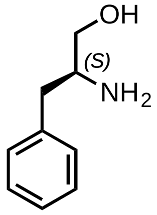

Yield: 68%. ^{1}H NMR (400 MHz, $CDCl_3$) δ 7.92 – 6.40 (m, 5H), 3.66 (dd, *J* = 10.7, 3.9 Hz, 1H), 3.42 (dd, *J* = 10.7, 7.2 Hz, 1H), 3.21 – 3.04 (m, 1H), 2.82 (dd, *J* = 13.5, 5.2 Hz, 1H), 2.55 (dd, *J* = 13.5, 8.6 Hz, 1H), 2.07 (s, 3H). ^{13}C NMR (101 MHz, $CDCl_3$) δ 138.7, 129.2, 128.6, 126.4, 66.3, 54.2, 40.8. [2]

(*R*)-2-amino-2-phenylethan-1-ol (2c)

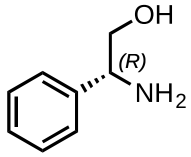

Yield: 60%. ^{1}H NMR (400 MHz, $CDCl_3$) δ 7.59 – 7.12 (m, 5H), 4.07 (dd, *J* = 8.3, 4.4 Hz, 1H), 3.76 (dd, *J* = 10.8, 4.4 Hz, 1H), 3.58 (dd, *J* = 10.8, 8.3 Hz, 1H), 2.09 (s, 3H). ^{13}C NMR (101 MHz, $CDCl_3$) δ 142.7, 128.6, 127.5, 126.4, 68.0, 57.3. [3]

(*R*)-2-amino-3-(naphthalen-2-yl)propan-1-ol (2d)

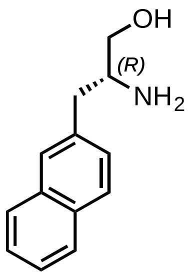

Yield:55%. ^{1}H NMR (300 MHz, $CDCl_3$) δ 7.89 – 7.71 (m, 3H), 7.64 (m, 1H), 7.54 – 7.39 (m, 2H), 7.33 (dd, *J* = 8.3, 1.8 Hz, 1H), 3.67 (dd, *J* = 10.6, 3.9 Hz, 1H), 3.44 (dd, *J* = 10.6, 7.1 Hz, 1H), 3.33 – 3.13 (m, 1H), 2.96 (dd, *J* = 13.2, 5.1 Hz, 1H), 2.68 (dd, *J* = 13.2, 8.7 Hz, 1H), 2.04 (s, 3H). ^{13}C NMR (75 MHz, $CDCl_3$) δ 136.2, 133.5, 132.2, 128.2, 127.6, 127.6, 127.5, 127.4, 126.10, 125.4, 66.4, 54.0, 41.0. [4]

(*R*)-2-(Benzylamino)-3-phenylpropan-1-ol (3a)

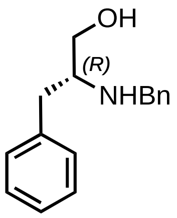

Yield: 56%. ^{1}H NMR (400 MHz, $CDCl_3$) δ 7.62 – 6.81 (m, 10H), 3.81 (s, 2H), 3.68 (dd, *J* = 10.8, 3.9 Hz, 1H), 3.38 (dd, *J* = 10.8, 5.3 Hz, 1H), 3.00-2.94 (m, 1H), 2.88-2.75 (m, 2H), 2.20 (brs, 2H). ^{13}C NMR (101 MHz, $CDCl_3$) δ 139.9, 138.4, 129.2, 128.6, 128.5, 128.0, 127.1, 126.4, 62.4, 59.3, 51.1, 38.1. [5]

(*S*)-2-(Benzylamino)-3-phenylpropan-1-ol (3b)

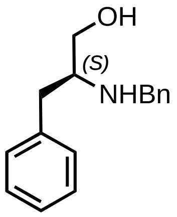

Yield: 64%. ^{1}H NMR (400 MHz, $CDCl_3$) δ 7.62 – 6.81 (m, 10H), 3.81 (s, 2H), 3.67 (dd, *J* = 10.8, 3.9 Hz, 1H), 3.38 (dd, *J* = 10.8, 5.3 Hz, 1H), 3.00-2.96 (m, 1H), 2.88-2.75 (m, 2H), 2.20 (brs, 2H). ^{13}C NMR (101 MHz, $CDCl_3$) δ 139.9, 138.4, 129.2, 128.6, 128.5, 128.0, 127.1, 126.4, 62.4, 59.3, 51.1, 38.1. [6]

(*R*)-2-(Benzylamino)-2-phenylethan-1-ol (3c)

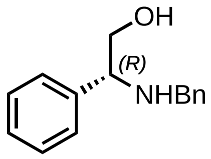

Yield: 85%. ^{1}H NMR (300 MHz, $CDCl_3$) δ 7.43 – 7.29 (m, 10H), 3.67 (dd, *J* = 10.6, 3.9 Hz, 1H), 3.44 (dd, *J* = 10.6, 7.1 Hz, 1H), 3.33 – 3.13 (m, 1H), 2.96 (dd, *J* = 13.2, 5.1 Hz, 1H), 2.68 (dd, *J* = 13.2, 8.7 Hz, 1H), 2.04 (s, 2H).

^{13}C NMR (75 MHz, $CDCl_3$) δ 140.5, 140.1, 128.7, 128.5, 128.2, 127.7, 127.3, 127.1, 66.7, 63.7, 51.2. [7]

(*R*)-2-(Benzylamino)-3-(naphthalen-2-yl)propan-1-ol (3d)

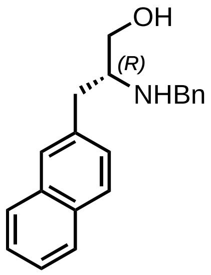

Yield: 80%. ^{1}H NMR (400 MHz, $CDCl_3$) δ 7.90 – 7.77 (m, 3H), 7.64 (s, 1H), 7.55 – 7.45 (m, 2H), 7.36 – 7.20 (m, 6H), 3.83 (s, 2H), 3.72 (dd, *J* = 10.8, 3.8 Hz, 1H), 3.44 (dd, *J* = 10.8, 5.1 Hz, 1H), 3.11 (m, 1H), 3.00 (m, 2H), 2.28 (s, 2H). ^{13}C NMR (101 MHz, $CDCl_3$) δ 139.8, 135.9, 133.5, 132.2, 128.5, 128.2, 128.0, 127.6, 127.6, 127.5, 127.5, 127.1, 126.1, 125.5, 62.4, 59.2, 51.1, 38.2. HRMS (ESI): m/z calcd for [$C_{20}H_{21}NO$+Na+]: 314.1515 [M+Na]+; found 314.1520; $[\alpha]_D^{20}$= +21.8 (c=0.2 in MeOH).

(*2S,5R*)-[4,5-Dibenzylmorpholin-2-yl]methanol (4a)

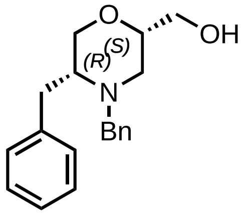

Yield 75%. ^{1}H NMR (400 MHz, $CDCl_3$) δ 7.44 – 7.08 (m, 10H), 3.86, 3.77 (AB system, *J* = 13.3 Hz, 2H), 3.76 – 3.69 (m, 2H), 3.68 – 3.65 (m, 2H), 3.61 (ddd, *J* = 11.2, 2.7, 1.2 Hz, 1H), 3.03 – 2.89 (m, 2H), 2.85 – 2.77 (m, 1H), 2.65 (dd, *J* = 11.6, 10.2 Hz, 1H), 2.50 (dd, *J* = 11.7, 2.9 Hz, 1H) 2.1 (brs, 1H). ^{13}C NMR (101 MHz, $CDCl_3$) δ 140.0, 138.4, 129.4, 128.8, 128.4, 128.4, 127.2, 125.9, 76.1, 67.6, 64.2, 59.1, 59.0, 47.93 28.0. HRMS (ESI): m/z calcd for [$C_{19}H_{23}NO_2$+Na+]: 320.1621 [M+Na]+; found 320.1628; $[\alpha]_D^{20}$= +7.8 (c=0.2 in MeOH).

[(*2S,5R*)-4,5-Dibenzylmorpholin-2-yl]-methanol (4b)

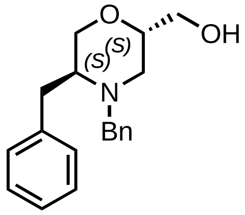

Yield 67%. ^{1}H NMR (400 MHz, $CDCl_3$) δ 7.45 – 7.16 (m, 10H), 4.37 (d, *J* = 13.4 Hz, 1H), 3.73 (dd, *J* = 11.4, 3.3 Hz, 1H), 3.65-3.60 (m, 1H), 3.57-3.40 (m, 3H), 3.27 (dd, *J* = 13.9, 4.0 Hz, 1H), 3.20 (d, *J* = 13.4 Hz, 1H), 2.75 – 2.60 (m, 2H), 2.53 (dd, *J* = 13.9, 9.0 Hz, 1H), 2.17 (brs, 1H), 2.06 (dd, *J* = 11.6, 10.3 Hz, 1H). ^{13}C NMR (101 MHz, $CDCl_3$) δ 138.4, 138.3, 129.1, 129.0, 128.5, 128.4, 127.1, 126.3, 76.1, 70.8, 64.0, 61.2, 58.4, 53.4, 36.1. HRMS (ESI): m/z calcd for [$C_{19}H_{23}NO_2$+Na+]: 320.1621 [M+Na]+; found 320.1622; $[\alpha]_D^{20}$= +95.5 (c=0.2 in MeOH).

[(*2S,5R*)-4-Benzyl-5-phenylmorpholin-2-yl]methanol (4c)

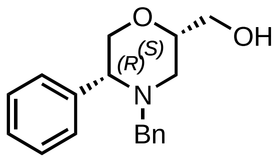

Yield 66%. ^{1}H NMR (400 MHz, $CDCl_3$) δ 7.51-7.45 (m, 2H), 7.41-7.22 (m, 8H), 4.23 (dd, *J* = 11.7, 7.3 Hz, 1H), 4.05 (dd, *J* = 11.8, 9.3 Hz, 1H), 3.92-3.86 (m, 1H), 3.83 – 3.69 (m, 3H), 3.53 (dd, *J* = 9.3, 3.8 Hz, 1H), 2.93 (d, *J* = 13.4 Hz, 1H), 2.85 (dd, *J* = 12.1, 2.6 Hz, 1H), 2.52 (dd, *J* = 12.1, 4.1 Hz, 1H), 1.61 (brs, 1H). ^{13}C NMR (101 MHz, $CDCl_3$) δ 138.8, 137.9, 128.7, 128.6 (x2), 128.4, 128.0, 127.1, 73.2, 68.2, 66.7, 63.7, 59.1, 51.6. [8]

(*2S,5R*)-4-Benzyl-5-(naphthalen-2-yl-methyl)morpholin-2-yl]methanol (4d)

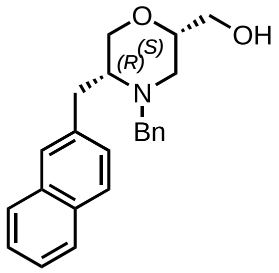

Yield 68%. ^{1}H NMR (400 MHz, $CDCl_3$) δ 7.85 – 7.72 (m, 3H), 7.60 (s, 1H), 7.52 – 7.30 (m, 7H), 7.22 (dd, *J* = 8.4, 1.6 Hz, 1H), 3.92,3.84 (AB system, *J* = 13.3 Hz, 2H), 3.80 – 3.67 (m, 3H), 3.65 – 3.59 (m, 2H), 3.10-3.00 (m, 2H), 2.92 (d, *J* = 10.6 Hz, 1H), 2.75-2,60 (m, 1H) 2.54 (dd, *J* = 11.7, 2.8 Hz, 1H), 2.08 (brs, 1H). ^{13}C NMR (101 MHz, $CDCl_3$) δ 138.4, 137.5, 133.5, 131.9, 128.8, 128.4, 128.0, 127.9, 127.8, 127.62, 127.4, 127.3, 126.0, 125.2, 76.2, 67.6, 64.3, 59.2, 59.0, 47.9, 28.2. HRMS (ESI): m/z calcd for $[C_{23}H_{25}NO_2+Na+]$: 370.1778 [M+Na]+; found 370.1780; $[\alpha]_D^{20}$= +33.1 (c=0.2 in MeOH)

Butyl (*2S,5R*)-5-benzyl-2-(hydroxymethyl)morpholine-4-carboxylate (5a)

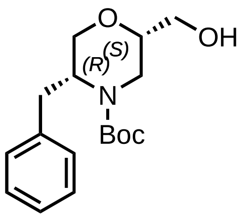

Yield 81%. Mixture of rotamers (6:4). ^{1}H NMR (400 MHz, $CDCl_3$) δ 7.40 -7.20 (m, 5H), 4.10-4.01 (4.21-4.16) (m, 1H), 3.93 (dd, *J* = 13.4, 2.4 Hz, 0.6H), 3.83-3.47 (m, 5.4H), 3.11 – 2.82 (m, 3H), 2.09 (1,68) (brs, 1H), 1.37 (1.44) (s, 9H). ^{13}C NMR (101 MHz, $CDCl_3$) δ 154.6, 138.5, (138.2), (129.5), 129.4, 128.5, (128.4), 126.4, (126.3), 80.0, 76.3, (75.8), 67.8, (67.4), 63.7, 53.1, (51.5), (41.3), 39.7, 35.3, (34.7), (28.3), 28.2[1]. HRMS (ESI): m/z calcd for $[C_{17}H_{25}NO_4+]$: 307.1787 [M+H]+; found: 307.1785; $[\alpha]_D^{20}$= +23.4 (c=0.2 in MeOH).

[1] in brackets the signals of the minor conformer

tButyl (2S,5S)-5-benzyl-2-(hydroxymethyl)morpholine-4-carboxylate (5b)

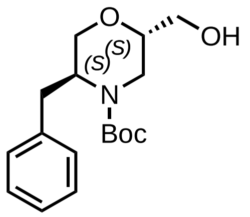

Yield 88%. ^{1}H NMR (400 MHz, $CDCl_3$) δ 7.64 – 6.85 (m, 5H), 4.20-4.10 (m, 1H), 4.02 – 3.93 (m, 1H), 3.92-3.84 (m, 1H), 3.80-3.74(m, 2H), 3.65-3.55 (m, 1H), 3.51 (dd, *J* = 12.0, 2.8 Hz, 1H), 3.42 (dd, *J* = 14.2, 4.8 Hz, 1H), 3.08 – 2.91 (m, 2H), 2.17 (brs, 1H), 1.42 (s, 9H). ^{13}C NMR (101 MHz, $CDCl_3$) δ 155.2, 138.1, 129.3, 128.5, 126.5, 80.2, 72.3, 61.4, 60.3, 53.3, 38.43, 35.7, 28.3. HRMS (ESI): m/z calcd for [$C_{17}H_{25}NO_4$+]: 307.1787 [M+H]+; found: 307.1787; $[\alpha]_D^{20}$= + 4.6 (c=0.2 in MeOH).

tButyl (2S,5R)-2-(hydroxymethyl)-5-phenylmorpholine-4-carboxylate (5c)

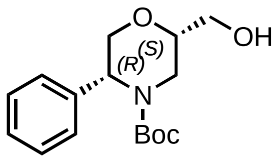

Yield 87%. Mixture of rotamers (8:2) ^{1}H NMR (400 MHz, $CDCl_3$) δ 7.50-7.42 (m, 2H), 7.41 – 7.21 (m, 3H), 5.06 (5.17) (brs, 1H), 4.47 (d, *J* = 10.4 Hz, 1H), 3.98 (dd, *J* = 12.0, 3.7 Hz, 1H), 3.93 – 3.13 (m, 4H), 2.92-2.81 (m, 1H), 1.66 (1.95) (brs, 1H), 1.49 (1.46) (s, 9H). ^{13}C NMR (101 MHz, $CDCl_3$) δ 154.8, 139.2, (138.9), (128.8), 128.5, (127.5), 127.2, (126.5), 126.4, 80.5, 78.7, 73.7, (70.5), 68.9, (65.4), 63.7, 56.3, (40.0), 36.6, 28.4, (28.3).[1] HRMS (ESI): m/z calcd for [$C_{16}H_{23}NO_4$+]: 293.1627 [M+H]+; found: 293.1624; $[\alpha]_D^{20}$= +107.3 (c=0.2 in MeOH).

tButyl (2S,5R)-2-(hydroxymethyl)-5-(naphthalen-2-yl-methyl)morpholine-4-carboxylate (5d)

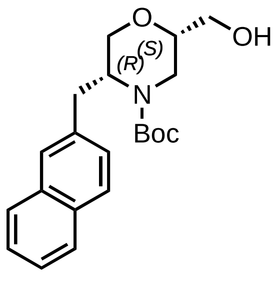

Yield 76%. ^{1}H NMR (400 MHz, $CDCl_3$) δ 7.86 - 7.78 (m, 3H), 7.66 (s, 1H), 7.52-7.42 (m, 2H), 7.36 (d, *J* = 8.1 Hz, 1H), 4.31 - 4.16 (m, 1H), 3.91-3.66 (m, 3H), 3.59 (m, 2H), 3.28 – 2.94 (m, 3H), 2.07 (s, 1H), 1.68 (s, 1H), 1.38 (s, 9H). ^{13}C NMR (101 MHz, $CDCl_3$) δ 154.6, 135.9, (135.8), 133.6, 132.2, 128.2, 128.1, (128.0), 127.9, (127.7), 127.60, 127.5, 126.1, (125.9), 125.5, (125.4), 80.1, (76.4), 75.8, 67.7, (67.2), 63.8, 53.1, (51.4), (41.4), 39.8, 35.5, (34.8), (28.3), 28.1.[1] HRMS (ESI): m/z calcd for [$C_{21}H_{27}NO_4$+]: 357.1940 [M+H]+; found: 358.1938; $[\alpha]_D^{20}$= +52.6 (c=0.2 in MeOH).

(2S,5R)-5-Benzyl-4-(tbutoxycarbonyl)morpholine)-2-carboxylic acid (6a)

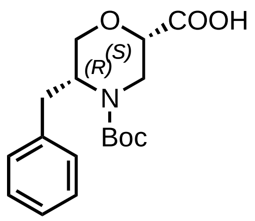

Yield 71%. ^{1}H NMR (400 MHz, $CDCl_3$) δ 7.36 – 7.19 (m, 5H), 5.90 (brs, 1H), 4.40-4.10 (m, 2H), 4.12 (dd, *J* = 11.4, 3.4 Hz, 1H), 3.93 (dd, *J* = 11.7, 1.0 Hz, 1H), 3.64 (dd, *J* = 11.7, 3.4 Hz, 1H), 3.19 (dd, *J* = 13.7, 11.4 Hz, 1H), 3.05 – 2.89 (m, 2H), 1.42 (s, 9H). ^{13}C NMR (101 MHz, $CDCl_3$) δ 171.6, 154.3, 138.0, 129.4, 128.6, 126.6, 80.1, 74.2, 68.1, 52.8, 40.0, 35.0, 28.2. HRMS (ESI): m/z calcd for [$C_{17}H_{23}NO_5$+Na+]: 344.1468 [M+Na]+; found 344.1465; $[\alpha]_D^{20}$= +19.4 (c=0.2 in MeOH).

(2S,5S)-5-Benzyl-(tbutoxycarbonyl)morpholine-2-carboxylic acid (6b)

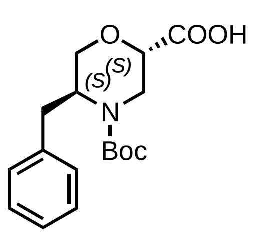

Yield 66%. ^{1}H NMR (400 MHz, $CDCl_3$) δ 7.43 – 7.11 (m, 5H), 5.03 (brs, 1H), 4.62 – 4.31 (m, 2H), 4.20-4.15 (m, 1H), 3.99 (dd, *J* = 12.1, 3.7 Hz, 1H), 3.64 (dd, *J* = 12.1, 1.6 Hz, 1H), 3.52 (dd, *J* = 14.2, 5.3 Hz, 1H), 3.00 (t, *J* = 7.4 Hz, 2H), 1.39 (s, 9H). ^{13}C NMR (101 MHz, $CDCl_3$) δ 173.9, 154.6, 137.9, 129.4, 128.6, 126.5, 80.7, 71.3, 63.7, 52.4, 39.1, 34.8, 28.2. HRMS (ESI): m/z calcd for [$C_{17}H_{23}NO_5$+Na+]: 344.1468 [M+Na]+; found 344.1470; $[\alpha]_D^{20}$= + 42.5 (c=0.2 in MeOH).

(2*S*,5*R*)-4-(*t*Butoxycarbonyl)-5-phenyl-morpholine-2-carboxylic acid (6c)

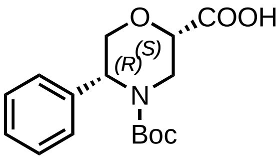

Yield 65%. ^{1}H NMR (400 MHz, MeOD) δ 7.45 (d, *J* = 7.6 Hz, 2H), 7.37 (t, *J* = 7.4 Hz, 2H), 7.29 (d, *J* = 7.0 Hz, 1H), 5.11 (s, 1H), 4.48 (d, *J* = 11.6 Hz, 1H), 4.25 (d, *J* = 13.3 Hz, 1H), 4.13 (d, *J* = 11.1 Hz, 1H), 3.98 (d, *J* = 11.0 Hz, 1H), 3.00 (t, *J* = 12.2 Hz, 1H), 1.49 (s, 9H). ^{13}C NMR (101 MHz, MeOD) δ 172.2, 155.0, 138.9, 128.6, 127.1, 127.0, 80.6, 68.5, 52.8, 41.5, 29.5, 27.2. HRMS (ESI): m/z calcd for [$C_{16}H_{21}NO_5$+Na+]: 330.1312 [M+Na]+; found 331.1318; $[\alpha]_D^{20}$= – 63.6 (c=0.2 in MeOH).

(2*S*,5*R*)-4-(*t*Butoxycarbonyl)-5-(naphthalen-2-yl-methyl)morpholine-2-carboxylic acid (6d)

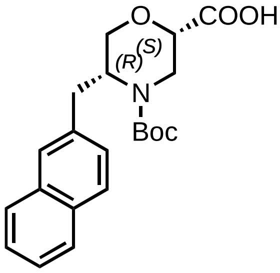

Yield 71%. ^{1}H NMR (400 MHz, MeOD) δ 7.88 – 7.76 (m, 3H), 7.70 (s, 1H), 7.53 – 7.34 (m, 3H), 4.34- 4.24 (m, 2H), 4.10 (d, *J* = 10.9 Hz, 1H), 3.95 (d, *J* = 11.7 Hz, 1H), 3.75-3.6 (m, 1H), 3.32 – 2.96 (m, 3H), 1.32 (s, 9H). ^{13}C NMR (101 MHz, MeOD) δ 171.0, 154.6, 134.8, 133.7, 132.4, 127.7, 127.5, 127.2, 127.1, 125.6 (x2), 125.1, 80.1, 74.3, 68.5, 53.0, 29.3, 26.9, 26.7. HRMS (ESI): m/z calcd for [$C_{21}H_{25}NO_5$+Na+]: 394.1625 [M+Na]+; found 394.1628; $[\alpha]_D^{20}$= + 13.6 (c=0.2 in MeOH).

(2*S*,5*R*)-5-Benzyl-morpholine-2-carboxilic acid TFA salt I

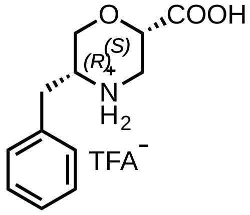

Yield: quantitative. ^{1}H NMR (400 MHz, MeOD) δ 7.39 – 7.31 (m, 5H), 4.51 (t, *J* = 4.8 Hz, 1H), 3.95 – 3.76 (m, 2H), 3.73 – 3.57 (m, 2H), 3.56 – 3.40 (m, 1H), 3.09-2.95 (m, 2H). ^{13}C NMR (101 MHz, MeOD) δ 170.5, 134.7, 128.9, 128.7, 127.2, 70.6, 64.1, 54.4, 42.0, 33.7. HRMS (ESI): m/z calcd for [$C_{12}H_{15}NO_3$+Na+]: 244.0944 [M+Na]+; found 244.0947; $[\alpha]_D^{20}$ = +20.4 (c = 0.2 in MeOH).

(2*S*,5*S*)-5-Benzyl-morpholine2-carboxilic acid TFA salt II

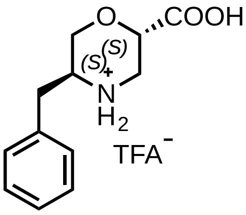

Yield: quantitative. ^{1}H NMR (400 MHz, MeOD) δ 7.40 – 7.27 (m, 5H), 4.25 (d, *J* = 10.6 Hz, 1H), 4.06 (d, *J* = 10.6 Hz, 1H), 3.68 – 3.57 (m, 3H), 3.12 (t, *J* = 12.0 Hz, 1H), 2.91 – 2.86 (m, 2H). ^{13}C NMR (101 MHz, MeOD) δ 169.6, 134.2, 128.9, 128.8, 127.3, 72.0, 67.6, 55.1, 44.9, 34.6. HRMS (ESI): m/z calcd for [$C_{12}H_{15}NO_3$+Na+]: 244.0944 [M+Na]+; found 244.0945; $[\alpha]_D^{20}$= – 18.0 (c=0.2 in MeOH).

(2*S*,5*R*)-5-phenylmorpholine-2-carboxylic acid TFA salt III

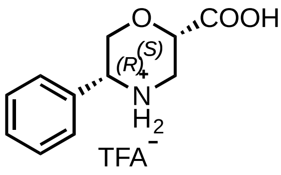

Yield: quantitative. ^{1}H NMR (400 MHz, MeOD) δ 7.58 – 7.38 (m, 5H), 4.60 (t, *J* = 4.0 Hz, 1H), 4.55 (dd, *J* = 9.0, 3.6 Hz, 1H), 4.39 (dd, *J* = 13.0, 8.9 Hz, 1H), 4.08 (dd, *J* = 13.0, 3.6 Hz, 1H), 3.64 (ddd, *J* = 17.5, 12.8, 3.9 Hz, 2H). ^{13}C NMR (101 MHz, MeOD) δ 171.2, 132.6, 129.7, 129.0, 127.9, 69.9, 64.8, 56.9, 42.9. HRMS (ESI): m/z calcd for

[$C_{11}H_{13}NO_3$+Na+]: 230.0788 [M+Na]+; found 230.0786; $[\alpha]_D^{20}$= + 25.3 (c=0.2 in MeOH).

(*2S,5R*)-5-(naphthalen-2-ylmethyl)Morpholine 2-carboxilic acid TFA salt IV

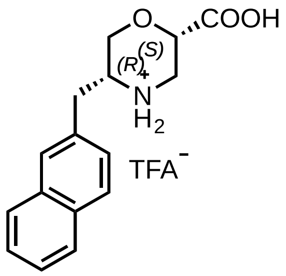

Yield: quantitative. ^{1}H NMR (400 MHz, MeOD) δ 7.96 – 7.73 (m, 4H), 7.62 – 7.40 (m, 3H), 4.47 (s, 1H), 4.04 – 3.81 (m, 2H), 3.81 – 3.61 (m, 2H), 3.57 – 3.39 (m, 1H), 3.19 (ddd, *J* = 20.1, 13.7, 7.4 Hz, 2H). ^{13}C NMR (101 MHz, MeOD) δ 170.8, 133.7, 132.8, 132.1, 128.6, 128.0, 127.3, 127.2, 126.5, 126.1, 125.80, 71.0, 64.3, 54.3, 42.2, 33.9. HRMS (ESI): m/z calcd for [$C_{16}H_{17}NO_3$+Na+]: 294.1101 [M+Na]+; found 294.1111; $[\alpha]_D^{20}$= + 11.2 (c=0.2 in MeOH).

Synthesis γ-Nitroaldehydes 9-22

Catalyst[a,b](1-5 mol%; see Table 2), was added to a solution of *N*-methylmorpholine (1-5 mol%),[c] nitroolefin **8a-f** (1.5 eq., 0.17 mmol) and aldehyde **7a-f** (1 eq., 0.11 mmol). *i*PrOH (0.380 mL). The reaction mixture was stirred at –10 °C for 24-48 h (see Table 2). The solvent was removed under reduced pressure and the crude mixture was subjected to flash chromatography (silica gel; 5% → 20% EtOAc in hexane,) to yield γ-nitroaldehyde **9-22**. The diastereomeric ratio was determined by ^{1}H NMR spectroscopic analysis of the crude mixture by comparison of the aldehyde R-C*H*O signals. The enantiomeric excess was determined by chiral stationary phase HPLC.

[a]The catalyst was indicated in the manuscript as **I**, **II, III**, and **IV**.

[b]To evaluate the importance of the free carboxylic group in the catalysis, a further attempt was performed by using (2*S*,5*R*)-methyl-5-benzylmorpholine-2-carboxylate as catalyst using aldehyde **7a** and **8a**. In this case, no formation of desired product **9** was observed.

[c]The used amount of the base is according to the amount of the catalyst.

Diastereoisomeric and Enantiomeric excess analyses for compound 9-22

The diastereomeric ratio was determined by ^{1}H NMR spectroscopic analysis of the crude mixture by comparison of the aldehyde [R-C*H*O] signals.

The enantiomeric excess was determined by chiral stationary phase HPLC and was compared with racemic mixture of **9-22** prepared by using racemic Proline as catalyst according to the above described procedure for catalysts **I-IV**.

(2*R*,3*S*)-2-Ethyl-4-nitro-3-phenyl-butanal (9)

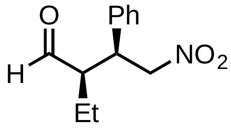

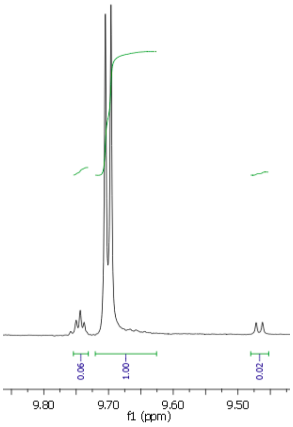

Figure S3: Magnification of the Aldehyde area of ^{1}H NMR Spectrum of compound **9**

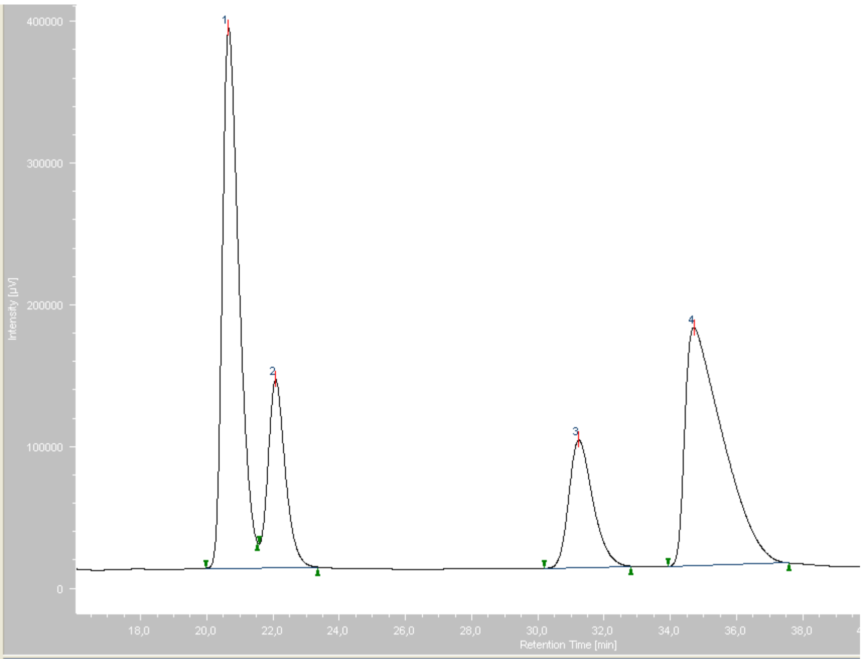

Peak Info. Table: butanale racemo

#	Peak Name	CH	tR	Area	Height	Area%	Height%	Quantity	NTP	Resolution	Symmetry Factor	Warning
1	Unknown	1	20,650	13461994	380453	37,190	49,392	N/A	7943	1,539	1,585	
2	Unknown	1	22,075	4831321	132389	13,347	17,187	N/A	9017	8,186	N/A	
3	Unknown	1	31,217	4535674	89729	12,530	11,649	N/A	9090	2,032	1,350	
4	Unknown	1	34,692	13369205	167704	36,933	21,772	N/A	4284	N/A	2,595	

Figure S4: Racemic mixture of **9**, evaluated by HPLC.

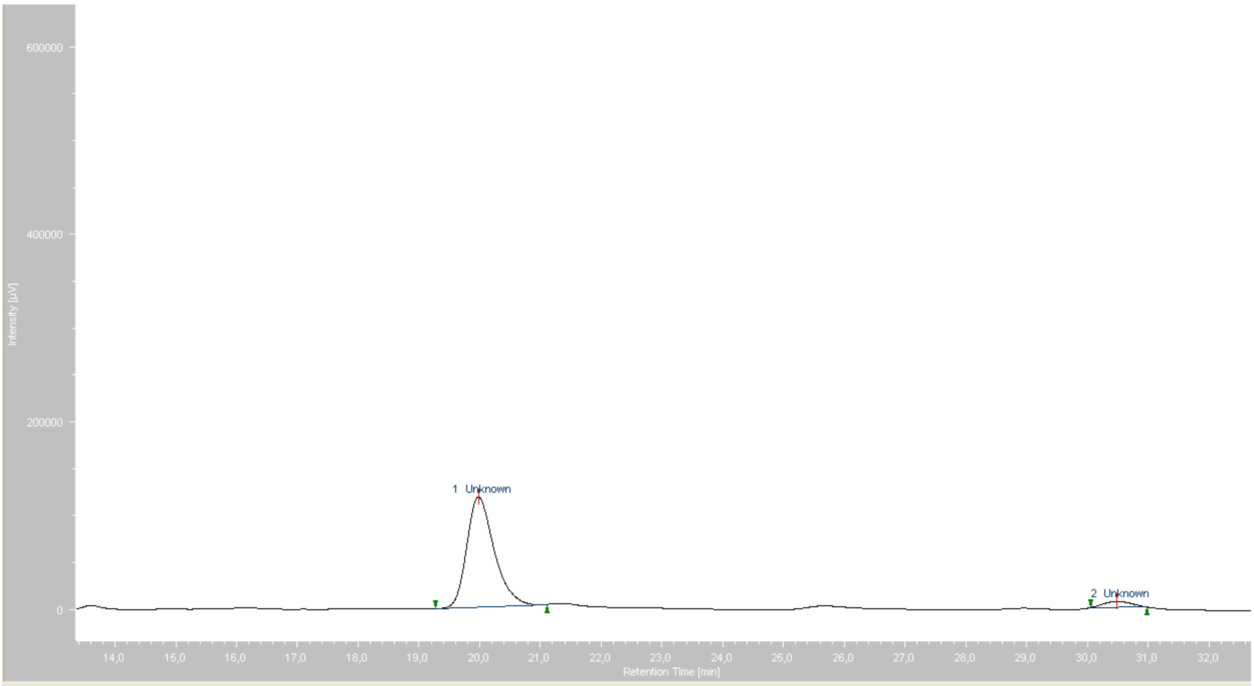

Peak Info. Table

#	Peak Name	tR	Area	Height	Area%	Height%	Peak Start	Peak End	Base Start	Base End	Peak Mark
1	Unknown	19,975	3680065	117308	94,942	95,026	19,267	21,100	19,267	21,100	Manual
2	Unknown	30,475	196054	6140	5,058	4,974	30,050	30,967	30,050	30,967	Manual

Figure S5: Enantiomeric excess of **9**, evaluated by HPLC of crude reaction sample.

Conversion = quantitative; yield = 90%; *d.r.* = 96%, *e.e.* = 92%. The enantiomeric excess was determined by chiral stationary phase HPLC: Chiralcel AD, hexane/EtOH 110:5, 0.8 mL/min, 25 °C, 220 nm, 19.9 min (*syn*, major), 30.4 min (*syn*, minor).

The NMR characterization is in agreement with the literature data. [10]

Synthesis of (*2R,3S*)-2-methyl-4-nitro-3-phenylbutanal (10)

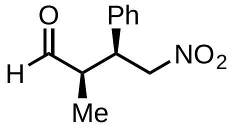

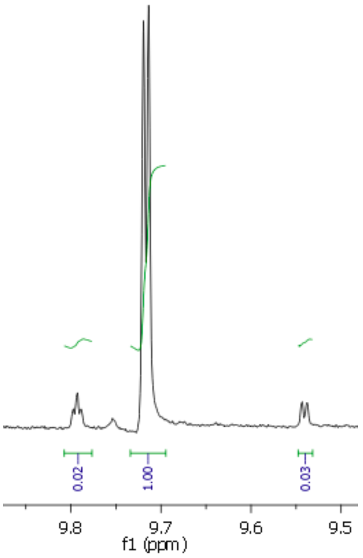

Figure S6: Magnification of the Aldehyde area of ^{1}H NMR Spectrum of compound **10**

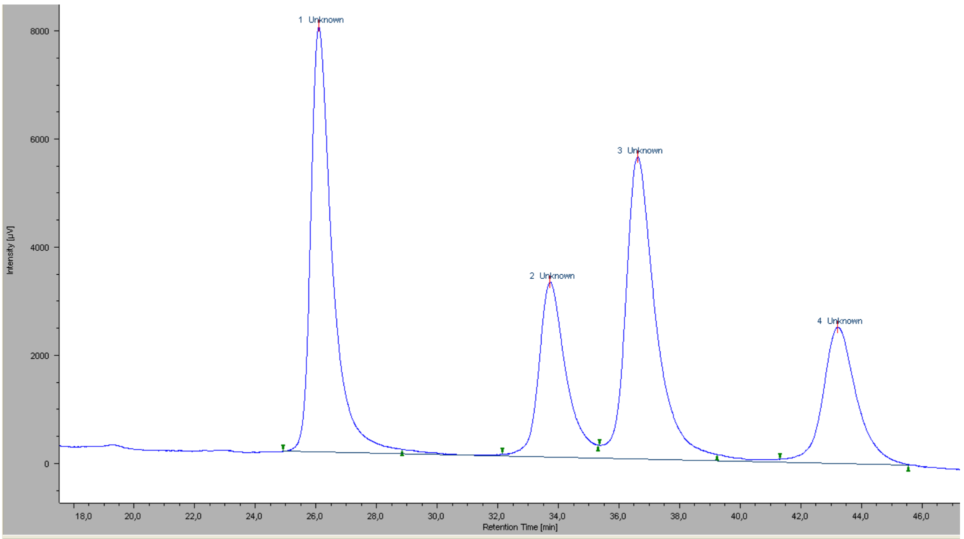

Peak Info. Table

#	Peak Name	tR	Area	Height	Area%	Height%	Peak Start	Peak End	Base Start	Base End	Peak Mark
1	Unknown	26,083	388741	7860	33,310	40,992	24,908	28,833	24,908	45,533	Manual
2	Unknown	33,708	201133	3229	17,234	16,839	32,150	35,300	24,908	45,533	Manual
3	Unknown	36,608	384416	5575	32,939	29,073	35,342	39,225	24,908	45,533	Manual
4	Unknown	43,200	192753	2511	16,516	13,095	41,283	45,533	24,908	45,533	Manual

Figure S7: Racemic mixture of **10**, evaluated by HPLC.

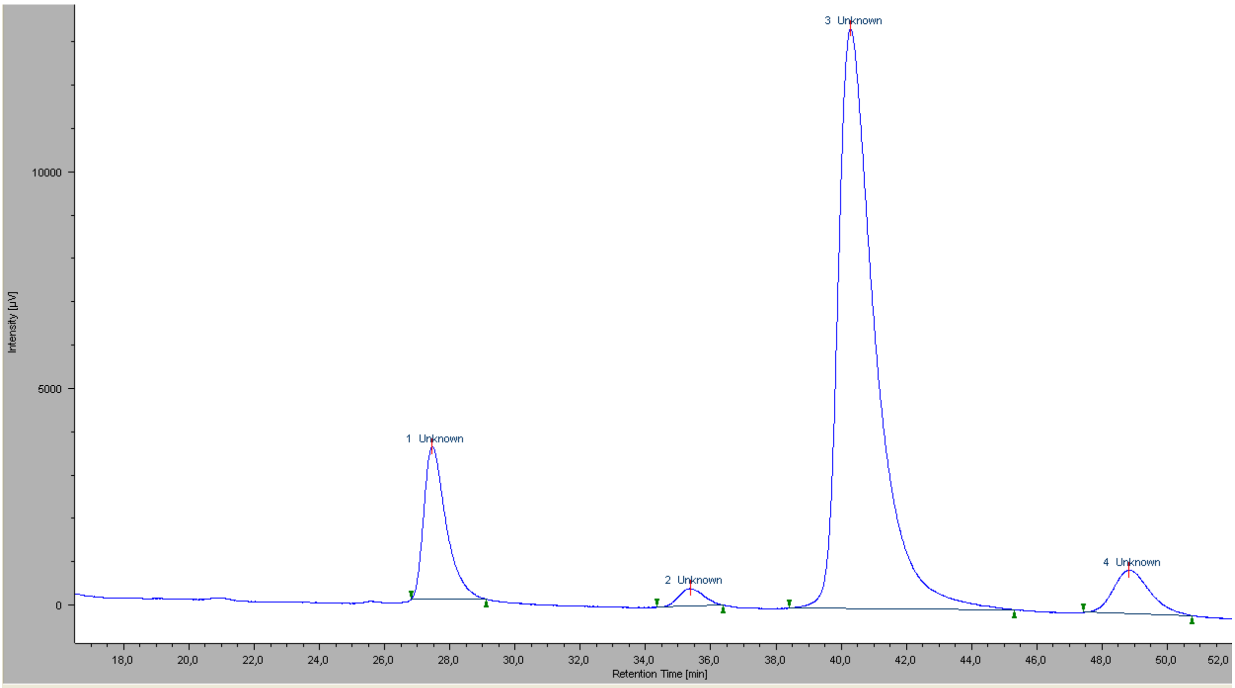

Peak Info. Table

#	Peak Name	tR	Area	Height	Area%	Height%	Peak Start	Peak End	Base Start	Base End	Peak Mark
1	Unknown	27,450	166628	3518	12,632	19,242	26,817	29,108	26,817	29,108	Manual
2	Unknown	35,350	21123	403	1,601	2,203	34,325	36,358	34,325	36,358	Manual
3	Unknown	40,267	1053290	13359	79,847	73,078	38,392	45,283	38,392	45,283	Manual
4	Unknown	48,792	78091	1001	5,920	5,476	47,400	50,717	47,400	50,717	Manual

Figure S8: Enantiomeric excess of **10**, evaluated by HPLC of crude reaction sample

Conversion = quantitative; yield = 95%; *d.e.* = 94%, *e.e.* = 73%. The enantiomeric excess was determined by chiral stationary phase HPLC: Chiralcel OD-H, *n*hexane/*i*PrOH 9 : 1; 1.0 mL/min; 25 °C; 254 nm, 27.4 min(*syn*, minor), 40.2 min (*syn*, major).

The NMR characterization and the HPLC chromatogram are in agreement with the literature data. [9]

Synthesis of (*R*)-2-((*S*)-2-nitro-1-phenylethyl)pentanal (11)

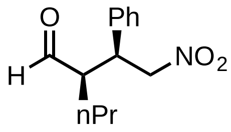

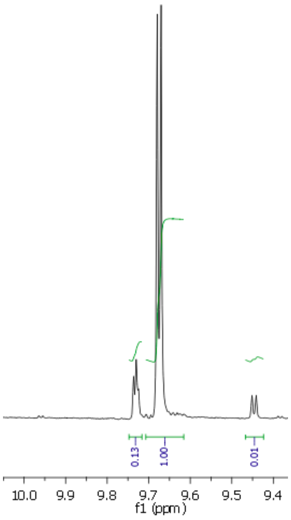

Figure S9: Magnification of the Aldehyde area of ^{1}H NMR Spectrum of compound **11**

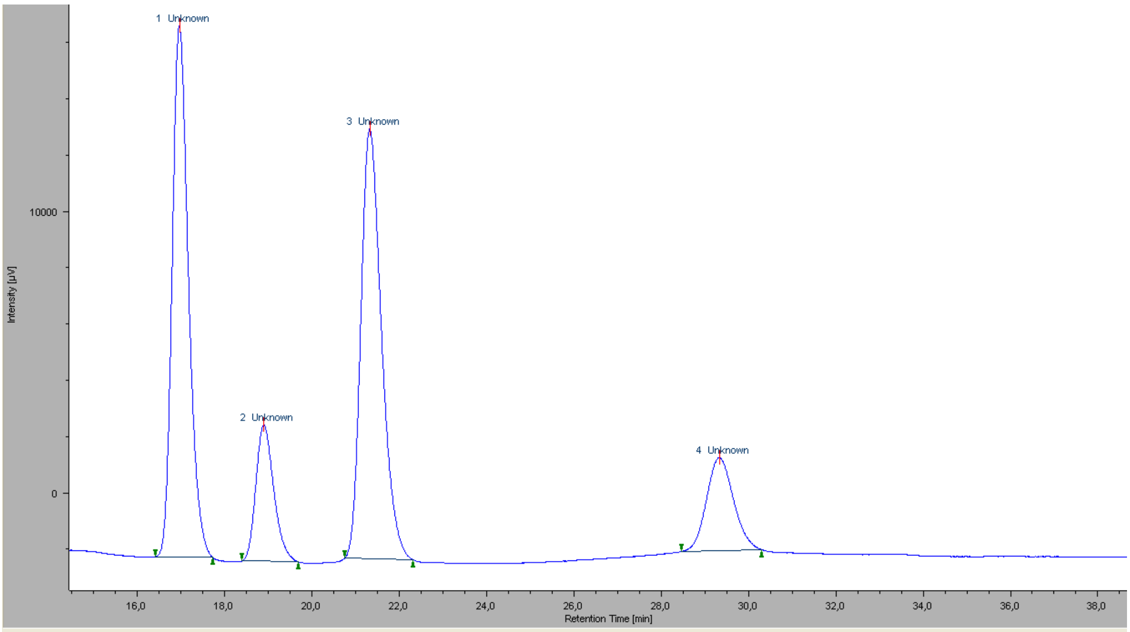

Peak Info. Table

#	Peak Name	tR	Area	Height	Area%	Height%	Peak Start	Peak End	Base Start	Base End	Peak Mark
1	Unknown	16,967	487388	18856	38,546	44,665	16,417	17,725	16,417	17,725	Manual
2	Unknown	18,900	137872	4840	10,904	11,464	18,392	19,683	18,392	19,683	Manual
3	Unknown	21,325	498529	15233	39,427	36,082	20,750	22,300	20,750	22,300	Manual
4	Unknown	29,325	140656	3288	11,124	7,788	28,442	30,275	28,442	30,275	Manual

Figure S10: Racemic mixture of **11**, evaluated by HPLC.

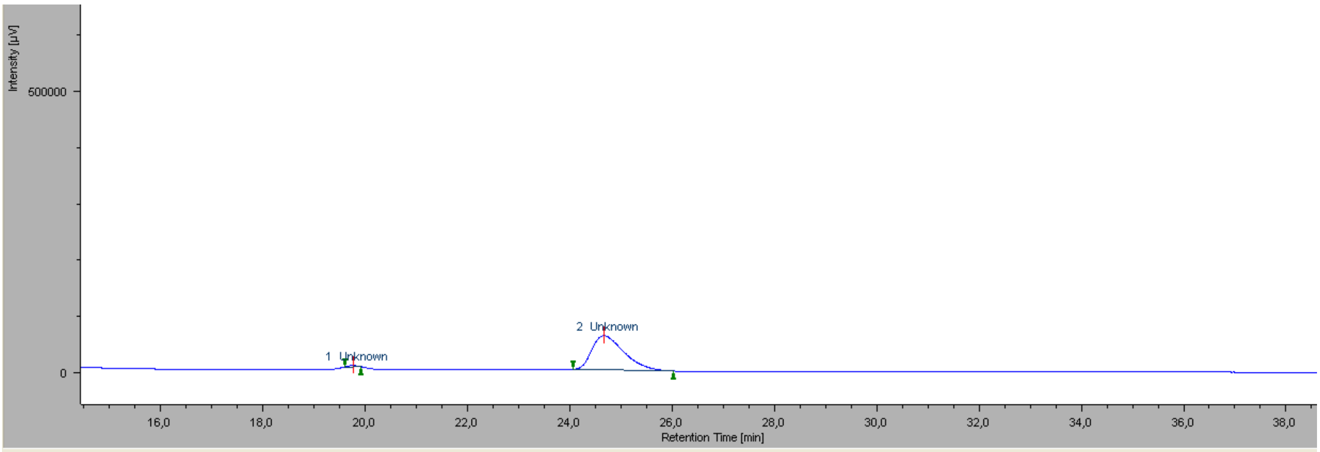

Peak Info. Table

#	Peak Name	tR	Area	Height	Area%	Height%	Peak Start	Peak End	Base Start	Base End	Peak Mark
1	Unknown	19,758	26454	2100	0,988	3,350	19,592	19,917	19,592	19,917	Manual
2	Unknown	24,658	2651611	60593	99,012	96,650	24,067	26,008	24,067	26,008	Manual

Figure S11: Enantiomeric excess of **11**, evaluated by HPLC of crude reaction sample

Conversion = 90%; yield = 86%; *d.r.* = 99%, *e.e.* = 99%. The enantiomeric excess was determined by chiral stationary phase HPLC: Chiralcel OD-H, hexane/iPrOH 9:1, 1.0 ml/min, 25 °C, 254 nm, 19.7. min (*syn*, major), 24.6 min (*syn*, minor).

The NMR characterization and the HPLC chromatogram are is in agreement with the literature data. [10]

Synthesis of (*2R,3S*)-2-isopropyl-4-nitro-3-phenylbutanal (12)

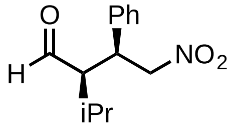

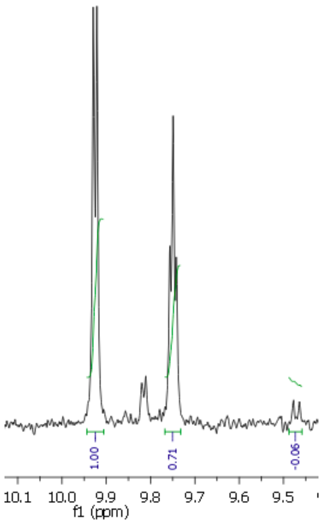

Figure S12: Magnification of the Aldehyde area of ^{1}H NMR Spectrum of compound **12** with 1% of catalyst **I**

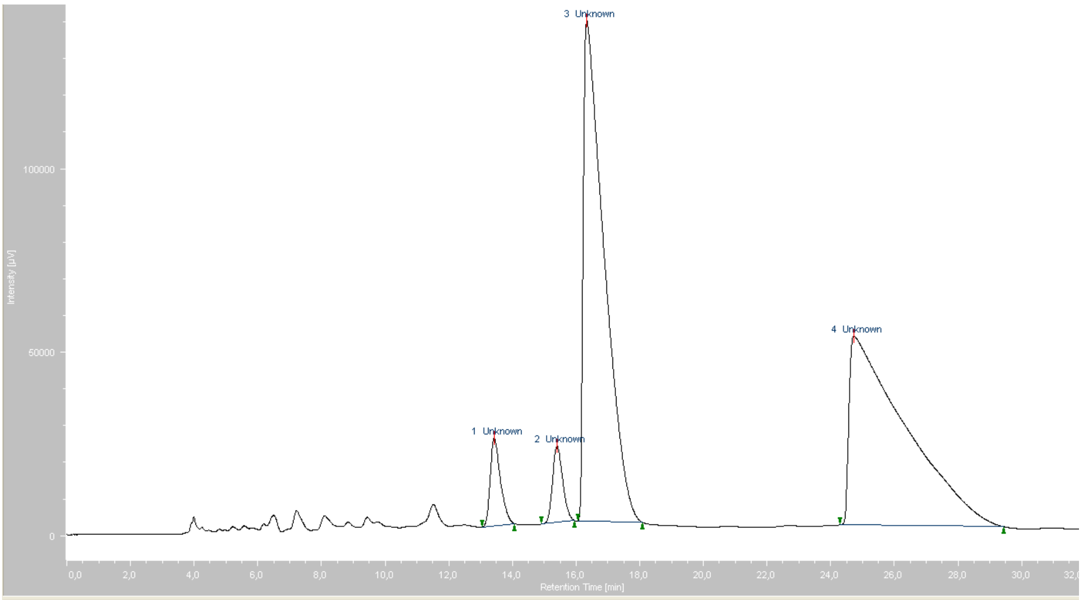

Peak Info. Table

#	Peak Name	tR	Area	Height	Area%	Height%	Peak Start	Peak End	Base Start	Base End	Peak Mark
1	Unknown	13,417	529652	23871	3,926	10,281	13,042	14,042	13,042	14,042	Manual
2	Unknown	15,392	458789	20766	3,401	8,944	14,908	15,925	14,908	15,925	Manual
3	Unknown	16,325	6193393	136179	45,912	58,652	16,042	18,058	16,042	18,058	Manual
4	Unknown	24,708	6307739	51366	46,760	22,123	24,275	29,400	24,275	29,400	Manual

Figure S13: Racemic mixture of **12**, evaluated by HPLC.

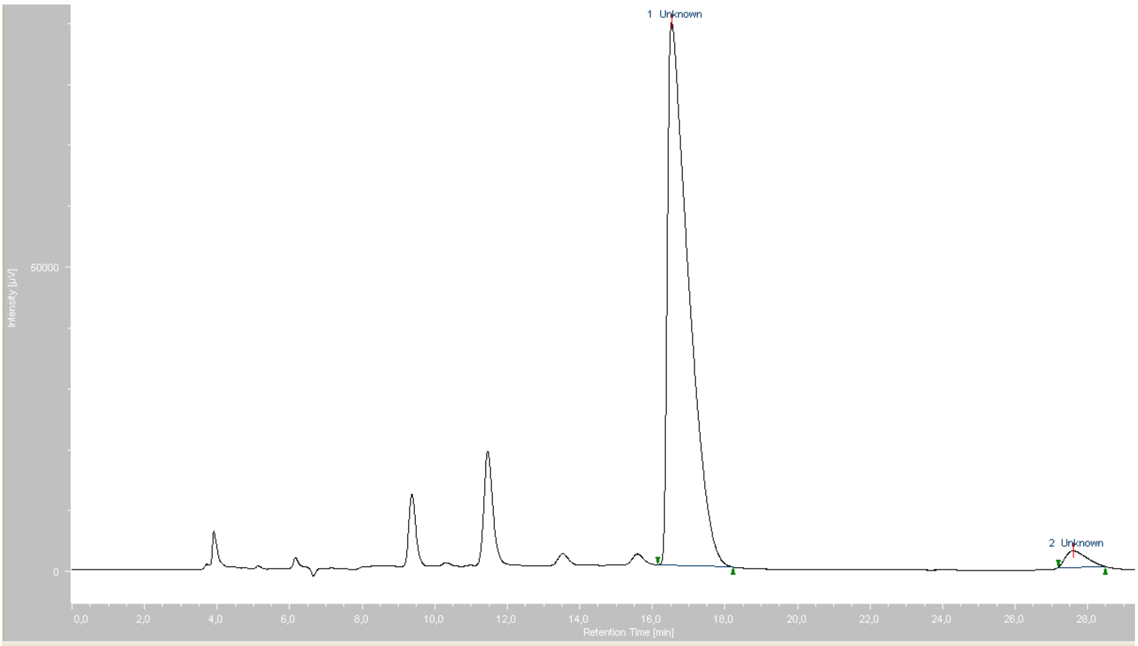

Peak Info. Table

#	Peak Name	tR	Area	Height	Area%	Height%	Peak Start	Peak End	Base Start	Base End	Peak Mark
1	Unknown	16,517	3592592	89091	97,095	97,107	16,133	18,208	16,133	18,208	Manual
2	Unknown	27,583	107483	2654	2,905	2,893	27,167	28,467	27,167	28,467	Manual

Figure S14: Enantiomeric excess of **12**, evaluated by HPLC of crude reaction sample

Loading catalyst: 1 mol %
Conversion = 68%, yield = 58%; *d.r.* = 99%, *e.e.* = 95%. The enantiomeric excess was determined by chiral stationary phase HPLC Chiralcel AD, hexane/iPrOH 9:1, 0.8 ml/min, 25 °C, 254 nm, 16.5 min (*syn*, major), 27.5 min (*syn*, minor).

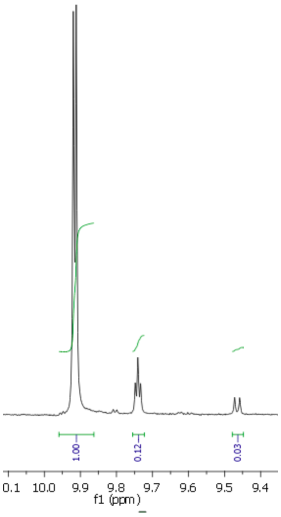

Figure S15: **Magnification of the Aldehyde area of ^{1}H NMR Spectrum of compound** 12 **with 5% of catalyst** I

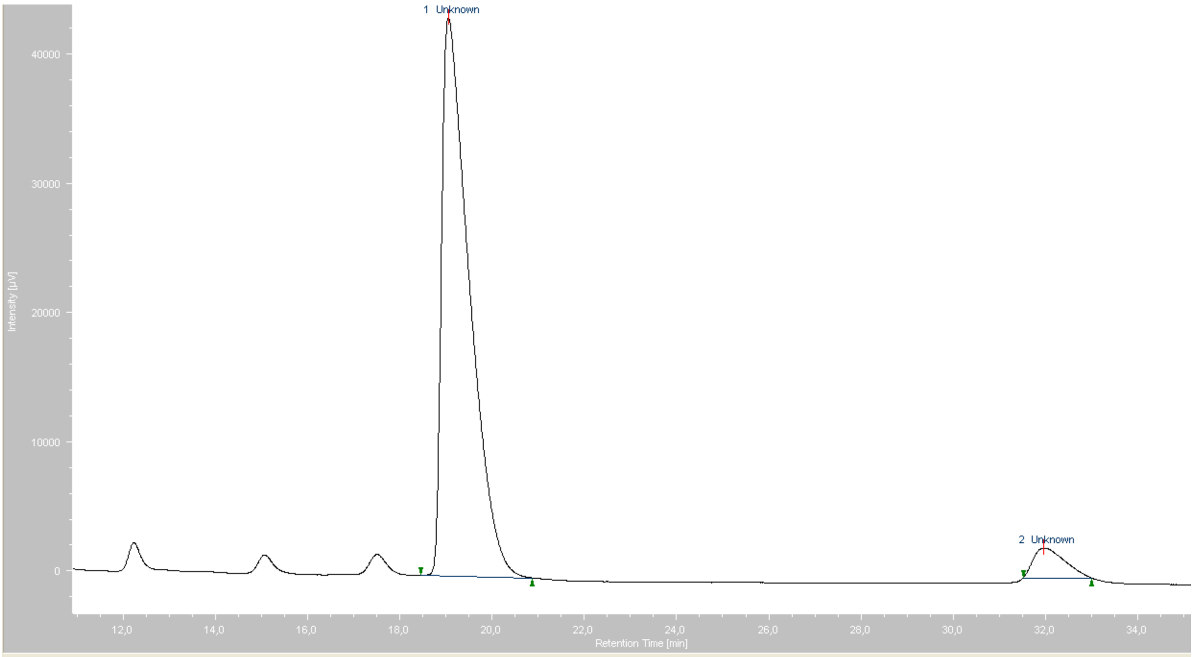

Peak Info. Table

#	Peak Name	tR	Area	Height	Area%	Height%	Peak Start	Peak End	Base Start	Base End	Peak Mark
1	Unknown	19,042	1795090	43141	94,335	94,931	18,442	20,867	18,442	20,867	Manual
2	Unknown	31,950	107805	2304	5,665	5,069	31,500	32,975	31,500	32,975	Manual

Figure S16: Enantiomeric excess of **12**, evaluated by HPLC of crude reaction sample

Loading catalyst: 5 mol %

Conversion = 91%, yield = 82%; *d.r.* = 94%, *e.e.* = 90%. The enantiomeric excess was determined by chiral stationary phase HPLC Chiralcel AD, hexane/iPrOH 9:1, 0.8 ml/min, 25 °C, 254 nm, 19.0 min (*syn*, major), 31.9 min (*syn*, minor).

The NMR characterization and the HPLC chromatogram are in agreement with the literature data.[10]

Synthesis of (*R*)-2-((*S*)-2-nitro-1-phenylethyl)hexanal (13)

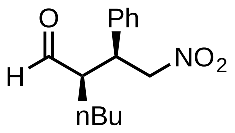

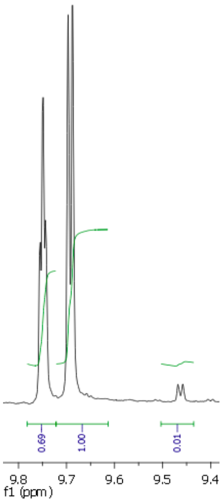

Figure S17: Magnification of the Aldehyde area of ^{1}H NMR Spectrum of compound **13**

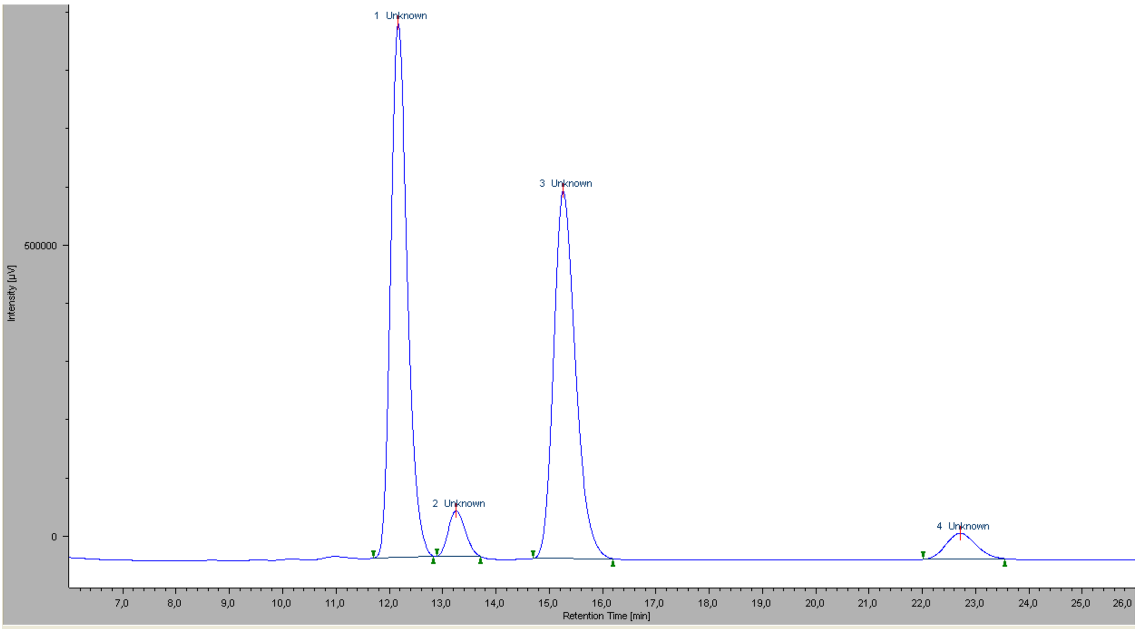

Peak Info. Table

#	Peak Name	tR	Area	Height	Area%	Height%	Peak Start	Peak End	Base Start	Base End	Peak Mark
1	Unknown	12,158	19800104	916233	48,070	54,874	11,700	12,817	11,700	12,817	Manual
2	Unknown	13,250	1711795	78173	4,156	4,682	12,883	13,700	12,883	13,700	Manual
3	Unknown	15,258	17964052	631114	43,612	37,798	14,692	16,183	14,692	16,183	Manual
4	Unknown	22,692	1714313	44184	4,162	2,646	22,008	23,525	22,008	23,525	Manual

Figure S18: Racemic mixture of **13**, evaluated by HPLC.

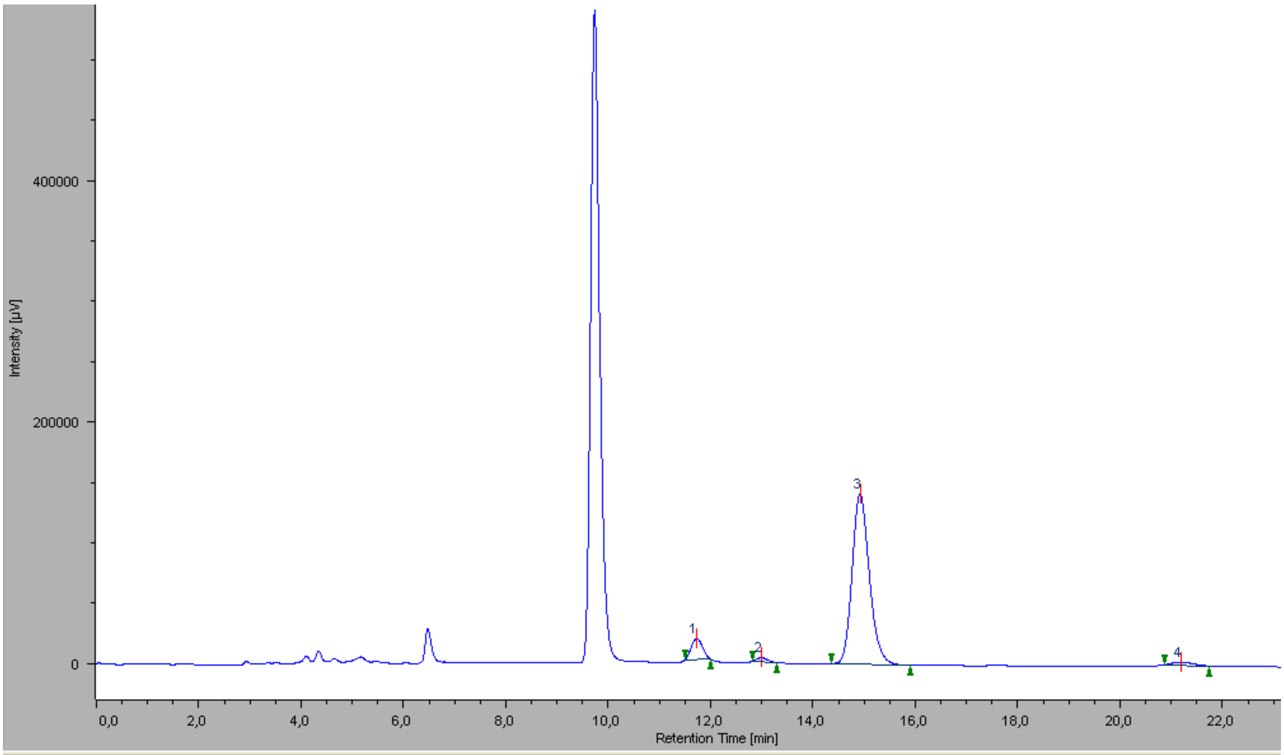

Peak Info. Table: MB159_hexa_ipa_meno10_dil

#	Peak Name	CH	tR	Area	Height	Area%	Height%	Quantity	NTP	Resolution	Symmetry Factor	Warning
1	Unknown	1	11,717	271132	17450	7,329	10,649	N/A	11782	3,016	1,086	
2	Unknown	1	12,983	44018	3014	1,190	1,839	N/A	16006	3,788	1,316	
3	Unknown	1	14,908	3312206	140698	89,537	85,862	N/A	9599	9,280	1,181	
4	Unknown	1	21,175	71893	2704	1,943	1,650	N/A	12907	N/A	1,291	

Figure S19: Enantiomeric excess of **13**, evaluated by HPLC of crude reaction sample

Conversion =60%, yield = 52%; *d.r.* = 98%, *e.e.* = 87%. The enantiomeric excess was determined by chiral stationary phase HPLC: Chiralcel OD-H, hexane/iPrOH 9:1, 1.0 ml/min, 25 °C, 216 nm, 11.7 min (*syn*, minor), 14.9 min (*syn*, major).
The NMR characterization and the HPLC chromatogram are in agreement with the literature data. [10]

Synthesis of (2*S*,3*S*)-4-nitro-2,3-diphenylbutanal (14)

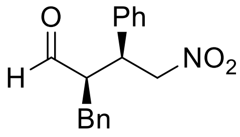

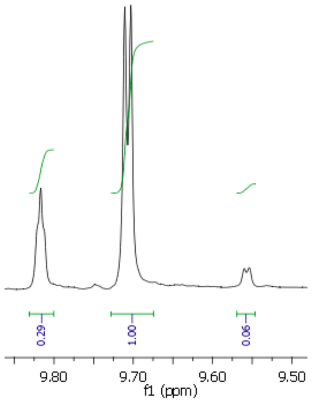

Figure S20: Magnification of the Aldehyde area of ^{1}H NMR Spectrum of compound **14** with 5% of catalyst **I**

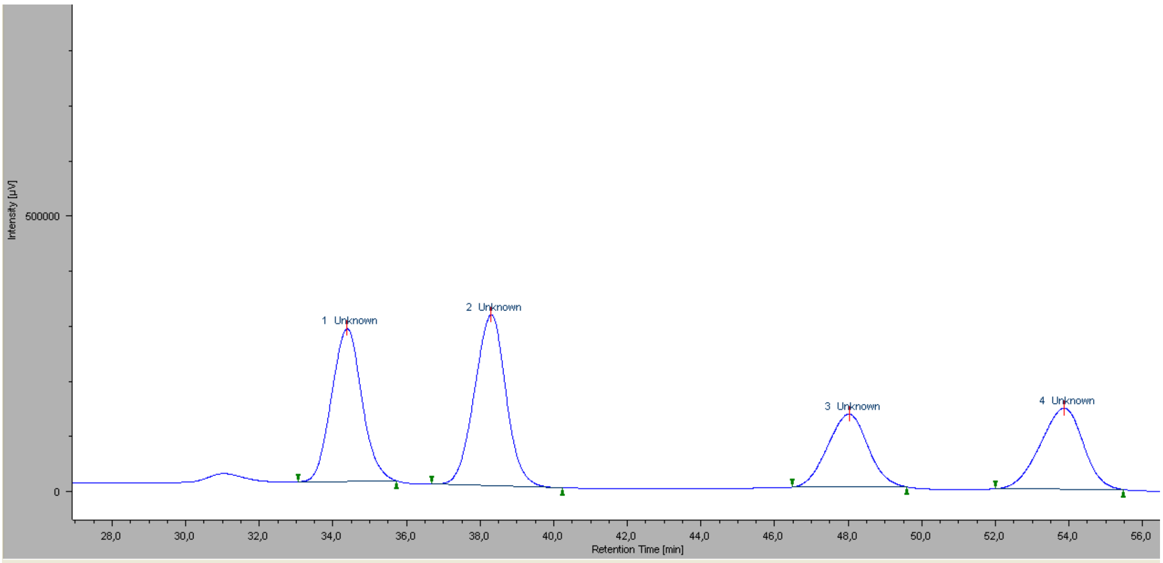

Peak Info. Table

#	Peak Name	tR	Area	Height	Area%	Height%	Peak Start	Peak End	Base Start	Base End	Peak Mark
1	Unknown	34,383	15817547	277009	28,082	31,964	33,050	35,717	33,050	35,717	Manual
2	Unknown	38,292	18520150	310582	32,880	35,838	36,675	40,225	36,675	40,225	Manual
3	Unknown	48,008	10060205	132101	17,861	15,243	46,467	49,567	46,467	49,567	Manual
4	Unknown	53,850	11928586	146940	21,178	16,955	51,975	55,450	51,975	55,450	Manual

Figure S21: Racemic mixture of **14**, evaluated by HPLC.

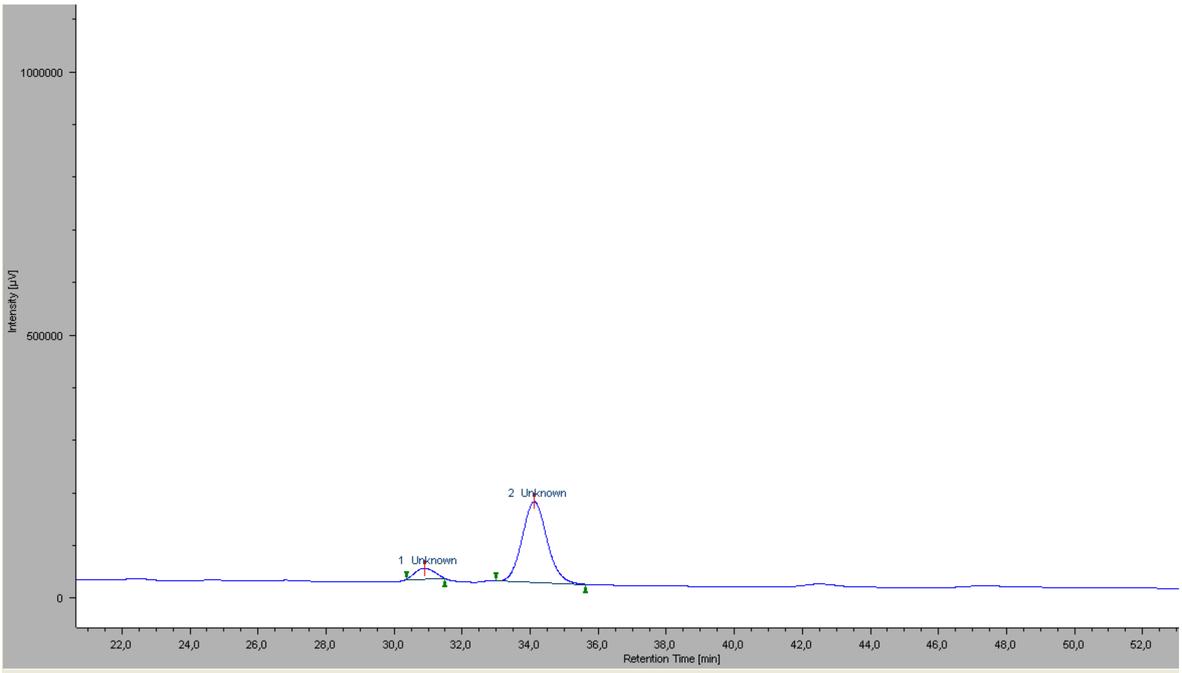

Peak Info. Table

#	Peak Name	tR	Area	Height	Area%	Height%	Peak Start	Peak End	Base Start	Base End	Peak Mark
1	Unknown	30,875	797554	20295	9,334	11,695	30,350	31,492	30,350	31,492	Manual
2	Unknown	34,108	7747336	153236	90,666	88,305	32,975	35,625	32,975	35,625	Manual

Figure S22: Enantiomeric excess of **14**, evaluated by HPLC of crude reaction sample

Loading catalyst = 5 mol %

Conversion =80%; yield = 72%; *d.r.* = 89%, *e.e.* = 82%. The enantiomeric excess was determined by chiral stationary phase HPLC: Chiralcel OD-H, hexane/iPrOH 93:7, 1.0 ml/min, 25 °C, 220 nm, 30.8 min (*syn*, minor), 34.1 min (*syn*, major).

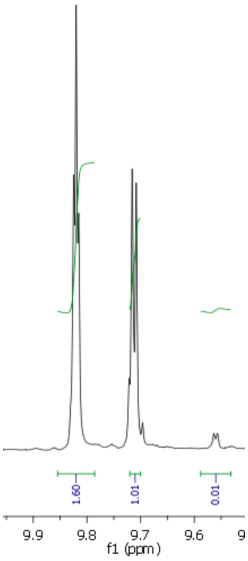

Figure S23:Magnification of the Aldehyde area of ^{1}H NMR Spectrum of compound 14 with 1% of catalyst I

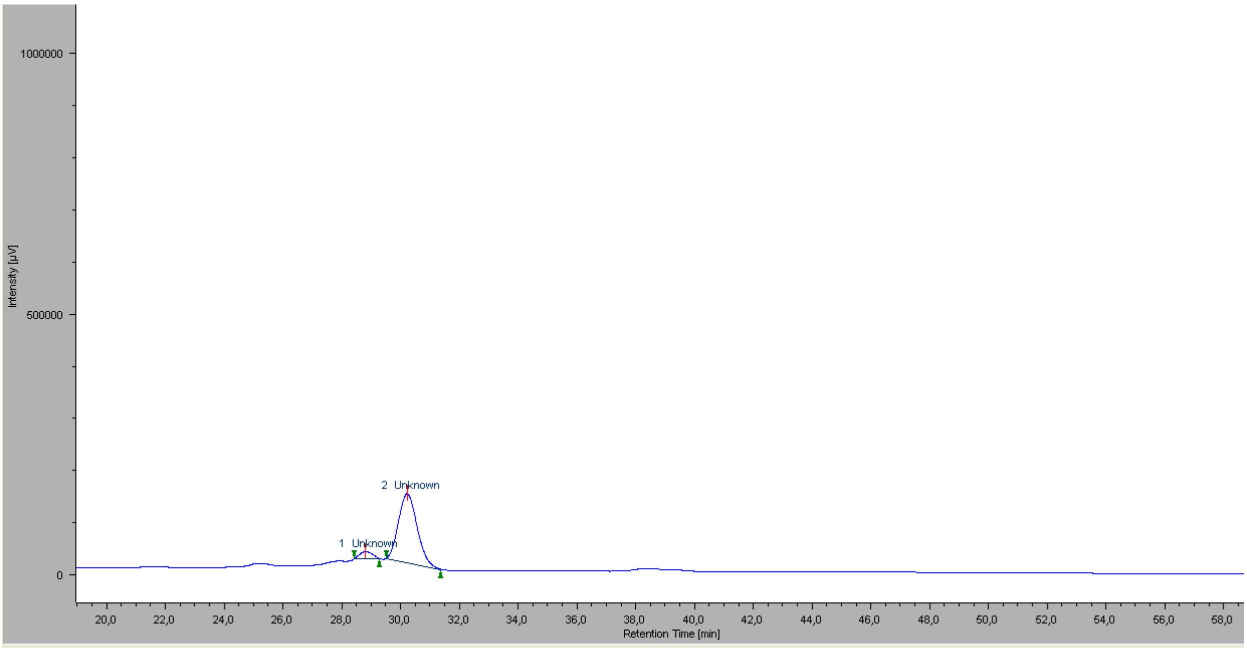

Peak Info. Table

#	Peak Name	tR	Area	Height	Area%	Height%	Peak Start	Peak End	Base Start	Base End	Peak Mark
1	Unknown	28,808	388492	13297	6,259	9,164	28,425	29,258	28,425	29,258	Manual
2	Unknown	30,217	5817951	131807	93,741	90,836	29,500	31,367	29,500	31,367	Manual

Figure S24: Enantiomeric excess of **14**, evaluated by HPLC of crude reaction sample

Loading catalyst = 1 mol %
Conversion = 40%; yield = 38%; d.r. = 99%, e.e. = 88%. The enantiomeric excess was determined by chiral stationary phase HPLC: Chiralcel OD-H, hexane/iPrOH 93:7, 1.0 ml/min, 25 °C, 220 nm, 30.8 min (syn, minor), 34.1 min (syn, major).
The NMR characterization and the HPLC chromatogram are in agreement with the literature data. [10]

Synthesis of (*R*)-1-(2-nitro-1-phenylethyl)cyclopentane-1-carbaldehyde (15)

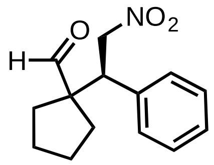

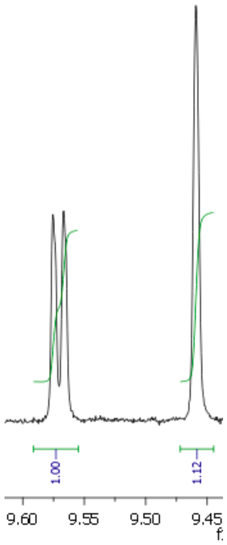

Figure S25: Magnification of the Aldehyde area of ^{1}H NMR Spectrum of compound **15**

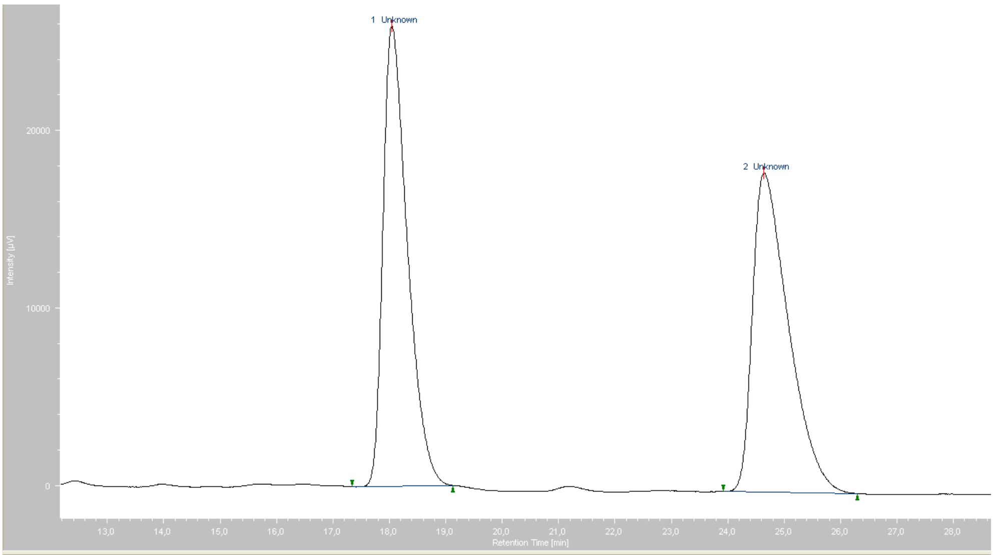

Peak Info. Table

#	Peak Name	tR	Area	Height	Area%	Height%	Peak Start	Peak End	Base Start	Base End	Peak Mark
1	Unknown	18,042	779063	25875	49,483	59,051	17,342	19,117	17,342	19,117	Manual
2	Unknown	24,633	795339	17943	50,517	40,949	23,908	26,292	23,908	26,292	Manual

Figure S26: Racemic mixture of **15**, evaluated by HPLC

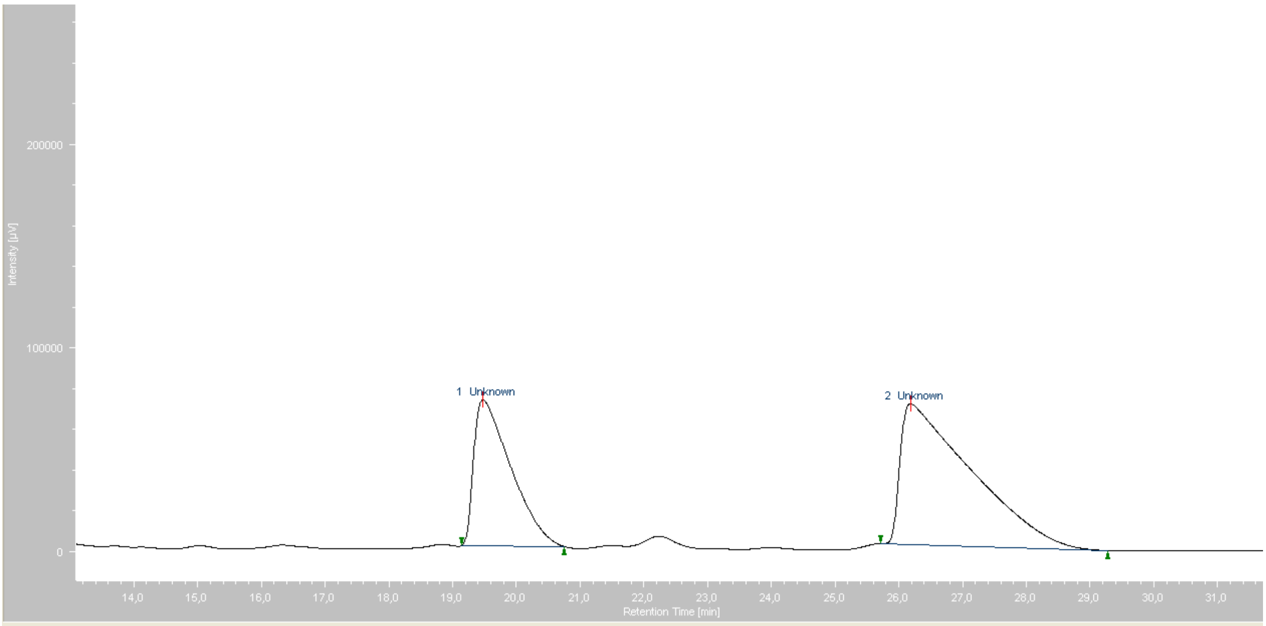

Peak Info. Table

#	Peak Name	tR	Area	Height	Area%	Height%	Peak Start	Peak End	Base Start	Base End	Peak Mark
1	Unknown	19,475	2891625	71546	35,068	50,884	19,133	20,742	19,133	20,742	Manual
2	Unknown	26,175	5354145	69061	64,932	49,116	25,700	29,267	25,700	29,267	Manual

Figure S27: Enantiomeric excess of **15**, evaluated by HPLC of crude reaction sample

Conversion = 53%; yield: 50%; 35% *e.e.*. The enantiomeric excess was determined by chiral stationary phase HPLC: Chiralcel AD, hexane/iPrOH 95:5, 0.8 ml/min, 25 °C, 254 nm, 19.4 min (*syn*, minor), 26.1 min (*syn*, major).
The NMR characterization and the HPLC chromatogram are in agreement with the literature data.[10]

Synthesis of (*2R,3S*)-3-(4-methoxyphenyl)-2-methyl-4-nitrobutanal (16)

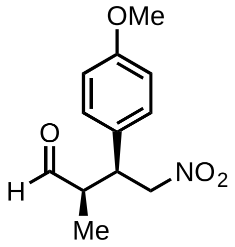

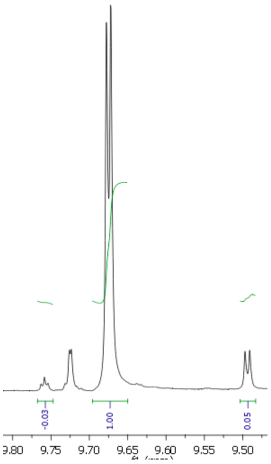

Figure S28: Magnification of the Aldehyde area of ^{1}H NMR Spectrum of compound **16**

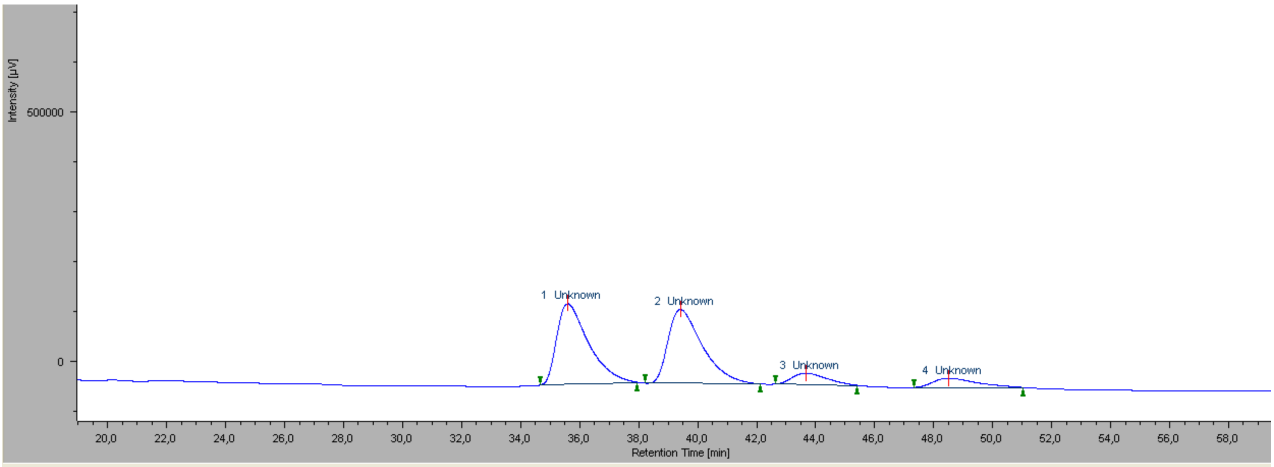

Peak Info. Table

#	Peak Name	tR	Area	Height	Area%	Height%	Peak Start	Peak End	Base Start	Base End	Peak Mark
1	Unknown	35,583	12053435	161264	43,419	45,960	34,658	37,942	34,658	37,942	Manual
2	Unknown	39,400	11988347	148212	43,185	42,240	38,217	42,100	38,217	42,100	Manual
3	Unknown	43,650	1837770	22830	6,620	6,507	42,617	45,383	42,617	45,383	Manual
4	Unknown	48,483	1881037	18571	6,776	5,293	47,325	50,992	47,325	50,992	Manual

Figure S29: Racemic mixture of 16, evaluated by HPLC

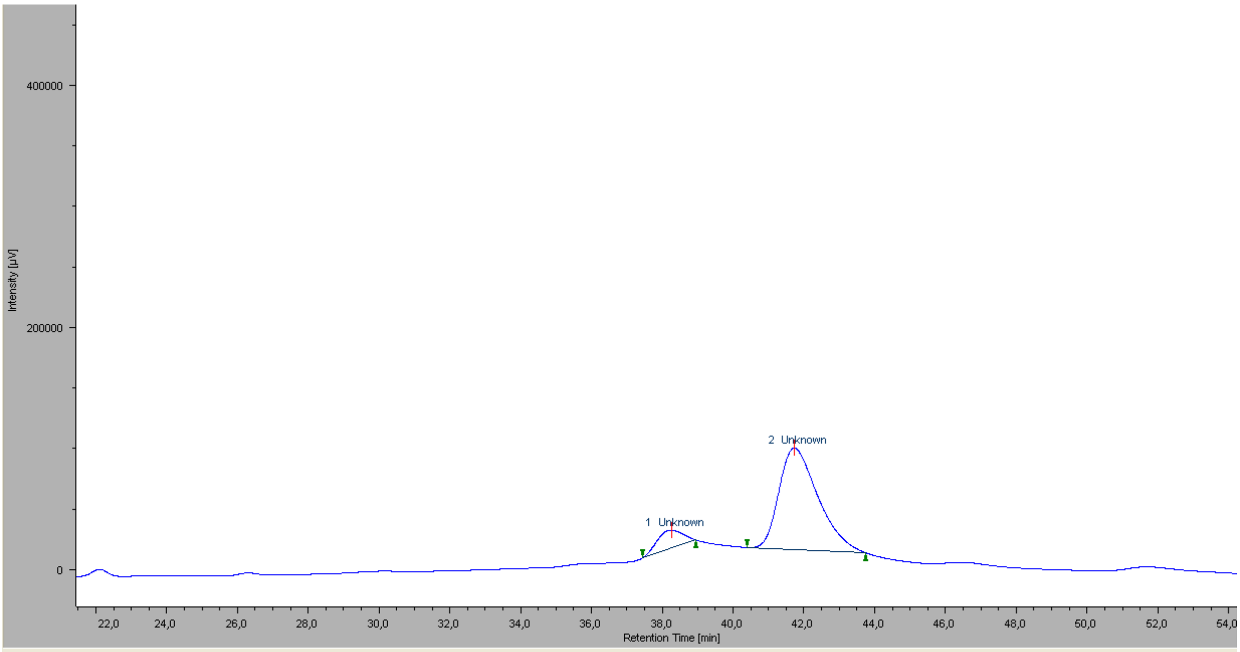

Peak Info. Table

#	Peak Name	tR	Area	Height	Area%	Height%	Peak Start	Peak End	Base Start	Base End	Peak Mark
1	Unknown	38,242	761764	14970	10,620	15,172	37,433	38,933	37,433	38,933	Manual
2	Unknown	41,717	6411429	83703	89,380	84,828	40,383	43,717	40,383	43,717	Manual

Figure S30: Enantiomeric excess of **16**, evaluated by HPLC of crude reaction sample

Conversion= quant%; yield = 87%; *d.r.* = 91%, *e.e.* = 80 %. The enantiomeric excess was determined by chiral stationary phase HPLC: Chiralcel OD-H, hexane/iPrOH 90:1, 1 ml/min, 25 °C, 210 nm, 38.2 min (*syn*, minor), 41.7 min (*syn*, major).

The NMR characterization and the HPLC chromatogram are in agreement with the literature data. [11]

Synthesis of (*R*)-2-((*S*)-1-(4-methoxyphenyl)-2-nitroethyl)pentanal (17)

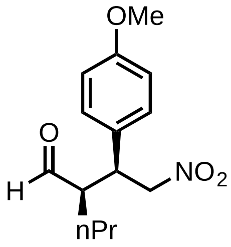

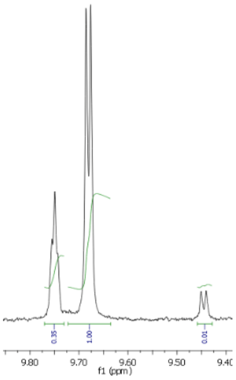

Figure S31: Magnification of the Aldehyde area of ^{1}H NMR Spectrum of compound **17**

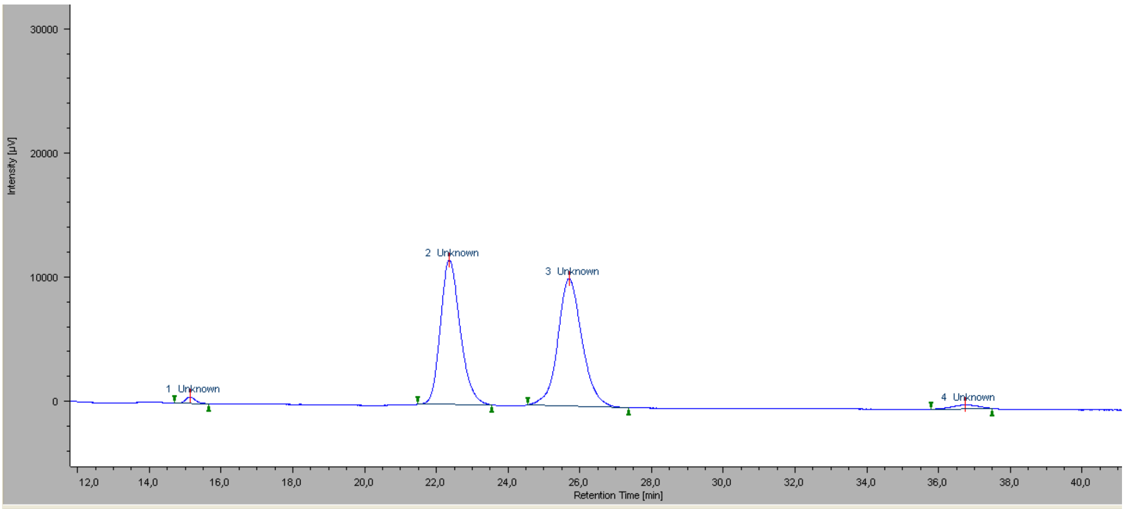

Peak Info. Table

#	Peak Name	tR	Area	Height	Area%	Height%	Peak Start	Peak End	Base Start	Base End	Peak Mark
1	Unknown	15,133	10072	524	1,060	2,306	14,692	15,650	14,692	15,650	Manual
2	Unknown	22,358	444496	11628	46,779	51,192	21,475	23,533	21,475	23,533	Manual
3	Unknown	25,692	480491	10255	50,567	45,147	24,533	27,350	24,533	27,350	Manual
4	Unknown	36,708	15144	308	1,594	1,355	35,758	37,475	35,758	37,475	Manual

Figure S32: Racemic mixture of **17**, evaluated by HPLC

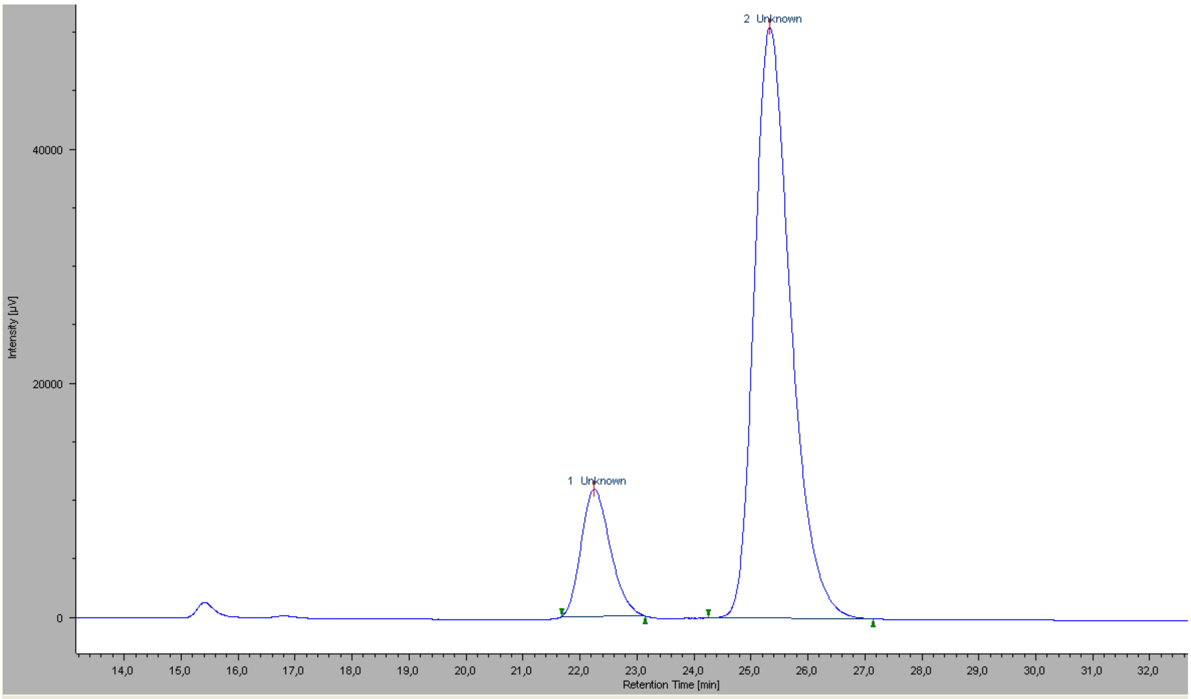

Peak Info. Table

#	Peak Name	tR	Area	Height	Area%	Height%	Peak Start	Peak End	Base Start	Base End	Peak Mark
1	Unknown	22,242	385927	10847	14,667	17,708	21,667	23,133	21,667	23,133	Manual
2	Unknown	25,326	2245343	50409	85,333	82,292	24,242	27,125	24,242	27,125	Manual

Figure S33: Enantiomeric excess of **17** evaluated by HPLC of crude reaction sample

Conversion =74%; yield = 70%; *d.r.* = 98%, *e.e.* = 70%. The enantiomeric excess was determined by chiral stationary phase HPLC: Chiralcel OD-H, hexane/iPrOH 9:1, 1.0 ml/min, 25 °C, 254 nm, 22.2 min (*syn*, minor), 25.3 min (*syn*, major).

The NMR characterization and the HPLC chromatogram are in agreement with the literature data. [12]

Synthesis of (*2R,3R*)-2-methyl-4-nitro-3-(thiophen-2-yl)butanal (18)

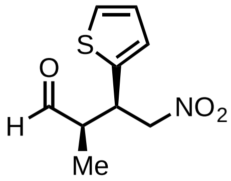

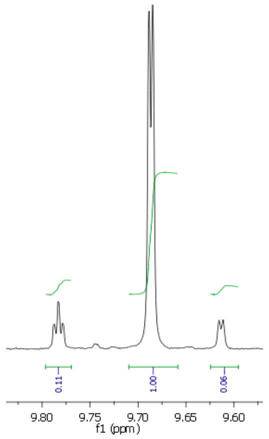

Figure S34: Magnification of the Aldehyde area of ^{1}H NMR Spectrum of compound **18**

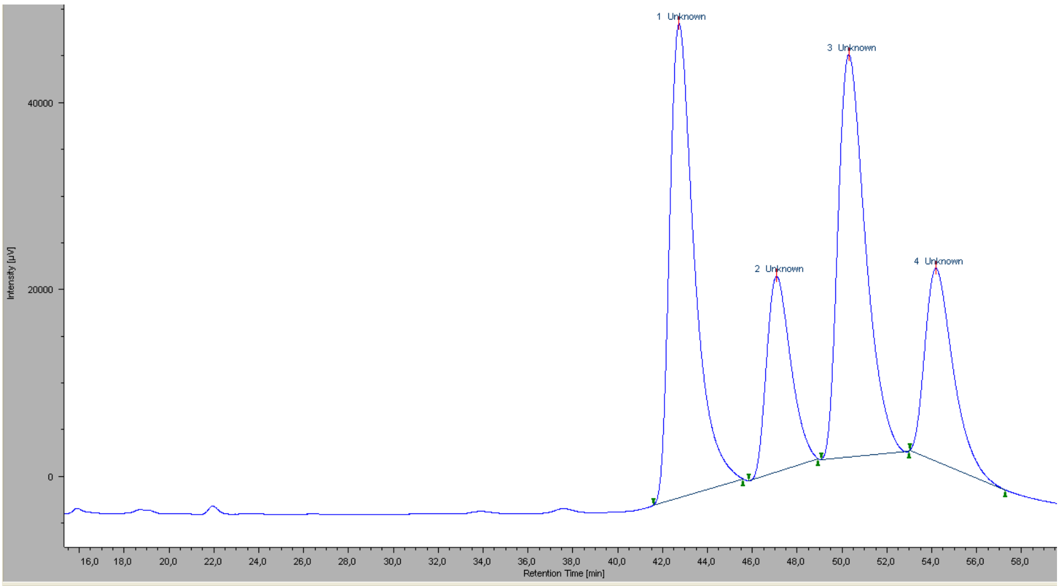

Peak Info. Table

#	Peak Name	tR	Area	Height	Area%	Height%	Peak Start	Peak End	Base Start	Base End	Peak Mark
1	Unknown	42,708	3914990	50678	35,732	37,469	41,567	45,558	41,567	45,558	Manual
2	Unknown	47,058	1564017	20917	14,275	15,466	45,808	48,892	45,808	48,892	Manual
3	Unknown	50,275	3671216	43018	33,507	31,806	49,042	52,933	49,042	52,933	Manual
4	Unknown	54,167	1806332	20638	16,486	15,259	52,983	57,225	52,983	57,225	Manual

Figure S35: Racemic mixture of **18**, evaluated by HPLC

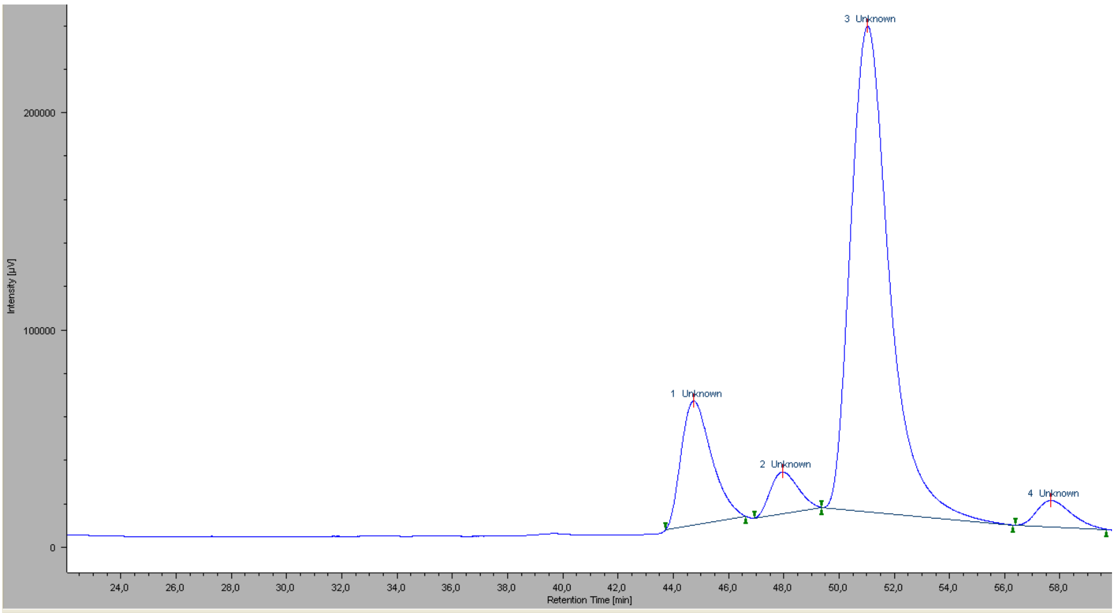

Peak Info. Table

#	Peak Name	tR	Area	Height	Area%	Height%	Peak Start	Peak End	Base Start	Base End	Peak Mark
1	Unknown	44,717	4367591	57078	15,313	18,336	43,700	46,600	43,700	46,600	Manual
2	Unknown	47,942	1323256	19088	4,639	6,132	46,917	49,342	46,917	49,342	Manual
3	Unknown	51,017	21791148	223077	76,399	71,662	49,350	56,258	49,350	56,258	Manual
4	Unknown	57,633	1040949	12048	3,650	3,870	56,367	59,650	56,367	59,650	Manual

Figure S36: Enantiomeric excess of **18** evaluated by HPLC of crude reaction sample

Conversion = quantitative; yield = 82%; *d.r.* = 89%*; e.e.* = 67%. The enantiomeric excess was determined by chiral stationary phase HPLC: Chiralcel OD-H, *n*hexane/*i*PrOH 95 : 5, 1.0 mL/min, 25 °C, 254 nm, 44.7 min (*syn*, minor), 51.0 min (*syn*, major).
The NMR characterization and the HPLC chromatogram are in agreement with the literature data. [13]

Synthesis of (*2R,3S*)-3-(4-chlorophenyl)-2-methyl-4-nitrobutanal (19)

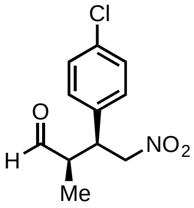

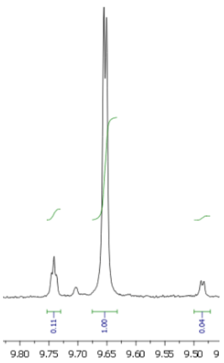

Figure S37: Magnification of the Aldehyde area of ^{1}H NMR Spectrum of compound **19**

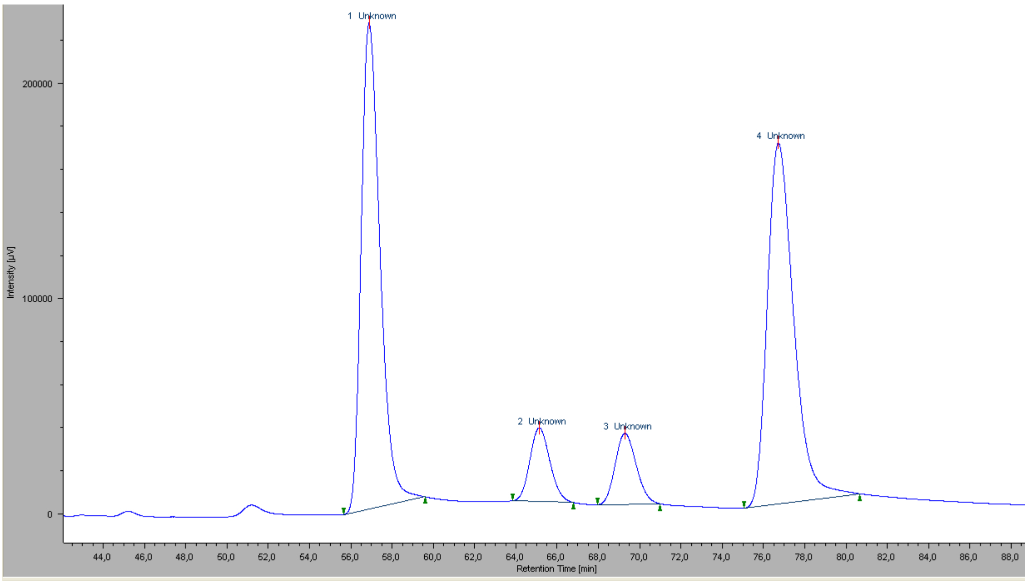

Peak Info. Table

#	Peak Name	tR	Area	Height	Area%	Height%	Peak Start	Peak End	Base Start	Base End	Peak Mark
1	Unknown	56,875	14323736	225452	42,314	49,010	55,642	59,583	55,642	59,583	Manual
2	Unknown	65,108	2309750	34220	6,823	7,439	63,825	66,783	63,825	66,783	Manual
3	Unknown	69,258	2339617	32954	6,912	7,164	67,917	70,950	67,917	70,950	Manual
4	Unknown	76,692	14877653	167385	43,951	36,387	75,042	80,650	75,042	80,650	Manual

Figure S38: Racemic mixture of **19**, evaluated by HPLC

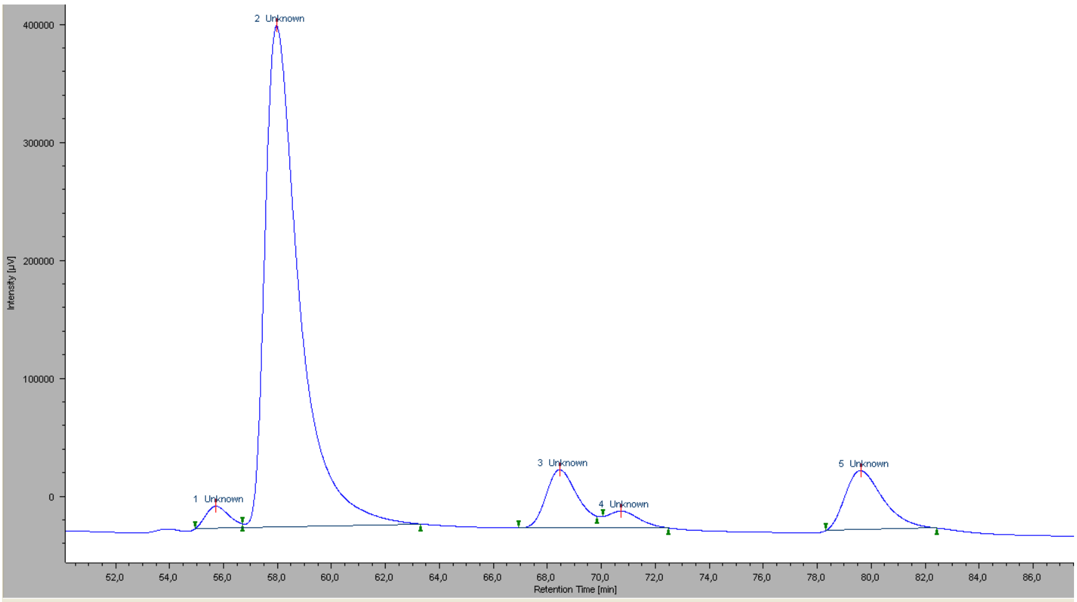

Peak Info. Table

#	Peak Name	tR	Area	Height	Area%	Height%	Peak Start	Peak End	Base Start	Base End	Peak Mark
1	Unknown	55,708	1104254	18542	2,267	3,333	54,958	56,700	54,958	63,292	Manual
2	Unknown	57,958	37570714	424147	77,123	76,250	56,700	63,292	54,958	63,292	Manual
3	Unknown	68,450	4003186	49281	8,218	8,859	66,933	69,817	66,933	72,467	Manual
4	Unknown	70,708	1152602	14245	2,366	2,561	70,042	72,467	66,933	72,467	Manual
5	Unknown	79,583	4884408	50044	10,026	8,997	78,300	82,392	78,300	82,392	Manual

Figure S39: Enantiomeric excess of **19** evaluated by HPLC of crude reaction sample

Conversion= 90%; yield = 87%; *d.r.* = 93%, *e.e.* = 77 %. The enantiomeric excess was determined by chiral stationary phase HPLC: Chiralcel AD-H, hexane/iPrOH 98:2, 0.5 ml/min, 25 °C, 220 nm, 57.9 min (syn, major), 79.5 min (syn, minor).
The NMR characterization and the HPLC chromatogram are in agreement with the literature data. [14]

Synthesis of (*R*)-2-((*S*)-1-(4-chlorophenyl)-2-nitroethyl)pentanal (20)

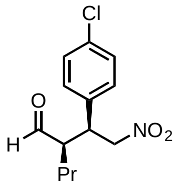

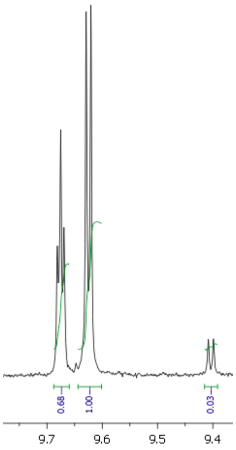

Figure S40: Magnification of the Aldehyde area of ^{1}H NMR Spectrum of compound 20

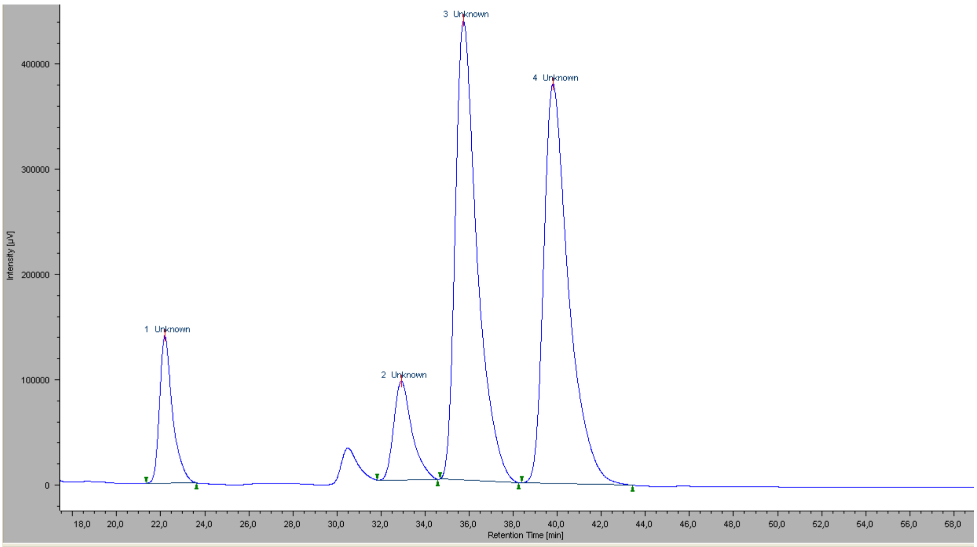

Peak Info. Table

#	Peak Name	tR	Area	Height	Area%	Height%	Peak Start	Peak End	Base Start	Base End	Peak Mark
1	Unknown	22,183	5709163	139729	8,046	13,324	21,342	23,617	21,342	23,617	Manual
2	Unknown	32,908	5375239	93576	7,575	8,923	31,817	34,558	31,817	34,558	Manual
3	Unknown	35,733	30029098	435967	42,318	41,571	34,667	38,233	34,667	38,233	Manual
4	Unknown	39,792	29846294	379468	42,061	36,183	38,375	43,400	38,375	43,400	Manual

Figure S41: Racemic mixture of **20**, evaluated by HPLC

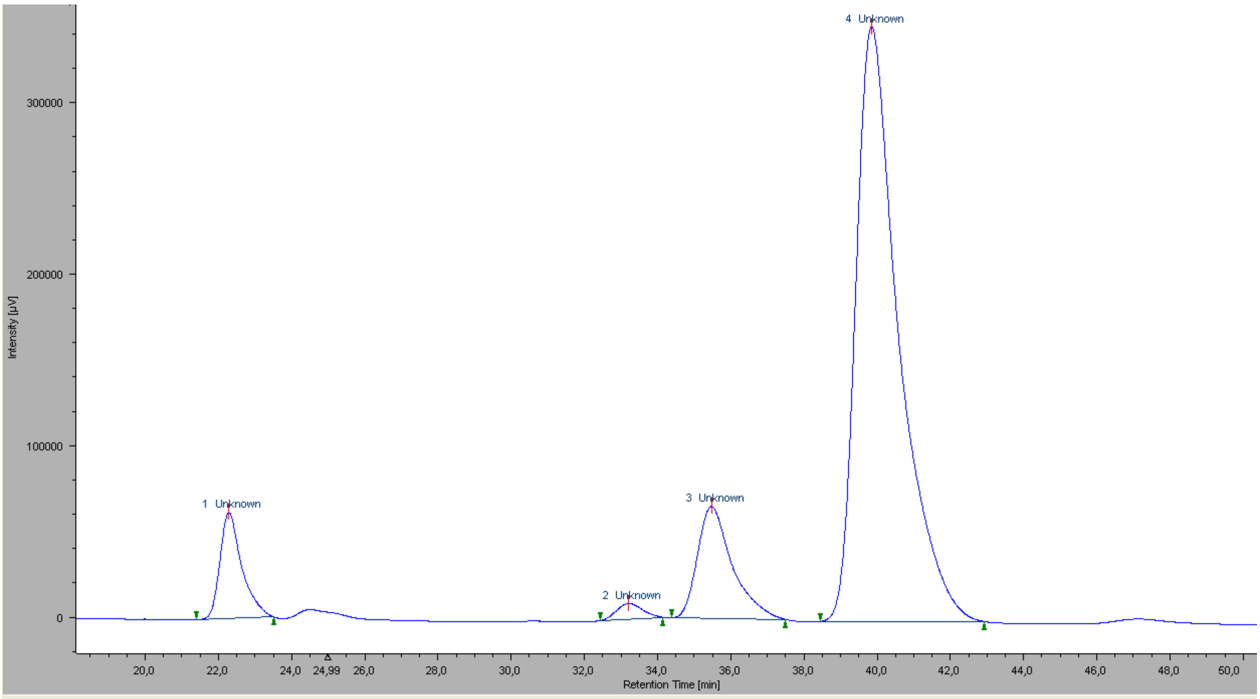

Peak Info. Table

#	Peak Name	tR	Area	Height	Area%	Height%	Peak Start	Peak End	Base Start	Base End	Peak Mark
1	Unknown	22,283	2559049	61631	7,222	12,786	21,383	23,508	21,383	23,508	Manual
2	Unknown	33,200	446059	9072	1,259	1,882	32,417	34,133	32,417	34,133	Manual
3	Unknown	35,458	4300367	65169	12,135	13,519	34,358	37,475	34,358	37,475	Manual
4	Unknown	39,833	28131032	346168	79,384	71,813	38,425	42,900	38,425	42,900	Manual

Figure S42: Enantiomeric excess of **20** evaluated by HPLC of crude reaction sample

Conversion = 60%; yield = 55; *d.r.* = 94%, *e.e.* = 74 %. The enantiomeric excess was determined by chiral stationary phase HPLC: Chiralcel IC, hexane/iPrOH 90:10, 1 ml/min, 25 °C, 220 nm, 35.4 min (syn, minor), 39.8 min (syn, major).

The NMR characterization and the HPLC chromatogram are in agreement with the literature data. [10]

Synthesis of (2*R*,3*R*)-3-cyclohexyl-2-methyl-4-nitrobutanal (21)

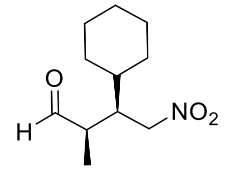

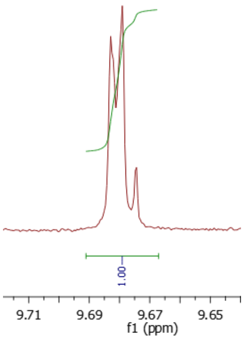

Figure S43: Magnification of the Aldehyde area of ^{1}H NMR Spectrum of (2R,3R)-3-cyclohexyl-2-methyl-4-nitrobutanal synthesized by using 1% of Catalyst I at -10 °C

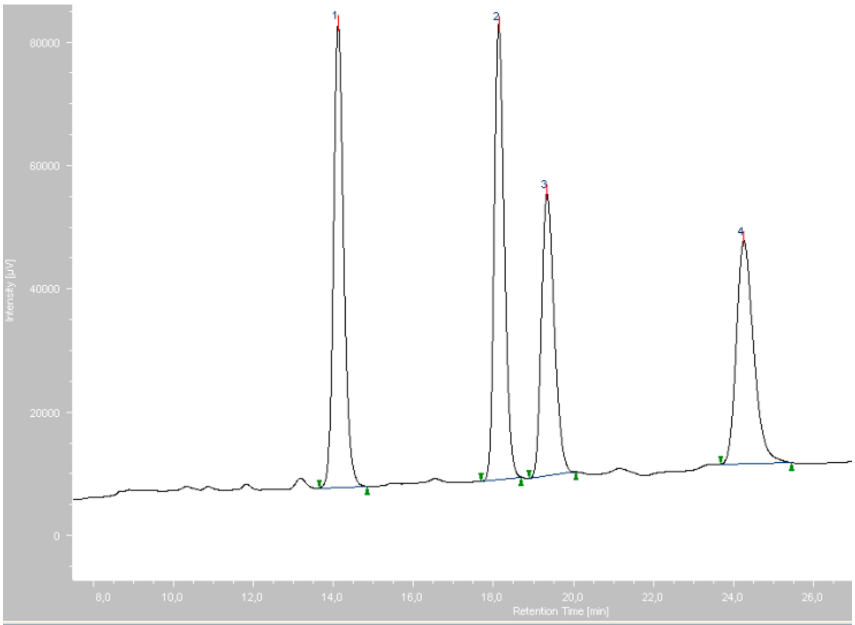

Peak Info. Table: RBA222

#	Peak Name	CH	tR	Area	Height	Area%	Height%	Quantity	NTP	Resolution	Symmetry Factor	Warning
1	Unknown	1	14,108	1323381	74999	28,150	32,491	N/A	15544	8,832	1,202	
2	Unknown	1	18,117	1286723	73697	27,371	31,927	N/A	25089	2,322	1,233	
3	Unknown	1	19,325	1018102	45864	21,657	19,869	N/A	17396	7,344	1,280	
4	Unknown	1	24,250	1072890	36269	22,822	15,712	N/A	16348	N/A	1,270	

Figure S44: Racemic mixture of (2*R*,3*R*)-3-cyclohexyl-2-methyl-4-nitrobutanal, evaluated by HPLC

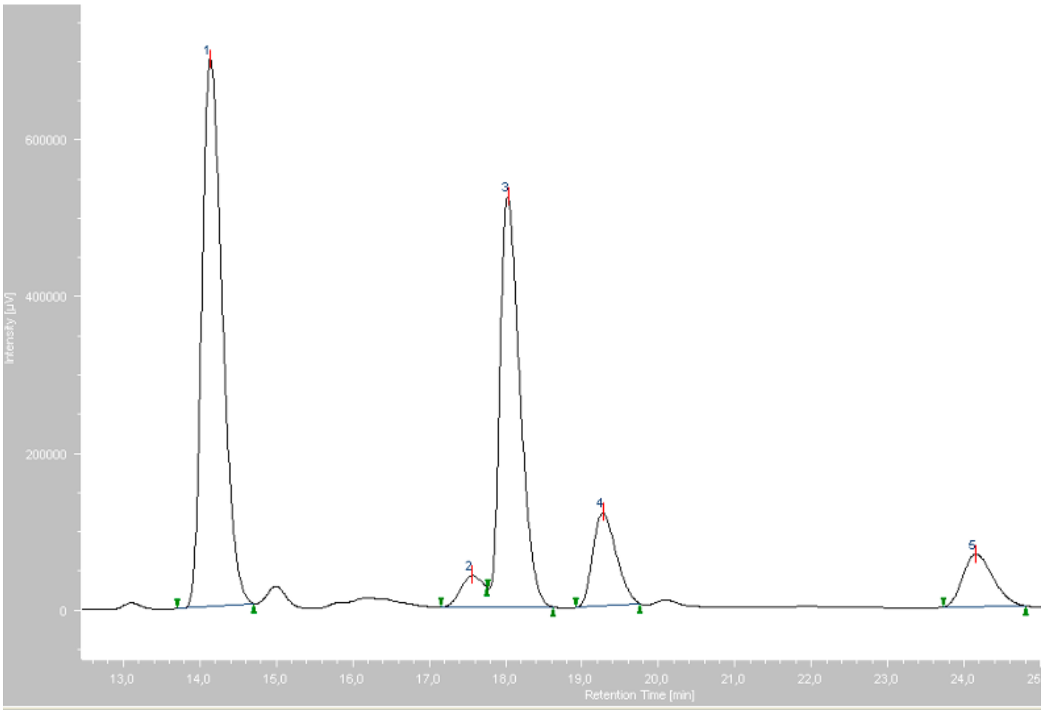

Peak Info. Table: RB226B_10_bis

#	Peak Name	CH	tR	Area	Height	Area%	Height%	Quantity	NTP	Resolution	Symmetry Factor	Warning
1	Unknown	1	14,142	12963006	697854	46,862	48,253	N/A	13278	N/A	1,313	
2	Unknown	1	17,558	796650	40269	2,880	2,784	N/A	N/A	N/A	N/A	
3	Unknown	1	18,025	9544660	522650	34,504	36,139	N/A	22491	2,364	1,382	
4	Unknown	1	19,267	2526080	118666	9,132	8,205	N/A	18122	7,482	1,264	
5	Unknown	1	24,142	1831830	66794	6,622	4,618	N/A	17303	N/A	1,255	

Figure S45: Enantiomeric excess of (2*R*,3*R*)-3-cyclohexyl-2-methyl-4-nitrobutanal evaluated by HPLC of crude reaction sample

Conversion = 60%; yield = 45; *d.r.* = 68%, *e.e.* = 15%. The enantiomeric and diasteroisomeric excess was determined by chiral stationary phase HPLC: Chiralcel ADH, hexane/iPrOH 99:1, 1 ml/min, 25 °C, 220 nm, 14.1 min (syn, major), 18.0 min (syn, minor).

The NMR characterization and the HPLC chromatogram are in agreement with the literature data.[14]

Synthesis of (2*R*,3*R*)-2-methyl-3-(nitromethyl)heptanal (22)

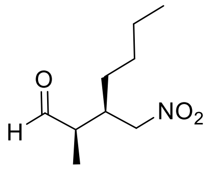

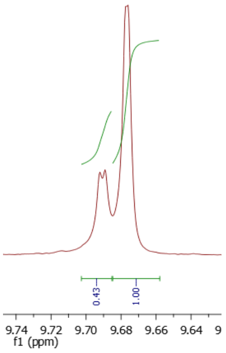

Figure S46: Magnification of the Aldehyde area of 1H NMR Spectrum of (2*R*,3*R*)-2-methyl-3-(nitromethyl)heptanal synthesized by using 1% of Catalyst **I** at -10 °C

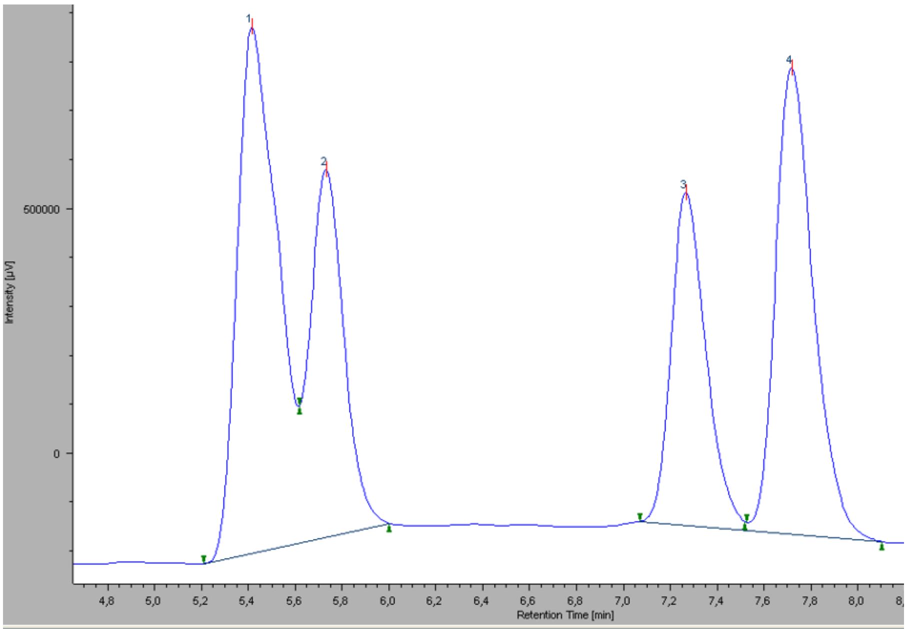

Peak Info. Table: RB227B_rac_210

#	Peak Name	CH	tR	Area	Height	Area%	Height%	Quantity	NTP	Resolution	Symmetry Factor	Warning
1	Unknown	1	5,417	12753800	1073834	33,585	31,102	N/A	4069	1,032	N/A	
2	Unknown	1	5,733	7552525	748737	19,888	21,686	N/A	6913	5,667	N/A	
3	Unknown	1	7,267	6982724	678265	18,388	19,645	N/A	11872	1,620	1,277	
4	Unknown	1	7,717	10685779	951767	28,139	27,567	N/A	11303	N/A	1,293	

Figure S47: Racemic mixture of (2*R*,3*R*)-2-methyl-3-(nitromethyl)heptanal, evaluated by HPLC

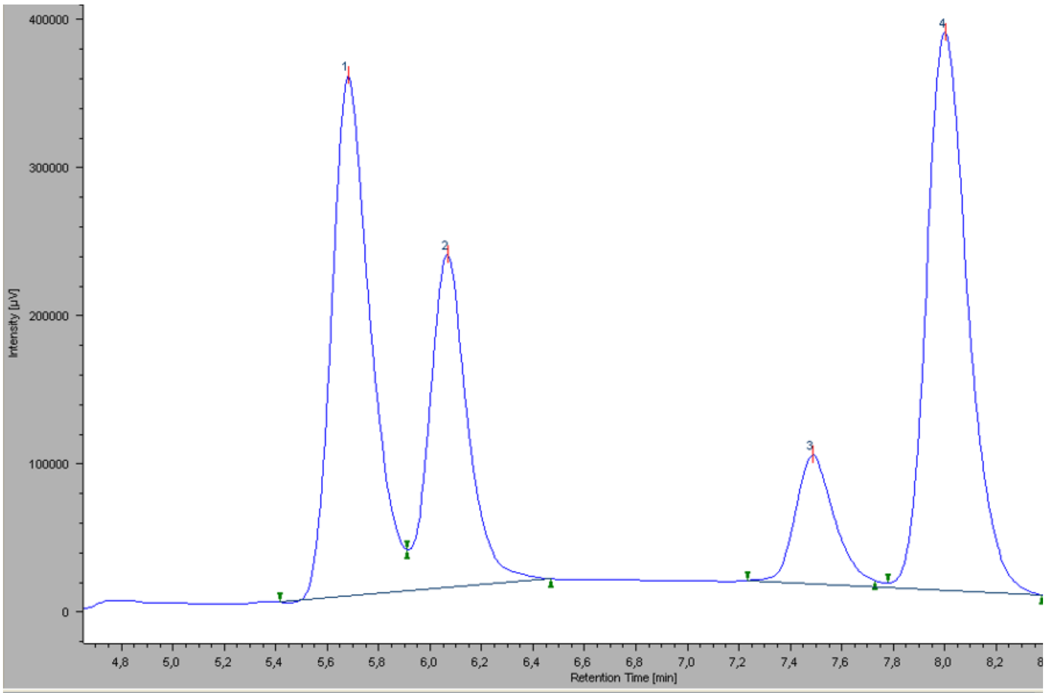

Peak Info. Table: RB229_10

#	Peak Name	CH	tR	Area	Height	Area%	Height%	Quantity	NTP	Resolution	Symmetry Factor	Warning
1	Unknown	1	5,683	3668997	350669	32,998	33,789	N/A	7219	1,469	N/A	
2	Unknown	1	6,067	2311671	224572	20,790	21,638	N/A	8994	5,402	N/A	
3	Unknown	1	7,483	913895	86762	8,219	8,360	N/A	12295	1,844	1,307	
4	Unknown	1	8,000	4224335	375831	37,992	36,213	N/A	12024	N/A	1,231	

Figure S48: Enantiomeric excess of (2R,3R)-2-methyl-3-(nitromethyl)heptanal evaluated by HPLC of crude reaction sample

Conversion = 50%; yield = 40; *d.r.* = 42%, *e.e.* = rac. The enantiomeric and diasteroisomeric excess was determined by chiral stationary phase HPLC: Chiralcel ODH, hexane/EtOH 110:5, 1 ml/min, 25 °C, 220 nm, 8 min (syn, major), 5.6 min (syn, minor).

The NMR characterization and the HPLC chromatogram are in agreement with the literature data.[14]

References for the synthesis and Spectroscopical analysis of known compounds

[1] S.B. Jadhav, R. Chegondi, Diastereoselective Desymmetrization of p-Quinamines through Regioselective Ring Opening of Epoxides and Aziridines, Org. Lett. 21 (2019) 10115–10119. https://doi.org/10.1021/acs.orglett.9b04110.

[2] S. Samadi, A. Ashouri, M. Samadi, Synthesis of Chiral Allylic Esters by Using the New Recyclable Chiral Heterogeneous Oxazoline-Based Catalysts, ACS Omega. 5 (2020) 22367–22378. https://doi.org/10.1021/acsomega.0c02764.

[3] P. Mendonça Matos, W. Lewis, S.P. Argent, J.C. Moore, R.A. Stockman, General Method for the Asymmetric Synthesis of N–H Sulfoximines via C–S Bond Formation, Org. Lett. 22 (2020) 2776–2780. https://doi.org/10.1021/acs.orglett.0c00761.

[4] C.N. Slattery, S. O'Keeffe, A.R. Maguire, Electronic effects of aryl-substituted bis(oxazoline) ligands on the outcome of asymmetric copper-catalysed C–H insertion and aromatic addition reactions, Tetrahedron: Asymmetry. 24 (2013) 1265–1275. https://doi.org/https://doi.org/10.1016/j.tetasy.2013.09.009.

[5] D.E. Karakaş, F. Durap, M. Aydemir, A. Baysal, Synthesis, characterization and first application of chiral C2-symmetric bis(phosphinite)–Pd(II) complexes as catalysts in asymmetric intermolecular Heck reactions, Appl. Organomet. Chem. 30 (2016) 193–198. https://doi.org/https://doi.org/10.1002/aoc.3416.

[6] M.B. Plewe, L.R. Whitby, S. Naik, E.R. Brown, N. V Sokolova, V.R. Gantla, J. York, J.H. Nunberg, L. Zhang, B. Kalveram, A.N. Freiberg, D.L. Boger, G. Henkel, K. McCormack, SAR studies of 4-acyl-1,6-dialkylpiperazin-2-one arenavirus cell entry inhibitors, Bioorg. Med. Chem. Lett. 29 (2019) 126620. https://doi.org/https://doi.org/10.1016/j.bmcl.2019.08.024.

[7] Y. Zhang, L. Huang, X. Li, L. Wang, H. Feng, Chemo- and Diastereoselective Synthesis of N-Propargyl Oxazolidines through a Copper-Catalyzed Domino A3 Reaction, J. Org. Chem. 84 (2019) 5046–5055. https://doi.org/10.1021/acs.joc.8b03244.

[8] M. Breuning, M. Winnacker, M. Steiner, Efficient One-Pot Synthesis of Enantiomerically Pure 2-(Hydroxymethyl)morpholines, European J. Org. Chem. 2007 (2007) 2100–2106. https://doi.org/https://doi.org/10.1002/ejoc.200601006.

[9] J.S. Möhler, T. Schnitzer, H. Wennemers, Amine Catalysis with Substrates Bearing N-Heterocyclic Moieties Enabled by Control over the Enamine Pyramidalization Direction, Chem. – A Eur. J. 26 (2020) 15623–15628. https://doi.org/https://doi.org/10.1002/chem.202002966.

[10] A. Castán, R. Badorrey, J.A. Gálvez, P. López-Ram-de-Víu, M.D. Díaz-de-Villegas, Michael addition of carbonyl compounds to nitroolefins under the catalysis of new pyrrolidine-based bifunctional organocatalysts, Org. Biomol. Chem. 16 (2018) 924–935. https://doi.org/10.1039/C7OB02798B.

[11] Z. Zhao, D. Feng, G. Xie, X. Ma, Functionalized hollow double-shelled polymeric nano-bowls as effective heterogeneous organocatalysts for enhanced catalytic activity in asymmetric Michael addition, J. Catal. 359 (2018) 36–45. https://doi.org/https://doi.org/10.1016/j.jcat.2017.12.019.

[12] C.M. Darapaneni, P. Ghosh, T. Ghosh, G. Maayan, Unique β-Turn Peptoid Structures and Their Application as Asymmetric Catalysts, Chem. – A Eur. J. 26 (2020) 9573–9579. https://doi.org/https://doi.org/10.1002/chem.202000595.

[13] J. Weng, H.-B. Ai, Luo Ren-Shi, G. Lu, Asymmetric Michael reaction of aldehydes with β-nitroalkenes catalyzed by pyrrolidine–camphor derived organocatalysts bearing hydrogen-bond donors, Chirality. 24 (2012) 271–275. https://doi.org/https://doi.org/10.1002/chir.21991.

[14] R.-S. Luo, J. Weng, H.-B. Ai, G. Lu, A.S.C. Chan, Highly Efficient Asymmetric Michael Reaction of Aldehydes to Nitroalkenes with Diphenylperhydroindolinol Silyl Ethers as Organocatalysts, Adv. Synth. Catal. 351 (2009) 2449–2459. https://doi.org/https://doi.org/10.1002/adsc.200900355.

^{1}H and ^{13}C NMR of new compounds

Compound 3d

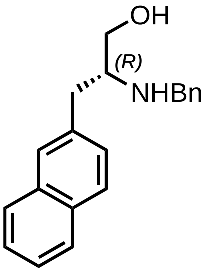

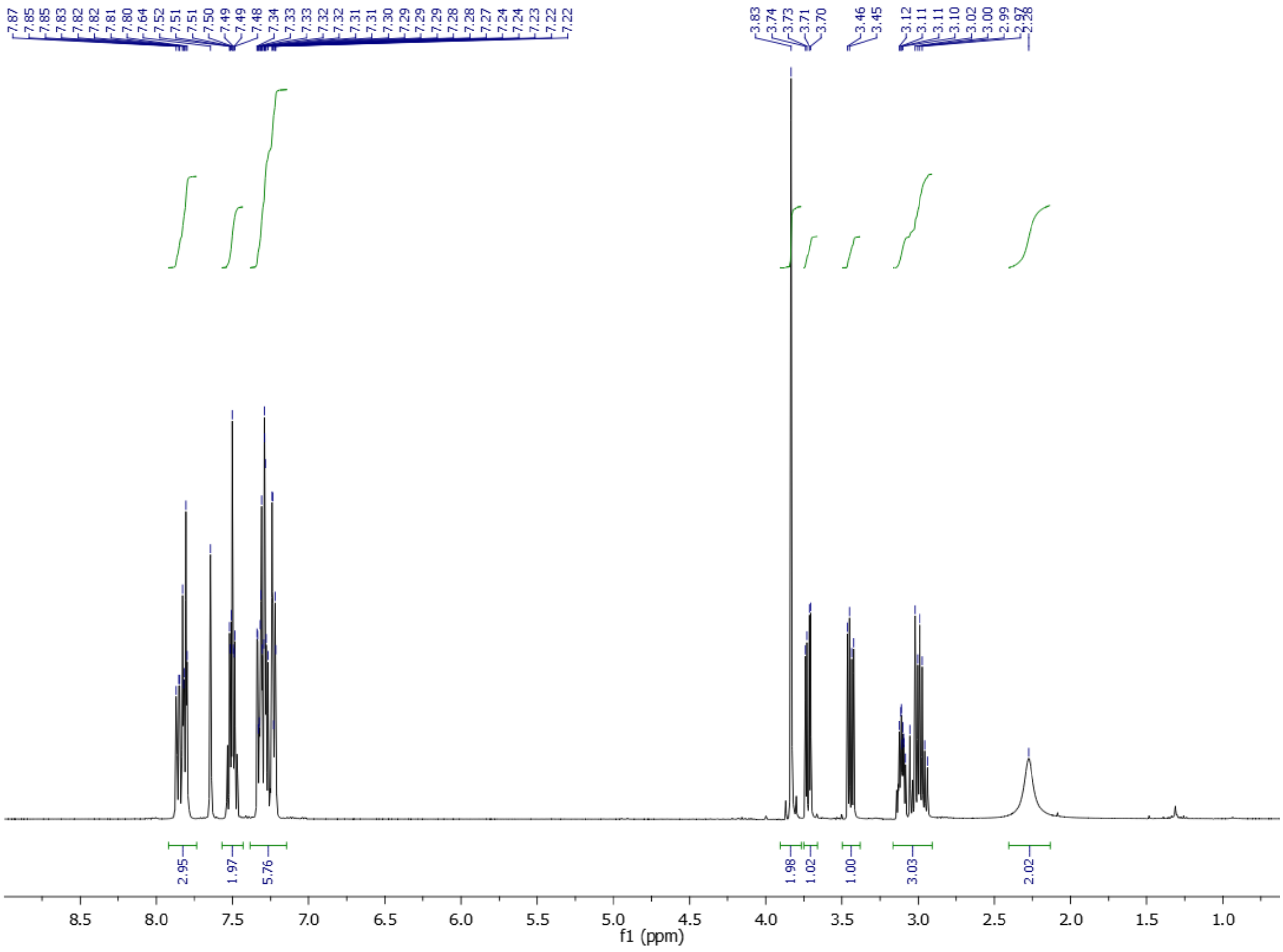

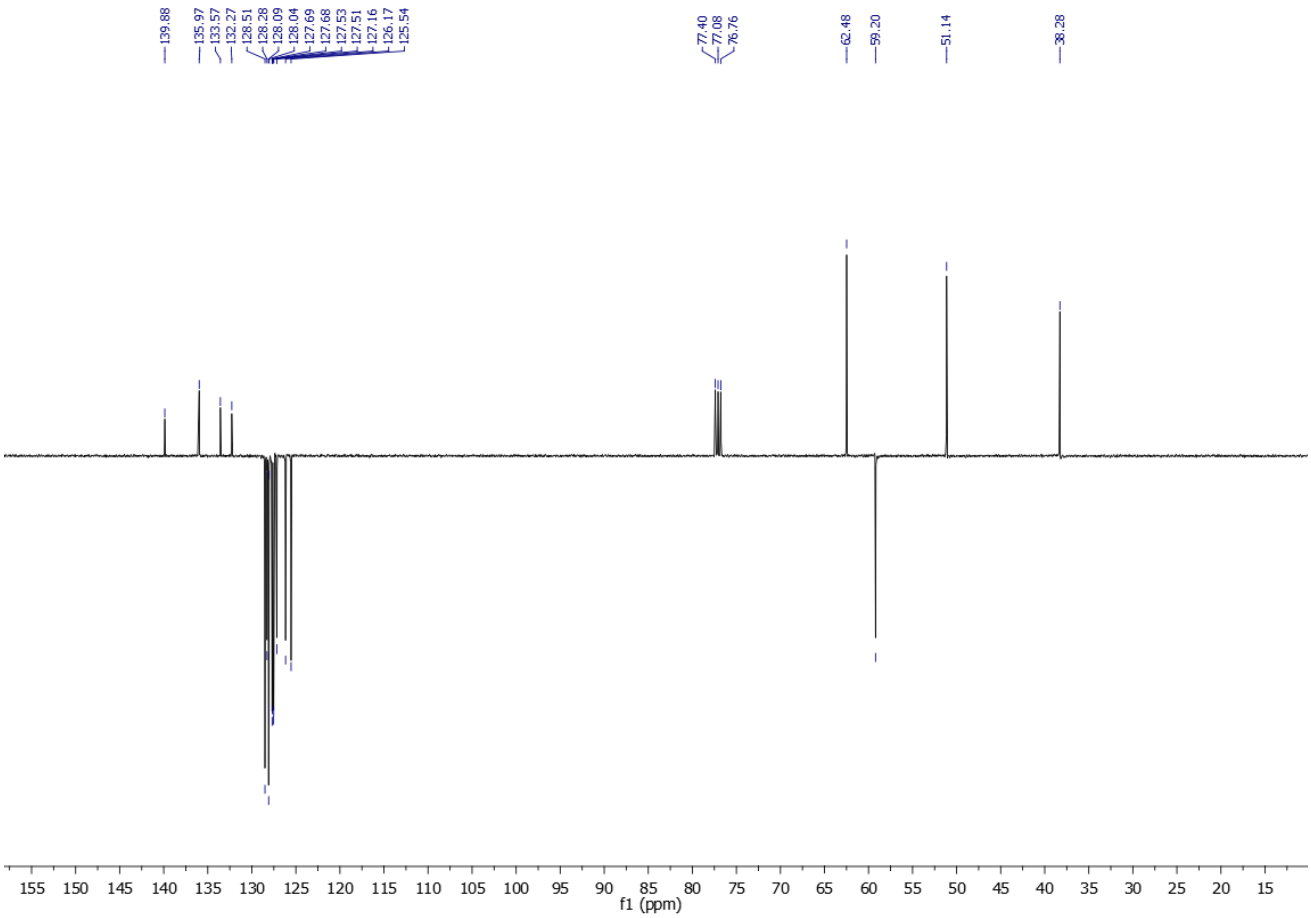
139.88
135.97
133.57
132.27
128.51
128.28
128.09
128.04
127.69
127.68
127.53
127.51
127.16
126.17
125.54
77.40
77.08
76.76
62.48
59.20
51.14
38.28
155 150 145 140 135 130 125 120 115 110 105 100 95 90 85 80 75 70 65 60 55 50 45 40 35 30 25 20 15
f1 (ppm)

Compound 4a

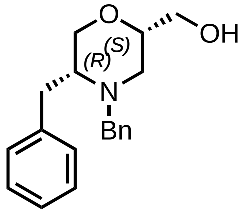

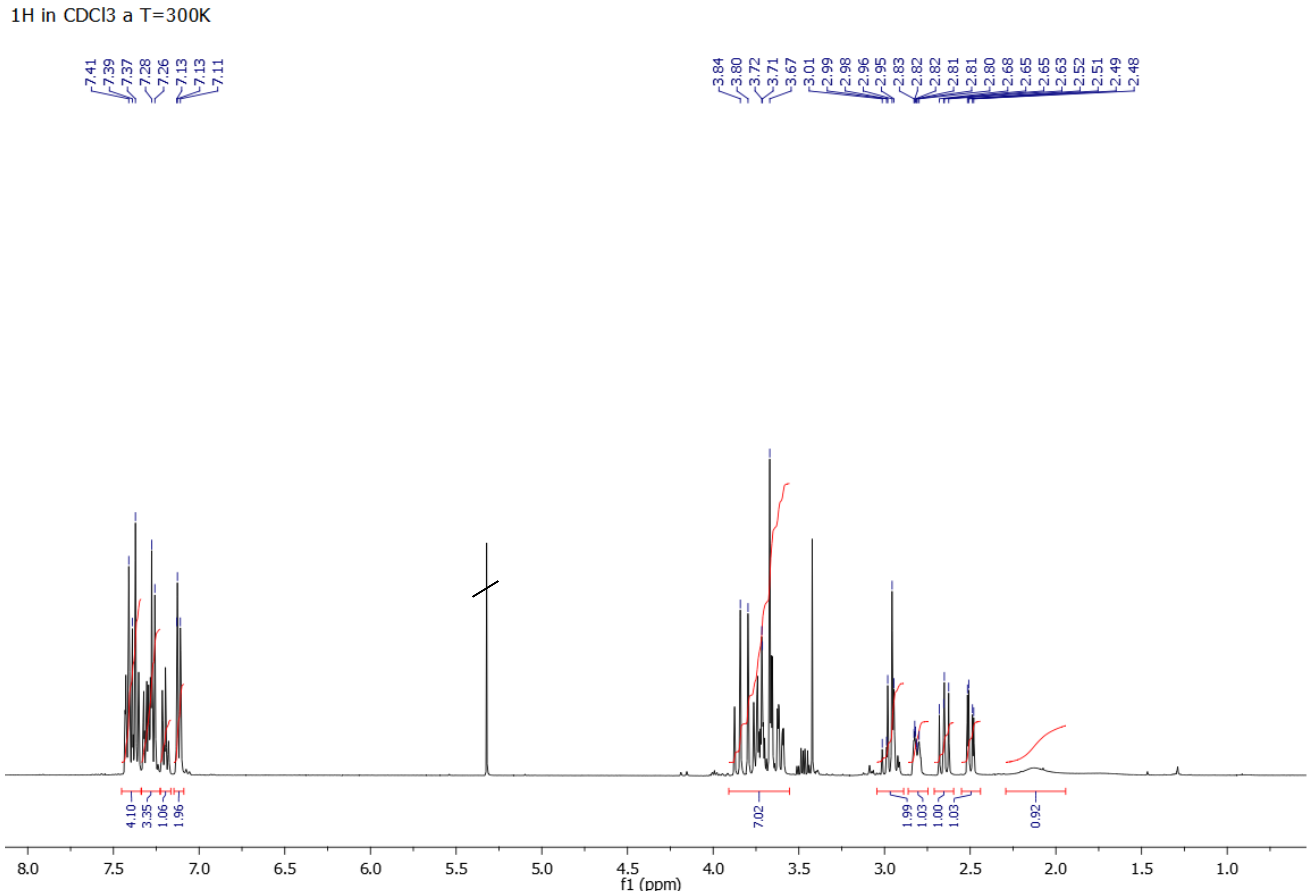

13C-apt in CDCl3 a T=300K

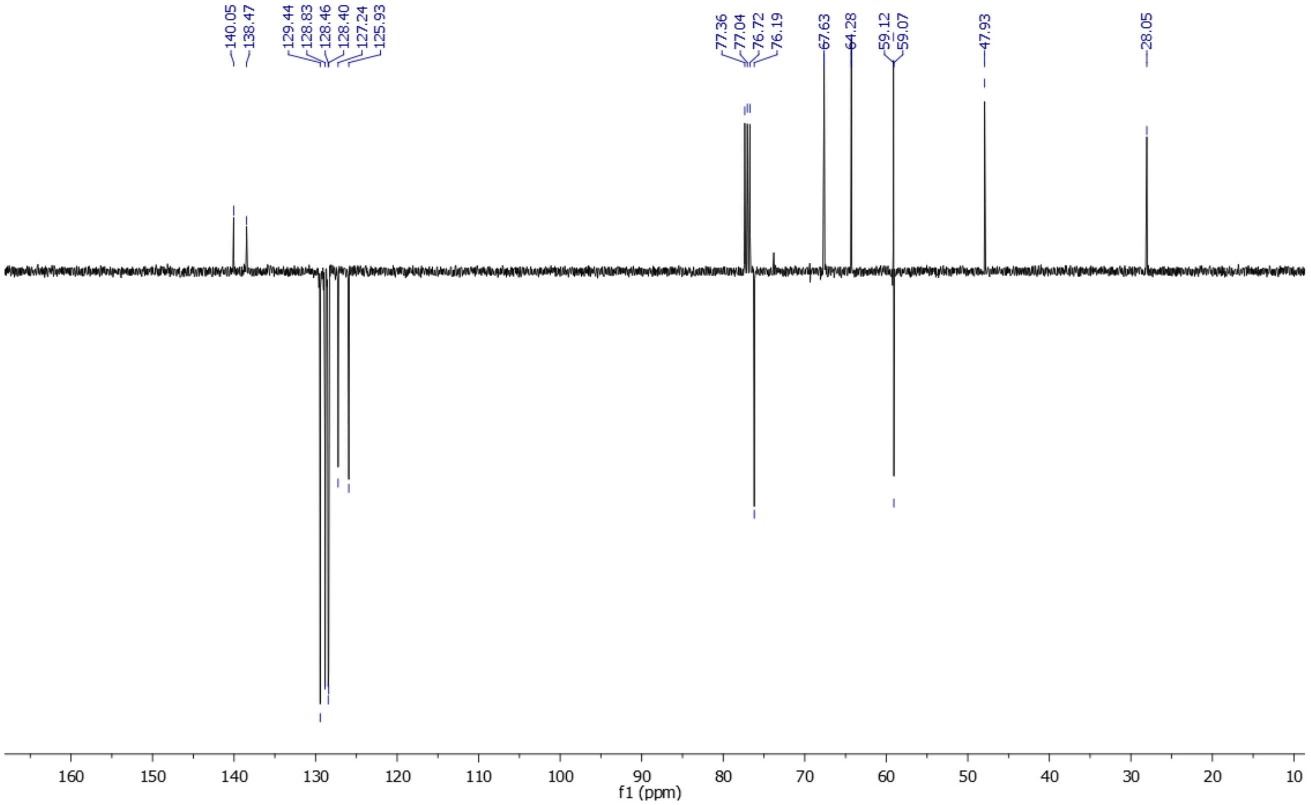

Compound 4b

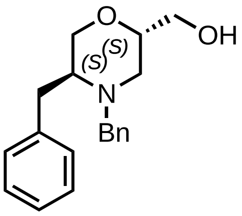
O
OH
(S)
(S)
N
Bn

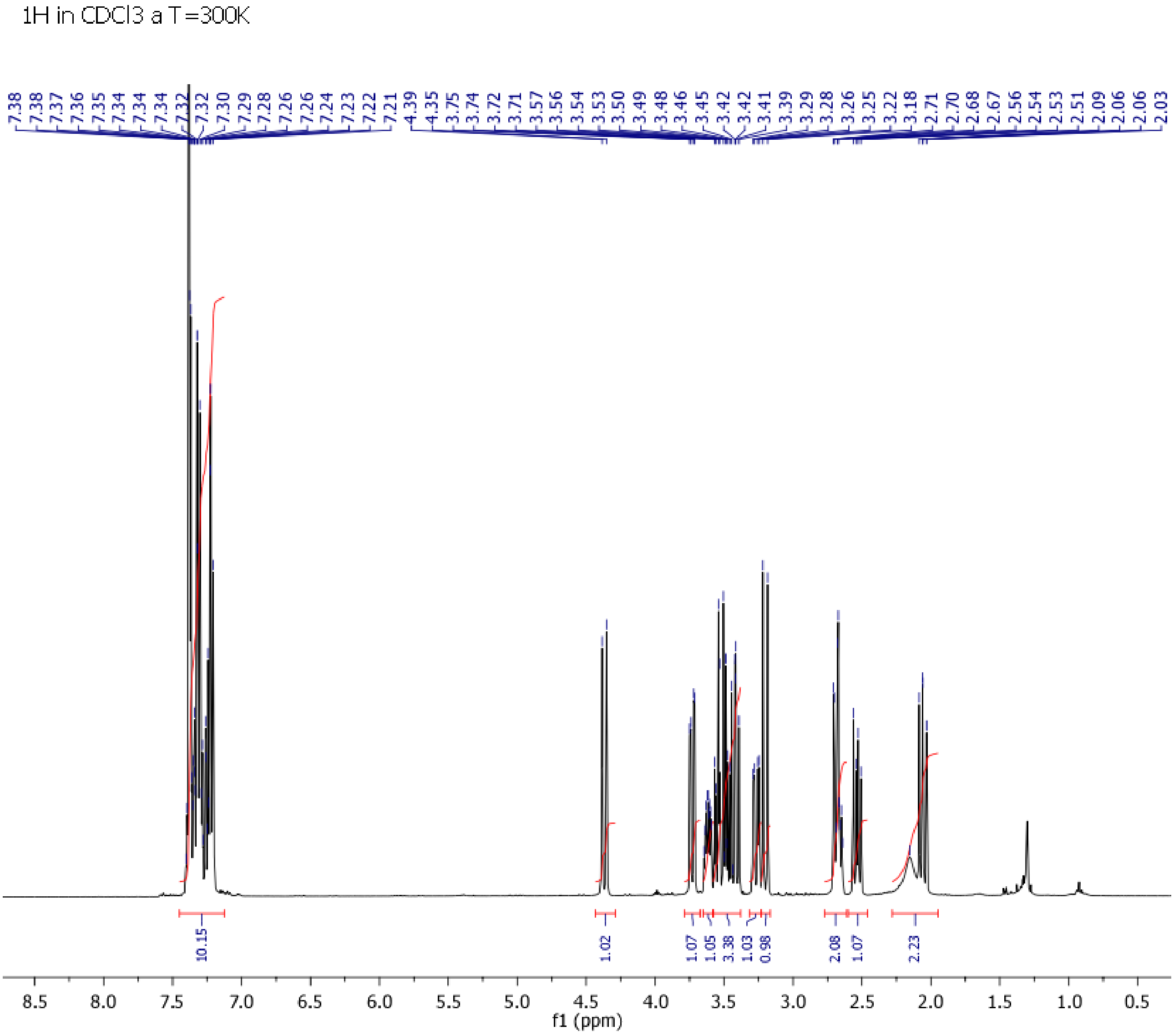
1H in CDCl3 a T=300K
7.38 7.38 7.37 7.36 7.35 7.34 7.34 7.34 7.32 7.32 7.30 7.29 7.28 7.26 7.26 7.24 7.23 7.22 7.21
4.39 4.35 3.75 3.74 3.72 3.71 3.57 3.56 3.54 3.53 3.50 3.49 3.48 3.46 3.45 3.42 3.42 3.41 3.39 3.29 3.28 3.26 3.25 3.22 3.18 2.71 2.70 2.68 2.67 2.56 2.54 2.53 2.51 2.09 2.06 2.03
10.15
1.02
1.07
1.05
3.38
1.03
0.98
2.08
1.07
2.23
8.5 8.0 7.5 7.0 6.5 6.0 5.5 5.0 4.5 4.0 3.5 3.0 2.5 2.0 1.5 1.0 0.5
f1 (ppm)

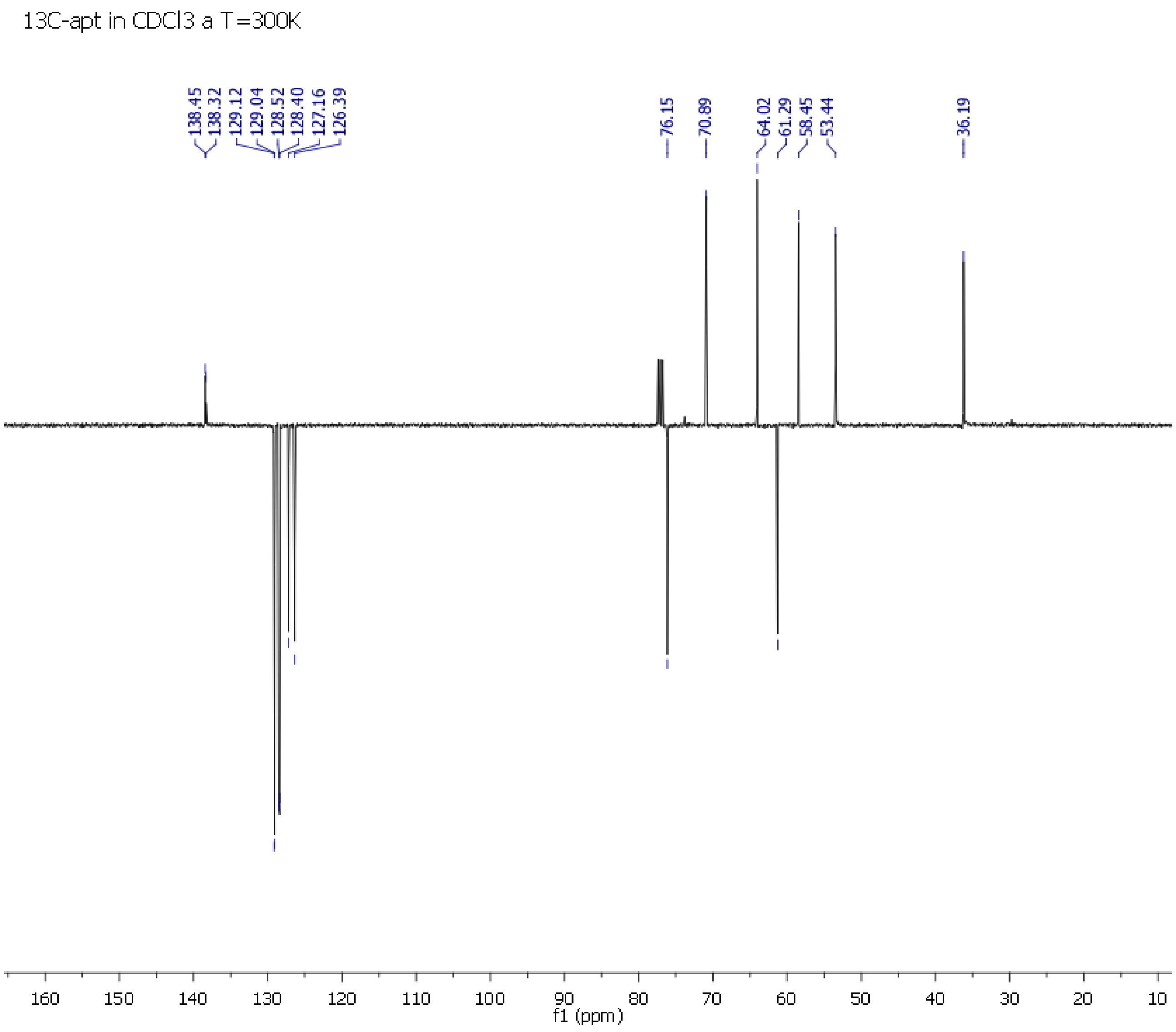
13C-apt in CDCl3 a T=300K
138.45
138.32
129.12
129.04
128.52
128.40
127.16
126.39
76.15
70.89
64.02
61.29
58.45
53.44
36.19
160
150
140
130
120
110
100
90
80
70
60
50
40
30
20
10
f1 (ppm)

Compound 4c

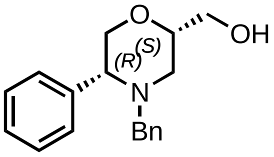

1H in CDCl3 a T=300K

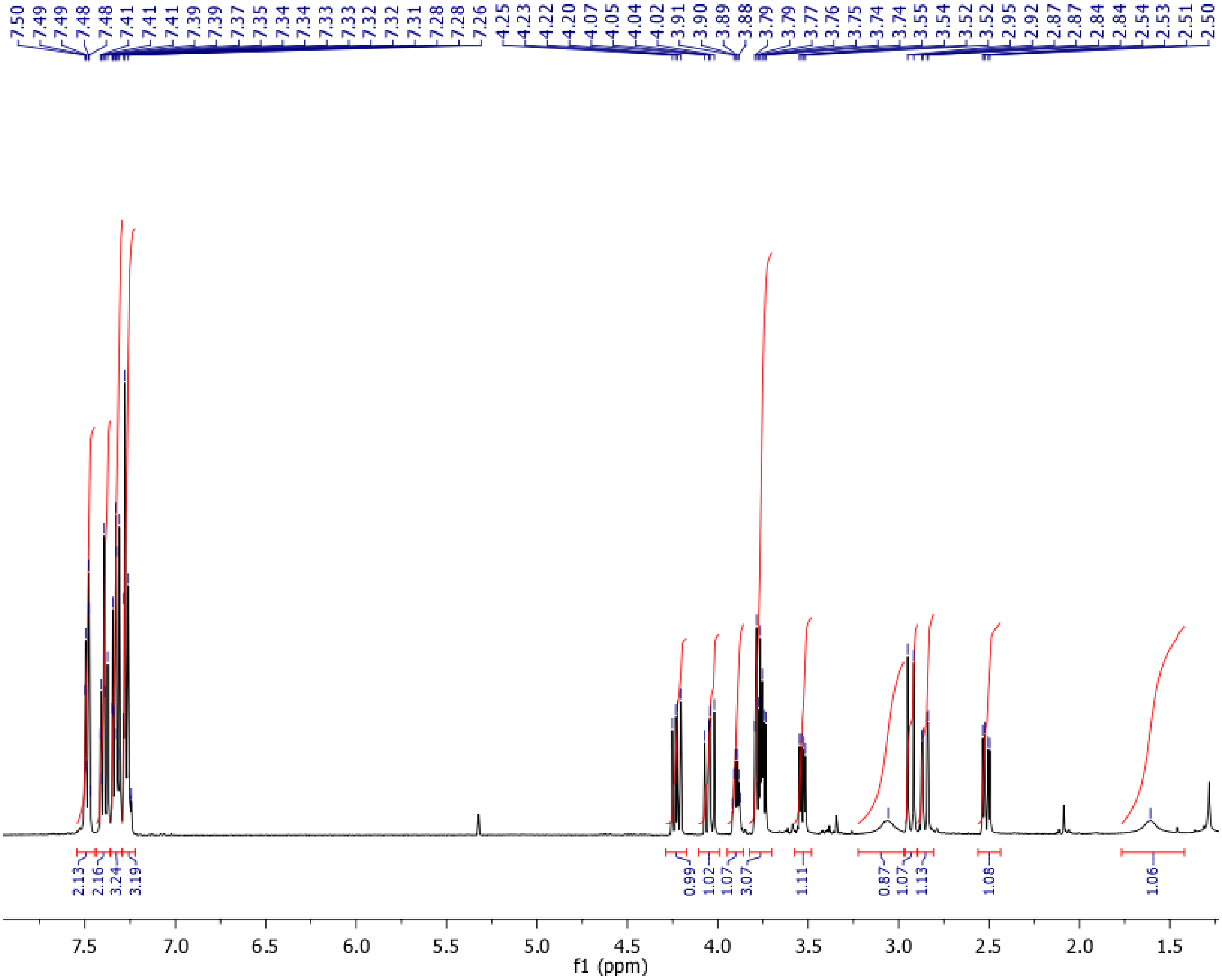

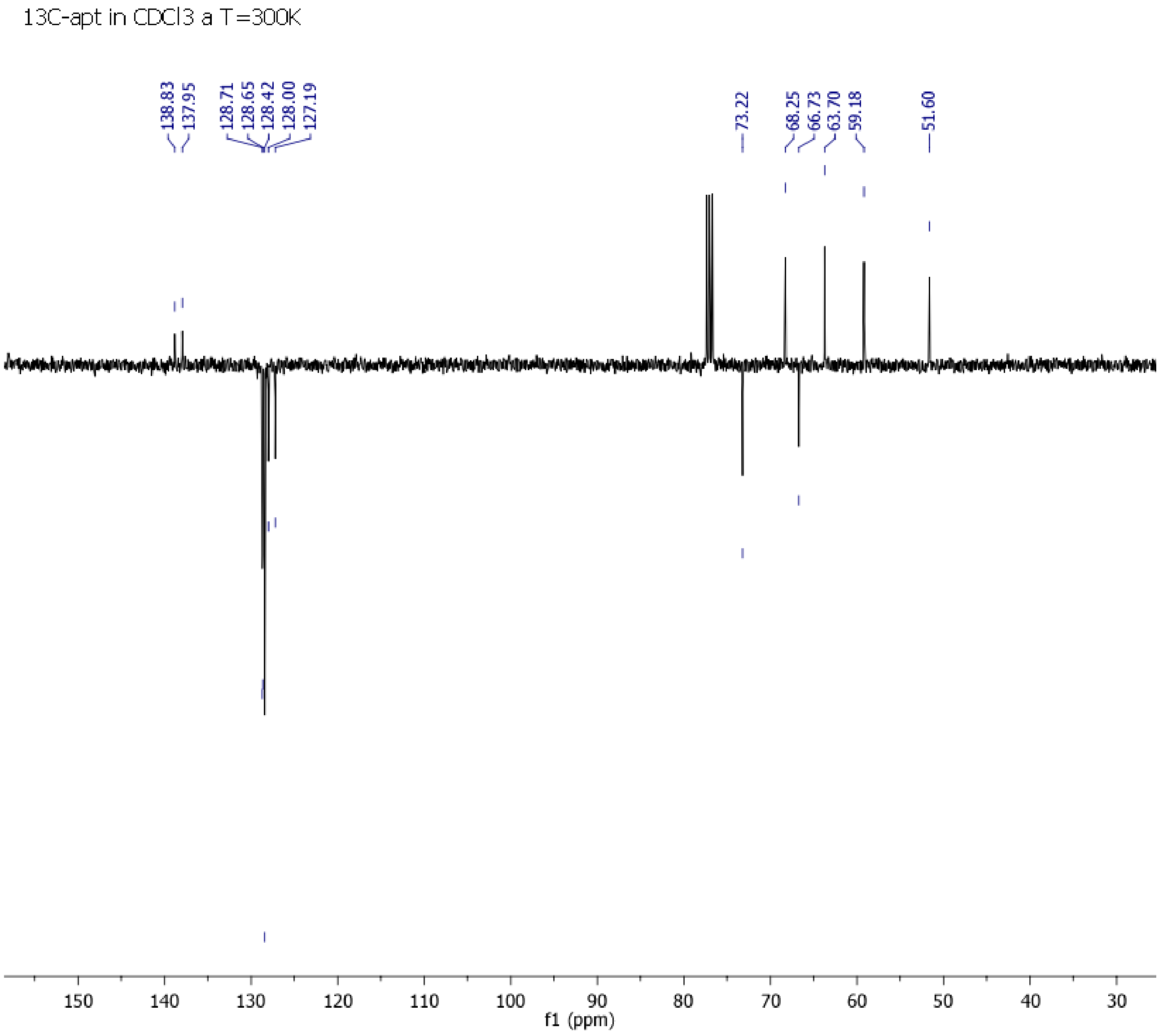
13C-apt in CDCl3 a T=300K
138.83
137.95
128.71
128.65
128.42
128.00
127.19
73.22
68.25
66.73
63.70
59.18
51.60
150
140
130
120
110
100
90
80
70
60
50
40
30
f1 (ppm)

Compound 4d

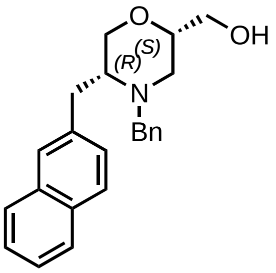
O
OH
(S)
(R)
N
Bn

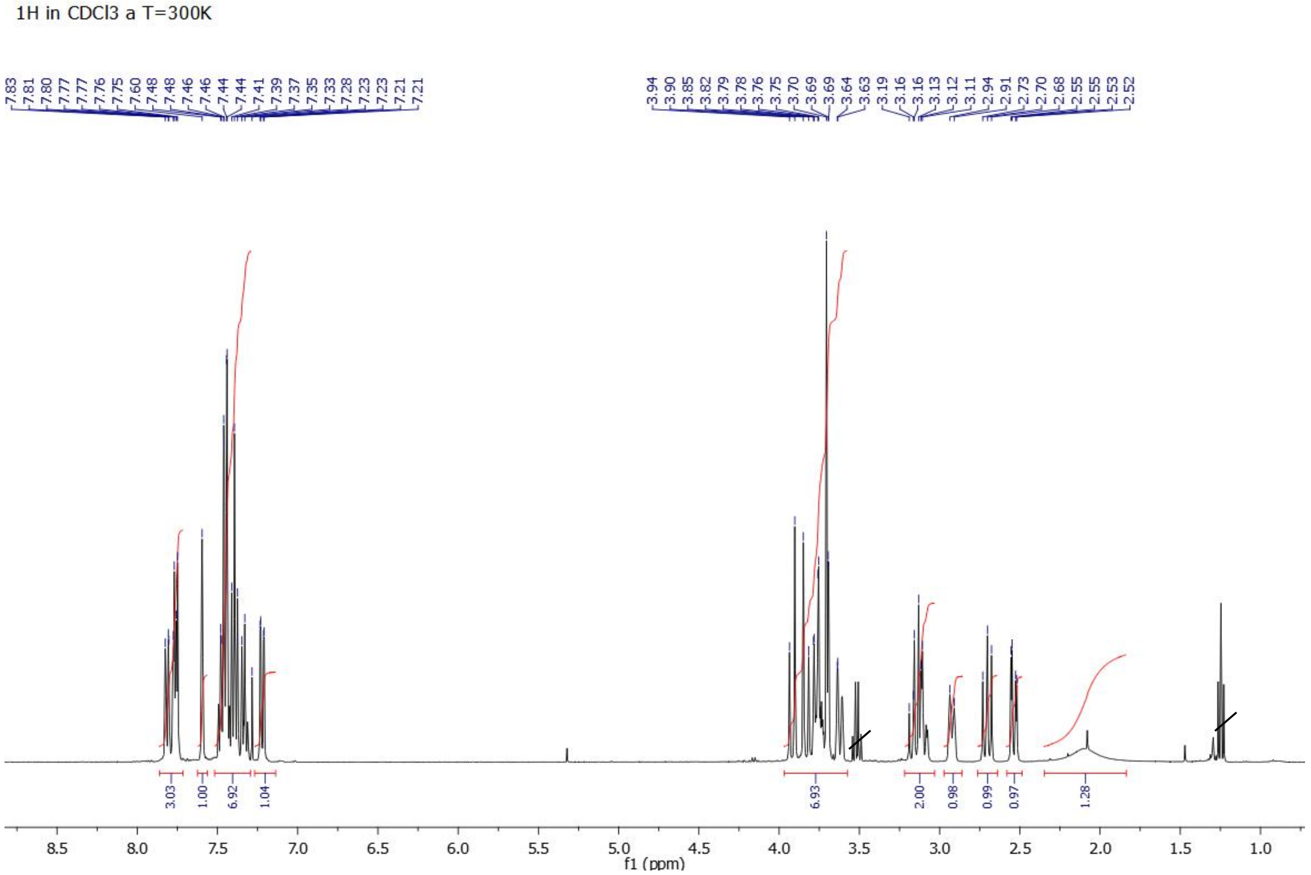
1H in CDCl3 a T=300K
7.83 7.81 7.80 7.77 7.77 7.76 7.75 7.60 7.48 7.46 7.44 7.44 7.41 7.39 7.37 7.35 7.33 7.28 7.23 7.23 7.21 7.21
3.94 3.90 3.85 3.82 3.79 3.78 3.76 3.75 3.70 3.69 3.69 3.64 3.63 3.19 3.16 3.16 3.13 3.12 3.11 2.94 2.91 2.73 2.70 2.68 2.55 2.55 2.53 2.52
3.03
1.00
6.92
1.04
6.93
2.00
0.98
0.99
0.97
1.28
8.5 8.0 7.5 7.0 6.5 6.0 5.5 5.0 4.5 4.0 3.5 3.0 2.5 2.0 1.5 1.0
f1 (ppm)

13C-apt in CDCl3 a T=300K

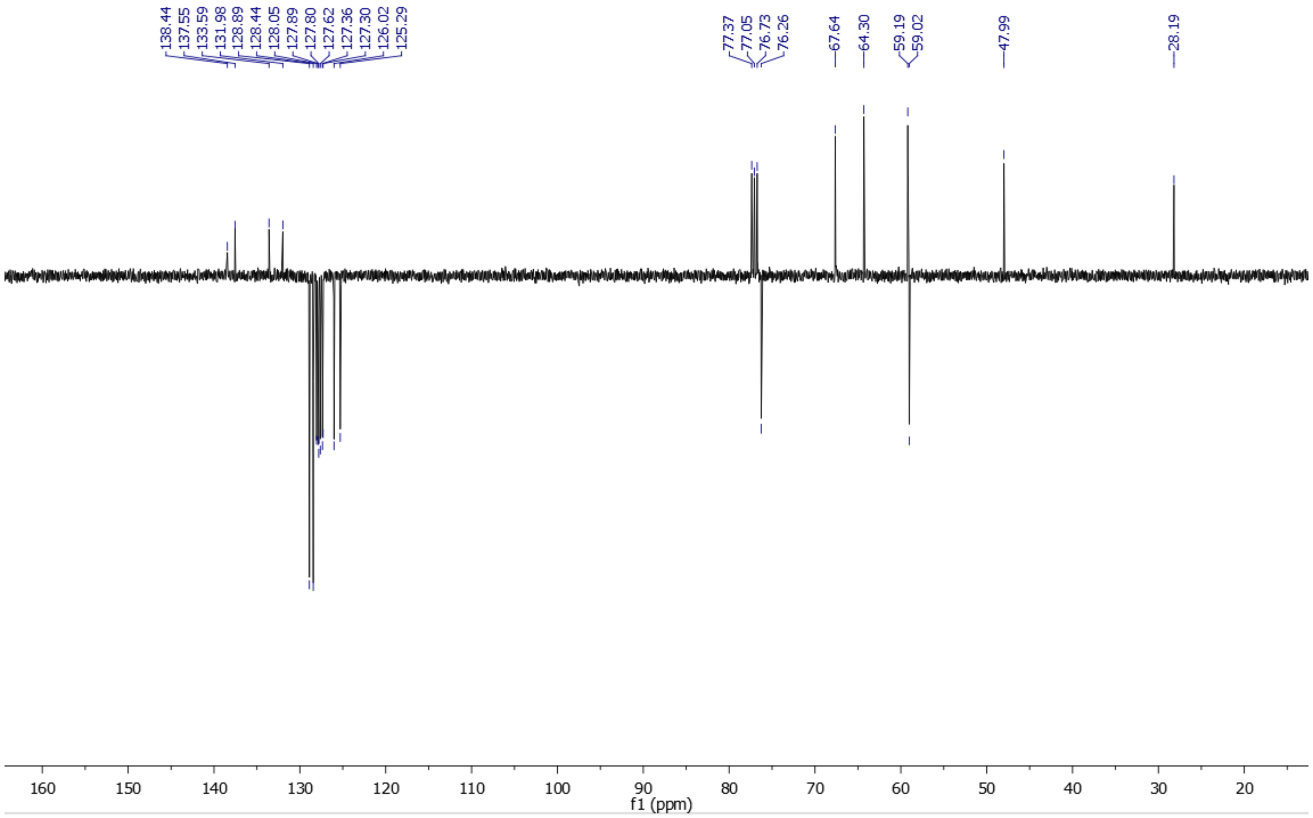

Compound 5a

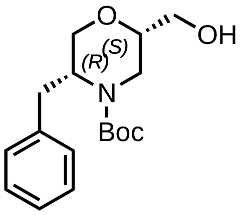

Mixture of rotamers (6:4)

1H in CDCl3 a T=300K

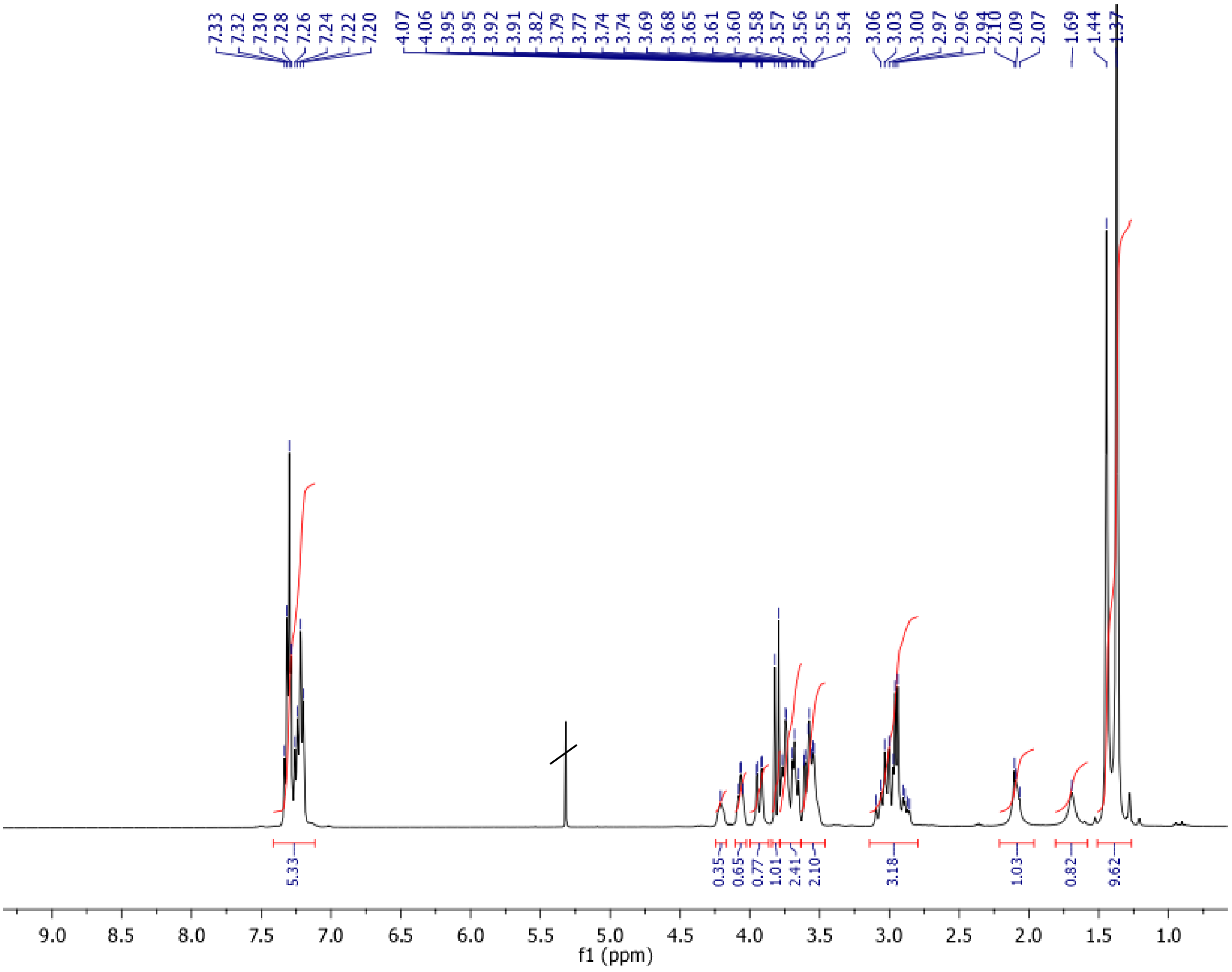

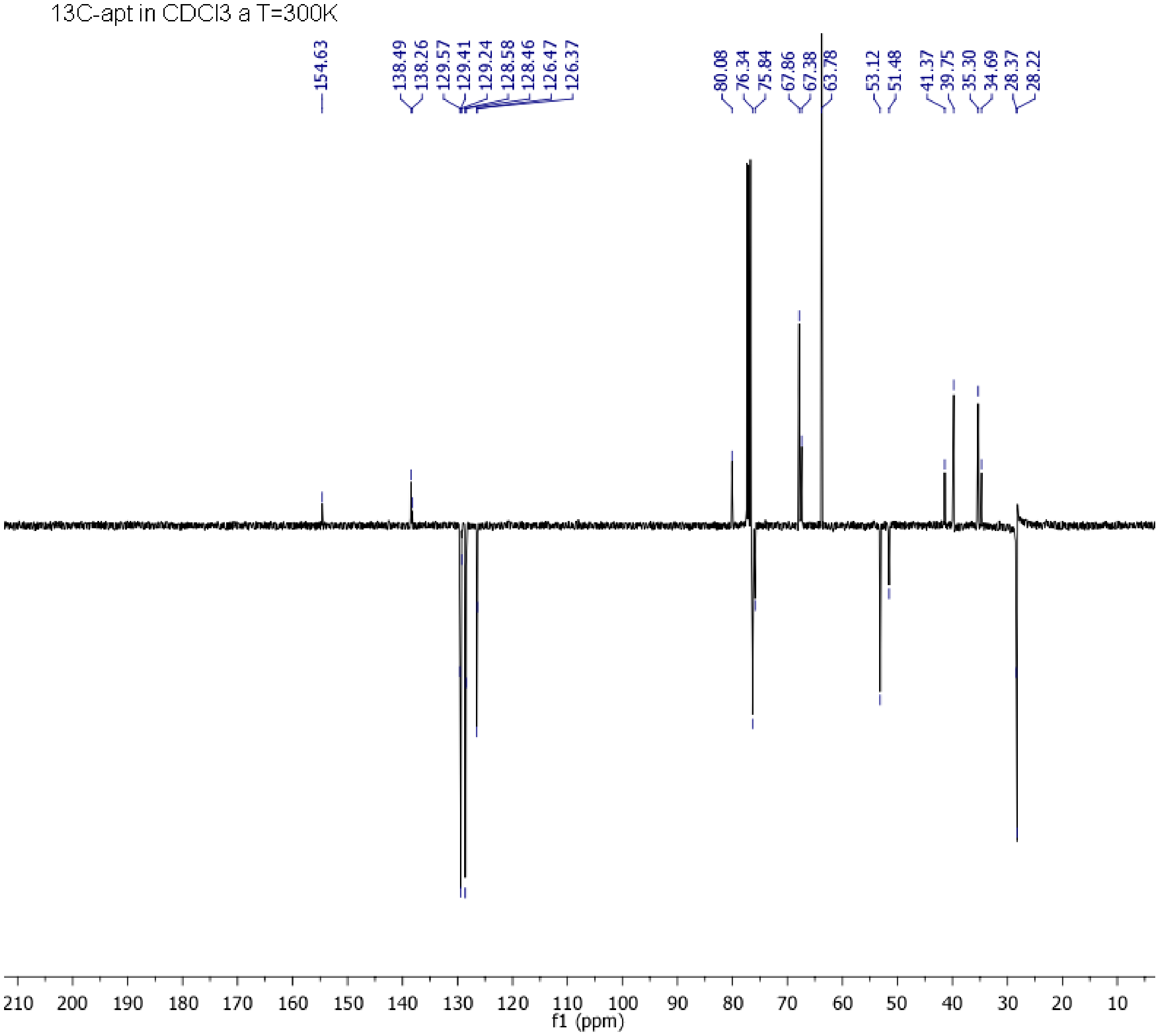
13C-apt in CDCl3 a T=300K
154.63
138.49
138.26
129.57
129.41
129.24
128.58
128.46
126.47
126.37
80.08
76.34
75.84
67.86
67.38
63.78
53.12
51.48
41.37
39.75
35.30
34.69
28.37
28.22
210 200 190 180 170 160 150 140 130 120 110 100 90 80 70 60 50 40 30 20 10
f1 (ppm)

Compound 5b

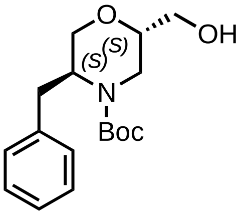

1H in CDCl3 a T=300K

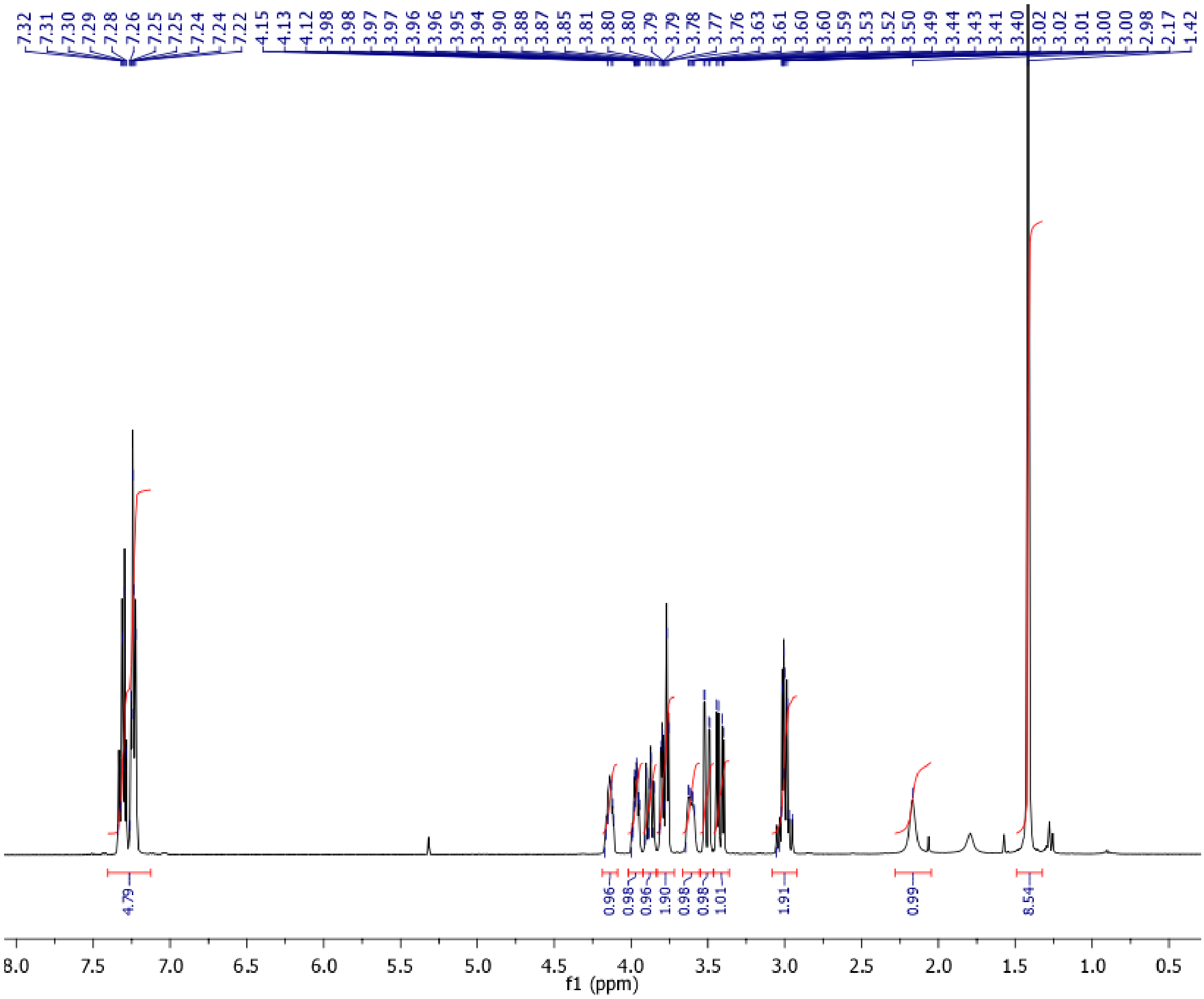

13C-apt in CDCl3 a T=300K

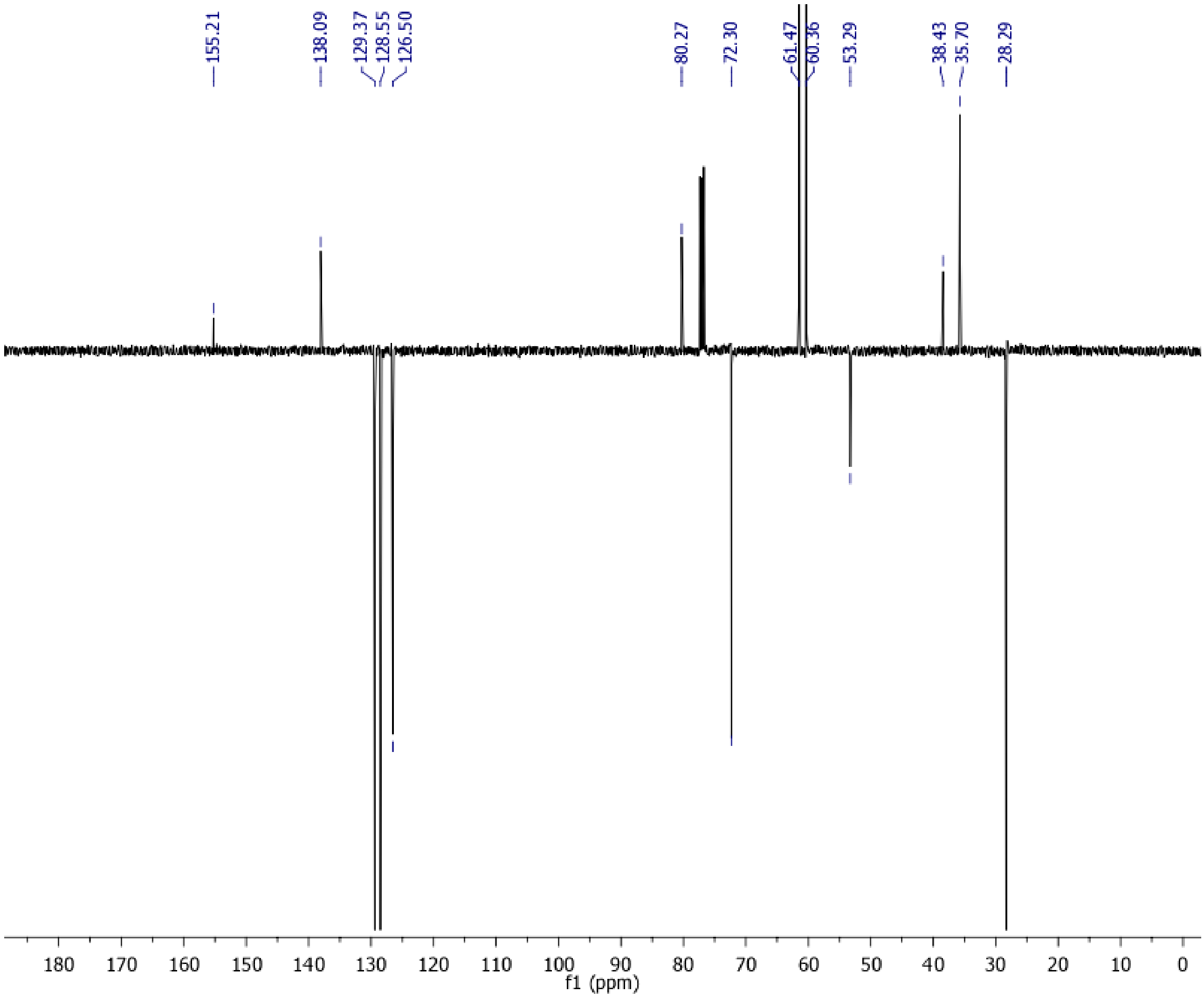

Compound 5c

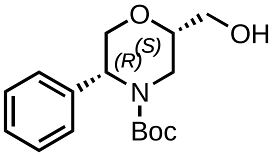

Mixture of rotamers (8:2)

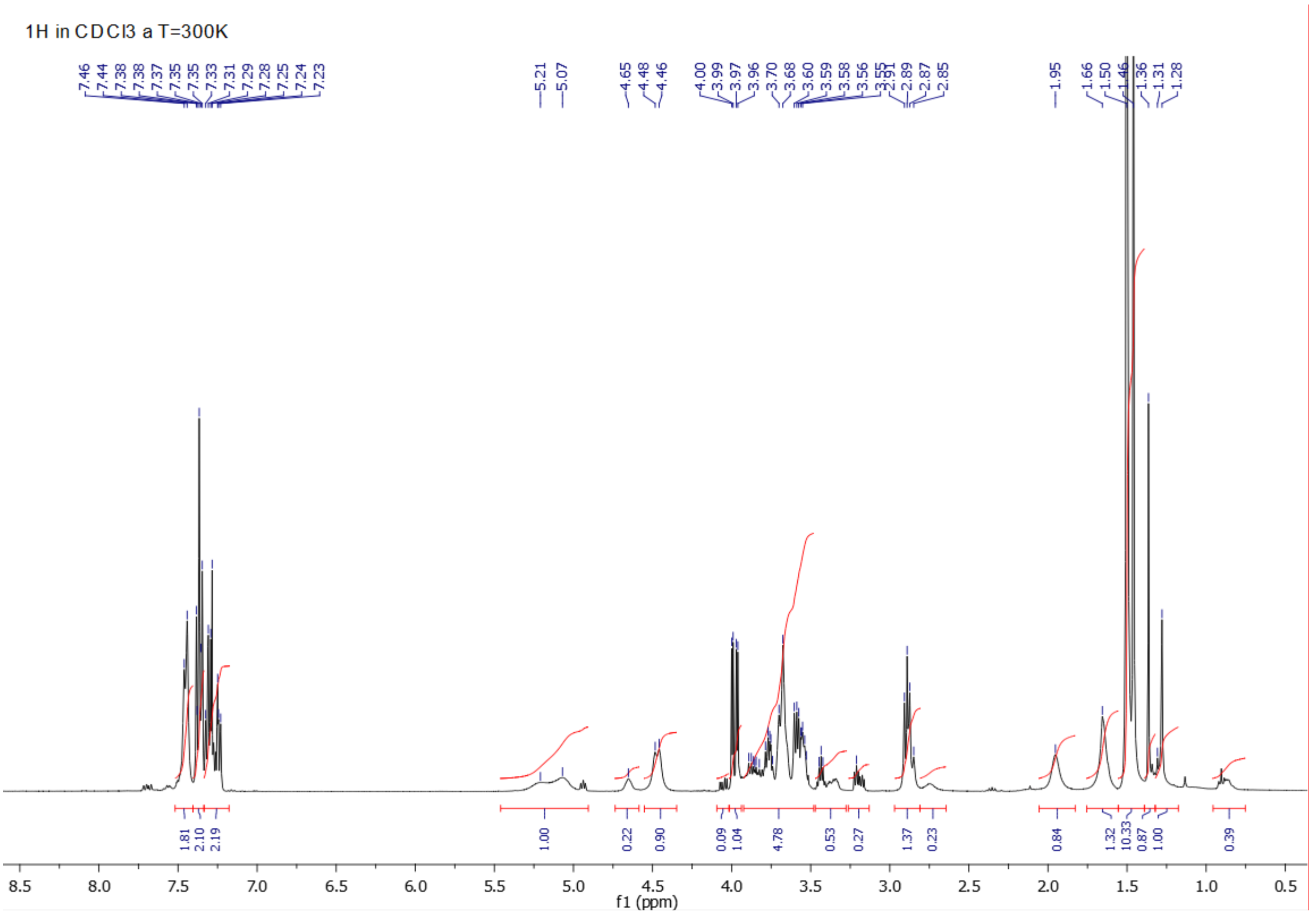

13C-apt in CDCl3 a T=300K

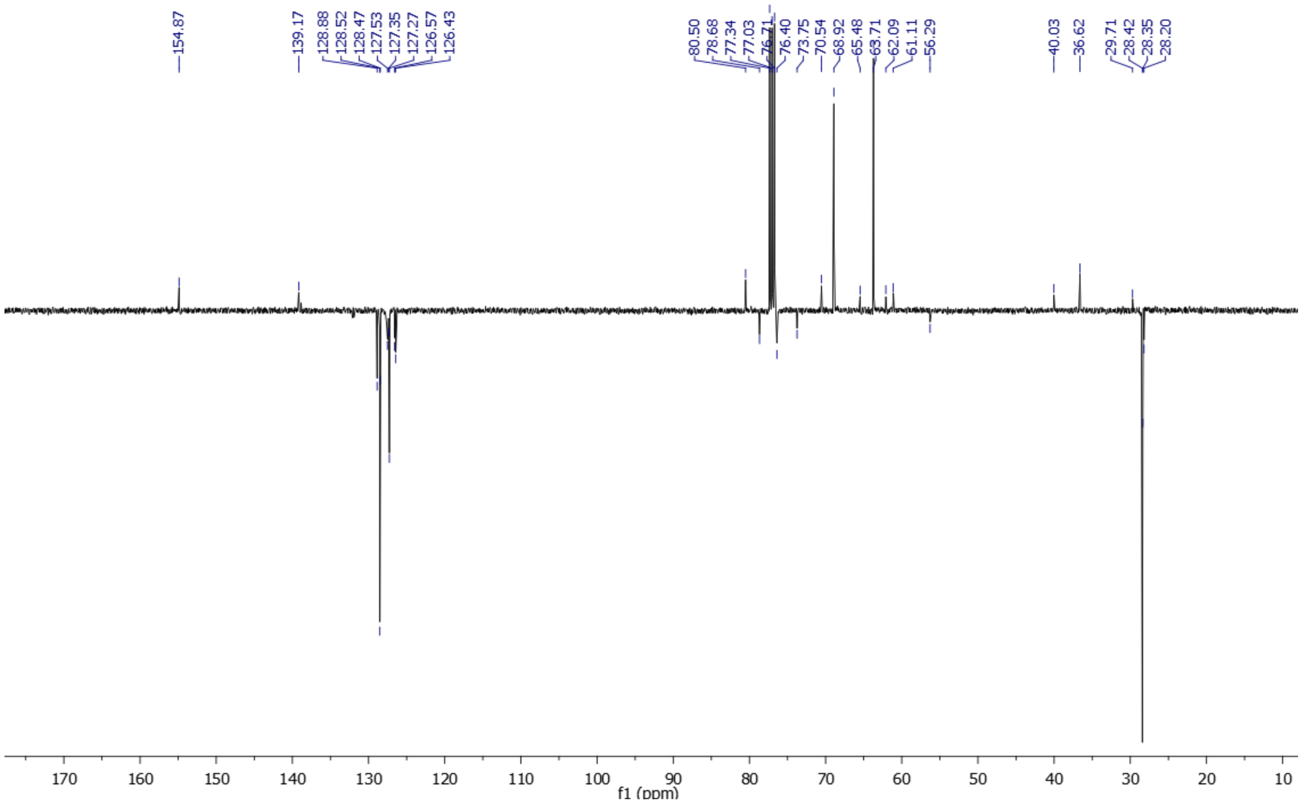

Compound 5d

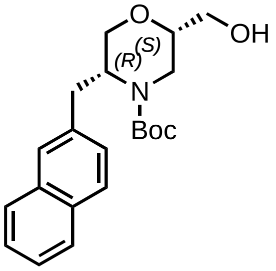

1H in CDCl3 a T=300K

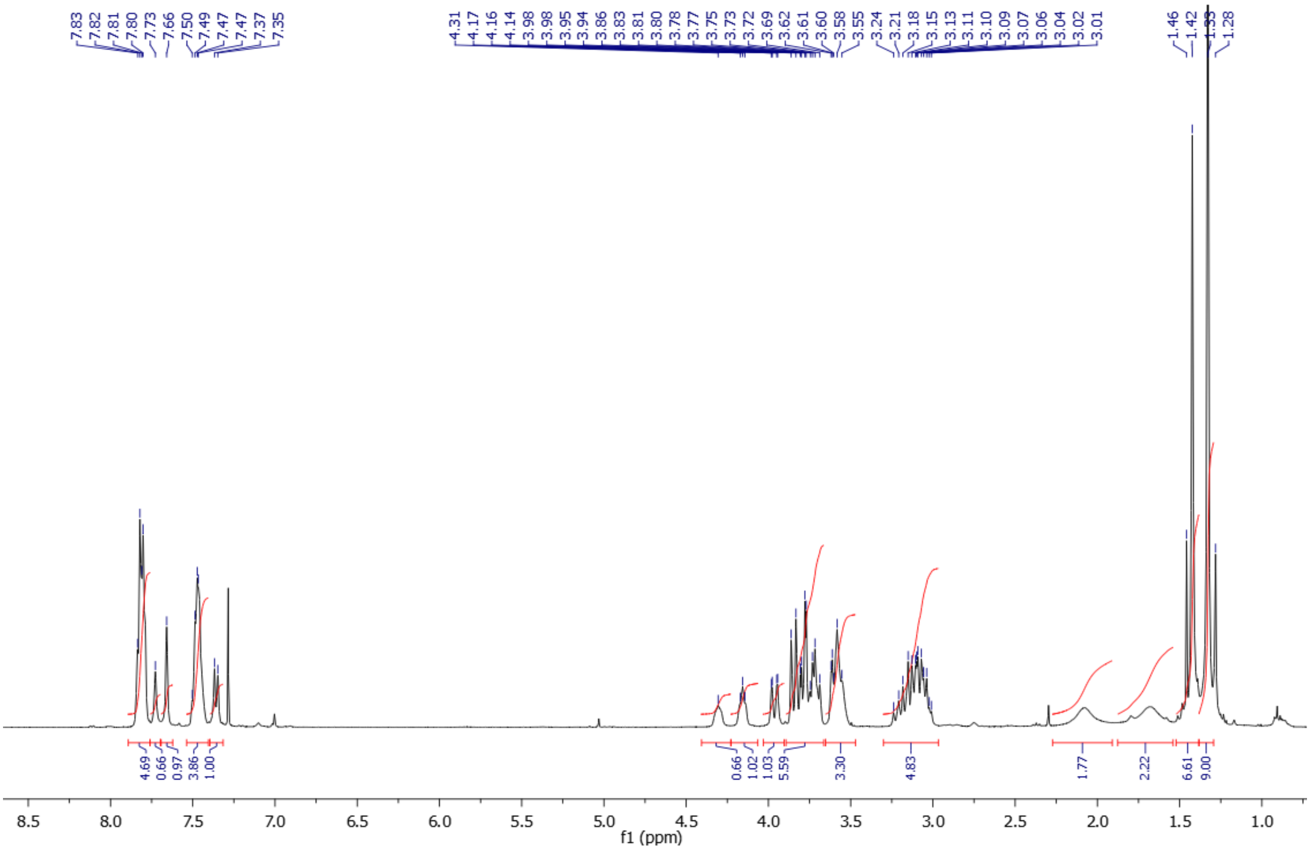

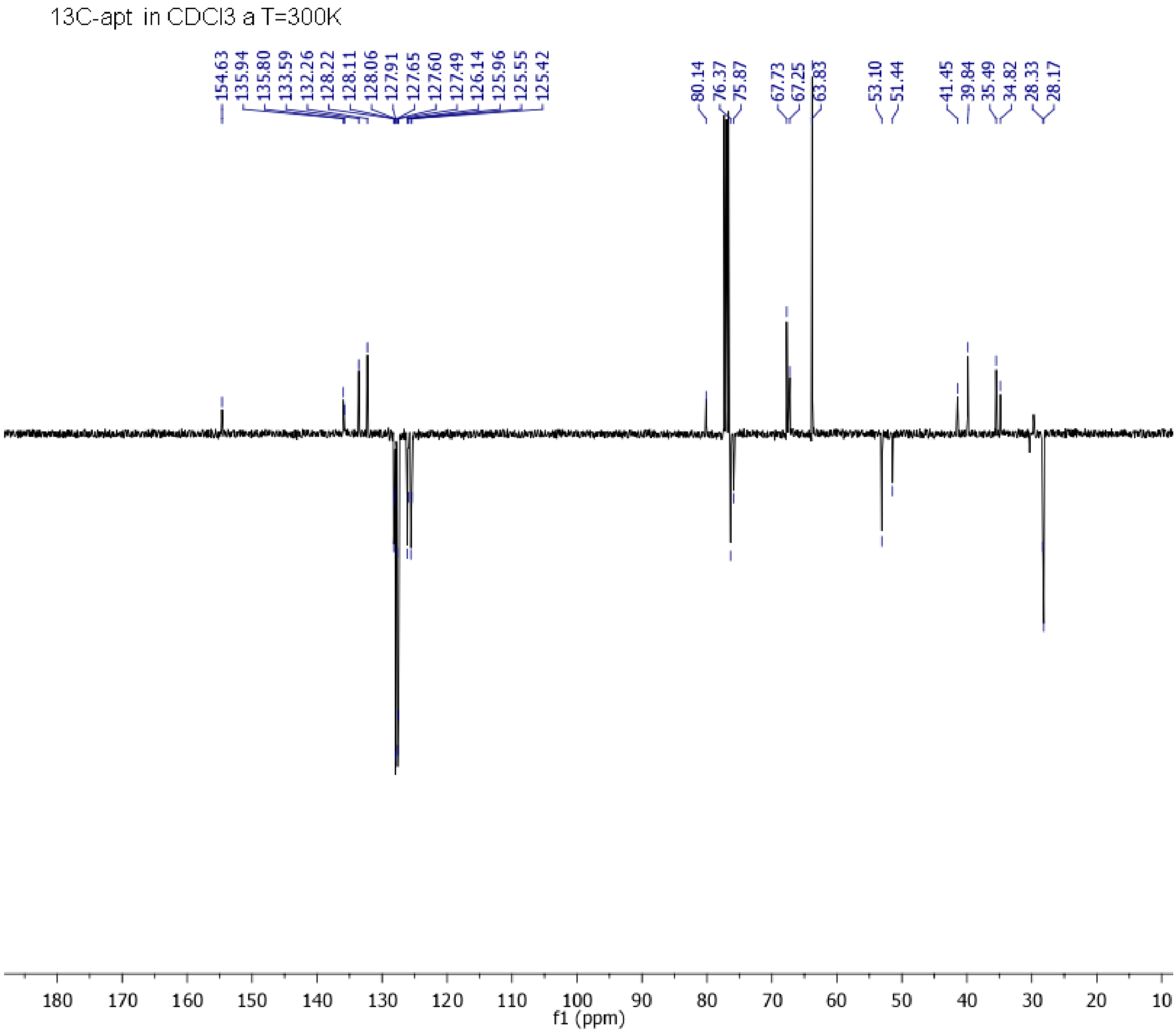
13C-apt in CDCl3 a T=300K
154.63
135.94
135.80
133.59
132.26
128.22
128.11
128.06
127.91
127.65
127.60
127.49
126.14
125.96
125.55
125.42
80.14
76.37
75.87
67.73
67.25
63.83
53.10
51.44
41.45
39.84
35.49
34.82
28.33
28.17
180 170 160 150 140 130 120 110 100 90 80 70 60 50 40 30 20 10
f1 (ppm)

Compound 6a

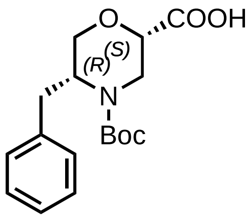

1H in CDCl3 a T=300K

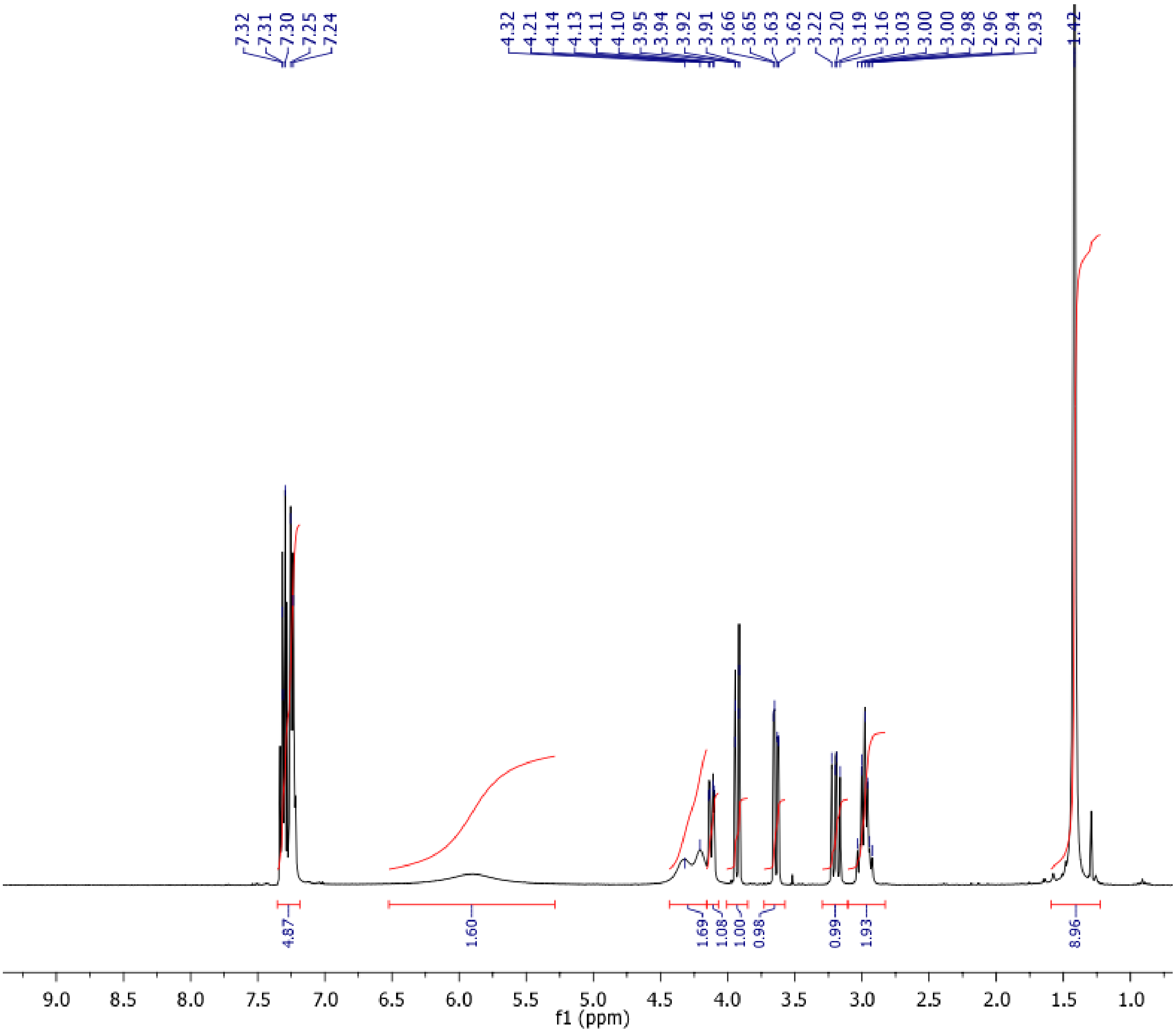

13C-apt in CDCl3 a T=300K

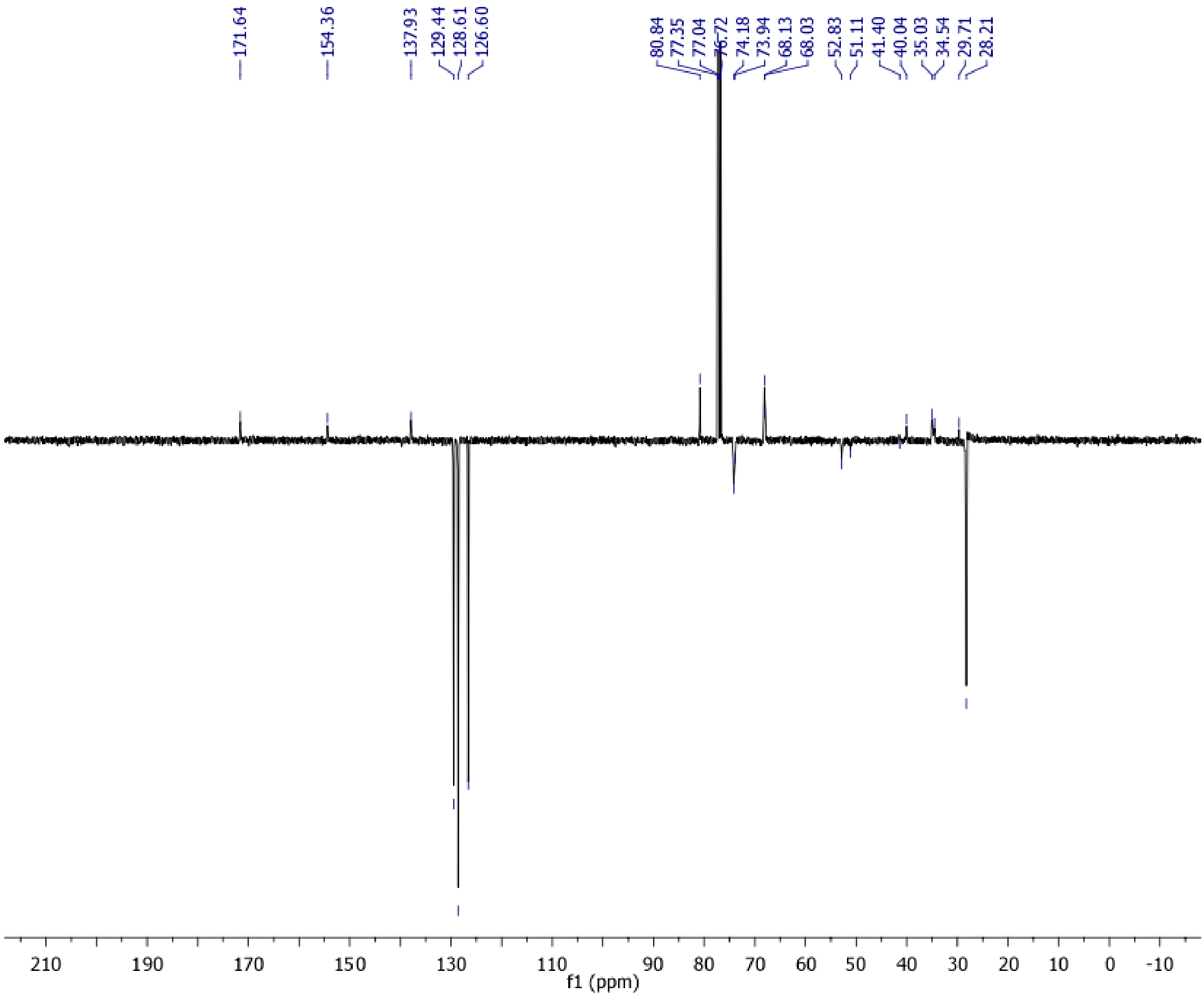

Compound 6b

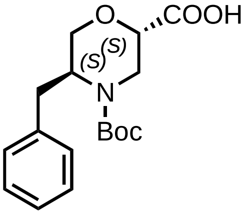

O
COOH
(S)
(S)
N
Boc

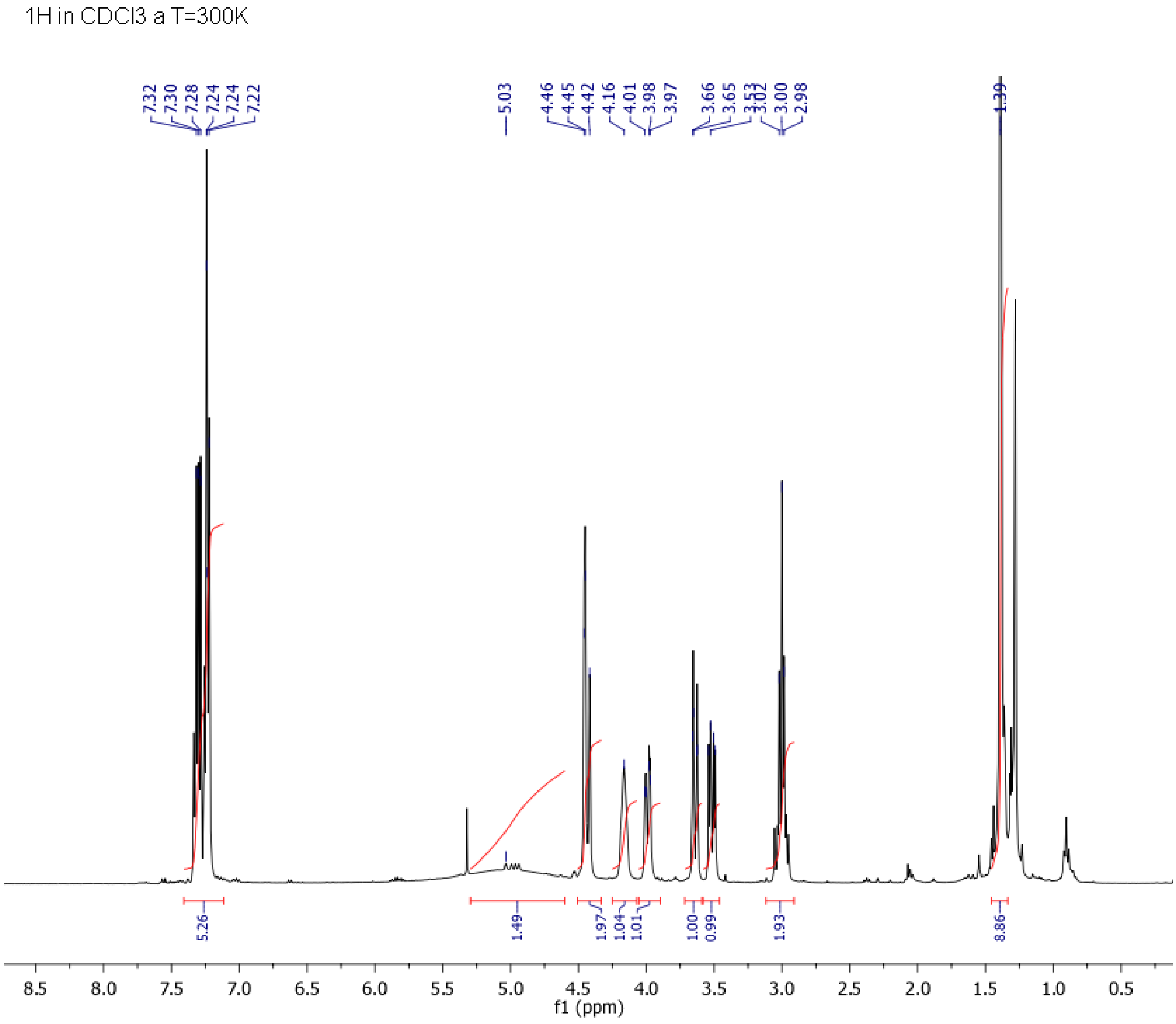

1H in CDCl3 a T=300K
7.32
7.30
7.28
7.24
7.24
7.22
5.03
4.46
4.45
4.42
4.16
4.01
3.98
3.97
3.66
3.65
3.53
3.52
3.02
3.00
2.98
1.39
5.26
1.49
1.97
1.04
1.01
1.00
0.99
1.93
8.86
8.5
8.0
7.5
7.0
6.5
6.0
5.5
5.0
4.5
4.0
3.5
3.0
2.5
2.0
1.5
1.0
0.5
f1 (ppm)

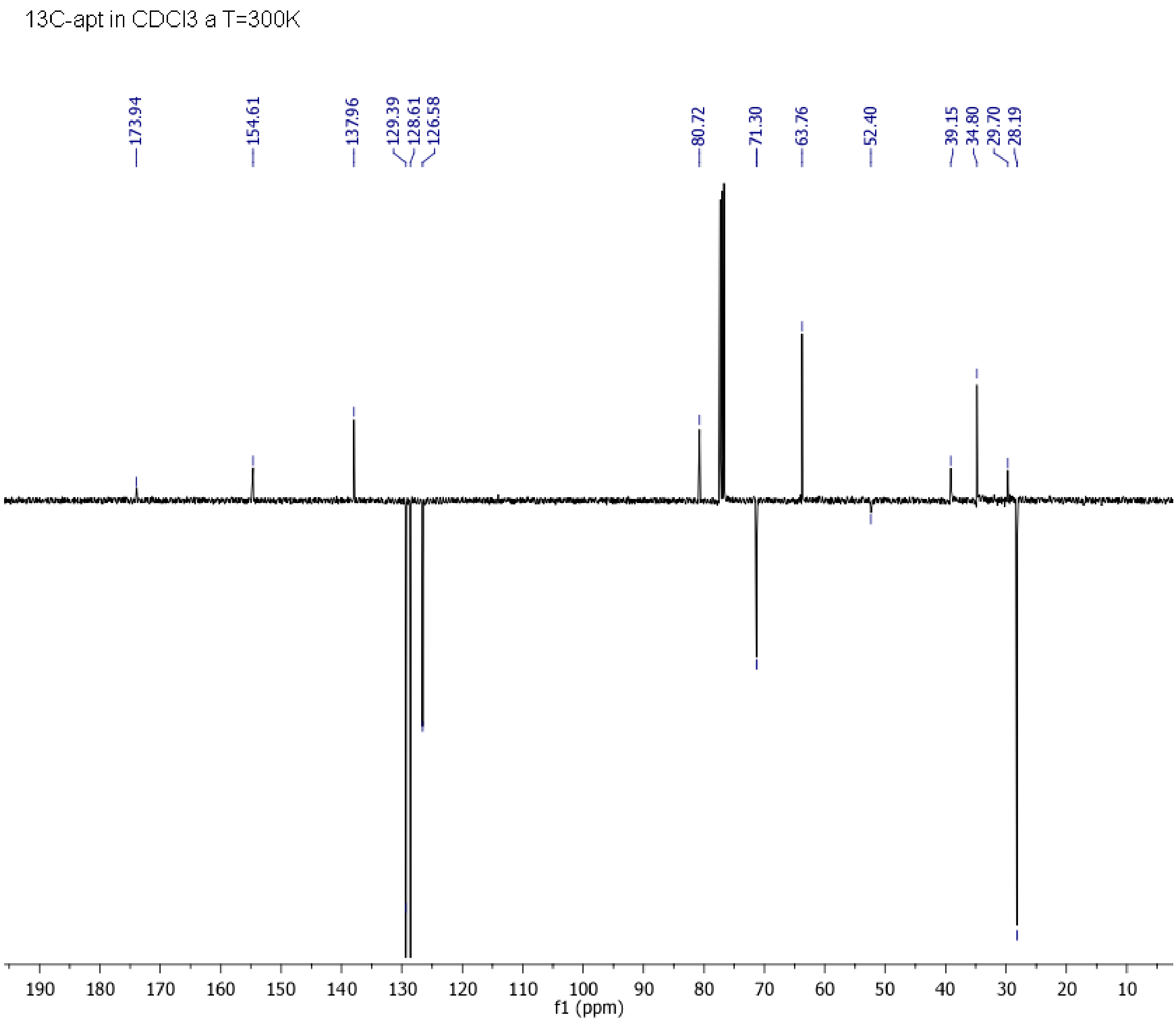

13C-apt in CDCl3 a T=300K
173.94
154.61
137.96
129.39
128.61
126.58
80.72
71.30
63.76
52.40
39.15
34.80
29.70
28.19
190 180 170 160 150 140 130 120 110 100 90 80 70 60 50 40 30 20 10
f1 (ppm)

Compound 6c

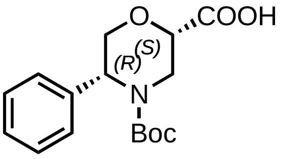
COOH
(S)
(R)
O
N
Boc

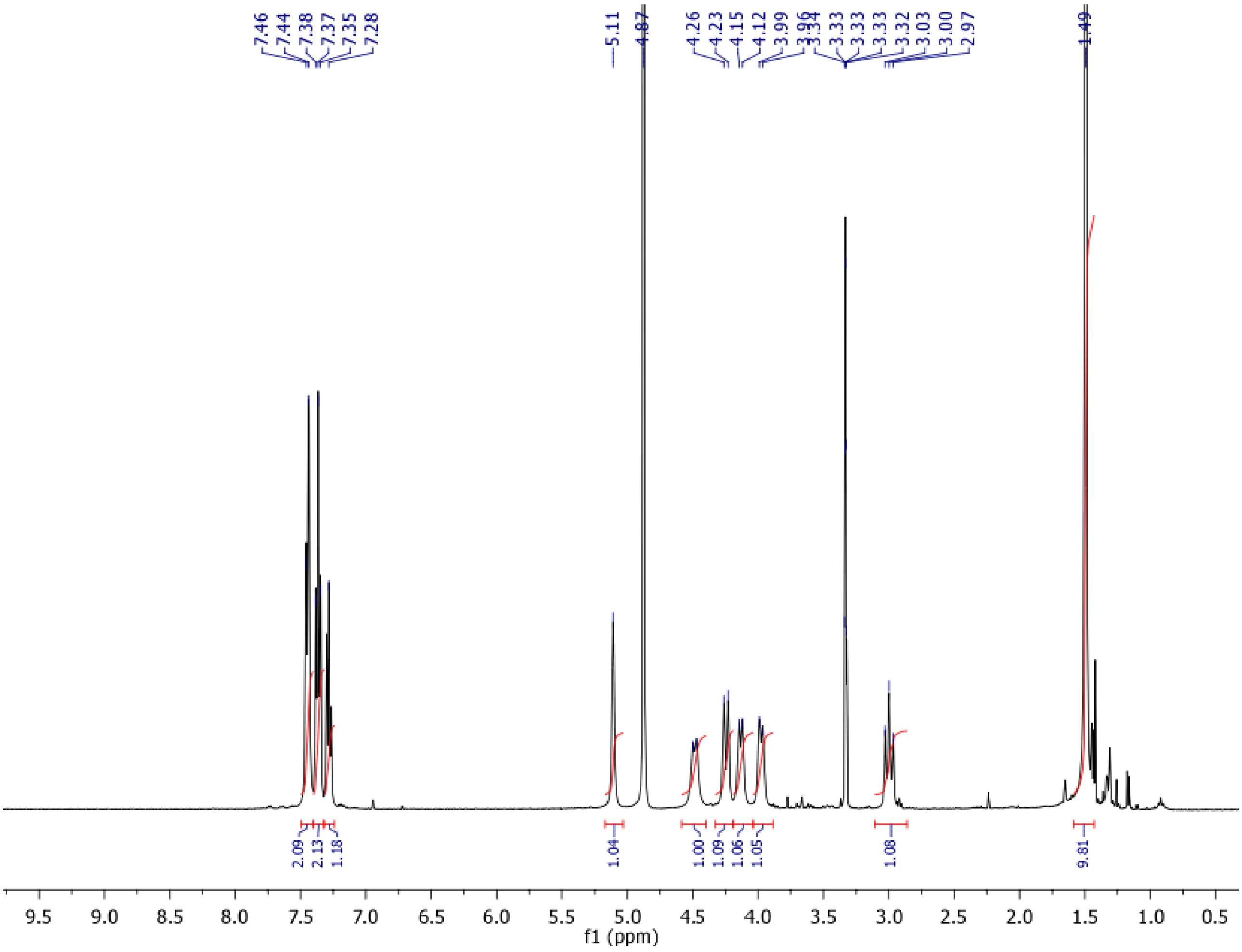
1H in CD3OD a T=300K
7.46
7.44
7.38
7.37
7.35
7.28
5.11
4.87
4.26
4.23
4.15
4.12
3.99
3.96
3.34
3.33
3.33
3.33
3.32
3.03
3.00
2.97
1.49
2.09
2.13
1.18
1.04
1.00
1.09
1.06
1.05
1.08
9.81
9.5
9.0
8.5
8.0
7.5
7.0
6.5
6.0
5.5
5.0
4.5
4.0
3.5
3.0
2.5
2.0
1.5
1.0
0.5
f1 (ppm)

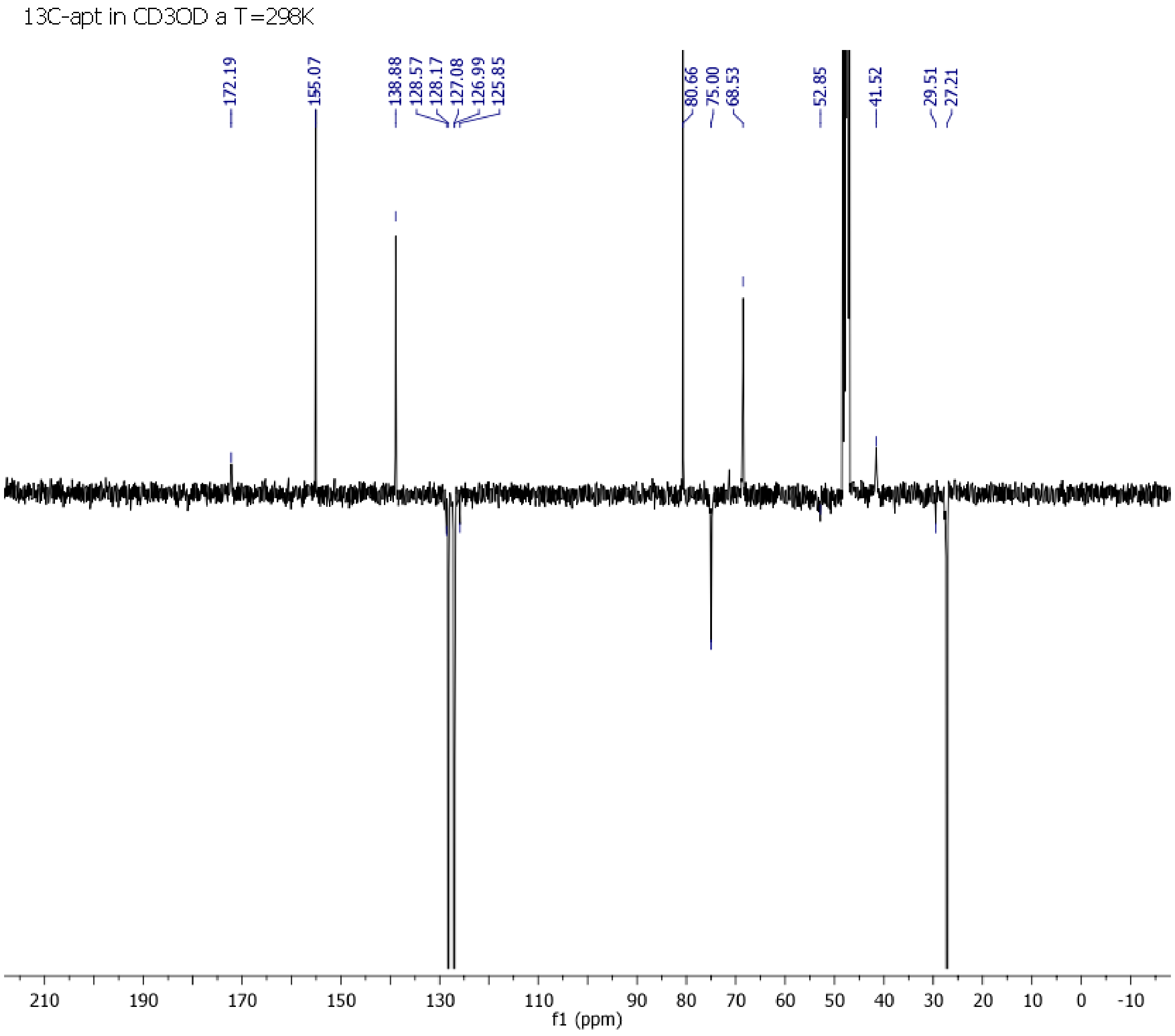
13C-apt in CD3OD a T=298K
172.19
155.07
138.88
128.57
128.17
127.08
126.99
125.85
80.66
75.00
68.53
52.85
41.52
29.51
27.21
210
190
170
150
130
110
90
80
70
60
50
40
30
20
10
0
-10
f1 (ppm)

Compound 6d

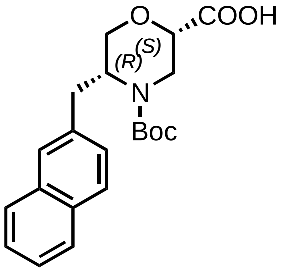

1H in CD3OD a T=300K

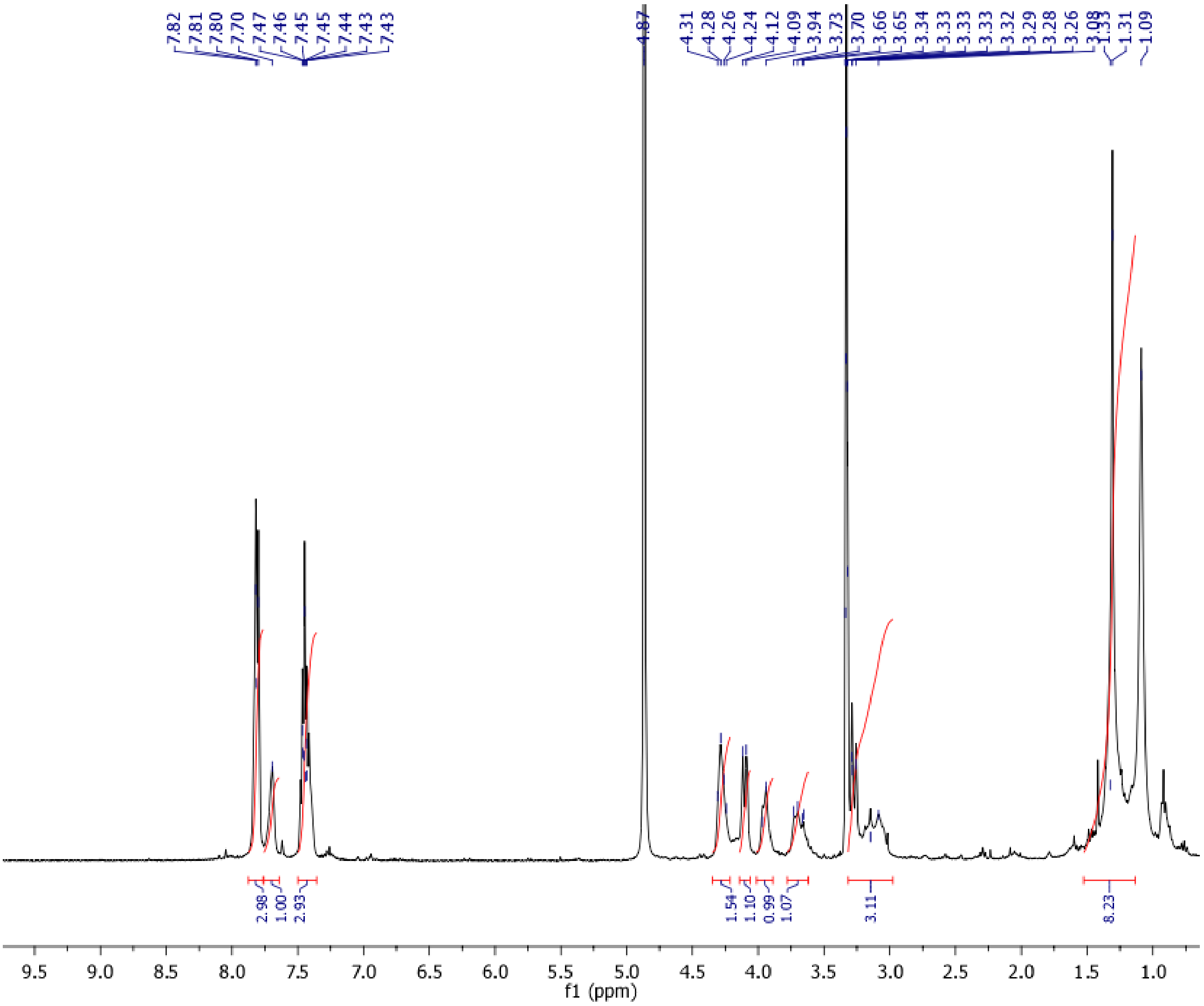

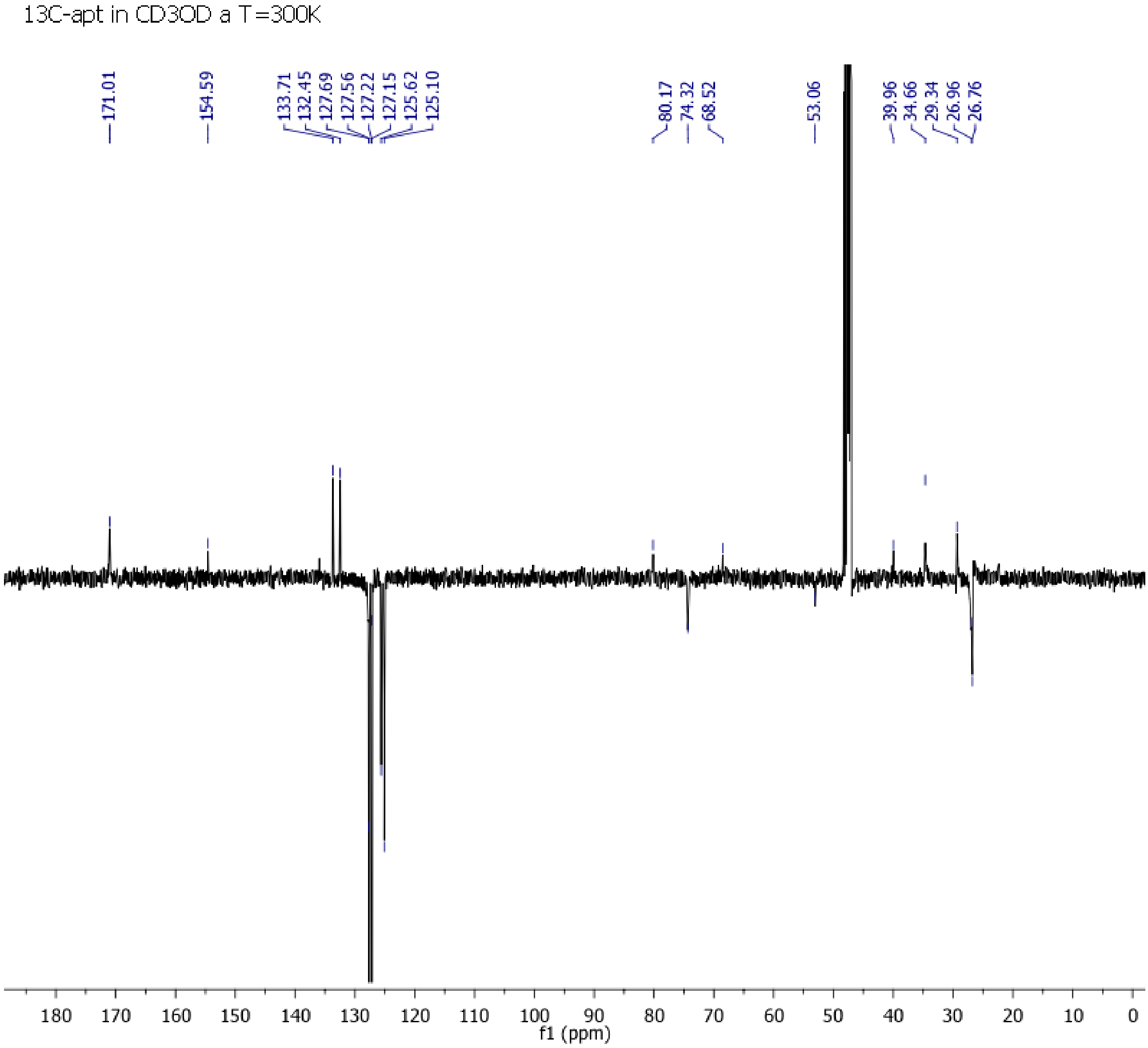

13C-apt in CD3OD a T=300K
171.01
154.59
133.71
132.45
127.69
127.56
127.22
127.15
125.62
125.10
80.17
74.32
68.52
53.06
39.96
34.66
29.34
26.96
26.76
180 170 160 150 140 130 120 110 100 90 80 70 60 50 40 30 20 10 0
f1 (ppm)

Catalyst I

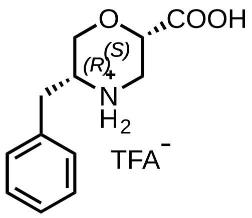

1H in CD3OD a T=300K

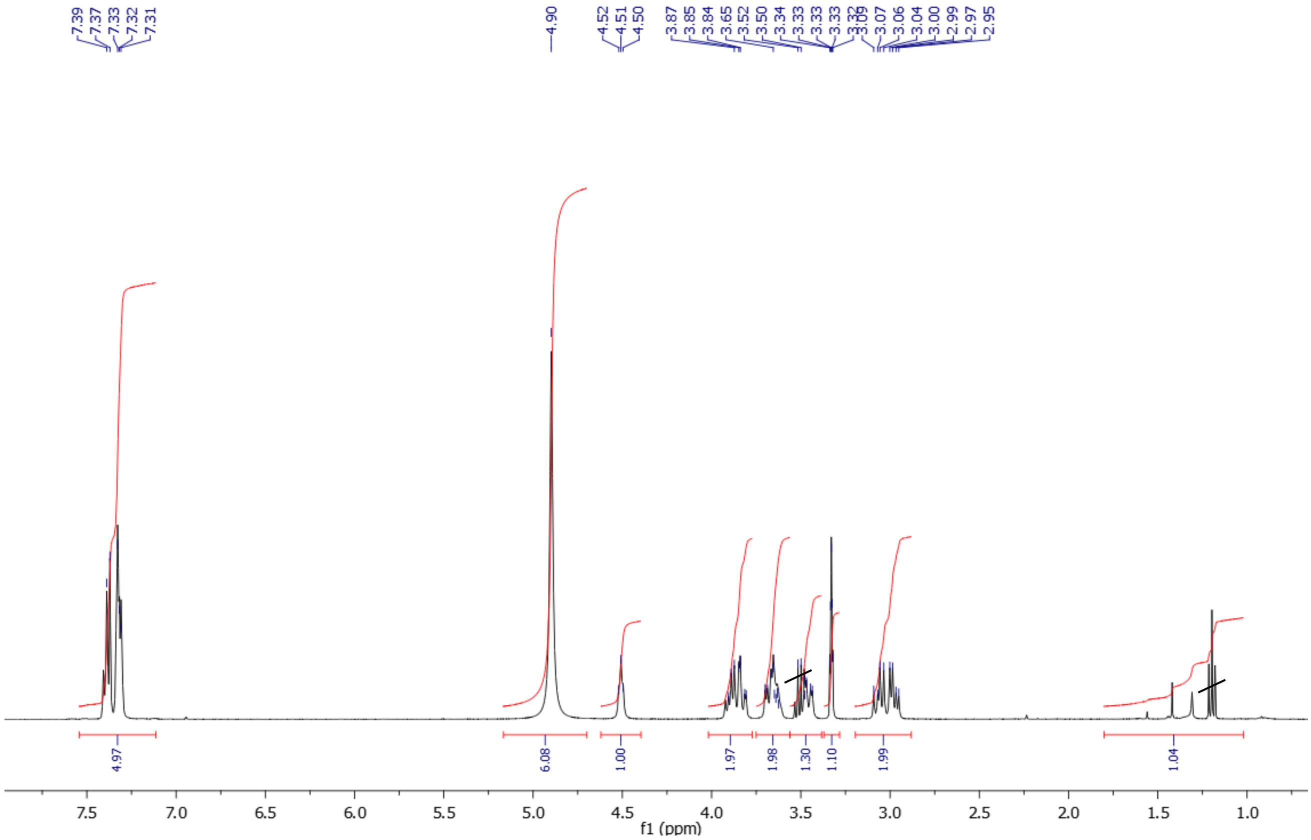

13C-apt in CD3OD a T=300K

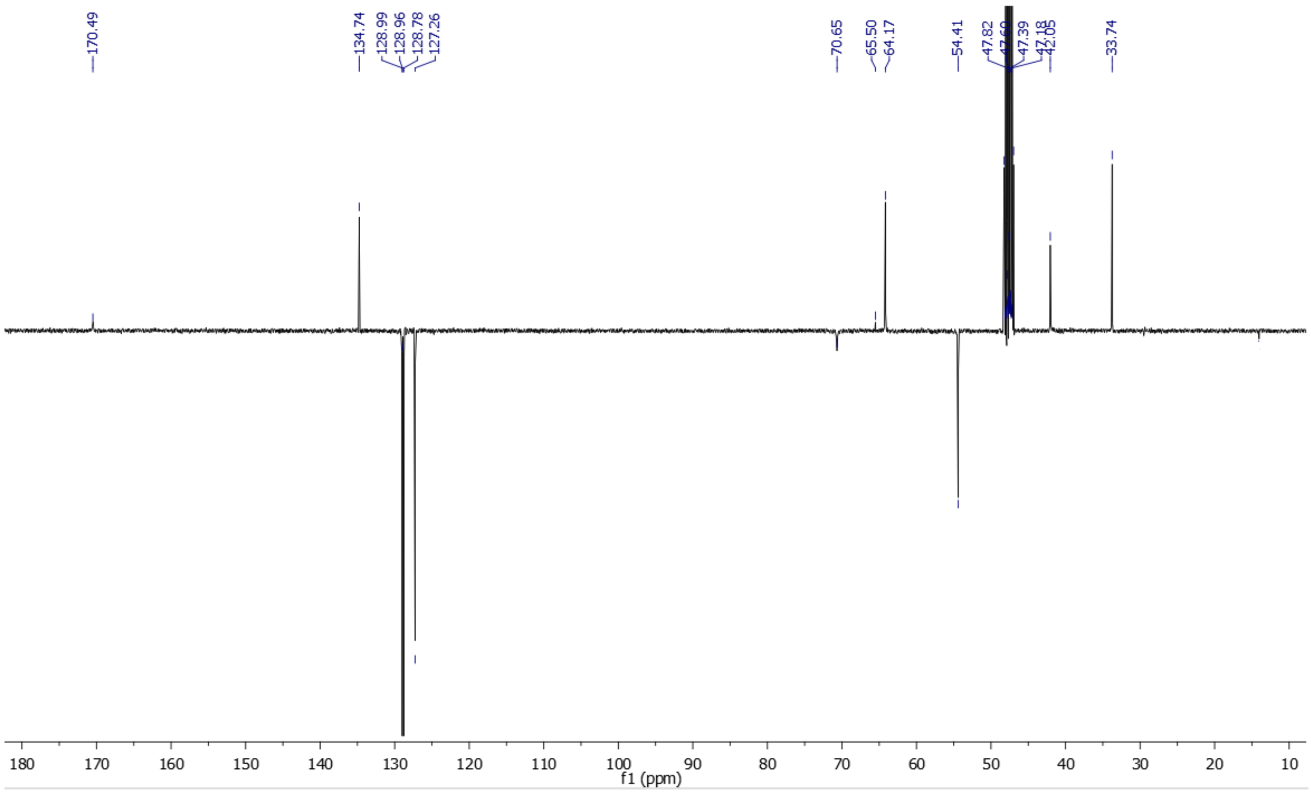

Catalyst II

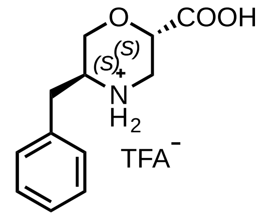

O
COOH
(S)
(S)
N
H2
TFA-

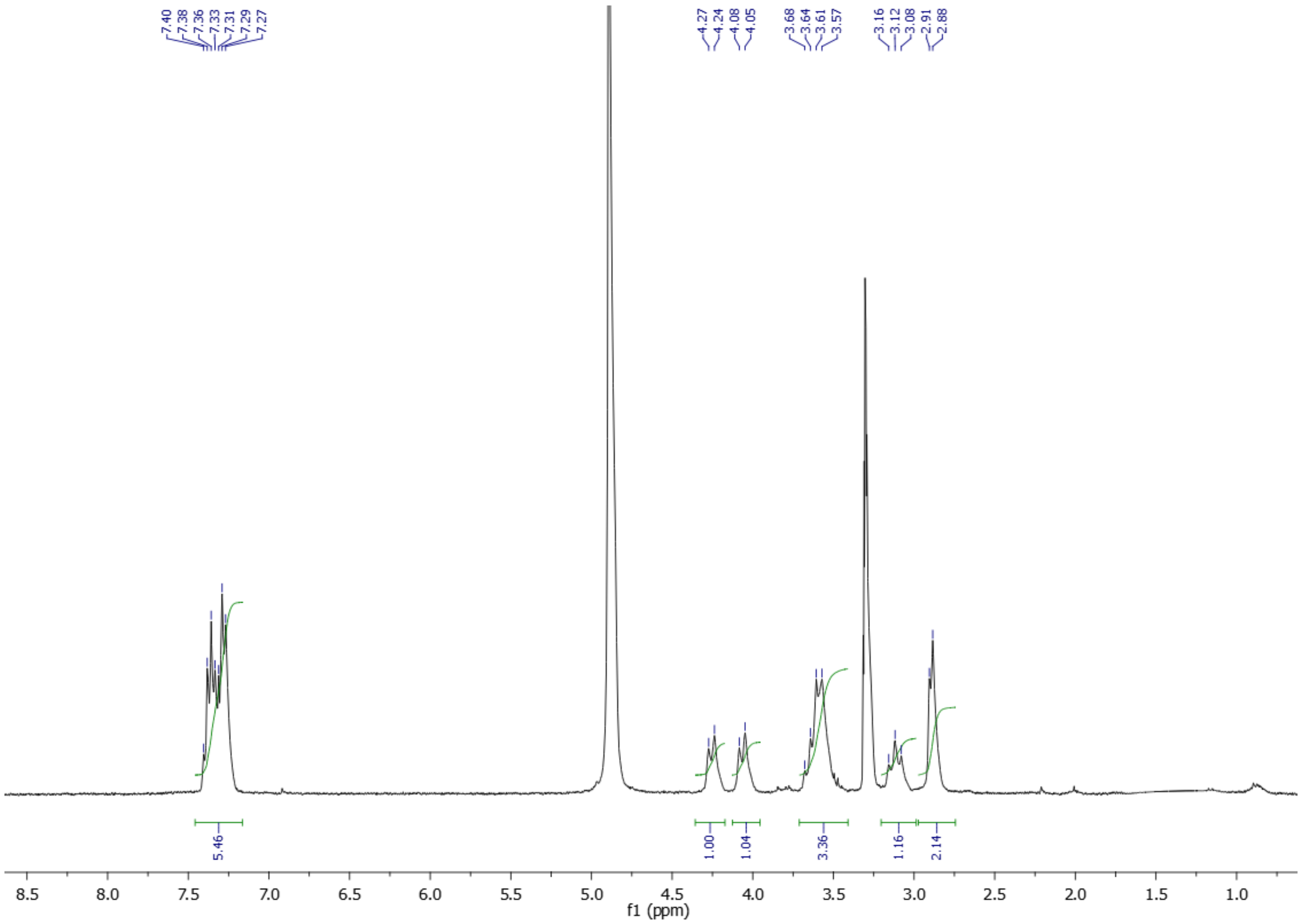

7.40 7.38 7.36 7.33 7.31 7.29 7.27
4.27 4.24 4.08 4.05
3.68 3.64 3.61 3.57
3.16 3.12 3.08 2.91 2.88
5.46
1.00 1.04 3.36 1.16 2.14
8.5 8.0 7.5 7.0 6.5 6.0 5.5 5.0 4.5 4.0 3.5 3.0 2.5 2.0 1.5 1.0
f1 (ppm)

13C-apt in CD3OD a T=300K

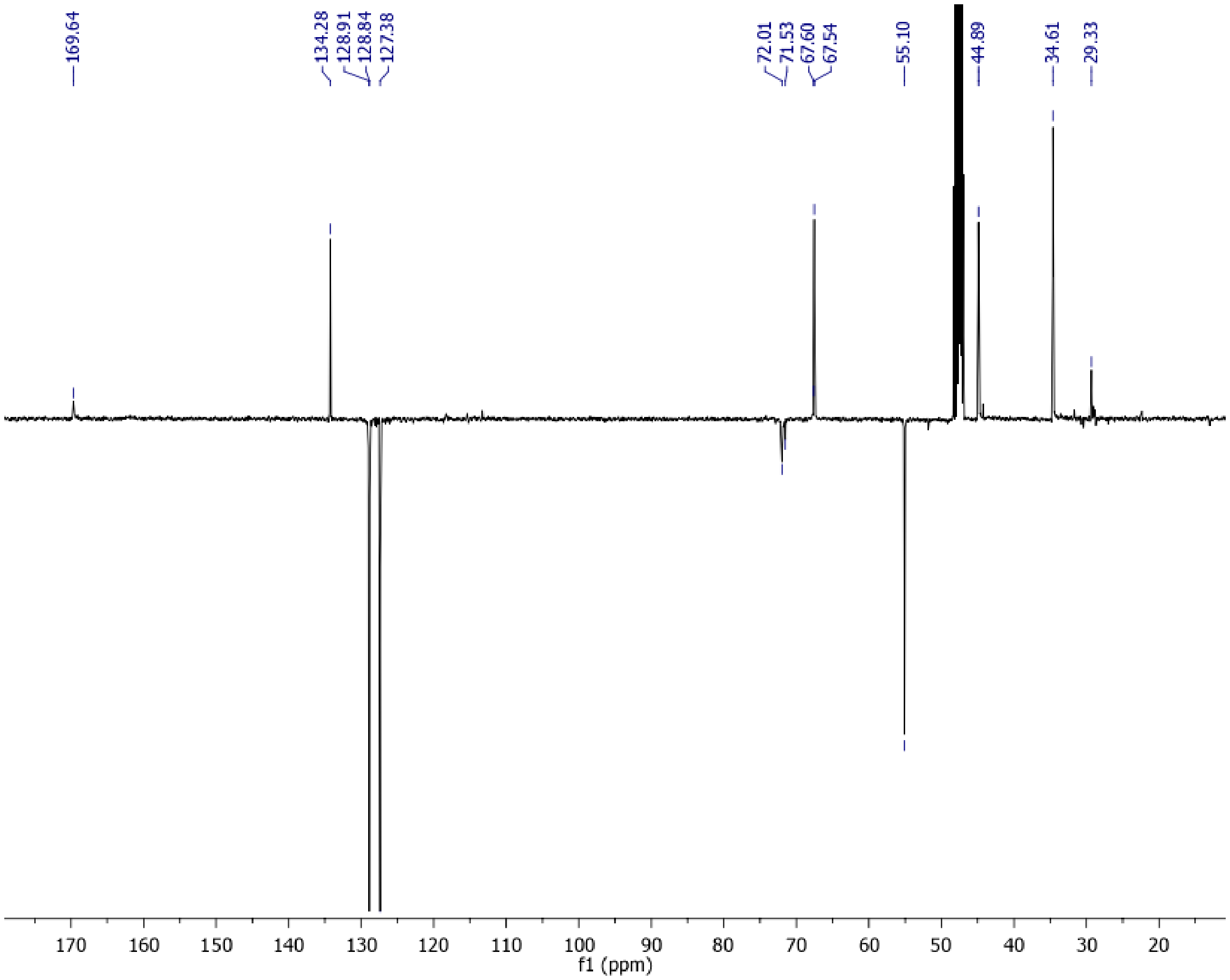

Catalyst III

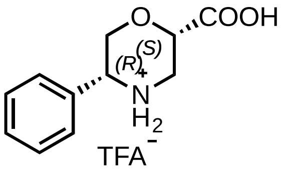

1H in CD3OD a T=300K

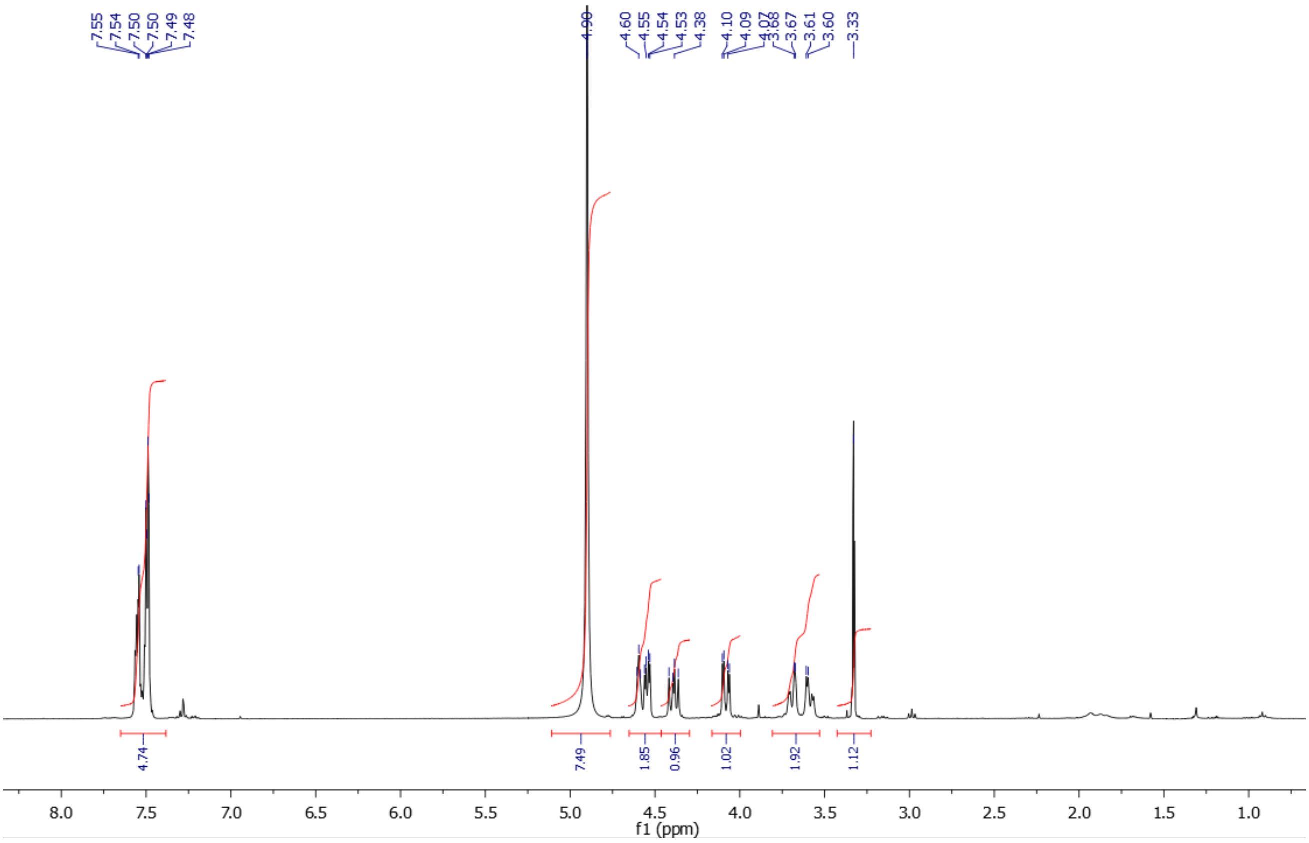

13C-apt in CD3OD a T=300K

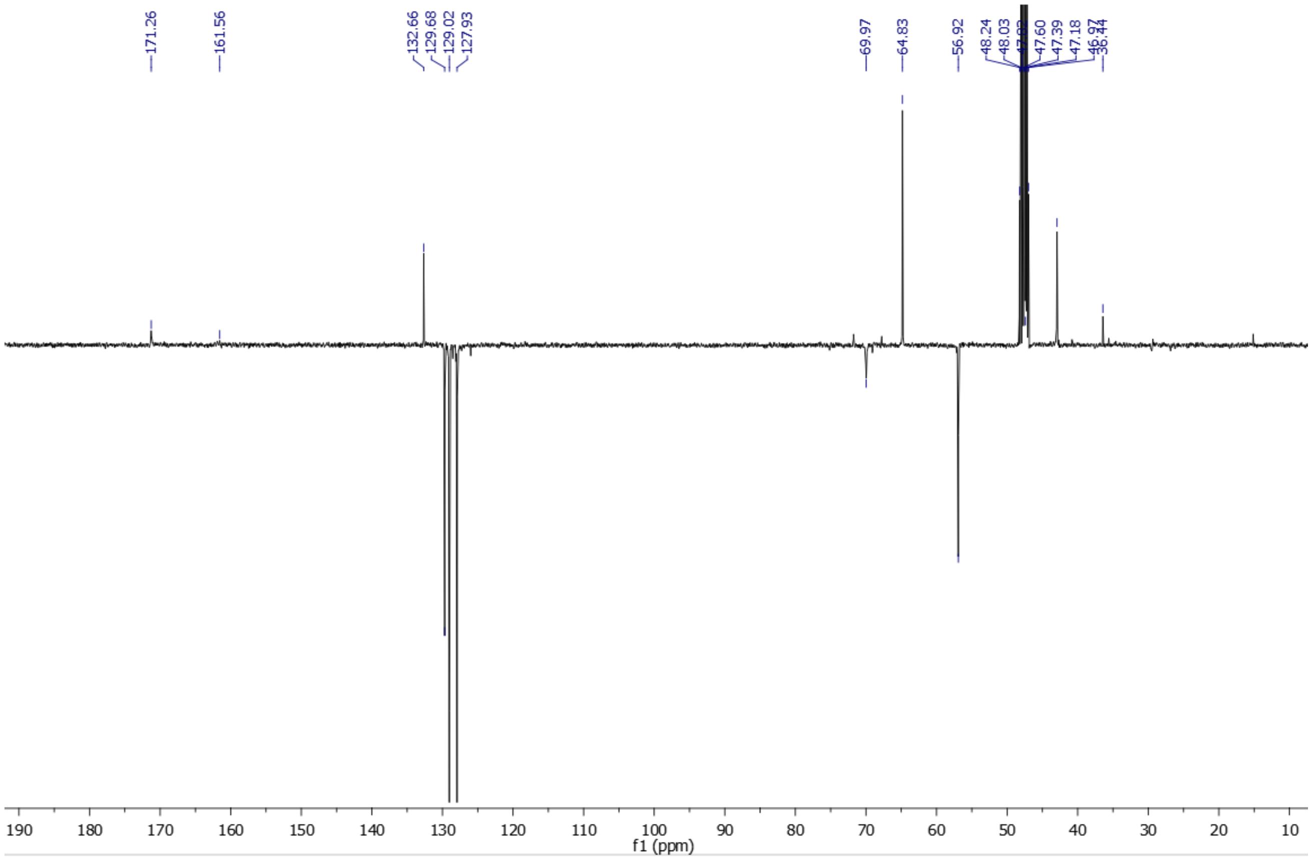

Catalyst IV

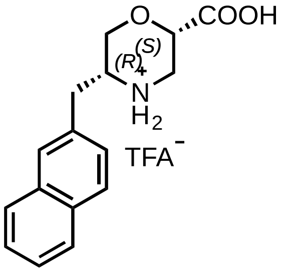
O
COOH
(S)
(R)
N
H2
TFA-

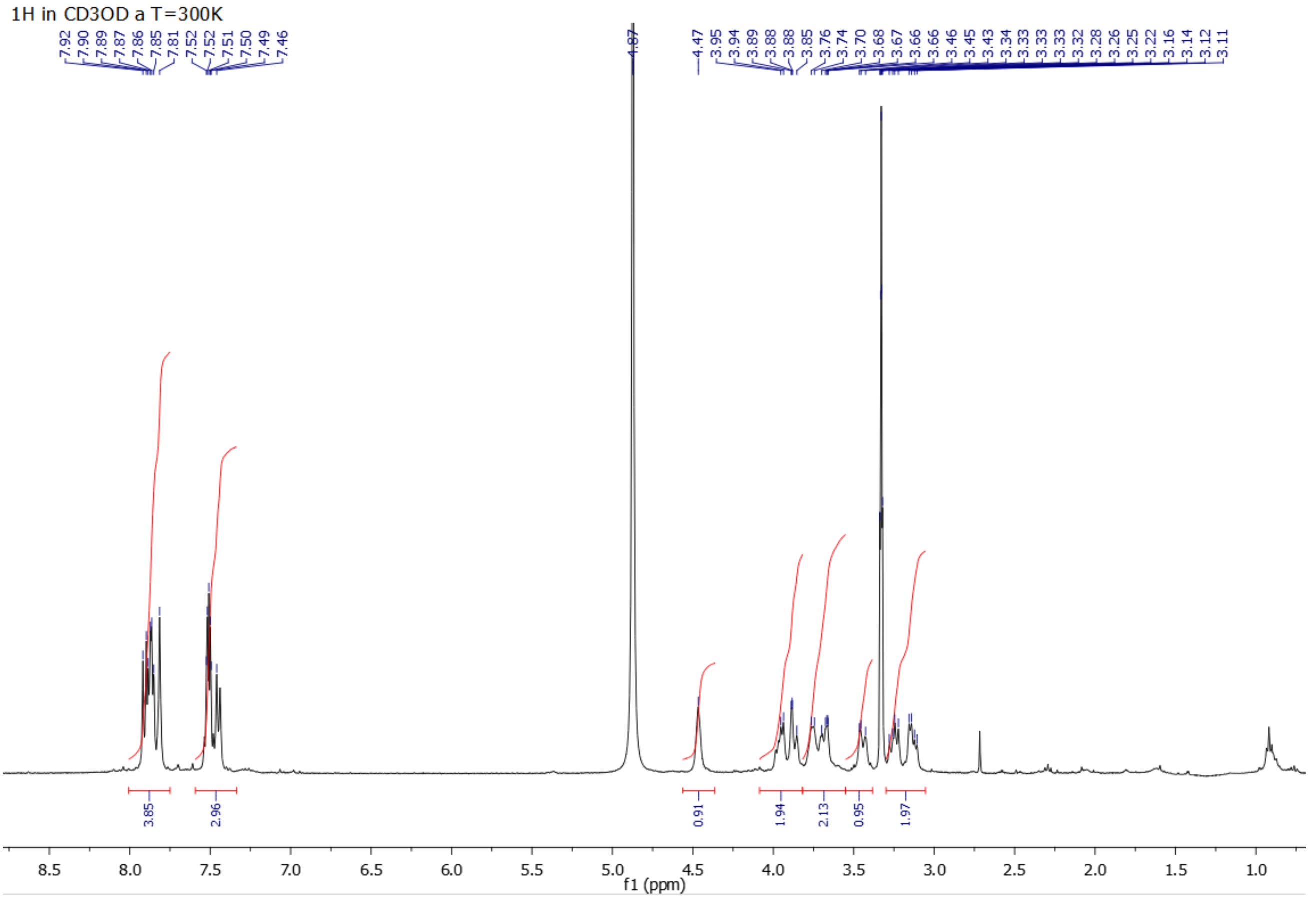
1H in CD3OD a T=300K
7.92 7.90 7.89 7.87 7.86 7.85 7.81 7.52 7.52 7.51 7.50 7.49 7.46
4.87
4.47 3.95 3.94 3.89 3.88 3.88 3.85 3.76 3.74 3.70 3.68 3.67 3.66 3.66 3.46 3.45 3.43 3.34 3.33 3.33 3.33 3.32 3.28 3.26 3.25 3.22 3.16 3.14 3.12 3.11
3.85
2.96
0.91
1.94
2.13
0.95
1.97
8.5 8.0 7.5 7.0 6.5 6.0 5.5 5.0 4.5 4.0 3.5 3.0 2.5 2.0 1.5 1.0
f1 (ppm)

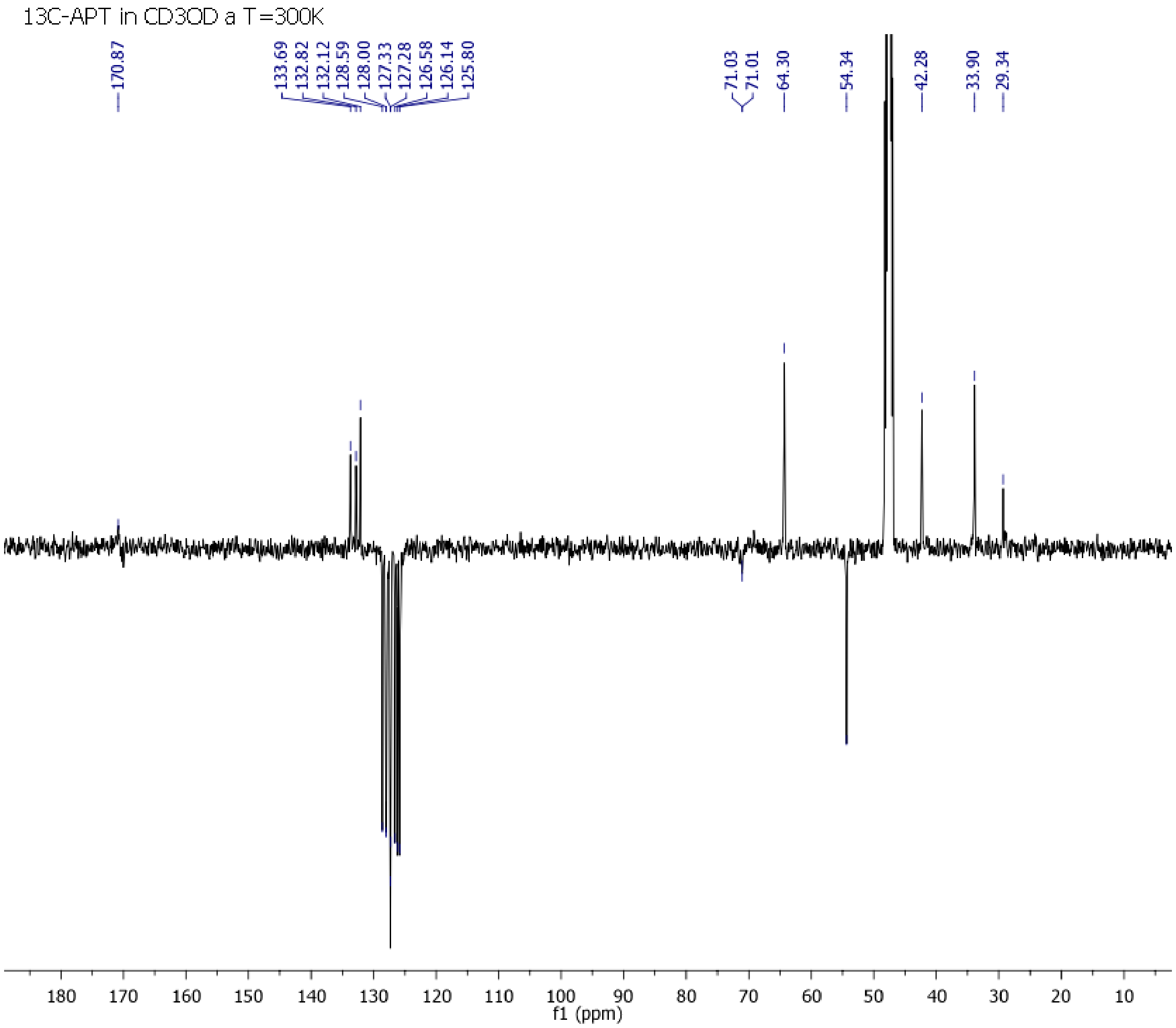
13C-APT in CD3OD a T=300K
170.87
133.69
132.82
132.12
128.59
128.00
127.33
127.28
126.58
126.14
125.80
71.03
71.01
64.30
54.34
42.28
33.90
29.34
180
170
160
150
140
130
120
110
100
90
80
70
60
50
40
30
20
10
f1 (ppm)

Copy of γ-Nitroaldehydes ^{1}H-NMR

^{1}H NMR spectra of *syn/anti* mixture of γ-nitroaldehydes obtained from **7** and **8** with catalyst **I** (isolated by flash chromatography)

Compound 9

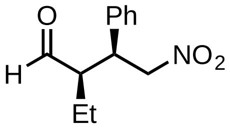

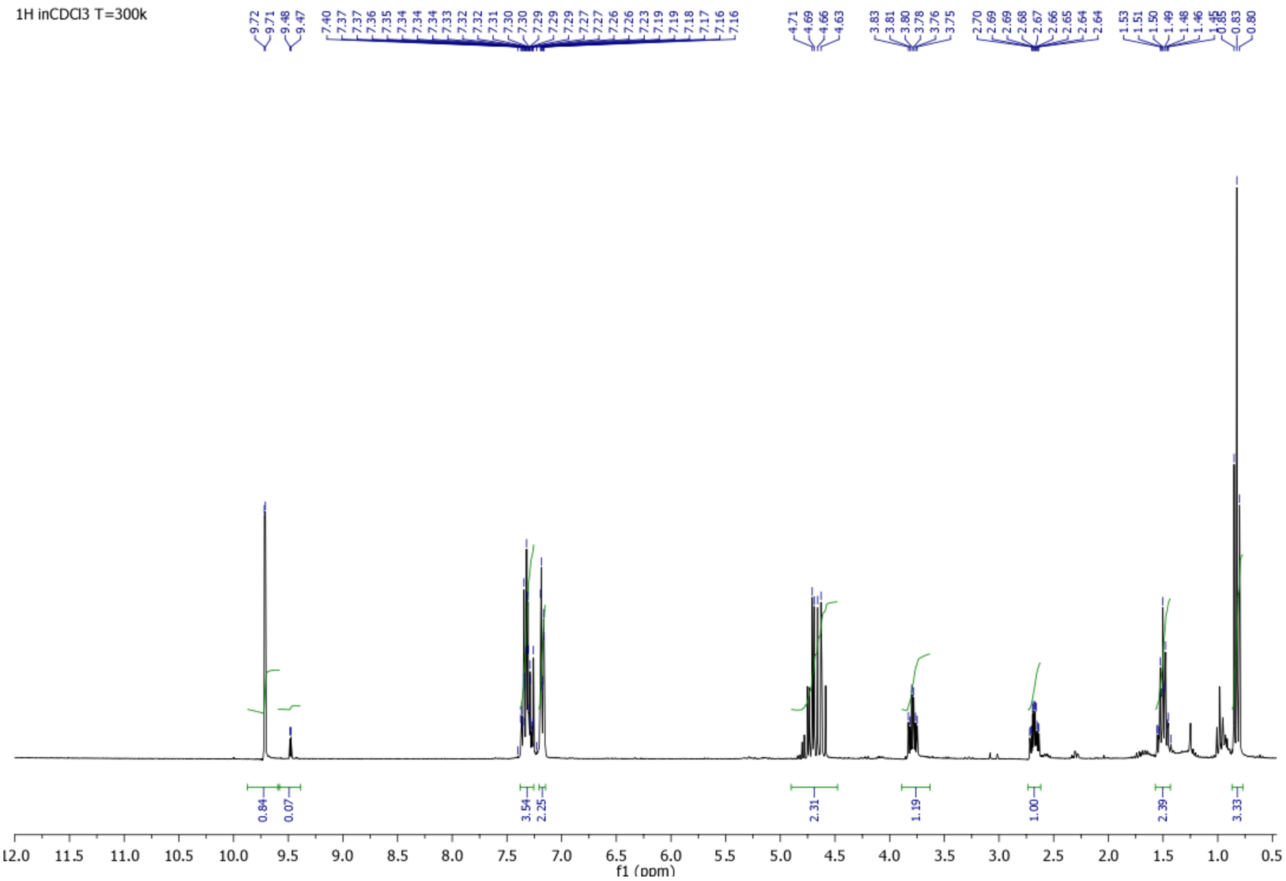

Compound 10

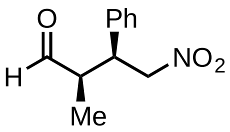

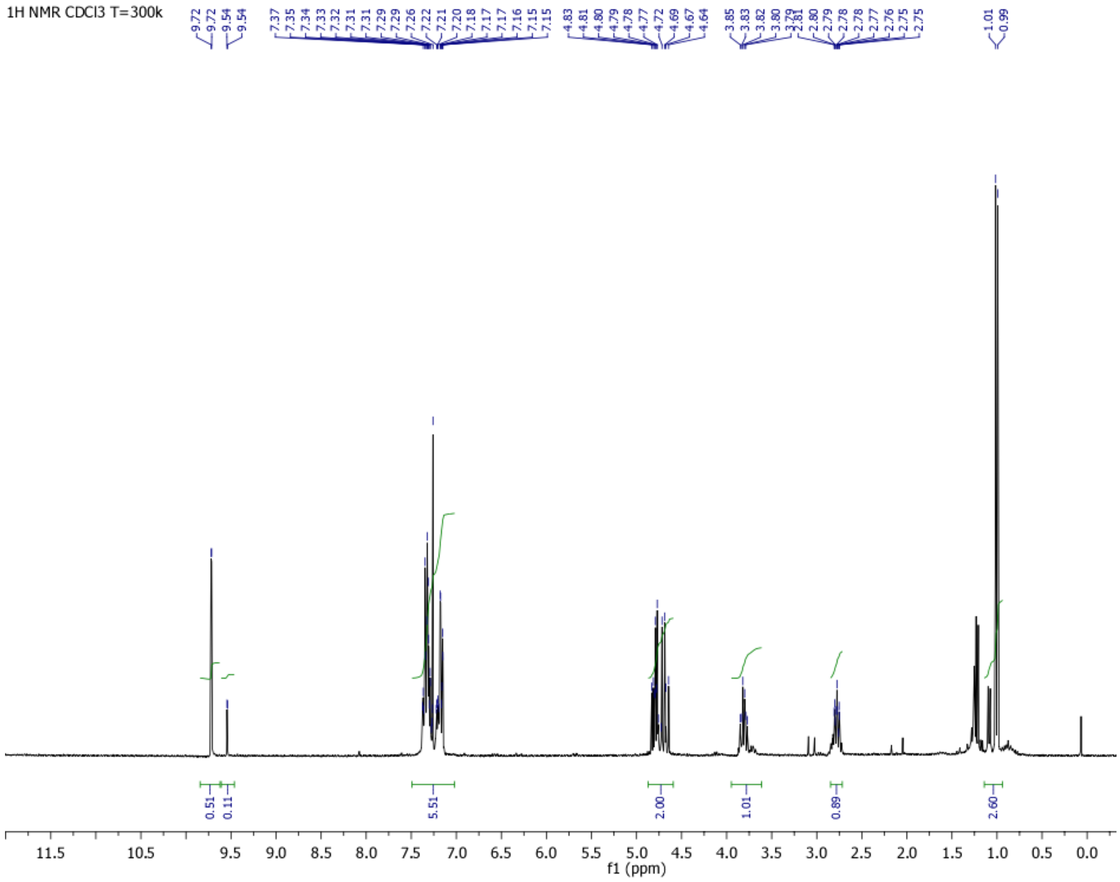

Compound 11

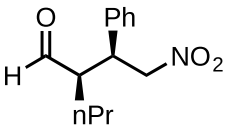

1H in CDCl3 a T=300K

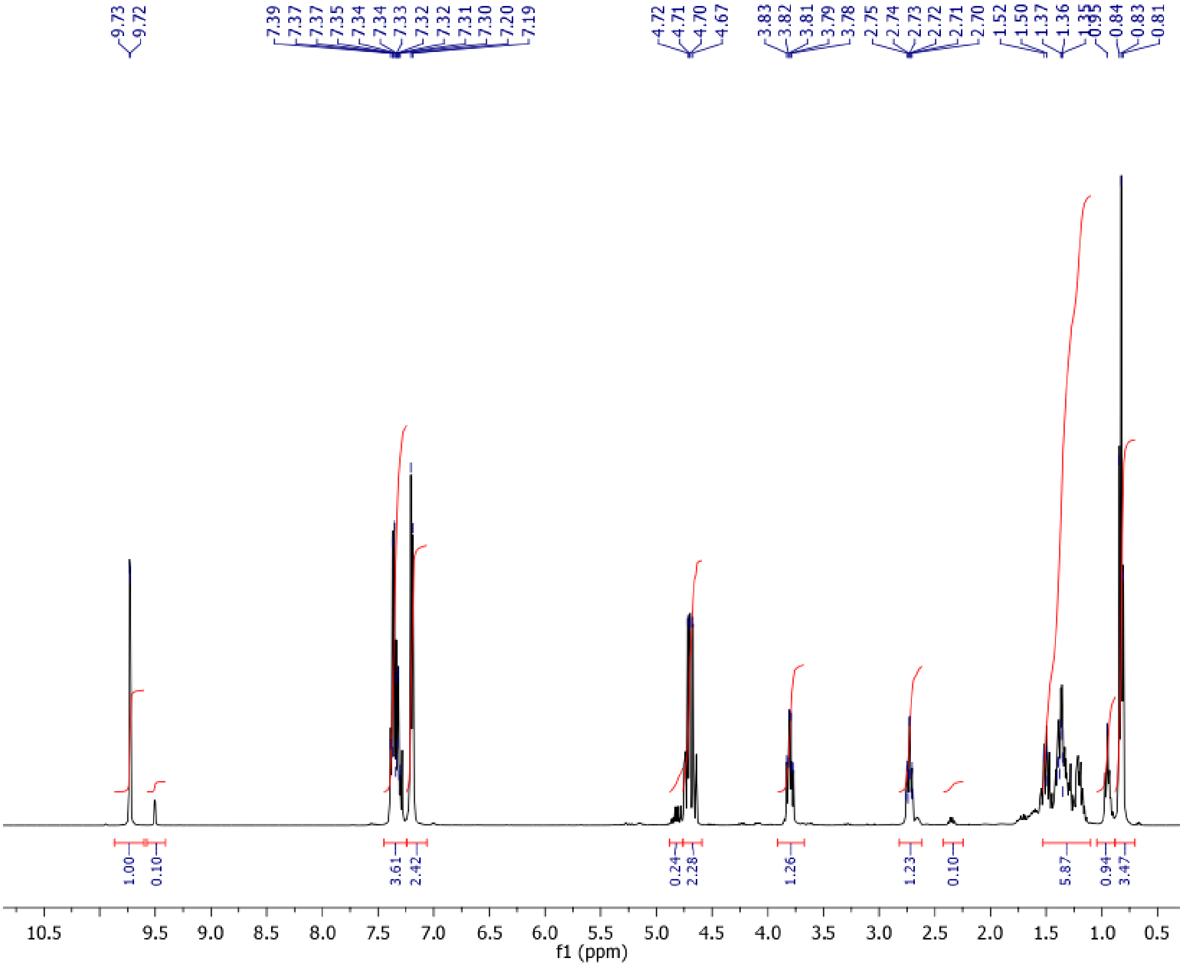

Compound 12

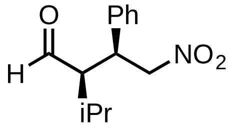
O
Ph
H
NO2
iPr

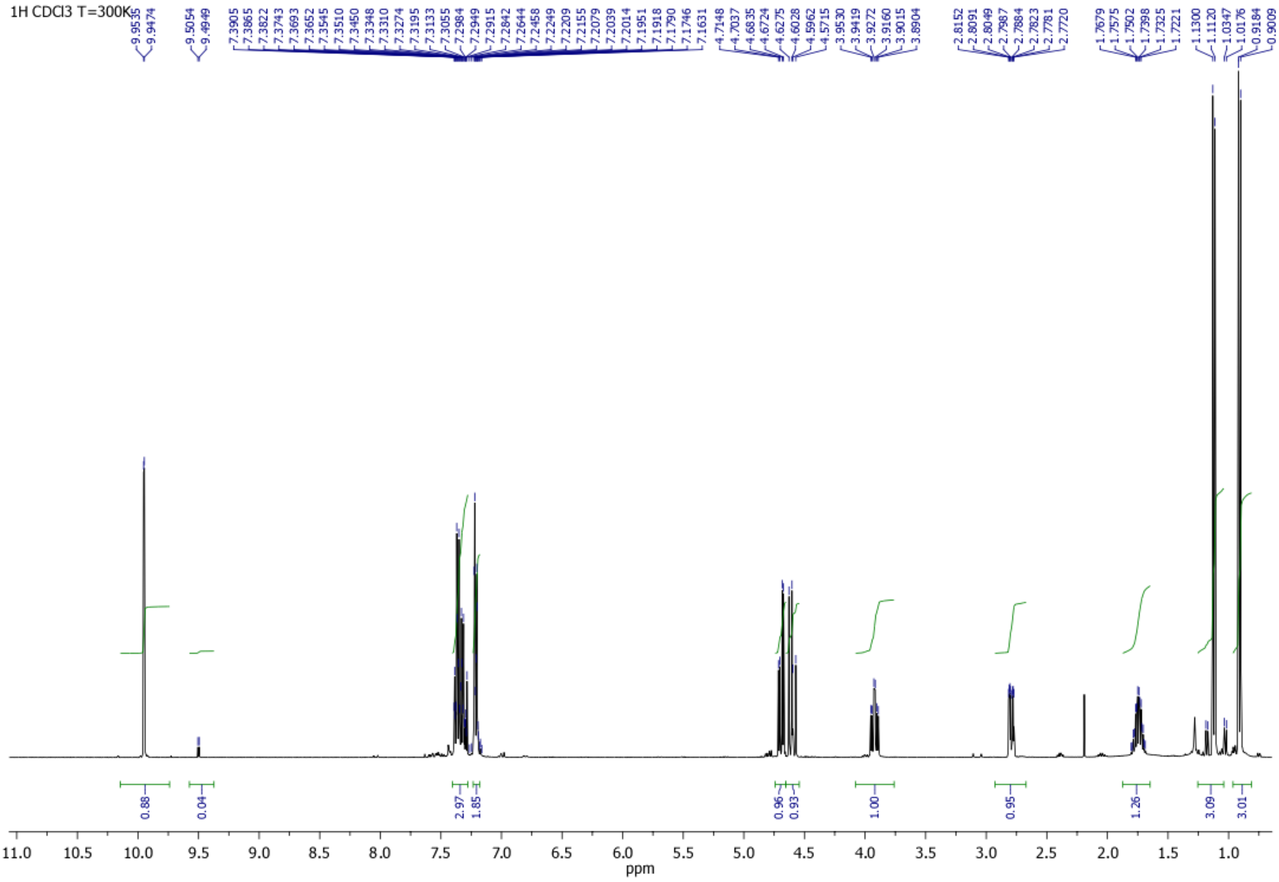
1H CDCl3 T=300K
9.9535
9.9474
9.5054
9.4949
7.3905
7.3865
7.3822
7.3743
7.3693
7.3652
7.3545
7.3510
7.3450
7.3348
7.3310
7.3274
7.3195
7.3133
7.3055
7.2984
7.2949
7.2915
7.2842
7.2644
7.2458
7.2249
7.2209
7.2155
7.2079
7.2039
7.2014
7.1951
7.1918
7.1790
7.1746
7.1631
4.7148
4.7037
4.6835
4.6724
4.6275
4.6028
4.5962
4.5715
3.9530
3.9419
3.9272
3.9160
3.9015
3.8904
2.8152
2.8091
2.8049
2.7987
2.7884
2.7823
2.7781
2.7720
1.7679
1.7575
1.7502
1.7398
1.7325
1.7221
1.1300
1.1120
1.0347
1.0176
0.9184
0.9009
0.88
0.04
2.97
1.85
0.96
0.93
1.00
0.95
1.26
3.09
3.01
11.0
10.5
10.0
9.5
9.0
8.5
8.0
7.5
7.0
6.5
6.0
5.5
5.0
4.5
4.0
3.5
3.0
2.5
2.0
1.5
1.0
ppm

Compound 13

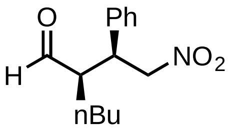
O
Ph
H
NO_2
nBu

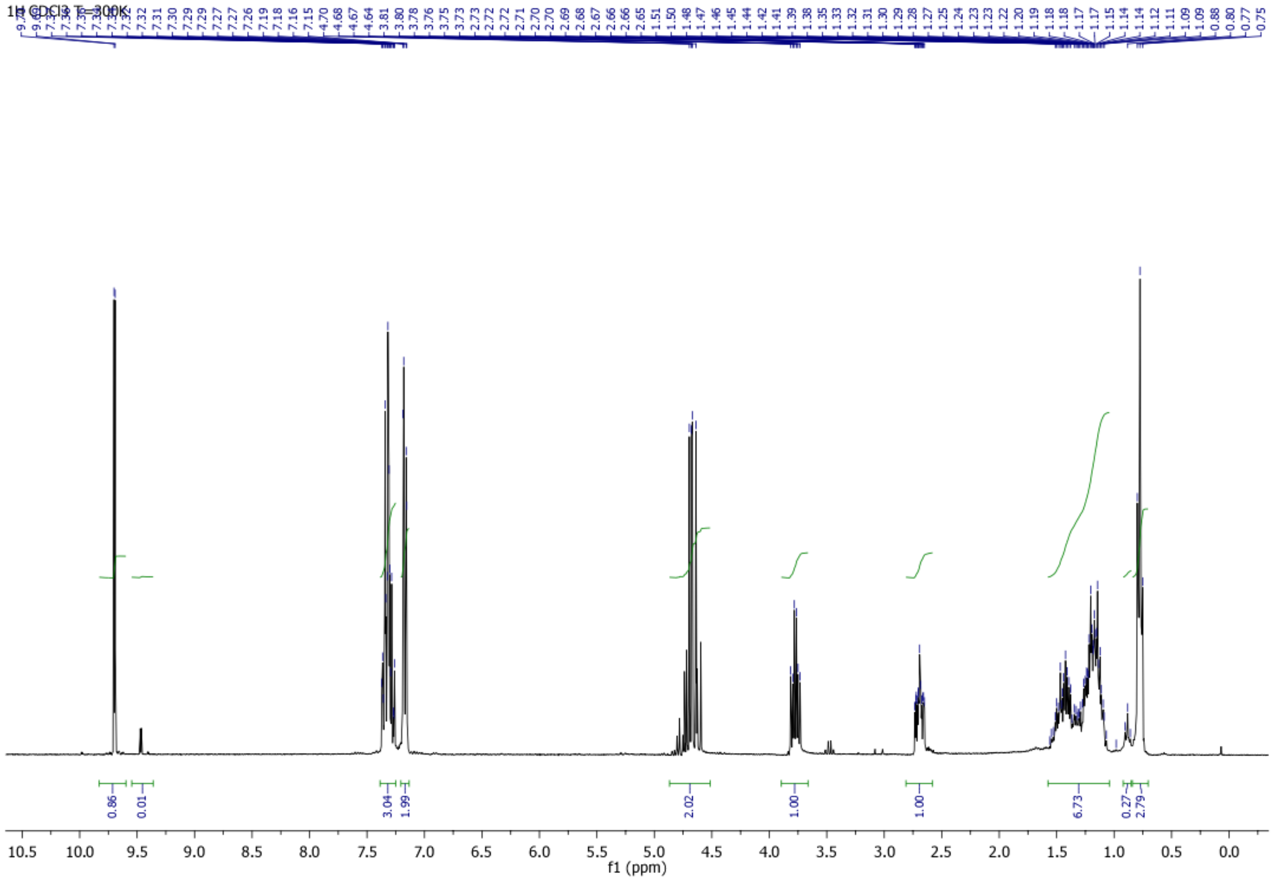
1H CDCl3 T=300K
0.86
0.01
3.04
1.99
2.02
1.00
1.00
6.73
0.27
2.79
10.5 10.0 9.5 9.0 8.5 8.0 7.5 7.0 6.5 6.0 5.5 5.0 4.5 4.0 3.5 3.0 2.5 2.0 1.5 1.0 0.5 0.0
f1 (ppm)

Compound 14

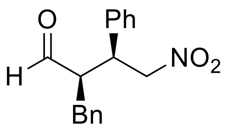

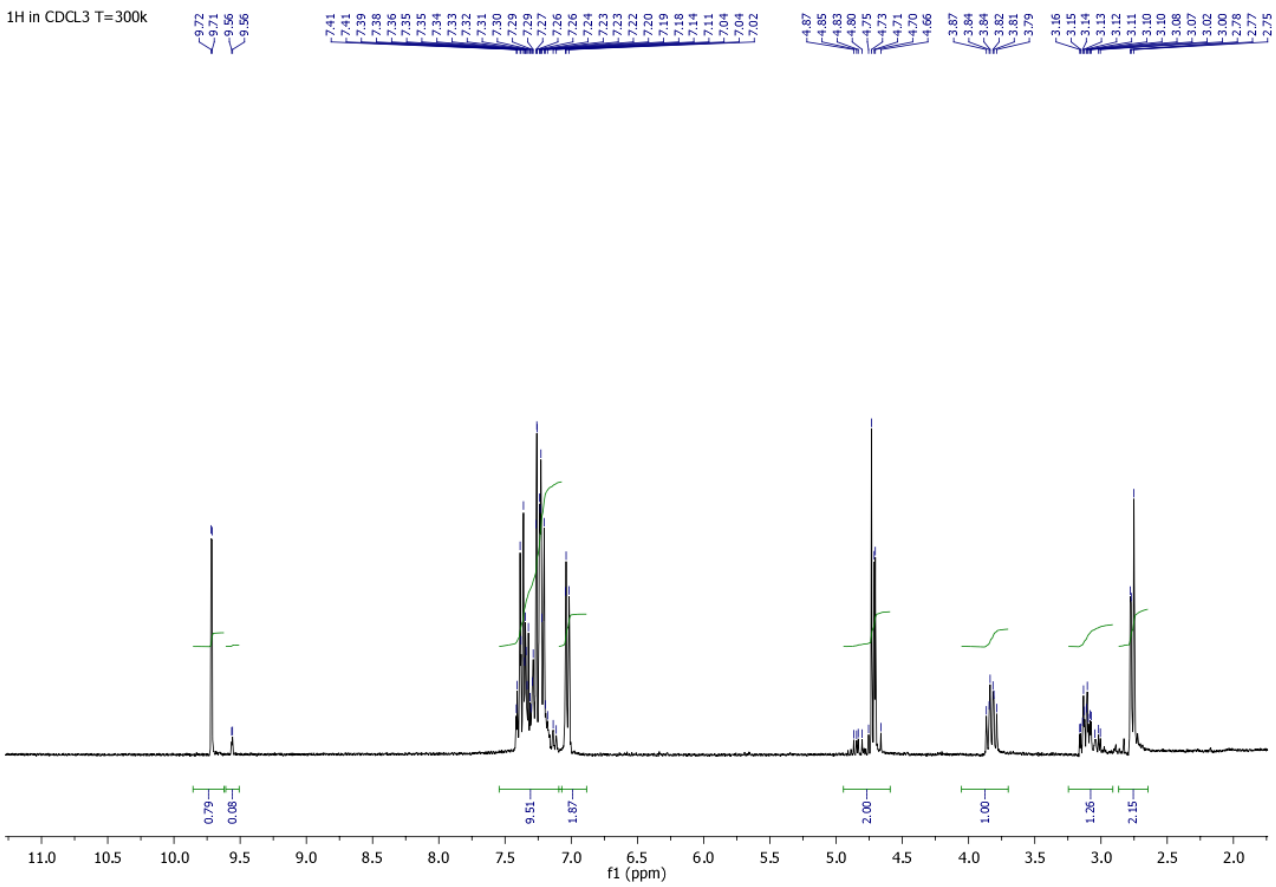

Compound 15

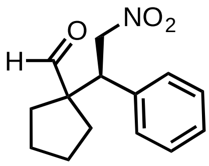

NO2
O
H

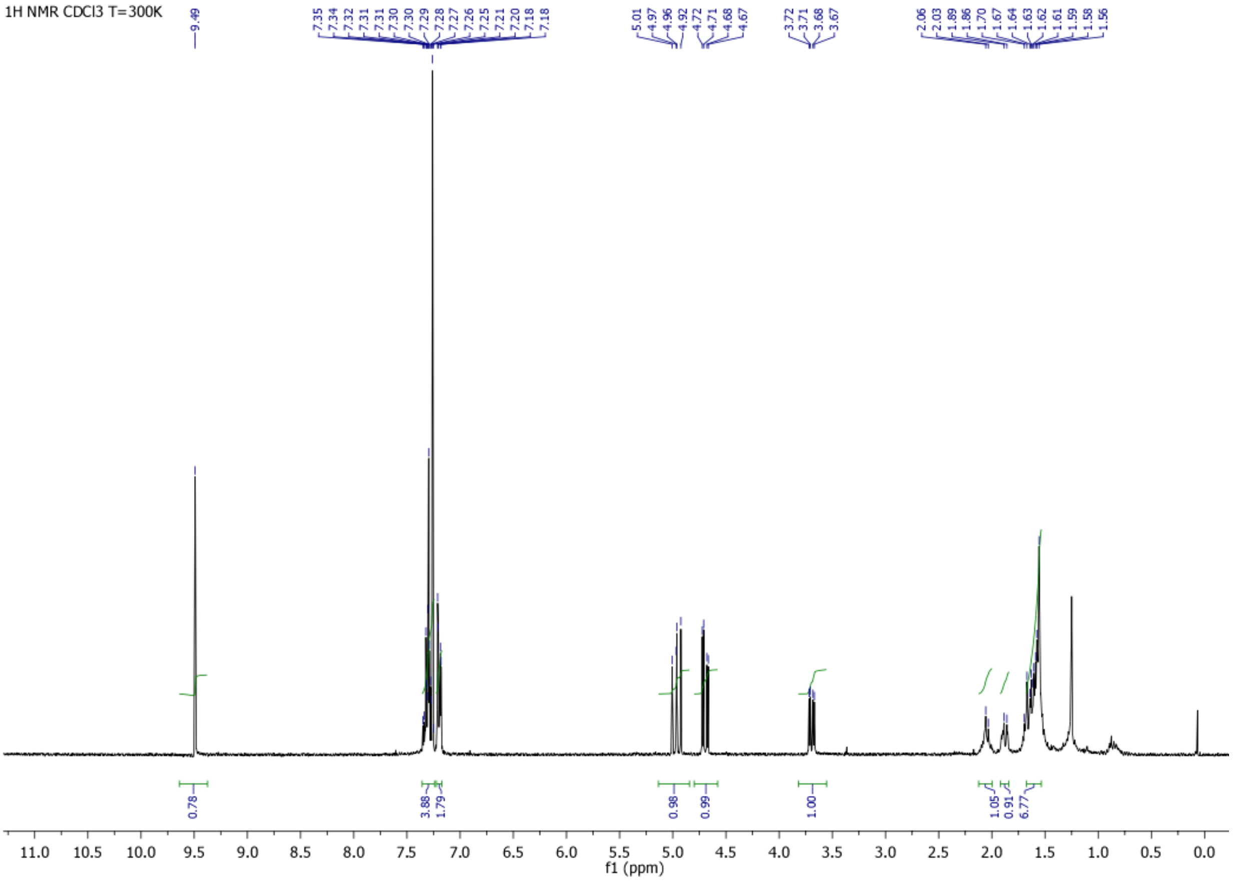

1H NMR CDCl3 T=300K
9.49
7.35 7.34 7.32 7.31 7.31 7.30 7.29 7.28 7.27 7.26 7.25 7.21 7.20 7.18
5.01 4.97 4.96 4.92 4.72 4.71 4.68 4.67
3.72 3.71 3.68 3.67
2.06 2.03 1.89 1.86 1.70 1.67 1.64 1.63 1.62 1.61 1.59 1.58 1.56
0.78
3.88 1.79
0.98 0.99
1.00
1.05 0.91 6.77
11.0 10.5 10.0 9.5 9.0 8.5 8.0 7.5 7.0 6.5 6.0 5.5 5.0 4.5 4.0 3.5 3.0 2.5 2.0 1.5 1.0 0.5 0.0
f1 (ppm)

Compound 16

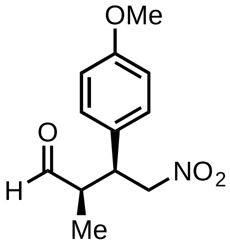

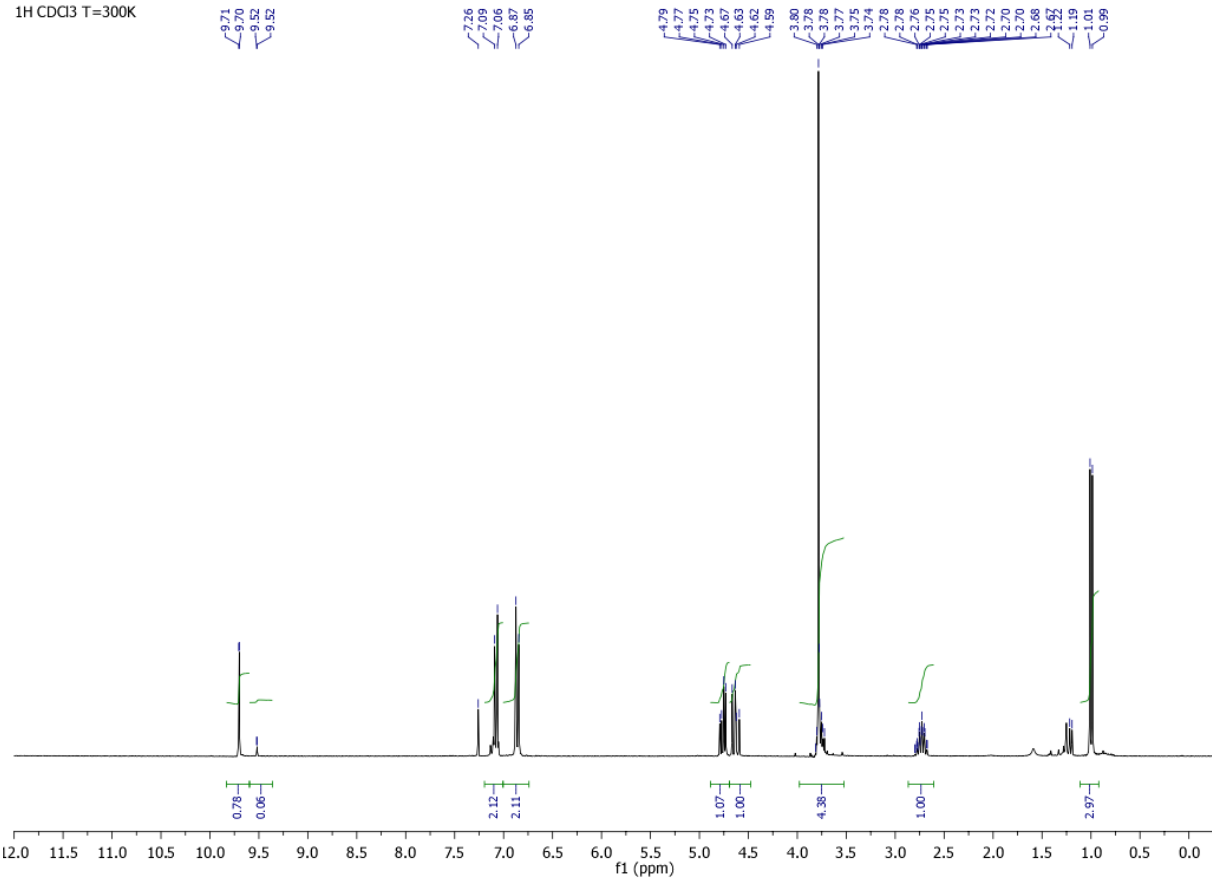

Compound 17

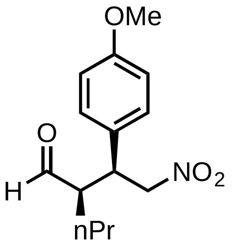

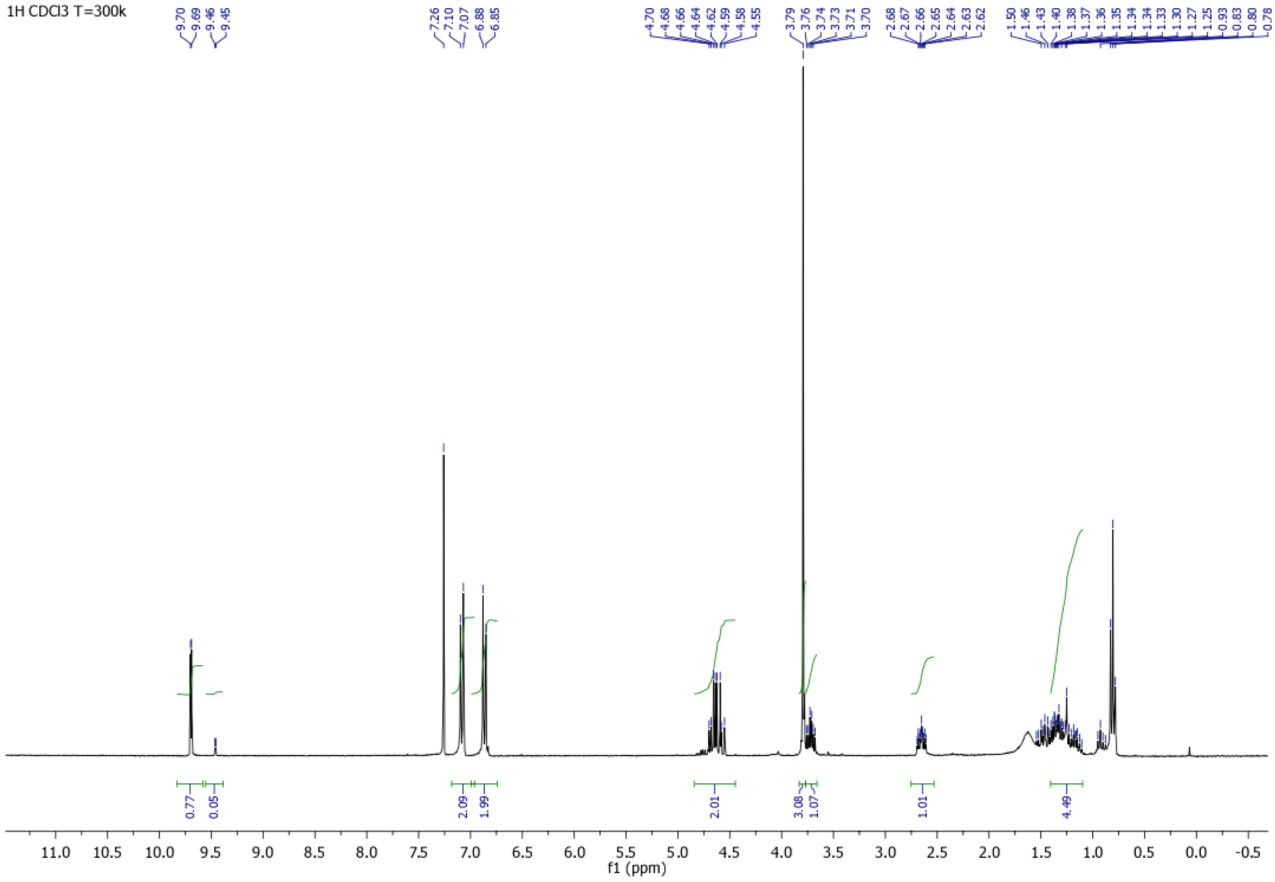

Compound 18

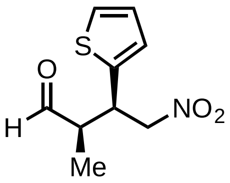

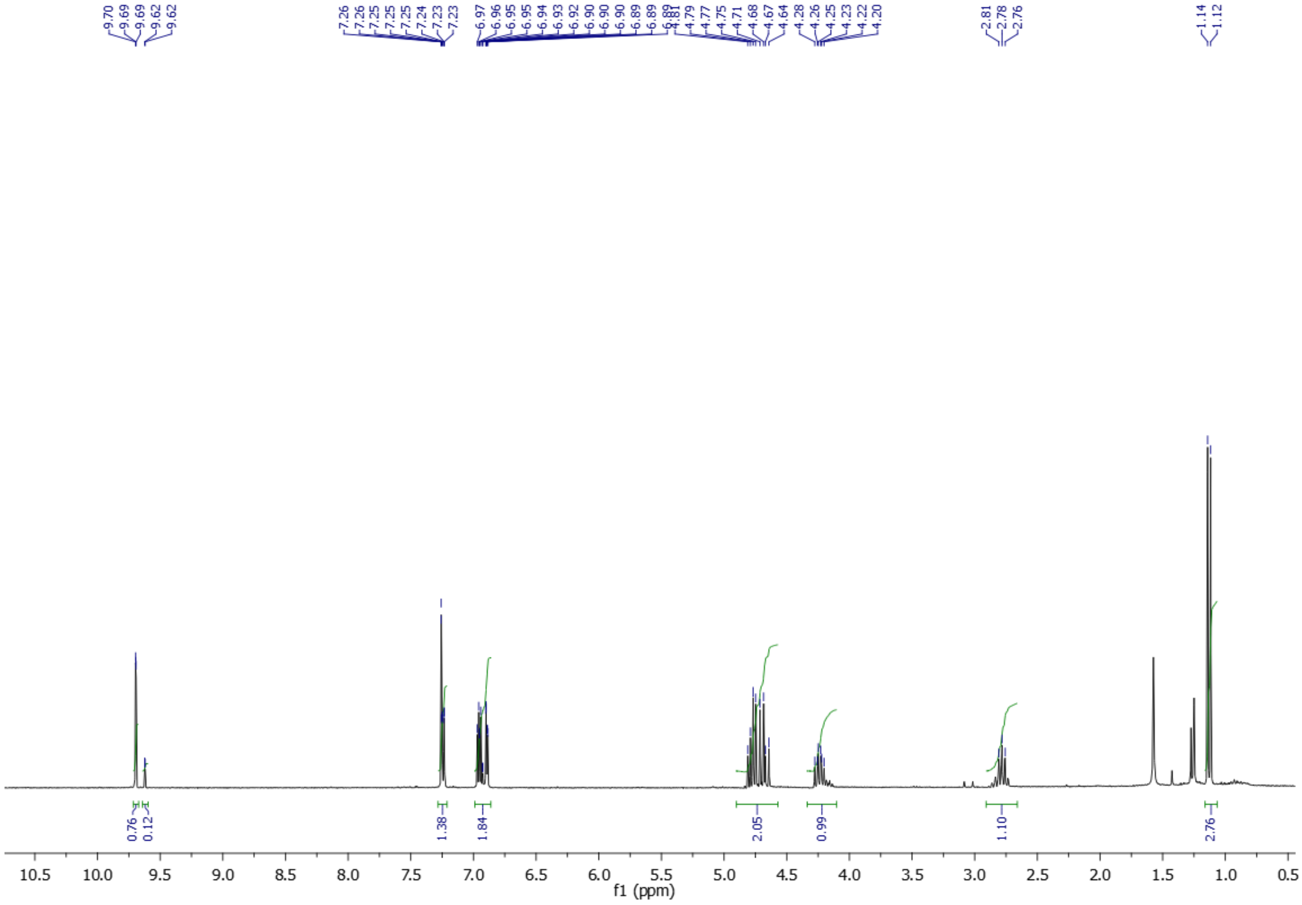
9.70
9.69
9.69
9.62
9.62
7.26
7.26
7.25
7.25
7.25
7.24
7.23
7.23
6.97
6.96
6.95
6.95
6.94
6.93
6.92
6.90
6.90
6.90
6.89
6.89
6.89
4.81
4.79
4.77
4.75
4.71
4.68
4.67
4.64
4.28
4.26
4.25
4.23
4.22
4.20
2.81
2.78
2.76
1.14
1.12
0.76
0.12
1.38
1.84
2.05
0.99
1.10
2.76
10.5
10.0
9.5
9.0
8.5
8.0
7.5
7.0
6.5
6.0
5.5
5.0
4.5
4.0
3.5
3.0
2.5
2.0
1.5
1.0
0.5
f1 (ppm)

Compound 19

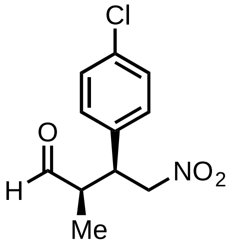

1H in CDCl3 a T=300K

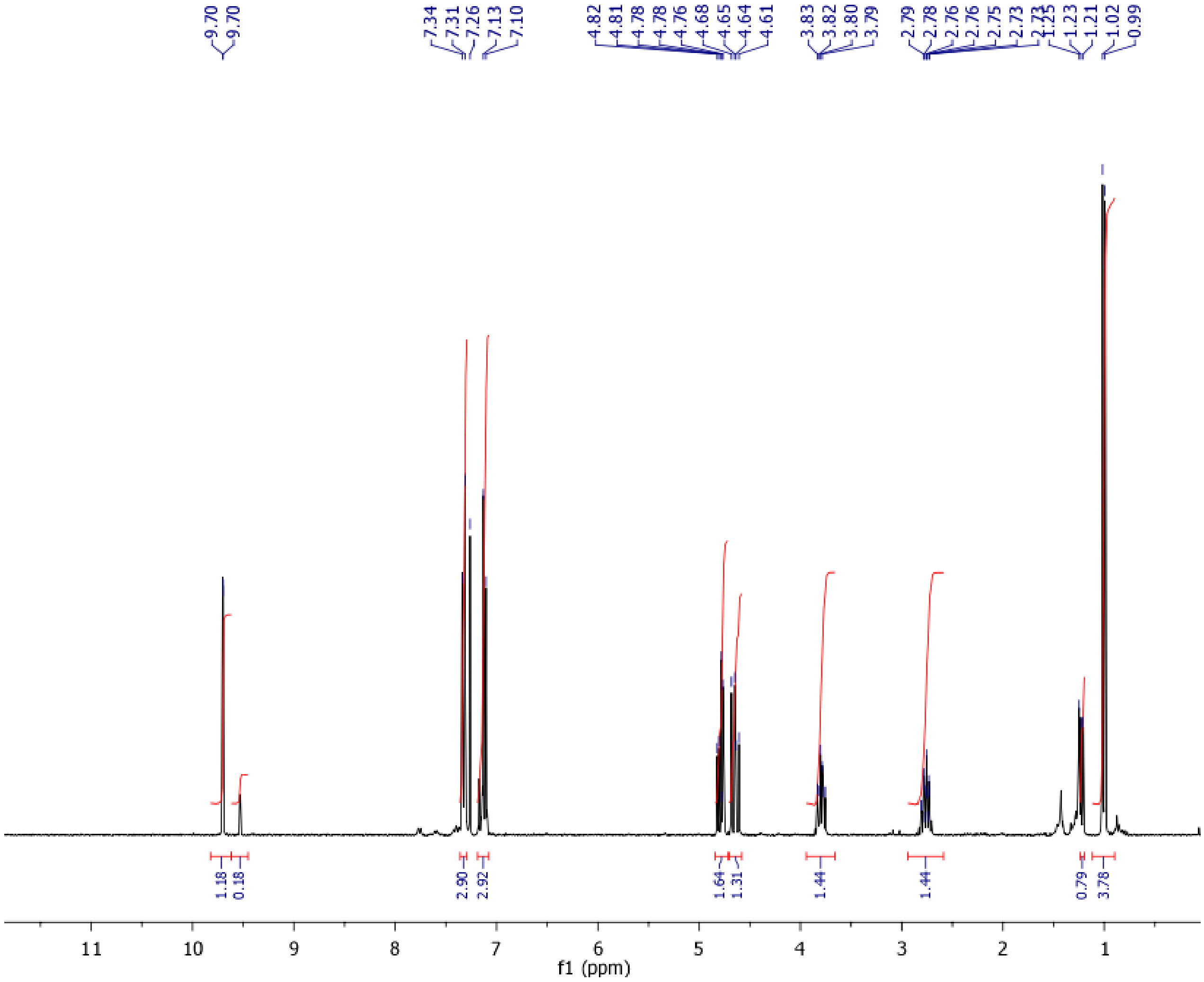

Compound 20

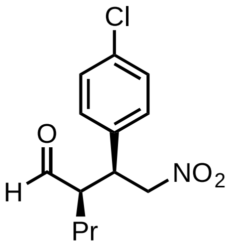

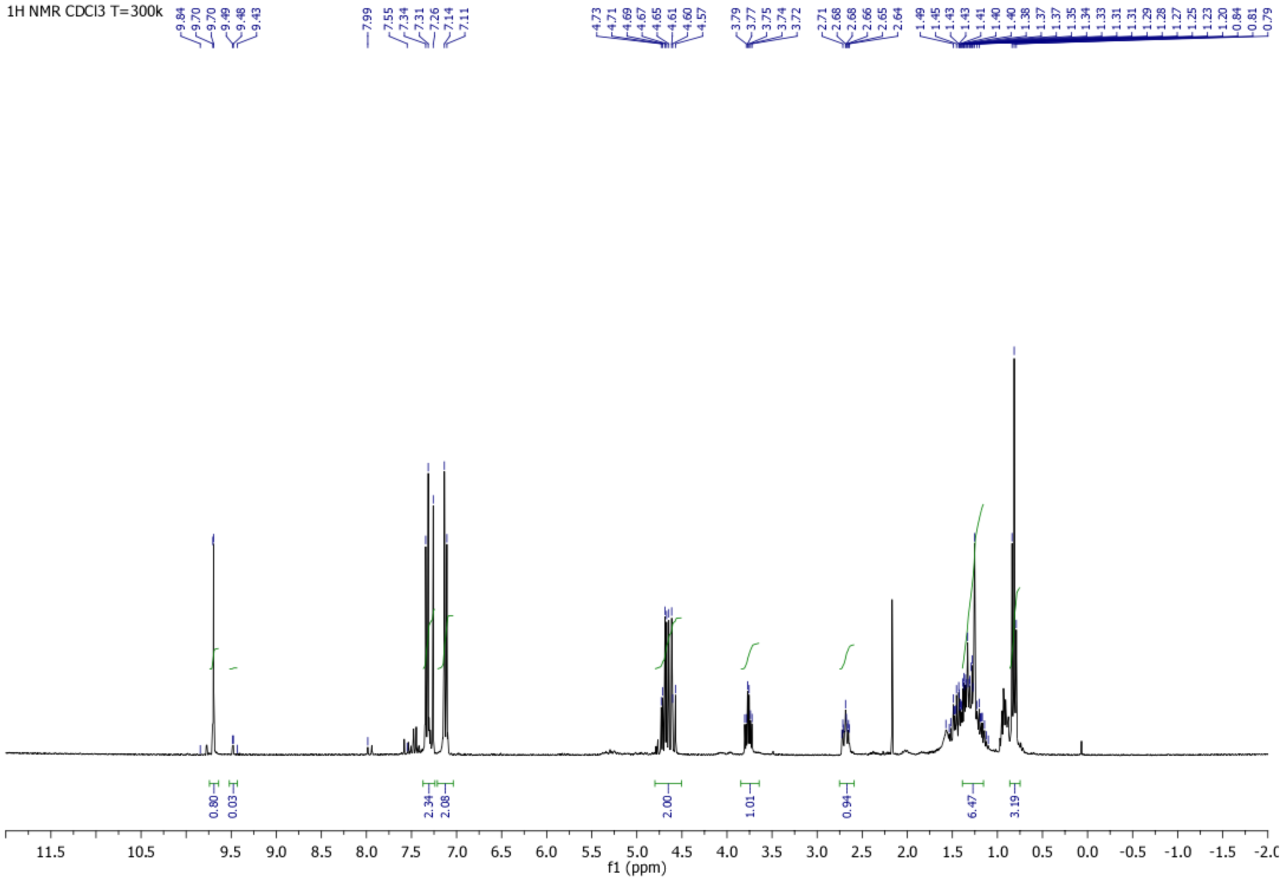

(2*R*,3*R*)-3-cyclohexyl-2-methyl-4-nitrobutanal (21)

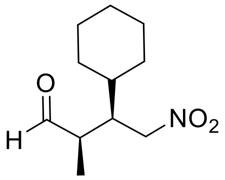

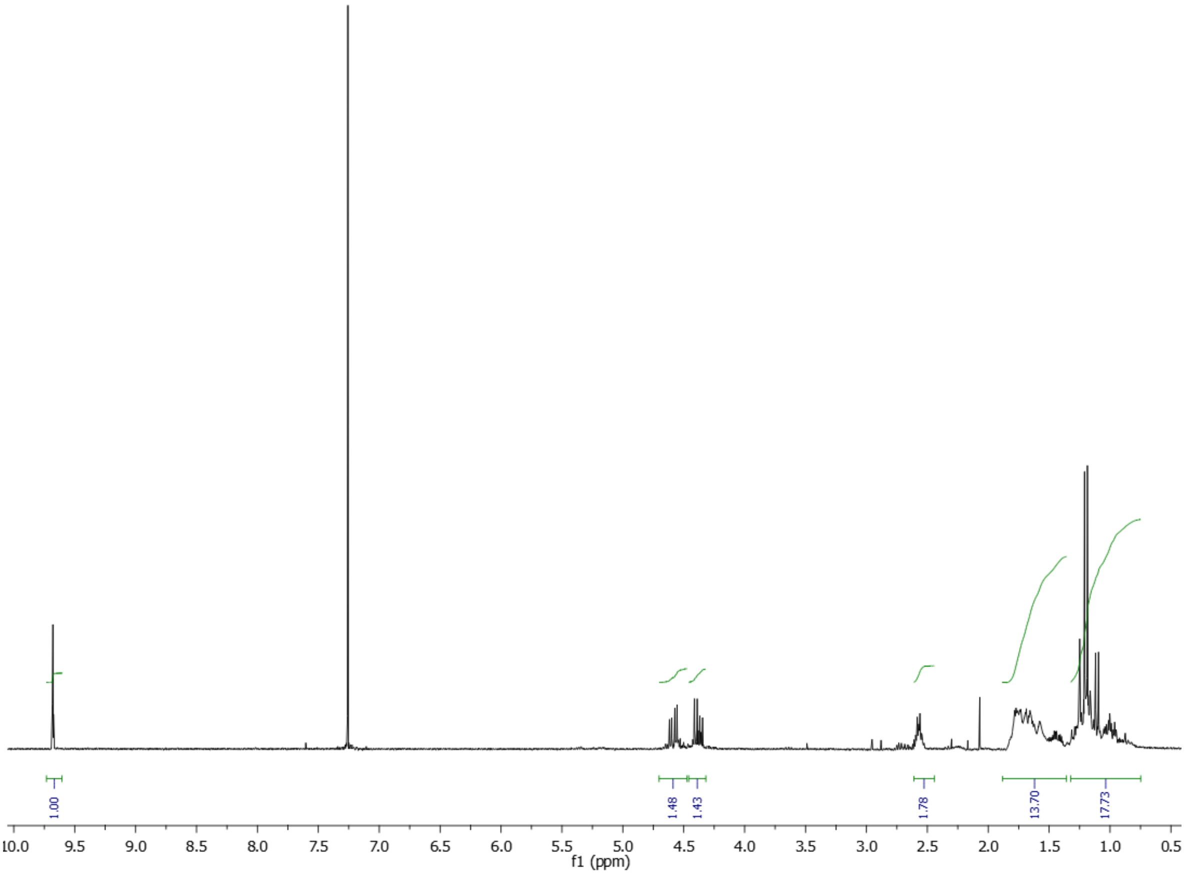

(2*R*,3*R*)-2-methyl-3-(nitromethyl)heptanal (22)

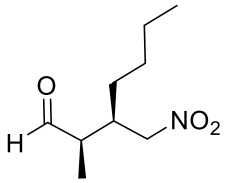

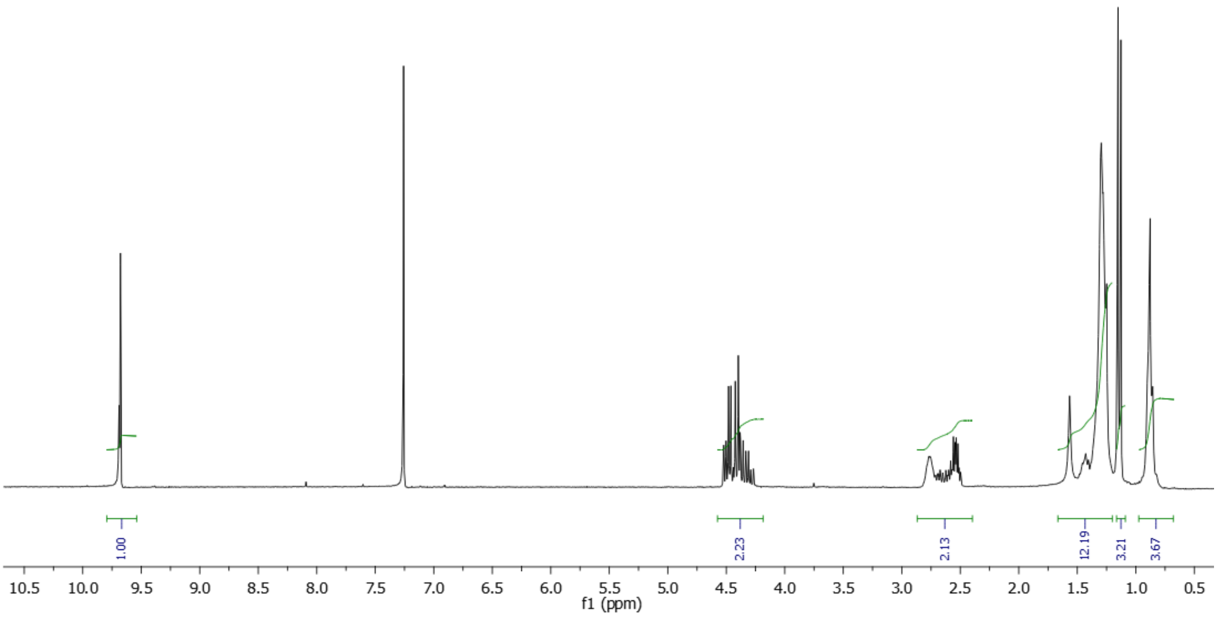

Supplement: Supplementary file 1 [file DataSheet1.PDF]
